# β-hydroxybutyrate impairs bovine oocyte maturation via pyruvate dehydrogenase (PDH) associated energy metabolism abnormality

**DOI:** 10.3389/fphar.2023.1243243

**Published:** 2023-08-11

**Authors:** Kai-Yan Zhang, Jing Guo, Cheng-Lin Zhan, Chong-Shan Yuan, Chang-Guo Min, Zhi-Qiang Li, Hong-Yu Liu, Jun Wang, Jing Zhao, Wen-Fa Lu, Xin Ma

**Affiliations:** ^1^ Key Laboratory of the Animal Production, Product Quality and Security, Ministry of Education, Jilin Agricultural University, Changchun, Jilin, China; ^2^ Jilin Provincial International Joint Research Center of Animal Breeding and Reproduction Technology, Jilin Agricultural University, Changchun, Jilin, China; ^3^ College of Animal Science and Technology, Jilin Agricultural University, Changchun, Jilin, China

**Keywords:** β-hydroxybutyrate (βHB), PDH, acetylation modification, oocyte maturation, bovine reproductive performance

## Abstract

**Background:** Ketosis is one of the most frequent and costly metabolic disorders in high-producing dairy cows, and negatively associated with the health and reproductive performance of bovine. Ketosis is mainly caused by the accumulation of ketone body β-hydroxybutyric acid and its diagnosis is based on β-hydroxybutyrate (βHB) concentration in blood.

**Methods:** In this study, we investigated the effects of βHB on bovine oocyte maturation in the concentration of subclinical (1.2 mM) βHB and clinical (3.6 mM).

**Results:** The results showed βHB disrupted bovine oocyte maturation and development capacity. Further analysis showed that βHB induced oxidative stress and mitochondrial dysfunction, as indicated by the increased level of reactive oxygen species (ROS), disrupted mitochondrial structure and distribution, and depolarized membrane potential. Furthermore, oxidative stress triggered early apoptosis, as shown by the enhanced levels of Caspase-3 and Annexin-V. Moreover, 3.6 mM βHB induced the disruption of the pyruvate dehydrogenase (PDH) activity, showing with the decrease of the global acetylation modification and the increase of the abnormal spindle rate.

**Conclusion:** Our study showed that βHB in subclinical/clinical concentration had toxic effects on mitochondrial function and PDH activity, which might affect energy metabolism and epigenetic modification of bovine oocytes and embryos.

## 1 Introduction

Ketosis is one of the most detrimental metabolic diseases in dairy cows, caused by negative energy balance (NEB), have been associated with reduced fertility in cows (Bernabucci et al., 2005). Ketone body, which includes acetoacetate, acetone, and β-hydroxybutyrate (βHB), are generated in the liver from fats by β-oxidation and circulated through the bloodstream, serving as an energy source (Newman and Verdin, 2014; Sangalli et al., 2018). βHB accounts for about 70% of the total ketone body. It is reported that there is a strong correlation between βHB levels in follicular fluid and serum (Leroy et al., 2004). Therefore, serum βHB is reflected in the follicular fluid, in which the oocyte matures. The increase in intrafollicular concentrations of βHB compromises bovine follicular growth (Missio et al., 2022). In addition, βHB induces the reduction of oocyte maturation, cleavage, and blastocyst production rates in ovine, which might attribute to the impaired oocyte quality (Nandi et al., 2018). However, the effects of βHB on bovine oocytes maturation and energy metabolism are still unknown.

Oocyte development competence is important for fertilization and reproductive efficiency. Mitochondria are believed to play essential roles during oocyte maturation, fertilization, and embryo development, and act as a hub in cellular signaling, energetics, and redox balances (Lonergan et al., 2001). Mitochondria have a crucial part in producing energy for oocyte maturation and embryo development throughout precise cellular functions comprising Ca^2+^ homeostasis regulation, glycolysis, amino acid, and fatty acid metabolism, and regulation of apoptosis (Belli et al., 2021). The quality of the oocyte is directly determined by the normal functionality of the mitochondria. Studies have shown that mitochondrial functions is a key determinant of oocyte developmental potential (Cecchino et al., 2018; Labarta et al., 2019) and that mitochondrial dysfunction leads to meiotic defects in oocytes (Grindler and Moley, 2013; Zhang et al., 2019; Lentscher et al., 2021) and the arrest of pre-implantation embryos *in vitro* (Belli et al., 2021).

Meanwhile, the mitochondrial matrix is the main site of the tricarboxylic acid (TCA) cycle, and it is also the key checkpoint of the link between oxidative phosphorylation and the TCA cycle. Mammalian oocytes lack the ability to take up glucose (Sui et al., 2020), pyruvate is the primary metabolic substrate used by oocyte mitochondria, and may also be important in zygotic genome activation (Nagaraj et al., 2017). Pyruvate is supplied to oocytes by cumulus cells, readily taken up into the oocyte and the mitochondrial matrix via monocarboxylate transporters (MCTs), and converted to ATP and acetyl-CoA by activation of pyruvate dehydrogenase (PDH) (Nandi et al., 2017; Liu et al., 2019). Thus, mitochondrial PDH is an essential metabolic enzyme for oocyte maturation. PDH is a multienzyme complex, the first enzyme of the TCA cycle and acts as a mediator between glycolysis and the TCA cycle (Nie et al., 2020). During the oocyte maturation, the activity of the PDH complex increases and produce sufficient ATP, Acetyl-CoA, and metabolites for meiotic maturation (Johnson et al., 2007; Hou et al., 2015). Ketone bodies are small lipid-derived molecules that act as a circulating energy source for tissues during fasting or prolonged exercise (Newman and Verdin, 2014). In addition, BHB also influence the TCA cycle to produce ATP (Youm et al., 2015; Hwang et al., 2022). Pyruvate is not only the direct substrate of the TCA cycle, but also the energy substrate of oocyte maturation (Herta et al., 2022; Imanaka et al., 2022). We hypothesized that BHB affects the ATP production via regulating pyruvate metabolism during oocyte maturation.

In the present study, we explored the effects of βHB on mitochondria functions and energy metabolism during bovine oocyte maturation. The results illustrated that the potential negative effects of βHB were mediated by oxidative stress, apoptosis, PDH activity inhibition, and global acetylation reduction, provided new insights into the negative effects of ketosis on bovine reproductive efficiency.

## 2 Materials and methods

### 2.1 Antibodies and chemicals

ROS detection kit was purchased from Beyotime Biotechnology (S0033S, Beyotime, China), mitochondrial membrane potential and apoptosis detection kit were purchased from Beyotime Biotechnology (C1071, Beyotime, China), ATP assay kit was purchased from Thermo Fisher Scientific (A12410; BODIPY- ATP; United States), DL-β-Hydroxybutyric acid sodium salt (H6501-5G, Oakville, ON, Canada). Unless otherwise indicated, the related reagents of oocyte maturation culture system were purchased from Sigma aldrich or Life Technologies Corporation.

### 2.2 Oocyte collection and maturation

As previous study (Halstead et al., 2020), ovaries were procured from a local abattoir and transported to the laboratory in saline solution for 4 hours, complying with Jilin Agricultural University relevant ethical regulation for animal testing and research. Follicles measuring 3–8 mm were aspirated to obtain cumulus oocyte complexes (COCs). Only COCs with over the 3 layers of granulosa cells were selected for maturation. These were washed in collection Medium 199 (2230823, Life, United States of America) with 3% Fetal Bovine Serum (FBS, Sangon Biotech, China) and transferred to maturation Medium 199 (11150059, Life, United States) supplemented with ALA-glutamine (0.1 mM), Estradiol (10 ng/mL), cysteamine (0.1 mM), sodium pyruvate (0.2 mM), EGF (50 ng/mL), FSH (0.5 μg/mL), LH (0.5 μg/mL), cysteamine (0.1 mM), and 10% FBS (C04002, Gemini Bio, United States). COCs were in the maturation medium (with/without βHB) at 38.5°C, 5% CO_2_, and 100% humidity for 22 h. 360 mM βHB in TCM199 was diluted to concentration of 1.2 and 3.6 mM with maturation medium. The oocytes were randomly divided into the following three groups: 0, 1.2, and 3.6 mM βHB. And a solution of 100 mM DCA in TCM199 was diluted to concentration of 2 mM with maturation medium. The DCA was supplemented into maturation medium together with oocytes, βHB was supplemented 2 hours later. And the oocytes were randomly divided into the following two groups: 3.6 mM βHB and 2 mM DCA+3.6 mM βHB.

### 2.3 *In vitro* fertilization and embryo culture

*In vitro* fertilization and embryo culture are performed as previous study (Halstead et al., 2020). After COCs were matured for 22 h, MII oocytes were washed in SOF-IVF medium (107.7 mM NaCl, 7.16 mM KCl, 1.19 mM KH_2_PO_4_, 0.49 mM, MgCl_2_, 1.17 mM CaCl_2_, 5.3 mM sodium lactate, 25.07 mM NaHCO_3_, 0.20 mM sodium pyruvate, 0.5 mM fructose, 1X nonessential amino acids, 5 μg/mL gentamicin, 10 μg/mL heparin, 6 mg/mL fatty acid-free BSA) and transferred to drops of SOF-IVF medium under mineral oil. Frozen semen was thawed, then the percoll separation of spermatozoa was performed. Frozen spermatozoa obtained from a bull were thawed in a 37 °C water bath for 30 s, layered on the percoll density gradient (45% and 90%), and centrifuged at 700 g for 15 min. The supernatant was discarded, and the sperm pellet was resuspended with SOF-IVF medium to obtain a concentration of 106 sperms/mL were added to drops with MII oocytes, which were incubated at 38.5°C for 12–18 h. Zygotes were removed from the fertilization medium, and cumulus cells were removed by vortex 3 min in the SOF-HEPES medium (107.7 mM NaCl, 7.16 mM KCl, 1.19 mM KH_2_PO_4_, 0.5 mM fructose, 4 mM NaHCO_3_, 0.33 mM sodium pyruvate, 21 mM HEPES, 1 mg/mL fatty acid-free BSA, 0.49 mM, MgCl_2_, 1.17 mM CaCl_2_, 5.3 mM sodium lactate, Essential amino acids (EAA, 50×), Essential amino acids, (NEAA, 100×)). Zygotes were then transferred to SOF-aa media (0.3147 g NaCl, 0.02667g KCl, 0.0081 g KH_2_PO_4_, 0.0023 g MgCl_2_, 0.0235 g Sodium DL-Lactate solution (60%), 0.0216 g D-(+)-Glucose, 0.13035 g NaHCO_3_, CaCl_2_·2H_2_O, 0.0018 g Sodium pyruvate, 1 mL EAA (50×), 0.5 mL NEAA (100×), 0.2g BSA, 0.0075 g Glutamin/50 mL) under mineral oil, and incubated at 38.5 °C in 5% CO_2_.

### 2.4 Immunofluorescence staining of oocytes

Oocytes were washed three times in PBS containing 1% PVP, fixed in 4% para-formaldehyde for 1 h, and permeabilized with 0.2% Triton X-100 in PBS-PVP solution for 30 min. After washing three times, the oocytes were blocked in PBS-PVP containing 3%BSA for 1.5 h at 38.5°C. The oocytes were incubated with anti-Caspase-3 antibody (1:50, Affinity, A11953, United States), anti-Phospho-PDHA1/2(Ser293/Ser291) Antibody (1:100, Affinity, AF8502, United States), PDHA1 Ab (1:100, Affinity, AF6680, United States), Anti-Acetyllysine Rabbit mAb (1:50, PTM BioLab, PTM-105RM, China), Anti-α-Tubulin-FITC antibody, OXCT1 Polyclonal antibody (1:300, proteintech, 12175-1-AP, China). Mouse monoclonal (1:150, Sigma, F2168, United States). Alexa Fluor™ 488 goat anti-rabbit lgG (H + L) (1:400, Invitrogen, 2256692, United States). After cell nucle staining with Hoechst33342, the oocytes were mounted on glass slides with a drop of antifade mounting medium (Beyotime, P0126, China) and analyzed using a Nikon Eclipse Ti-S microscope equipped with a 198 Nikon DS-Ri1 digital camera (Nikon, Tokyo, Japan).

### 2.5 Western blotting

After 22 h of culture, 100 COCs were collected, then the cumulus cells were removed in 0.1% hyaluronidase (Sigma-Aldrich, United States). Oocytes were treated with pronase (10 mg/mL) to completely remove the zona pellucida and washed with SOF-Hepes on a warming plate. The denatured lysate was prepared with protease inhibitor and phosphatase inhibitor in a ratio of 44:1:5. 24 μM mixture was added to each group of cells for cracking. After complete cell cracking, the cells were centrifuged at 12,000 g for 5 min. Mix well, and finally take a metal bath for 10 min to denature the protein, and store in a −80°C refrigerator. Protein samples from 100 bovine oocytes were resolved by 12% SDS-PAGE and electro transferred onto transfer membranes that were blocked with 5% BSA (V900933, VETEC, China) for 1 h at Room Temperature (RT). The blots were incubated overnight at 4°C with antibodies of PDHA (1:500, Affinity, AF6680, United States), β-actin Rabbit mAb (1:1000, Cell Signaling Technology, 4970), and anti-Phospho-PDHA1/2(Ser293/Ser291) (Affinity, AF8502, United States). After washing four times with TBS-T (0.1% Tween-20 in Tris-buffered saline, TBS) the blots were incubated with horseradish peroxidase-labeled goat anti-rabbit IgG antibodies, then membranes were washed again four times with TBS-T. Immunoblots were visualized using an enhancer chemiluminescence detection kit (ECL Prime; GE Healthcare) and imaged using an Amersham Imager 600 All Western blot images are representative of 3 different experiments or biological repeats.

### 2.6 Intracellular ROS and GSH levels

To determine intracellular ROS levels, oocytes were incubated for 20 min in a PBS-PVP medium containing 10 µM 2′,7′-dichlorodihydrofluorescein diacetate. Dihydroethidium (DHE) and 2′,7′-dichlorodihydrofluorescein diacetate were dyed to observeROS level. ROS fluorescent probe-dihydroethidium (DHE) was a classic method to test ROS level in tissues or cells. DHE could enter in cells through living cell membrane freely, then it was oxidized by ROS to form oxidized ethidium which could mix with chromosomal DNA to produce red fluorescence. To determine intracellular DHE levels, 15 oocytes per group were incubated for 25 min in a PBS-PVP medium containing 10 μM Dihydroethidium (S0063; Beyotime, China). To determine intracellular GSH levels, 15 oocytes per group were was incubated for 30 min in a PBS-PVP medium containing 10 μM 4-chloromethyl-6,8-difluoro-7-hydroxycoumarin (CMF2HC) (Invitrogen). Fluorescent signals were captured as a tagged image file format (TIFF) using a digital camera (DP72; Olympus, Tokyo, Japan) connected to the fluorescence microscope (IX70, Olympus, Tokyo, Japan). The same procedures, including incubation, rinsing, mounting, and imaging, were followed for all groups of 15 oocytes per group. The exposure times were kept constant for all the measurement. Fluorescence images of oocytes were captured, ImageJ software was used to analyze the fluorescent intensities of the oocytes.

### 2.7 ΔΨm assay and Annexin V-FITC assay

The JC-1 probe was used to detect the mitochondrial membrane potential according to previously published methods (Elefantova et al., 2018). Briefly, after washing with SOF-HEPES medium for 3 times, the oocytes were placed in a culture medium, containing 0.5 μmol/L JC-1 (C2003S, Beyotime, China) in a 37°C, 5% CO_2_ incubator for 30 min. JC-1 forms J-aggregates and produces red fluorescence under normal mitochondrial membrane potential. When there is cell apoptosis, the mitochondrial membrane potential would be decreased or even lost, and JC-1 exists in J-monomers and produces green fluorescence. The ratio of red and green fluorescence intensities of oocytes reflected the mitochondrial membrane potential. Totally 15 oocytes were analyzed from each group. Data were presented as mean percentage (mean ± SEM) of at least three independent experiments. Active mitochondrial content performed using the mitochondrial mem-brane potential and apoptosis detection kit purchased from Beyotime Biotechnology (C1071, Beyotime, China). Briefly, oocytes were incubated in 188 μM Annexin V-FITC liquid containing 2 μM Mito-Tracker Red CMXRos, 5 μM Annexin V-FITC, 5 μM Hoechst 33342 for 30 min. Annexin V is one of the sensitive indicators for the detection of apoptosis. It is a phospholipid-binding protein that can bind to the membrane of early apoptotic cells, and the change of plasma membrane is one of the earliest changes in cell apoptosis. During apoptosis, the membrane phosphatidylserine (PS) turns from the inside of the plasma membrane to the outside. A fluorescent ring was formed in the outermost part of apoptotic oocytes. Fluorescence was visualized using the digital camera connected to the fluorescence microscope. The images were processed using the ImageJ software. The fluorescence intensity per pixel was automatically computed by ImageJ.

### 2.8 ATP content

Staining methods refer to a published study (Jin et al., 2017). Briefly, denuded oocytes were washed three times in 1% PBS-PVP and fixed with 4% paraformaldehyde-PBS-PVP for 1 h, washed three times, and incubated in 1%PBS-PVP supplemented with 500 nM BODIPY FL ATP for 1 h at room temperature in the dark. Oocytes were washed three times in 5%PBS-PVP and mounted on coverslips. Images of each oocyte were captured using an epifluorescence microscope (TE 2000- S; Nikon).

### 2.9 Reverse transcription quantitative polymerase chain reaction (RT-qPCR) analysis

Total RNA was extracted from a mixture of 50 oocytes, using the PicoPure™ RNA Separation kit (KIT0204, Applied Biosystems™, Sigma), according to the manufacturer’s instructions. Data were presented as mean percentage (mean ± SEM) of at least three independent experiments. First-strand cDNA was synthesized by the One-Step gDNA Removal and cDNA Synthesis SuperMix (TransGen). RT-qPCR was performed using SYBR Green, a fluorophore that binds double-stranded DNA, in a final reaction volume of 20 µL using the CFX96 touch RT-PCR detection system (Bio-Rad, Hercules, CA, United States). Gene expression was quantified by the 2^−ΔΔCT^ method, with normalization to the expression levels of 18SrRNA. The PCR primers used to amplify each gene are listed in Table 1.

**TABLE 1:** The primers used in RT-qPCR analysis.

Gene name	Gene sequence (5′-3′)	Genebank No.
*BMP-15*	F: ATC​ATG​CCA​TCA​TCC​AGA​ACC​TTG​TC	NM_001031752.1
	R: AGA​TAC​TCC​CAT​TTG​CCT​CAA​TCA​GAA​G	
*GDF-9*	F: TTC​CTA​TTA​GCC​TTG​ATT​CTC​AGC​CTT​C	NM_174681.2
	R: CCA​AGT​CTC​AGC​CTC​AGA​TTC​CAA​C	
*SOD1*	F: AGA​GGC​ATG​TTG​GAG​ACC​TG	NM_174615.2
	R: CAG​CGT​TGC​CAG​TCT​TTG​TA	
*CAT*	F: TGG​GAC​CCA​ACT​ATC​TCC​AG	NM_001035386.2
	R: AAG​TGG​GTC​CTG​TGT​TCC​AG	
*18SrRNA*	F: GAC​TCA​TTG​GCC​CTG​TAA​TTG​GAA​TGA​GTC	XM_024994376.1
	R: GCT​GCT​GGC​ACC​AGA​CTT​G	

### 2.10 *In vitro* acetylation assay

We added acetyl-CoA to rescue the reduction of acetylation levels. Acetyl-CoA is a cofactor required for acetylation, and adding it with 0.1 mM acetyl-CoA to the cell culture medium could potentially reverse the effects of βHB on acetylation levels.

### 2.11 Statistical analysis

All data were analyzed with GraphPad by one-way ANOVA, and immunofluorescence staining images were analyzed with ImageJ. The results were considered significantly different at *p* < 0.05, and extremely significant at *p* < 0.01.

## 3 Result

### 3.1 βHB exposure affects bovine oocyte maturation and developmental competence

**FIGURE 1:**
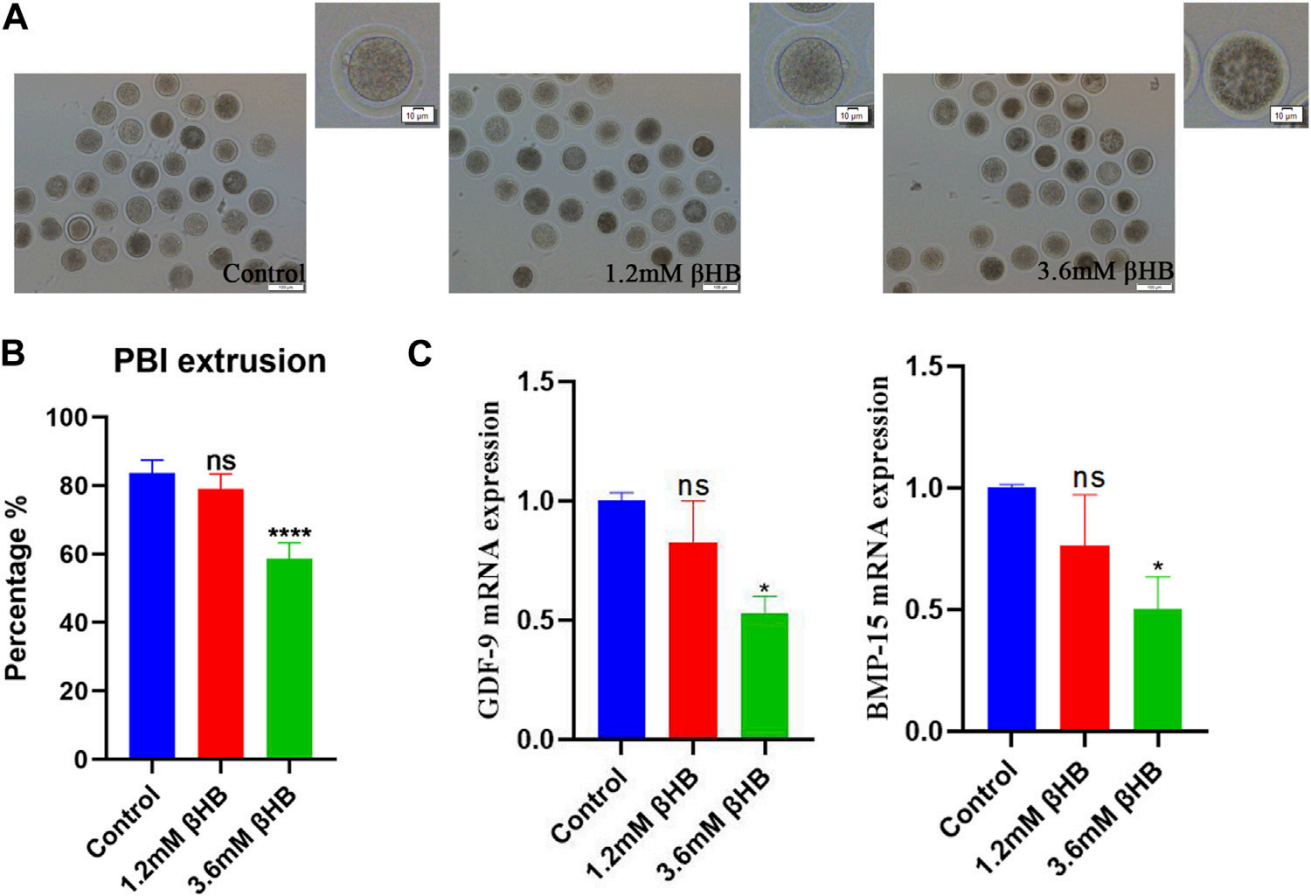
βHB exposure affects bovine oocyte maturation. **(A)** Representative morphology of oocytes maturation after 1.2 mM and 3.6 mM βHB exposure. Bar = 100 μm. The upper right corner is the enlarged image. **(B)** Rate of polar body extrusion in control and different concentrations of the βHB-exposed groups (1.2 and 3.6 mM). **(C)** Expression of genes was detected in control, 1.2 and 3.6 mM βHB groups. Significant difference (****, *p* < 0.000; ***, *p* < 0.001; **, *p* < 0.01; *, *p* < 0.05).

To evaluate the impact of βHB on bovine oocyte maturation, we exposed the oocytes to clinical (3.6 mM) and subclinical (1.2 mM) concentrations of βHB and assessed the maturation rate. The results showed that the 3.6 mM βHB group had a significantly lower maturation rate (*n* = 413, 58.66% ± 4.627%), compared to the control group (*n* = 406, 83.7% ± 3.772%, *p* < 0.0001), while the 1.2 mM βHB group exhibited a similar maturation rate of approximately 78%–80% (*n* = 396, 79% ± 4.396%, *p* > 0.05). Growth differentiation factor 9 (GDF-9) and bone morphogenetic protein 15 (BMP-15), which play a crucial role in regulating the growth, differentiation, and function of granulosa and thecal cells during follicular development, were measured (Figure 1C) to gather more insights into the effects of βHB on bovine oocyte maturation. As compared to the control group, the mRNA expression levels of BMP-15 and GDF-9 were significantly reduced in the 3.6 mM βHB group (*p* < 0.05), whereas the 1.2 mM βHB group exhibited similar mRNA expression levels. Notably, both the cleavage rate and blastocyst rate decreased significantly in the 3.6 mM βHB and 1.2 mM βHB groups when compared to the control group (Table 2). Our findings indicate that βHB has a prominent inhibitory effect on the maturation and developmental potential of bovine oocytes.

**TABLE 2:** Fertilization embryo cleavage rates and blastocyst rates treated with βHB.

Group	No. of embryos examined	No. of cleaved (%)	No. of embryos developed to the 8-cell stage (%)	No. of embryos developed to the blastocyst stage (%)
Control	204	169 (82.9 ± 5.17)^a^	131 (64.35 ± 1.55)^a^	87 (42.63 ± 5.12)^a^
1.2 mM βHB	212	144 (67.99 ± 8.99)^b^	113 (53.13 ± 3.01)^b^	79 (37.30 ± 7.67)^a^
3.6 mM βHB	199	115 (57.68 ± 6.64)^c^	76 (38.02 ± 3.02)^c^	52 (25.95 ± 3.93)^b^

Values with diferent superscripts (a,b,c) within the same column are signifcantly diferent, *p* < 0.05.

### 3.2 βHB reduces mitochondrial function, and induces oxidative stress and apoptosis in bovine oocytes

**FIGURE 2:**
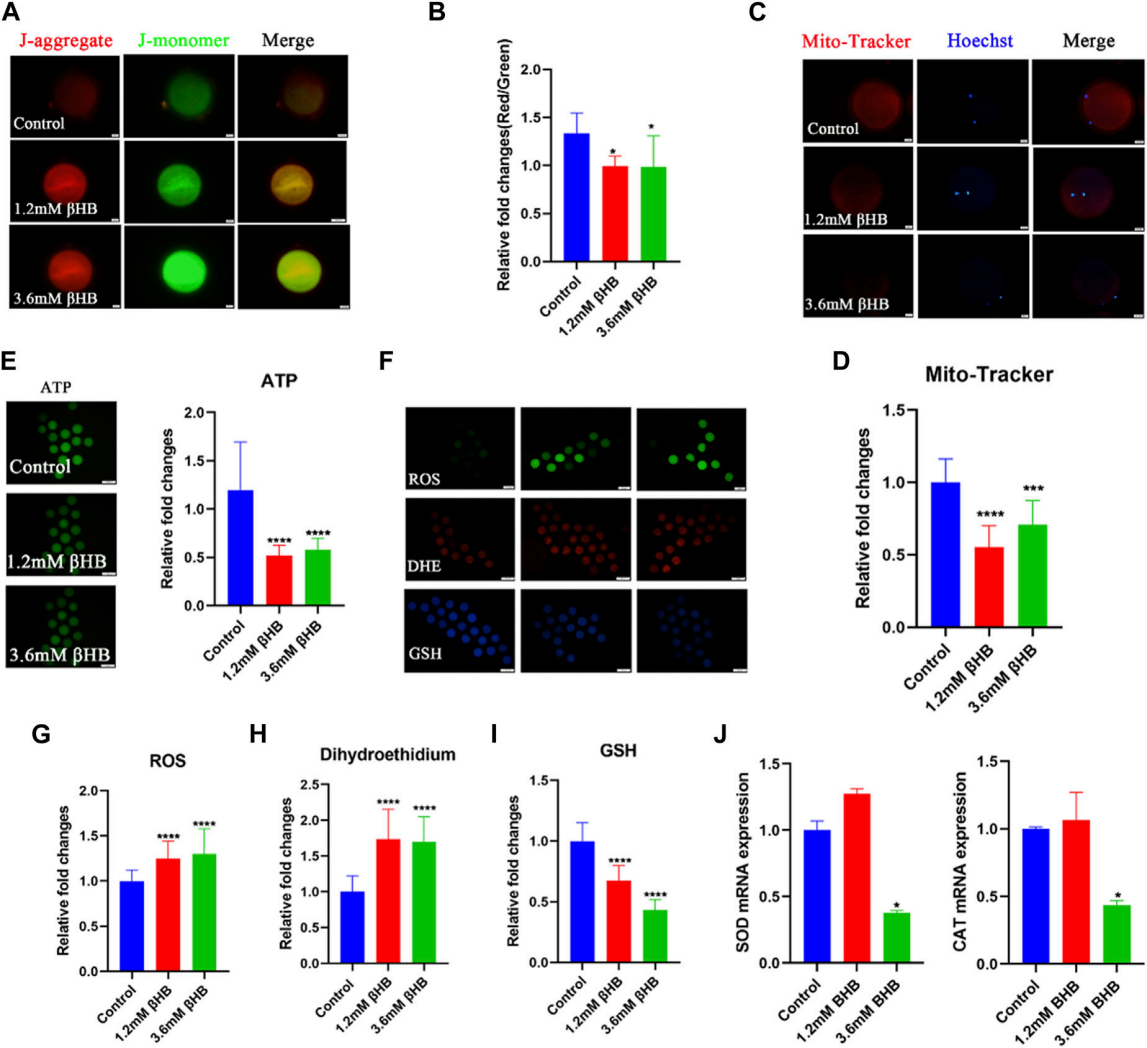
βHB exposure impairs mitochondria functions induced oxidative stress in bovine oocytes. **(A)** Mitochondrial membrane potential (ΔΨm) was detected by JC-1 staining in control, 1.2 mM and 3.6 mM βHB-exposed oocytes. Merged images represented JC-1 aggregate and JC-1 monomer. Scale bar = 20 μm. **(B)** The ratio of red and green fluorescence intensity was calculated in the control and βHB exposure group. **(C)** Representative images of mitochondria in control, 1.2 mM and 3.6 mM βHB-exposed oocytes. Mito-Tracker stained active mitochondria and hoechst stained nuclei is represented in merge. Bar = 20 μm. **(D)** Mean fluorescent intensity of Mito-Tracker in control, 1.2 mM and 3.6 mM βHB exposure. **(E)** Representative images of ATP, the relative fluorescence intensity of ATP was analyzed in control, 1.2 mM and 3.6 mM βHB-exposed oocyte. **(F)**. Representative images of GSH, DHE, ROS signals in control, 1.2 mM, and 3.6 mM βHB-exposed oocytes. Bar = 100 μm. The relative fluorescence intensity of **(G)** ROS, **(H)** DHE, **(I)** GSH were analyzed in control, 1.2 mM and 3.6 mM βHB-exposed oocyte. **(J)** Expression of oxidative stress related-genes was detected in control, 1.2 and 3.6 mM βHB groups. Significant difference (****, *p* < 0.000; ***, *p* < 0.001; **, *p* < 0.01; *, *p* < 0.05).

The mitochondria play a key role in the aerobic respiration of eukaryotes, providing much-needed energy in the form of ATP. To investigate their functions, we evaluated the mitochondrial membrane potential (ΔΨm) using JC-1 staining. Mitochondria with high membrane potential emitted a red fluorescence, whereas those with low membrane potential emitted a green fluorescence (Figure 2A). The quantitative analysis indicated that the ratio of red to green fluorescence was significantly reduced in βHB-treated oocytes compared to the control group (Figure 2B) (*p* < 0.05). We evaluated the mitochondrial content through Mito-Tracker staining and our results, as depicted in Figures 2C, D, showed a significant reduction in mitochondrial content in both the 1.2mM and 3.6 mM βHB-treated groups compared to the control group (*p* < 0.001). Furthermore, as showed in Figure 2E, the ATP content of oocytes treated with βHB was lower than that of the control group (*p* < 0.01). Hence, we infer that the decline in oocyte quality could be attributed to primary causes such as mitochondrial dysfunction and reduced ATP levels.

Mitochondrial dysfunction is closely associated with an abnormal oxidative phosphorylation process, leading to the excess generation of ROS, which in turn induces oxidative stress. To investigate this, we detected GSH and ROS. As shown in Figures 2F–I, GSH levels were significantly lower in the βHB-treated oocytes, especially in those treated with 3.6 mM βHB, while βHB-treated oocytes exhibited significantly increased fluorescence signals for ROS and DHE. Furthermore, the expression levels of superoxide dismutase (SOD1) and catalase (CAT) were markedly disrupted in the βHB-treated oocytes (*p* < 0.05) (Figure 2J), compared to the control group. Thus, we infer that the mitochondrial dysfunction induced oxidative stress.

**FIGURE 3:**
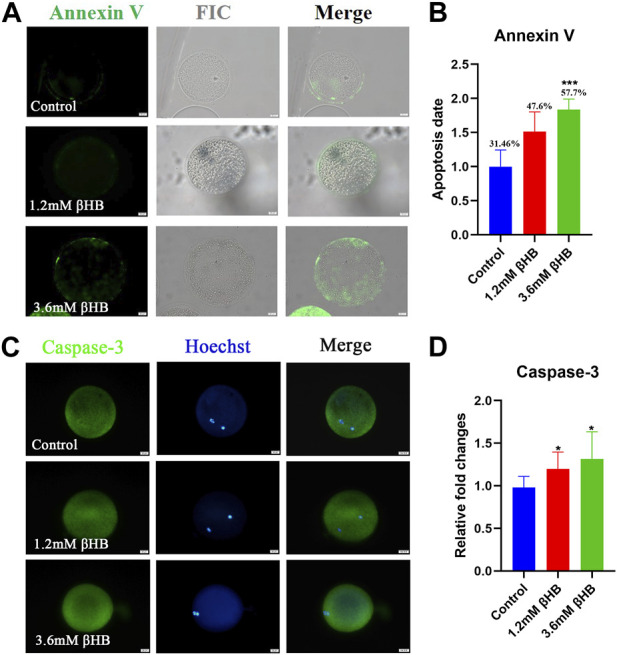
βHB exposure triggered early apoptosis in bovine oocytes. **(A)** Representative image of apoptotic status, assessed by Annexin-V staining in control, βHB oocytes. Green, Annexin-V-FITC. Annexin Ⅴ bind to cell membranes in merge. Bar = 20 µm. **(B)** The fluorescence intensity of An-nexin-V signals was quantified in the control and βHB groups. **(C)** Representative images of Caspase-3. Green, Caspase-3; blue, Hoechst. Caspase-3 protein and hoechst stained nuclei is represented in merge. Bar = 20 µm. **(D)** The relative fluorescence intensity of Caspase-3 of oocytes with or without βHB exposure. Significant difference (****, *p* < 0.000; ***, *p* < 0.001; **, *p* < 0.01; *, *p* < 0.05).

Oxidative stress is commonly regarded as a crucial indication of apoptosis. As expected, βHB exposure increased the incidence of apoptosis, as indicated by Annexin-V positive signals (Figure 3A). Compared to the control group (31.46% ± 7.725%, n = 97), the percentage of oocytes positive for apoptosis significantly increased in the 3.6 mM βHB group (57.76% ± 4.870%, *n* = 93, *p* < 0.001) (Figure 3B). To confirm this further, we evaluated the protein levels of caspase-3. The protein expression of caspase-3 (Figures 3C, D) was evidently upregulated in both the 1.2mM and 3.6 mM βHB exposed groups (*n* = 15, *p* < 0.05). In conclusion, the results indicate that exposure to βHB impairs mitochondrial function, induces oxidative stress, and triggers apoptosis in bovine oocytes.

### 3.3 βHB affects bovine oocyte maturation and embryo development potential by regulating PDH activity

**FIGURE 4:**
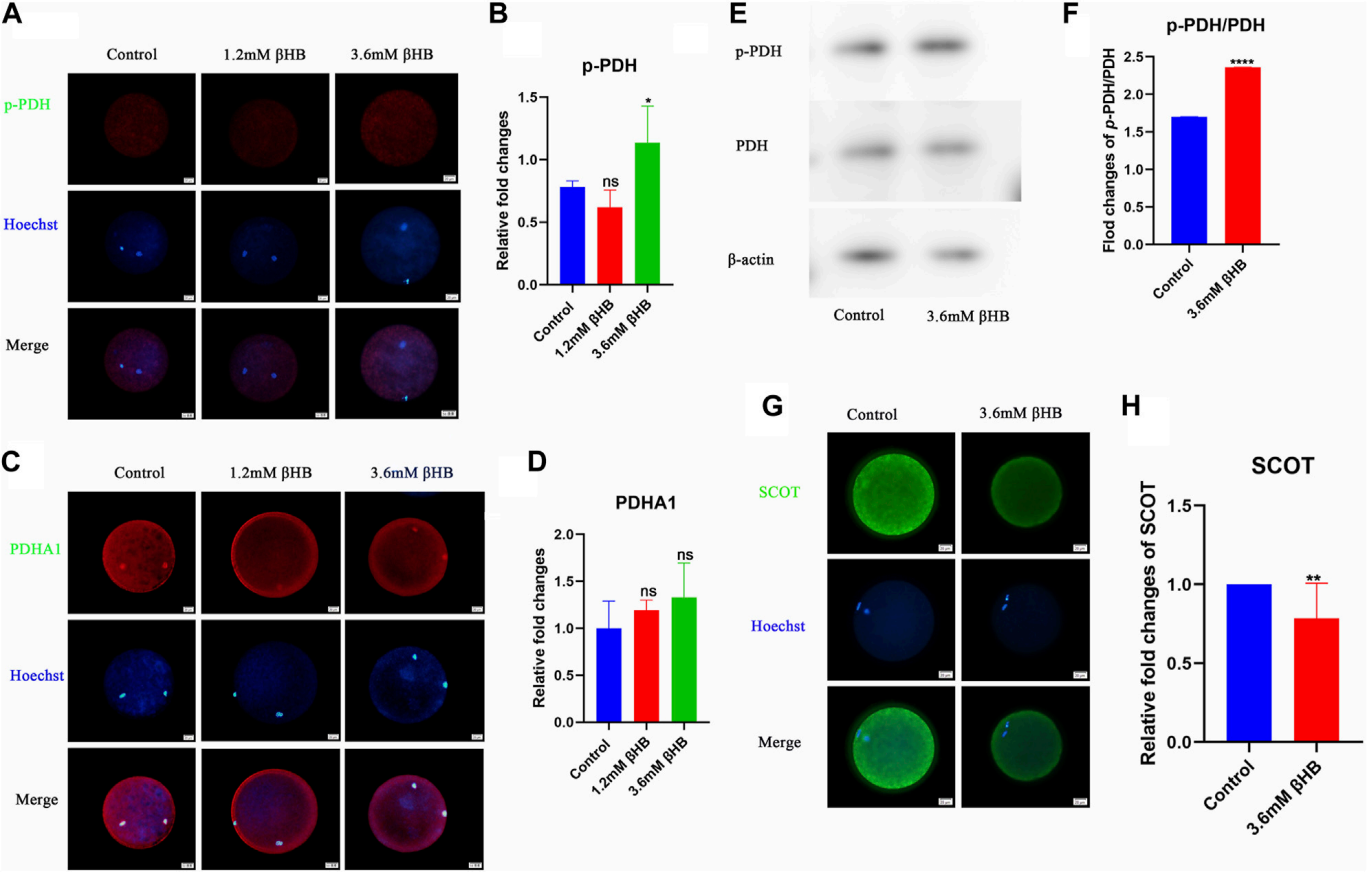
βHB exposure can reduce PDH activity on bovine oocyte maturation. **(A)** Representative images of Phospho-PDH (p-PDH). Green, p-PDH; blue, Hoechst. p-PDH protein and hoechst stained nuclei is represented in merge. Bar = 20 µm. **(B)** The relative fluorescence intensity of p-PDH of oocytes with or without βHB exposure. **(C)** Representative images of PDHA1. Green, PDHA1; blue, Hoechst. PDHA1 protein and hoechst stained nuclei is represented in merge. Bar = 20 µm. **(D)** The relative fluorescence intensity of PDHA1 of oocytes with or without βHB exposure. **(E)** p-PDH and PDH expression in bovine oocytes treated with βHB (0mM; 3.6 mM. Beta-actin was employed as protein loading control. **(F)** Representative Western blot of p-PDH/PDH in bovine oocytes stimulated with 3.6 mM βHB. **(G)** Representative images of SCOT. Green, SCOT; blue, Hoechst. SCOT protein and hoechst stained nuclei is represented in merge. Bar = 20 µm. **(H)** The relative fluorescence intensity of SCOT of oocytes with or without βHB exposure. Significant difference (****, *p* < 0.000; ***, *p* < 0.001; **, *p* < 0.01; *, *p* < 0.05).

Pyruvate serves as a direct energy substrate for the oxidative phosphorylation pathway in oocytes, so we examined the activity of pyruvate dehydrogenase (PDH), which is the first enzyme that limits the rate of the pyruvate metabolic pathway. As shown in Figures 4A, B, exposure to 3.6 mM βHB significantly increased the level of phosphorylated PDH (p-PDH) and significantly inhibited its activity (*p* < 0.01), while the level of PDHA1 remained unchanged (Figures 4C, D). Western blot analysis revealed that compared to the control group, the level of p-PDH/PDH was significantly higher in the βHB group (Figures 4E, F), which is consistent with previous findings (Liu et al., 2019; Cenigaonandia-Campillo et al., 2021; Yang et al., 2021). These results suggest that βHB-induced mitochondrial dysfunction may occur by inhibiting PDH activity. Furthermore, the significant decrease of the key enzyme SCOT in ketone body metabolism observed in the βHB group (Figures 4G, H) suggests an abnormal utilization of ketone bodies.

**FIGURE 5:**
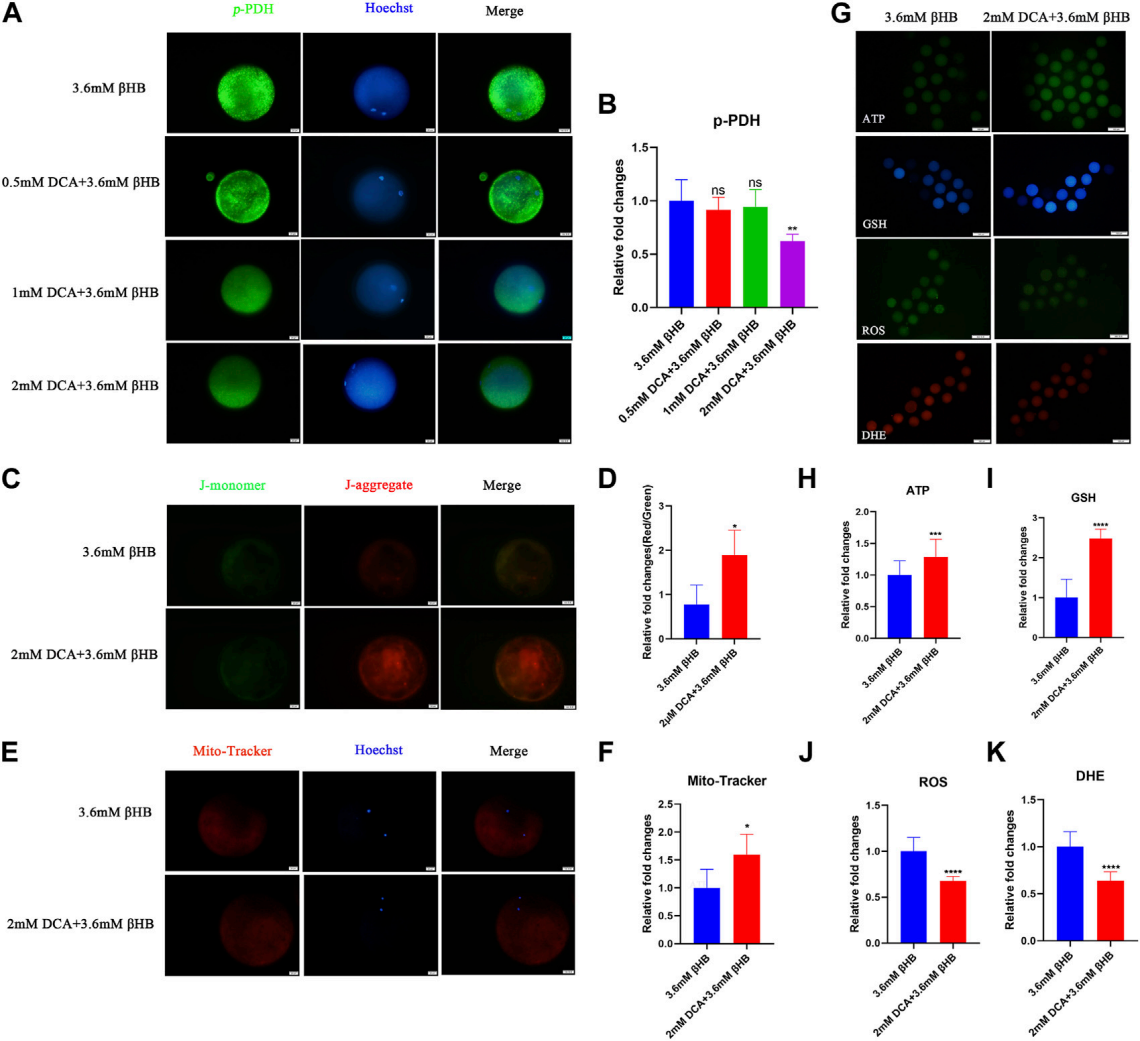
βHB affects bovine oocyte maturation and embryo development potential by regulating PDH activity. **(A)** Representative images of Phospho-PDH. Green, p-PDH; blue, Hoechst. p-PDH protein and hoechst stained nuclei is represented in merge. Bar = 20 µm. **(B)** The relative fluorescence intensity of p-PDH of oocytes with different concentrations of DCA on βHB-exposure oocytes. **(C)** Mitochondrial membrane potential (ΔΨm) was detected by JC-1 staining in βHB and DCA+βHB group. Merged images represented JC-1 aggregate and JC-1 monomer. Bar = 20 μm. **(D)** The ratio of red and green fluorescence intensity was calculated in the βHB and DCA+βHB group. **(E)** Representative images of mitochondria in βHB and DCA+βHB group. Mito-Tracker stained active mitochondria and hoechst stained nuclei is represented in merge. Bar = 20 μm. **(F)** Mean fluorescent intensity of Mito-Tracker in βHB and DCA+βHB group. **(G)** Representative images of ATP, GSH, DHE, and ROS signals in βHB and DCA+βHB group oocytes. Bar = 100 μm. The relative fluorescence intensity of **(H)** ATP, **(I)** GSH, **(J)** ROS, **(K)** DHE were analyzed in control, 1.2 mM and 3.6 mM βHB-exposed oocytes. Significant difference (****, *p* < 0.000; ***, *p* < 0.001; **, *p* < 0.01; *, *p* < 0.05).

In order to further investigate the crucial role of PDH, oocytes were treated with βHB and/or DCA (an activator of PDH). The findings indicated that PDH activity could be stimulated by 2 mM DCA supplementation (Figures 5A, B). The cleavage rate and blastocyst rate increased significantly in the 2 mM DCA+3.6 mM âHB when compared to the 3.6 mM âHB group (Table 3). And additionally, as depicted in Figures 5C–K, compared to the 3.6 mM βHB group, the supplementation of 2 mM DCA significantly enhanced the mitochondrial membrane potential (ΔΨm) (*p* < 0.05), mitochondrial content (*p* < 0.05), the levels of GSH and ATP (*p* < 0.01), reduced ROS (*p* < 0.001) and DHE (*p* < 0.001) levels. Thus, elevating PDH activity through DCA can alleviate the βHB-induced mitochondrial dysfunction and oxidative stress.

**TABLE 3:** Fertilization embryo cleavage rates and blastocyst rates treated with 2 mM DCA + 3.6 mM βHB.

Group	No. of embryos examined	No. of cleaved (%)	No. of embryos developed to the 8-cell stage (%)	No. of embryos developed to the blastocyst stage (%)
3.6 mM βHB	240	135 (56.13 ± 6.41)^a^	77 (31.67 ± 2.08)^a^	65 (27.23 ± 00.86)^a^
2 mM DCA+3.6 mM βHB	247	199 (80.54 ± 2.1)^b^	158 (63.81 ± 3.17)^b^	103 (41.60 ± 2.95)^b^

Values with diferent superscripts (a,b) within the same column are signifcantly diferent, *p* < 0.05.

### 3.4 βHB exposure impedes global acetylation levels and spindle assembly

**FIGURE 6:**
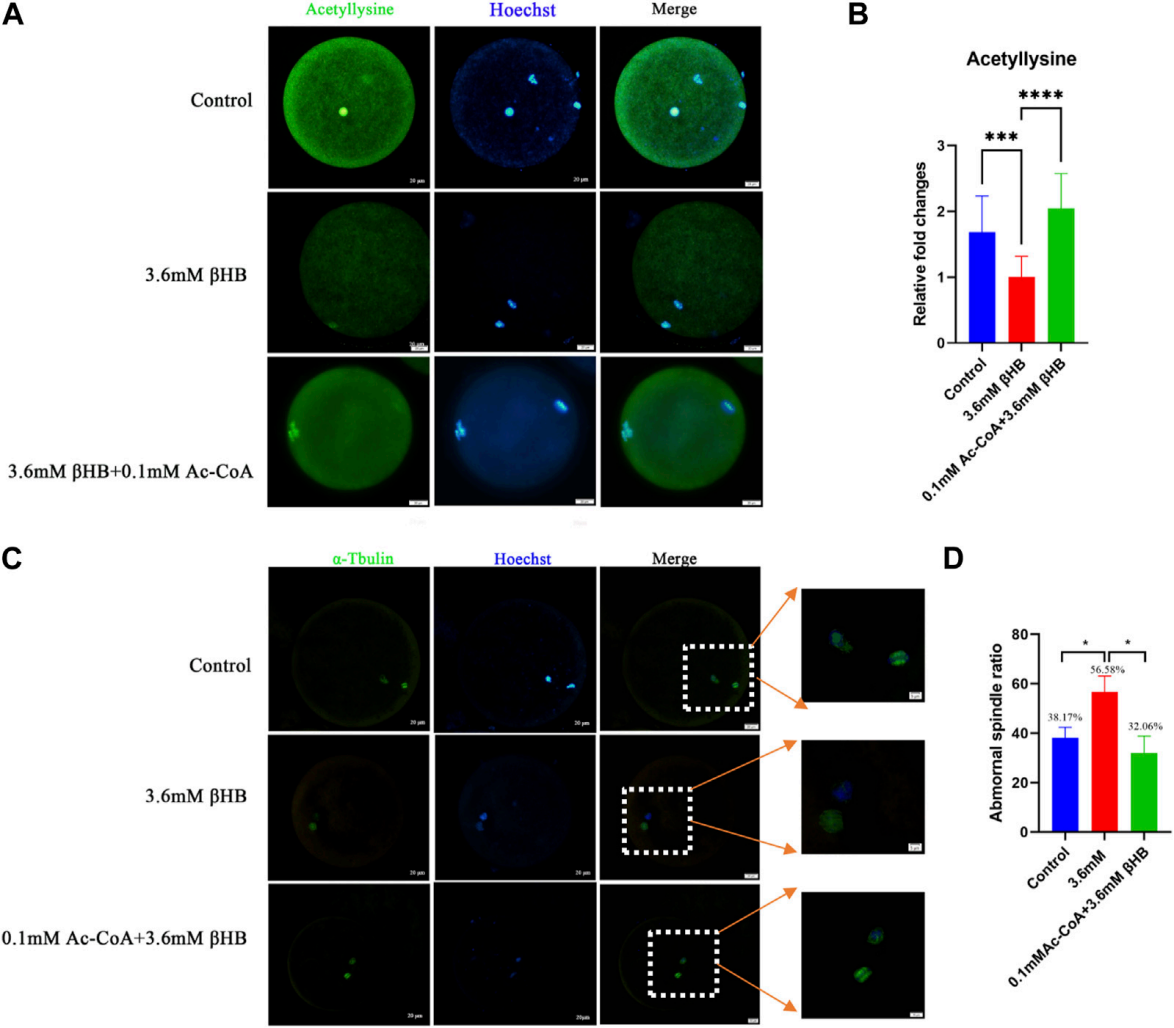
βHB exposure impedes the acetylation levels and spindle assembly, which can be rescued by Ac-CoA. **(A)** Representative images of Acetyllysine. Green, acetyllysine; blue, Hoechst. Acetyllysine protein and hoechst stained nuclei is represented in merge. Bar = 20 µm. **(B)** The relative fluorescence intensity of Acetyllysine of oocytes in control, 3.6 mM βHB group and 0.1 mM Ac-CoA+3.6 mM βHB. **(C)** Control, 3.6 mM βHB group, and 0.1 mM Ac-CoA+3.6 mM βHB were stained with α-tubulin antibody to visualize spindle (green) and counterstained with Hoechst to cell nuclear (blue). Control oocytes show a representative bar-rel-shaped spindle. Elongated and disorganized spindles (arrows) in βHB oocytes. Spindle and DNA is represented in merge. Bar = 20 µm. (Enlarge image, Scale bar: 5 μm). **(D)** Quantification of control (106), 3.6 mM βHB group (98) and 0.1 mM Ac-CoA+3.6 mM βHB (127) with spindle defects. Significant difference (****, *p* < 0.000; ***, *p* < 0.001; **, *p* < 0.01; *, *p* < 0.05).

PDH has a vital role in catalyzing the conversion of pyruvates to acetyl-CoA, which provides acetyl groups for protein modifications. Based on this, we conducted a study to investigate the levels of acetylation modification in bovine oocytes after βHB treatment. The findings demonstrated that the intracellular acetylation modification was significantly decreased following the 3.6 mM βHB treatment (Figures 6A–B) (*p* < 0.001). Furthermore, the rate of spindle morphology abnormalities was significantly elevated after βHB treatment (Figures 6C–D).

In order to validate our findings, we explored the possibility of restoring the acetylation levels reduction caused by βHB in bovine oocytes by adding acetyl-CoA. Oocytes were subjected to treatment with βHB and/or 0.1 mM acetyl-CoA. The results showed that compared to the βHB group, there was a significant restoration of intracellular acetylation modification in the Ac-CoA group (Figures 6A–B) (*p* < 0.0001), and a decrease in the rate of spindle morphological abnormalities (Control, *n* = 106, 38.17 ± 4.26; 3.6 mM βHB group, *n* = 98, 56.68 ± 6.40; 0.1 mM Ac-CoA+3.6 mM βHB, *n* = 127,32.06% ± 6.89%,Figures 6C–D). Therefore, we infer that the inhibition of PDH activity induced by βHB may lead to distorted Acetyl-CoA production, negatively affecting the intracellular acetylation levels and spindle morphology in bovine oocytes.

## 4 Discussion

Ketosis is common in high-yield dairy cows. It is caused by the accumulation of serum ketone βHB. Both subclinical ketosis and clinical ketosis can compromise reproductive performance and cause long-lasting negative effects on reproductive efficiency (Gong et al., 2022). In this study, we detected the effects of βHB on mitochondria functions and energy metabolism during bovine oocyte maturation. Our results indicated that βHB decreased bovine oocyte quality via mitochondria dysfunction-induced oxidative stress and PDH Activity Downregulation.

Oocyte maturation quality is vital for successful fertilization and embryo development during bovine reproduction (Dai et al., 2021). The polar body extrusion and embryo development potential are regarded as two critical indicators for oocyte maturation. Therefore, we tested the effects of βHB on these two processes. Our study found that the rate of the PBI extrusion was decreased obviously when oocytes were treated with 1.2 and 3.6 mM βHB, indicating a decline in oocyte maturation. A study about the effects of liver abnormality on bovine oocytes also confirmed the negative correlation between the rates of blastocyst formation and the concentrations of βHB in follicular fluid (Sarentonglaga et al., 2013). βHB in follicular fluid caused a concentration-depended reduction in the frequency of fertilized oocytes (Sarentonglaga et al., 2013). Similar studies in other species also reported that the rate of ovine oocyte maturation began to decrease significantly on exposure to 1 μM βHB concentration (Nandi et al., 2017; Nandi et al., 2018), consistent with our results. Therefore, we further explored the detailed mechanisms of βHB on bovine oocytes maturation in this study.

Proper functions of mitochondria are critical for oocyte maturation. Many studies have shown that mitochondrial dysfunction is one of the primary causes of oocyte quality decline (Labarta et al., 2019; Boguenet et al., 2021; Kirillova et al., 2021). The results of the present study revealed that mitochondrial content, number, and membrane potential were disturbed in βHB-treated oocytes. Thus, mitochondria dysfunction might be the potential incentive leading to the quality decline of βHB-treated oocytes. Oocyte mitochondria are the major source ofROS production, and mitochondrial dysfunction might lead to the accumulation of ROS, which will induce oxidative stress (Yousefian et al., 2021). Our results indicated that the levels of ROS significantly increased, then as expected, the GSH levels also observably decreased, suggesting oxidative stress of βHB treated oocytes. Furthermore, oxidative stress triggered early apoptosis, as shown by the enhanced levels of Caspase-3 and Annexin-V. The previous study reported that the increased βHB levels (from 1.2 mM) induced bovine abomasum smooth muscle cells oxidative stress and apoptosis by excessive ROS and the promoted expression of Bax and Caspase-12, 9, 3 (Tian et al., 2014). βHB is also able to induce oxidative stress on adult cardiomyocytes (Pelletier and Coderre, 2007). However, βHB shows protective effects on HEK293 cells (Shimazu et al., 2013) and PC12 cells (Cheng et al., 2013). These contrasting results may be caused by the different does and cell types. Together, these results indicated that βHB caused mitochondria dysfunction, further inducing oxidative stress and apoptosis in bovine oocytes.

Impairment of PDH activity may cause maturation decline in βHB-treated oocytes. In our study, we observed that ATP levels and the key enzyme SCOT, which is involved in the utilization of ketone bodies, were inhibited in bovine oocytes treated with βHB. A high concentration of βHB may suppress energy production processes, leading to an energy-shortage condition that decreases oocyte competence. PDH plays a crucial role in oocyte ATP supply, metabolism (Leese and Barton, 1984; Gardner and Leese, 1990; Harris et al., 2005), and scavenging of ROS (O'Donnell-Tormey et al., 1987), as it catalyzes the rate-limiting step of the tricarboxylic acid (TCA) cycle. In mammals, PDH activity is jointly regulated by pyruvate dehydrogenase kinase (PDK) and pyruvate dehydrogenase phosphatase (PDP) through a reversible phosphorylation dephosphorylation cycle (Gao et al., 2022). We have verified that βHB significantly increases the p-PDH, thereby inhibiting its activity. Consistent with a previous study that suggested PDK3-mediated phosphorylation of Ser293-PDHE1α disrupts mouse meiotic spindle morphology and chromosome alignment and reduces total ATP levels by inhibiting PDH activity (Hou et al., 2015). Another study also showed that the competency of mouse oocytes is influenced by a diabetic environment (an energy-related disease) and is mediated through the PDK1-controlled PDHE1α phosphorylation pathway (Ge et al., 2021). Disruption of PDH activity may be a potential cause for the decline in maturation of oocytes treated with βHB. However, further research is necessary to determine whether PDH phosphorylation is involved in the meiotic assembly of bovine oocytes and which specific regulatory sites are affected.

DCA displayed an promoting effect on PDH activity by inhibiting the phosphorylation and inactivation of the pyruvate dehydrogenase complex in previous studies. Other studies also demonstrated that DCA supplementation showed beneficial influence on embryo development via regulating mitochondrial dysfunction or ATP production (Penzias et al., 1993; McPherson et al., 2014). Our results also showed that DCA can rescued the βHB induced harmful effects via promoting PDH activity during oocyte maturation. These results demonstrated that βHB caused oocytes maturation arrest due to the decreased PDH activity.

PDH complex plays a pivotal role in oocyte meiotic maturation via its functions in catalyzing pyruvates to convert to acetyl-CoA. The previous study reported that PDH is highly expressed and translocated to the porcine embryo nucleus to maintain histone acetylation levels by producing sufficient amounts of acetyl-CoA during ZGA (Zhou et al., 2020). Thus, Acetyl-CoA can provide acetyl groups, and regulate the process of protein acetylation, which promoted us to investigate the global acetylation changes after treatment with βHB. We showed that 3.6 mM βHB obviously reduced the intracellular acetylation level in bovine oocytes, suggesting that βHB may induce the reduction of acetylation, which is important for many key cellular processes (He et al., 2021). Similar to our study, the previous research reported that βHB reduced p53 acetylation and activation (Liu et al., 2019). Acetylation of histone (He et al., 2021) and non-histone (Reichmann et al., 2020) have essential roles in follicle development and oocyte maturation. Therefore, oocyte quality reduction and the embryo development arrest may be caused by repressed PDH activity downregulates global acetylation through Acetyl-CoA production.

## 5 Conclusion

Our current study aims to investigate the impact of βHB at both subclinical and clinical concentrations on bovine oocytes. As shown in our results, βHB was found to impede oocyte maturation and development capacity though mitochondria dysfunction and Pyruvate dehydrogenase (PDH) associated energy metabolism abnormality, such as intracellular acetylation modification. These effects may contribute to the decline in oocyte quality and potentially result in reproductive disorder in both future embryos and offspring. Our findings offer novel insights into the detrimental effects of ketosis on bovine reproductive efficiency.

## Data Availability

The original contributions presented in the study are included in the article/Supplementary Material, further inquiries can be directed to the corresponding authors.
