# Supplementary figures and images for: β-hydroxybutyrate impairs bovine oocyte maturation via pyruvate dehydrogenase (PDH) associated energy metabolism abnormality (part 1 of 2)
Source: Front Pharmacol. 2023 Aug 11;14:1243243. doi: 10.3389/fphar.2023.1243243 (PMC10450765; doi:10.3389/fphar.2023.1243243)

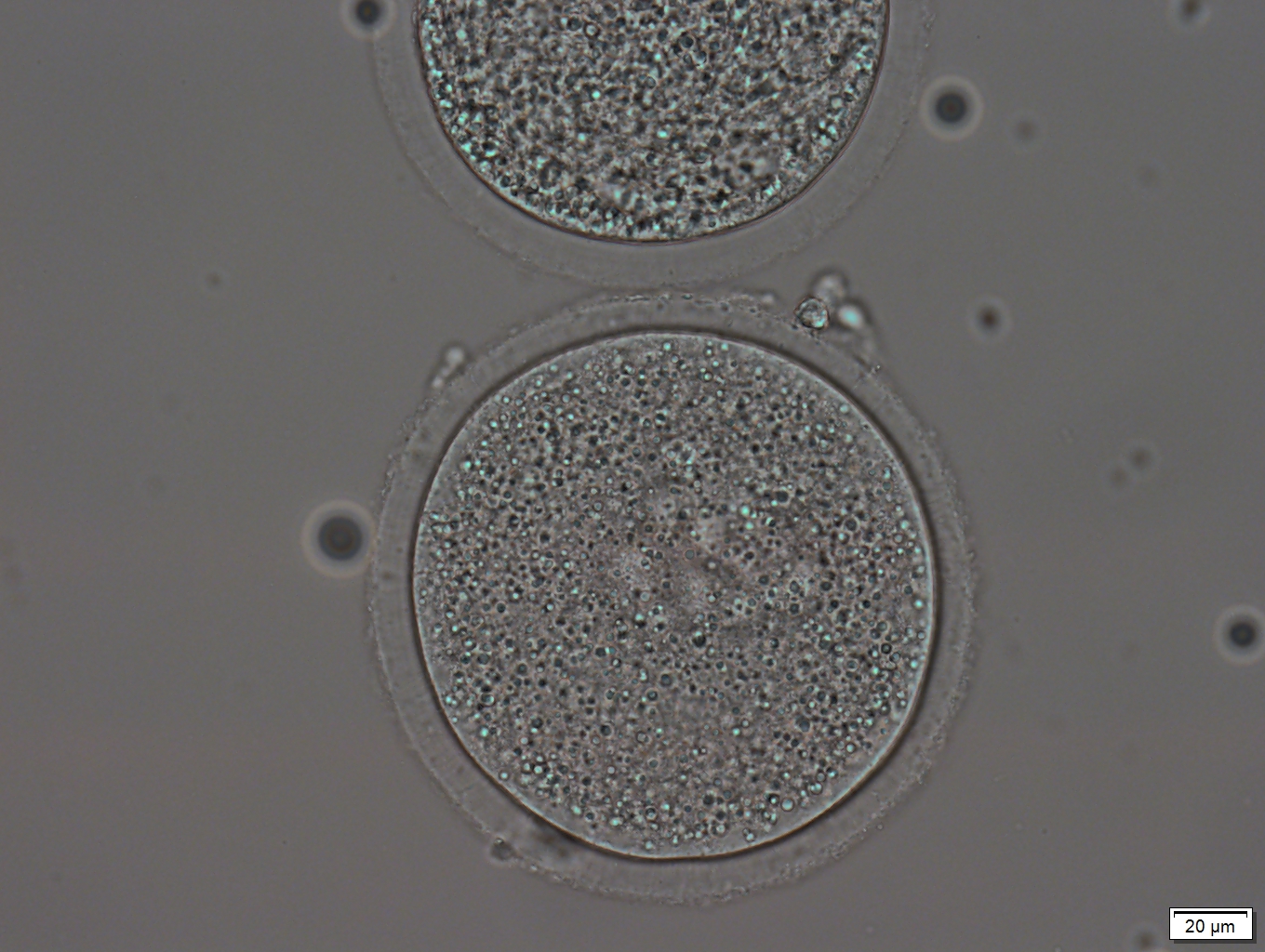

Supplement: Supplementary file 1 [file DataSheet3.ZIP › Figure3í╠/Annex ó⌡/1.2mM (2).jpg]

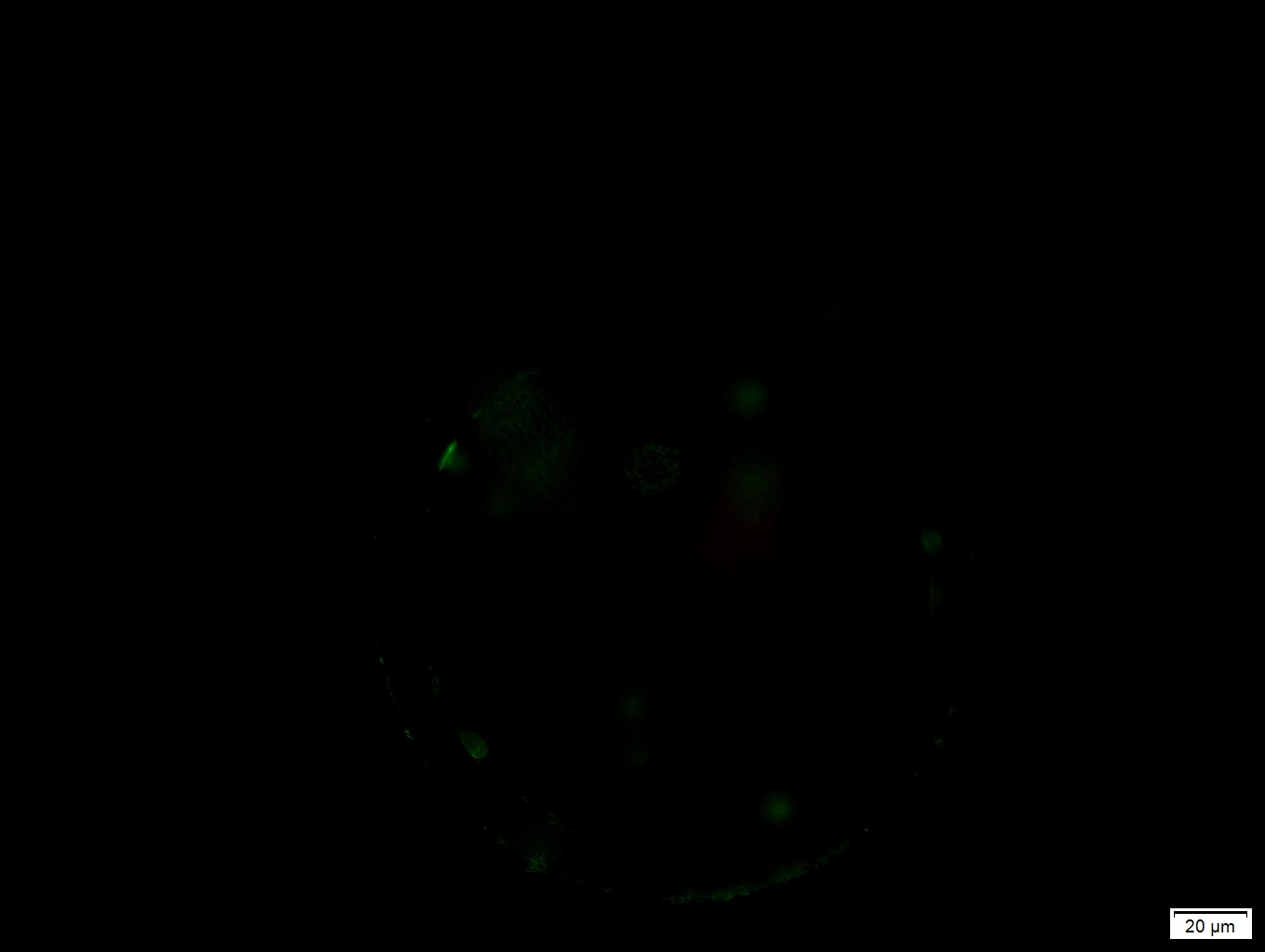

Supplement: Supplementary file 1 [file DataSheet3.ZIP › Figure3í╠/Annex ó⌡/1.2mM.jpg]

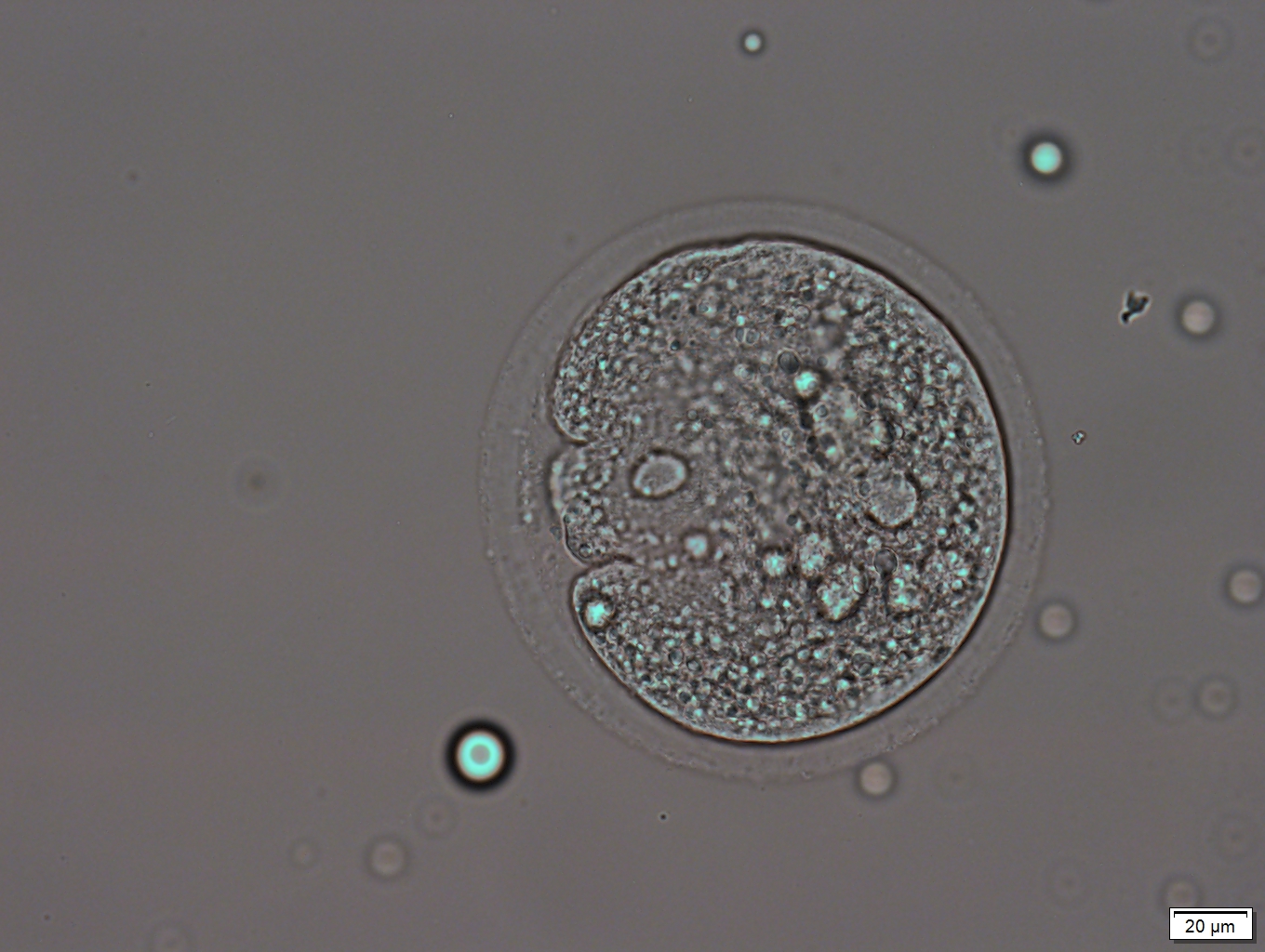

Supplement: Supplementary file 1 [file DataSheet3.ZIP › Figure3í╠/Annex ó⌡/3.6mM (2).jpg]

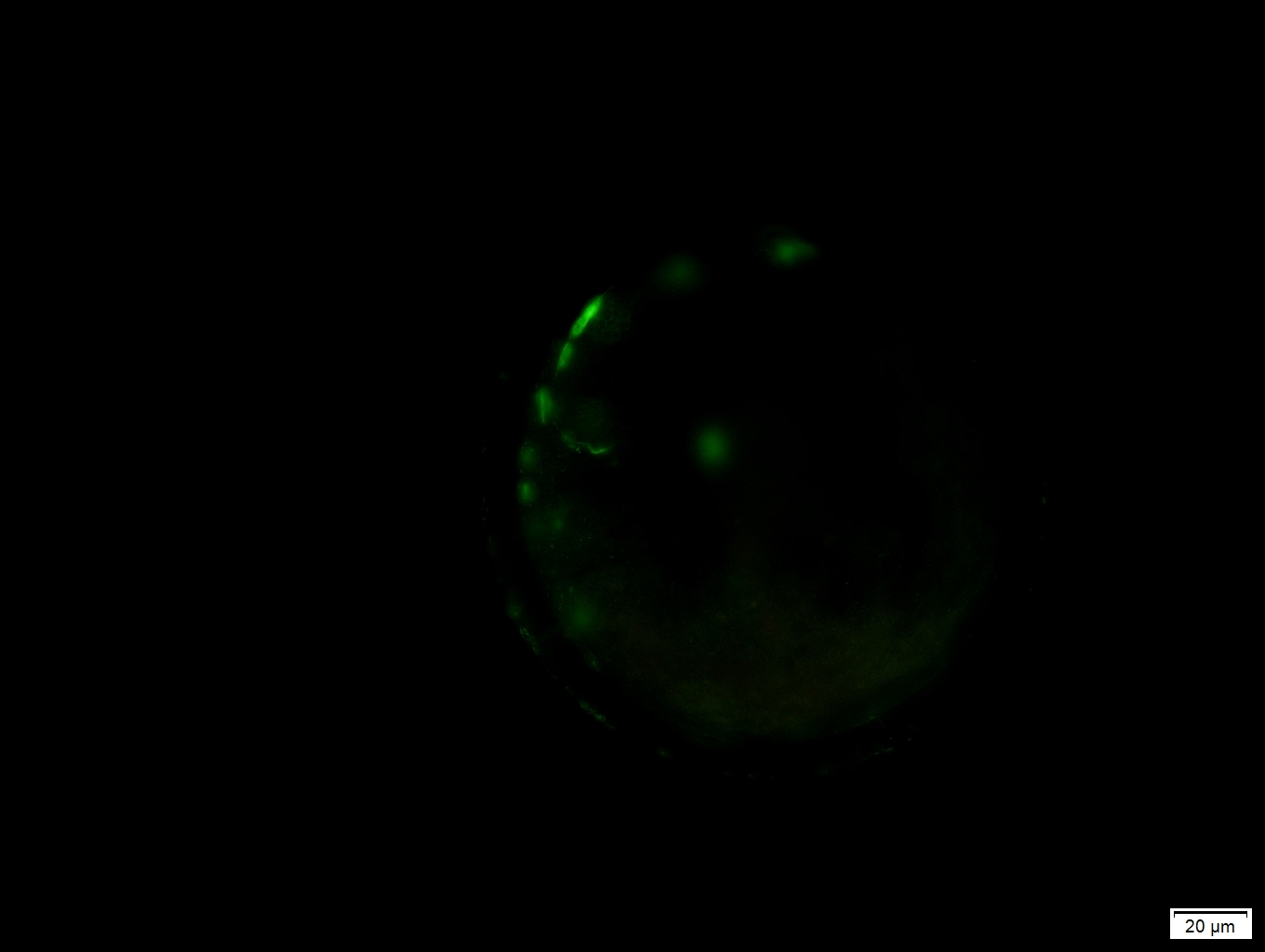

Supplement: Supplementary file 1 [file DataSheet3.ZIP › Figure3í╠/Annex ó⌡/3.6mM.jpg]

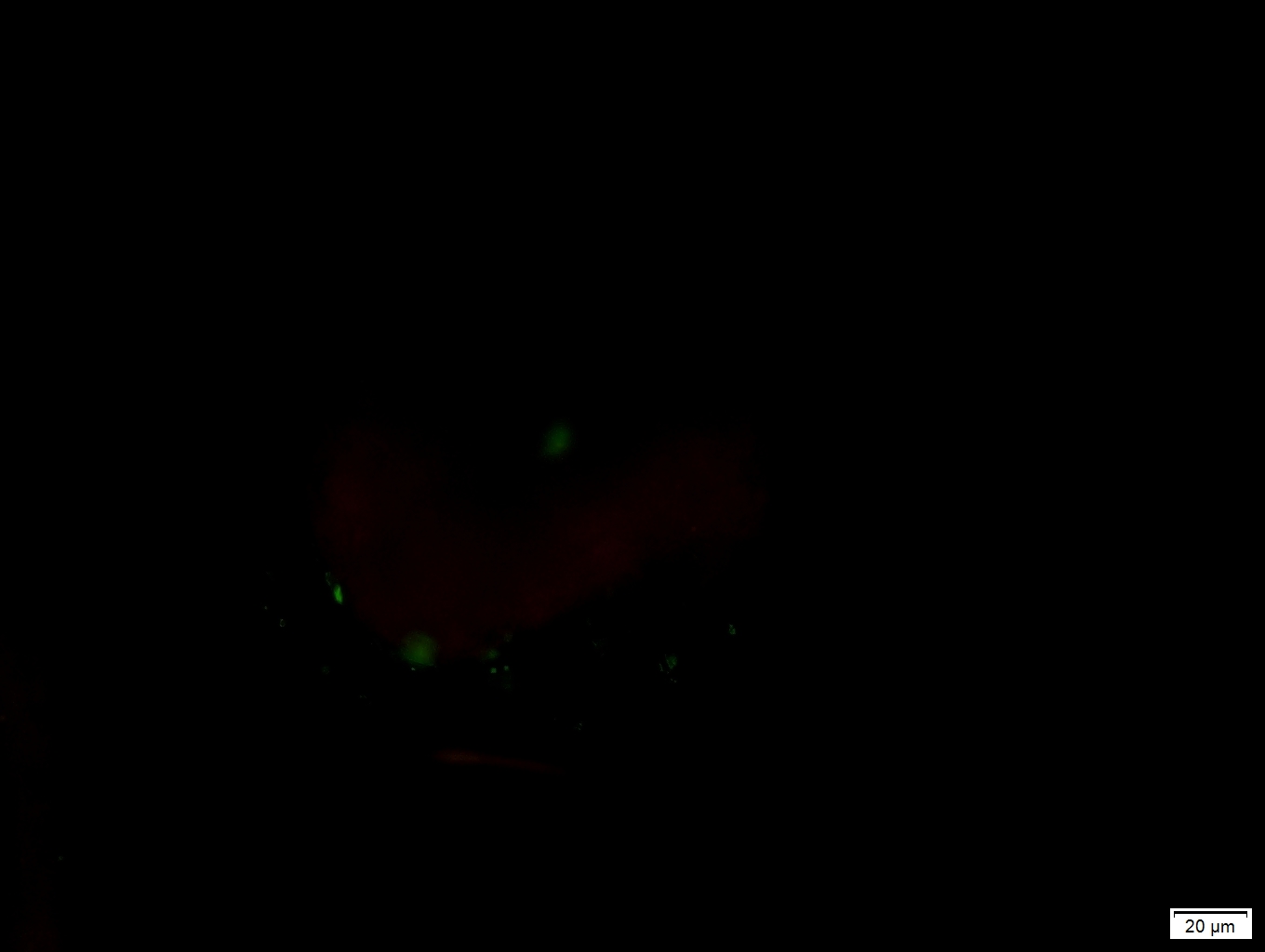

Supplement: Supplementary file 1 [file DataSheet3.ZIP › Figure3í╠/Annex ó⌡/C (1).jpg]

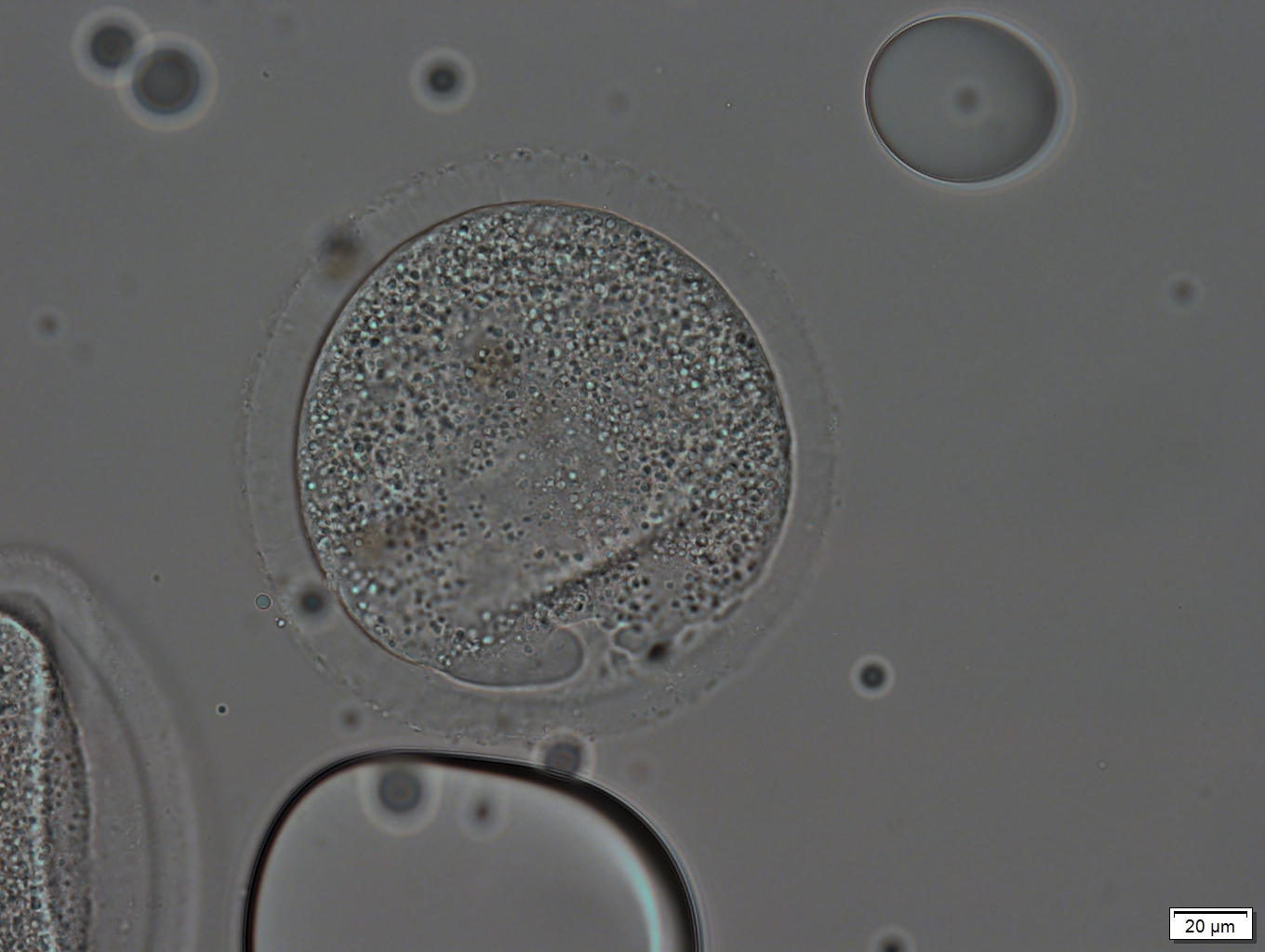

Supplement: Supplementary file 1 [file DataSheet3.ZIP › Figure3í╠/Annex ó⌡/C (2).jpg]

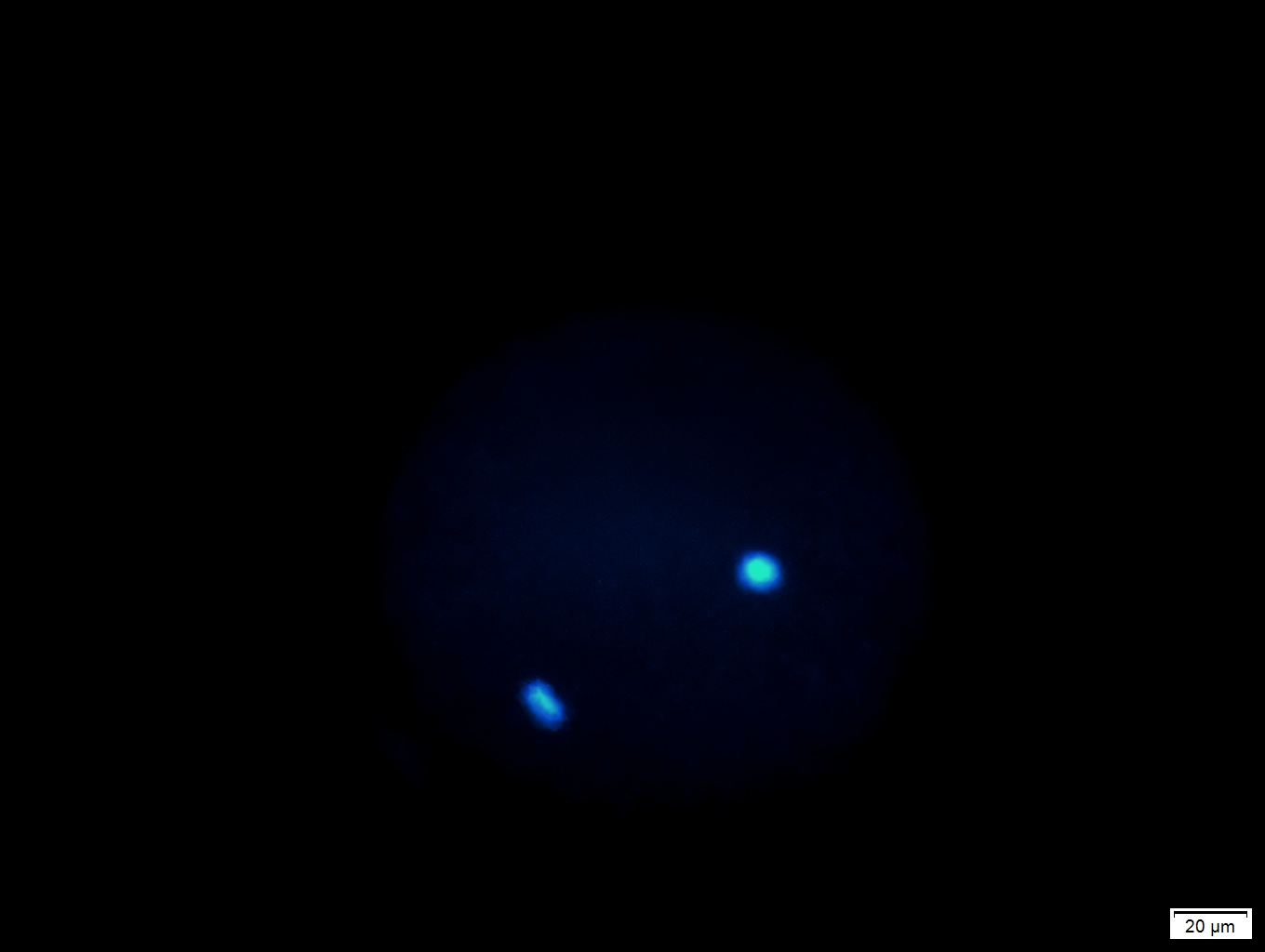

Supplement: Supplementary file 1 [file DataSheet3.ZIP › Figure3í╠/Caspase-3/Caspase3-1.2mM (2).jpg]

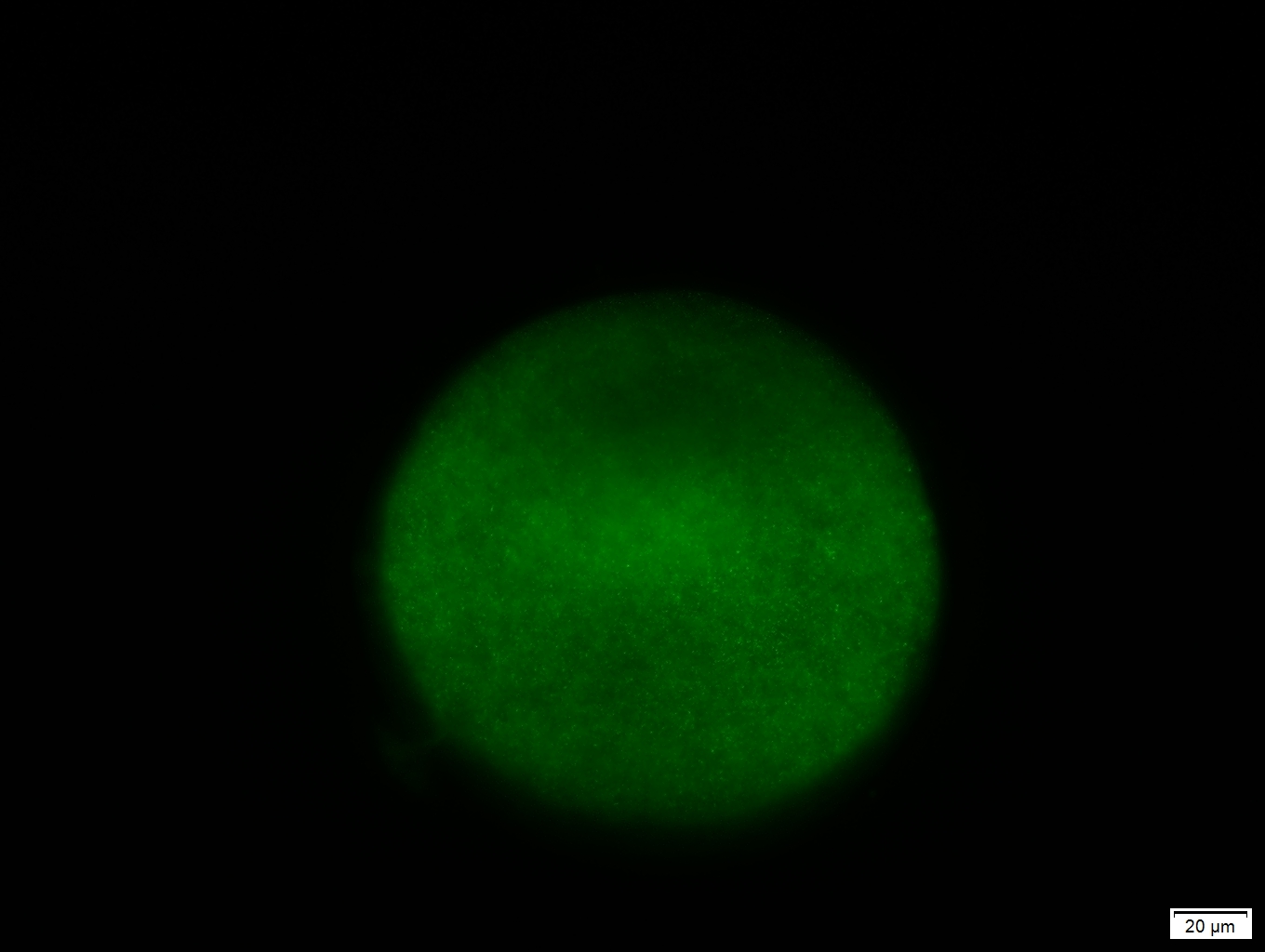

Supplement: Supplementary file 1 [file DataSheet3.ZIP › Figure3í╠/Caspase-3/Caspase3-1.2mM(1).jpg]

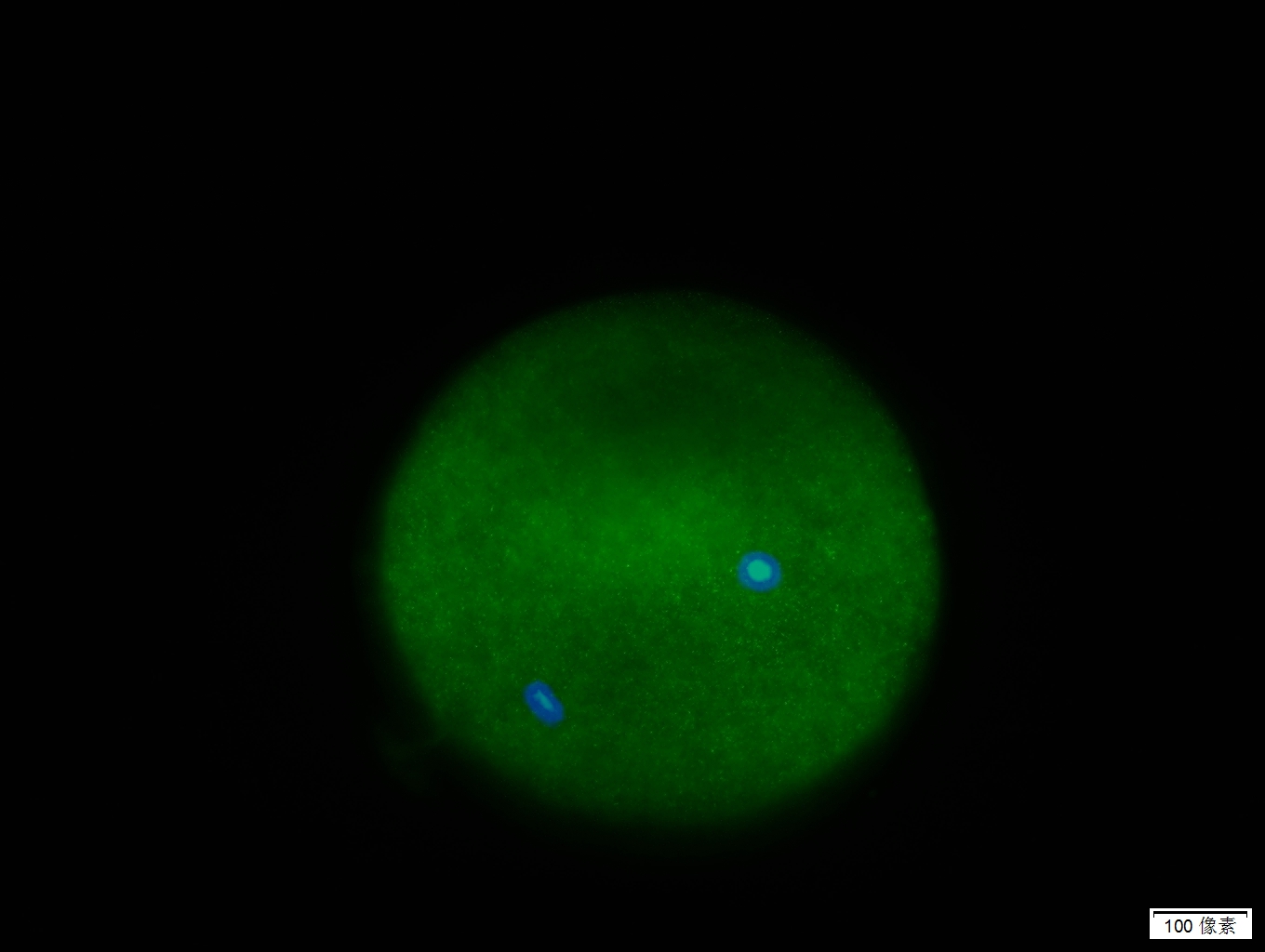

Supplement: Supplementary file 1 [file DataSheet3.ZIP › Figure3í╠/Caspase-3/Caspase3-1.2mM.jpg]

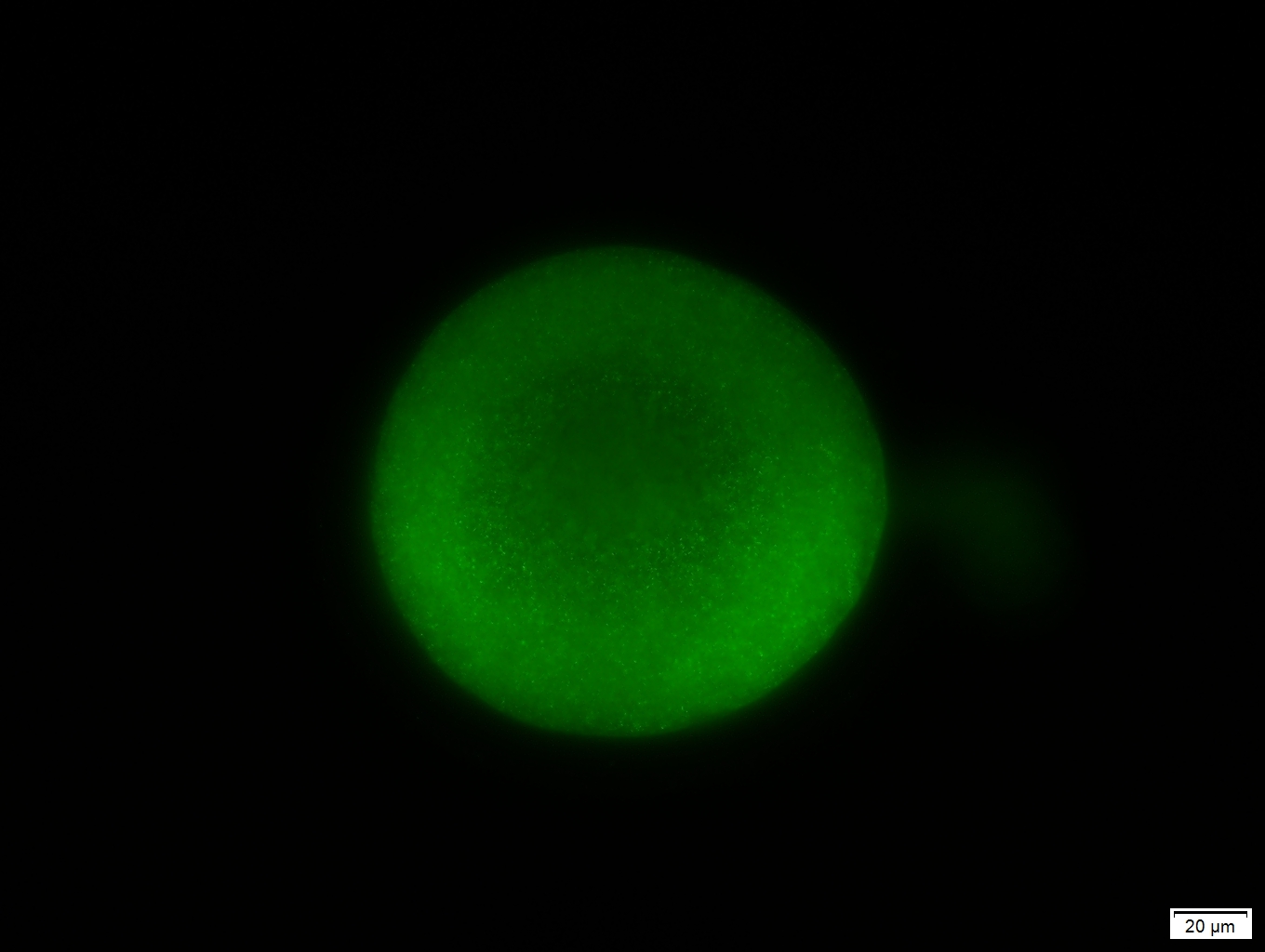

Supplement: Supplementary file 1 [file DataSheet3.ZIP › Figure3í╠/Caspase-3/Caspase-3-3.6mM (1).jpg]

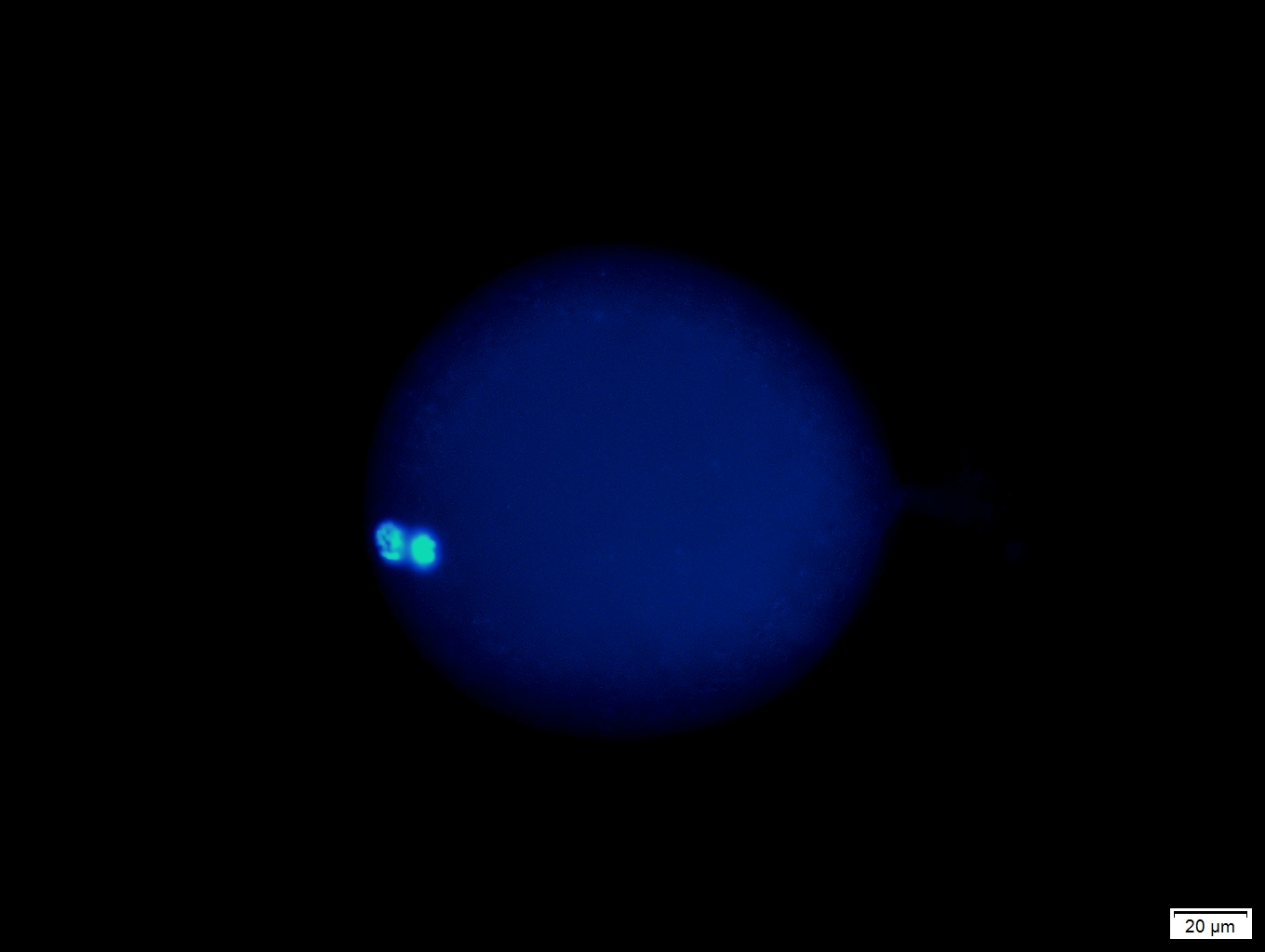

Supplement: Supplementary file 1 [file DataSheet3.ZIP › Figure3í╠/Caspase-3/Caspase-3-3.6mM (2).jpg]

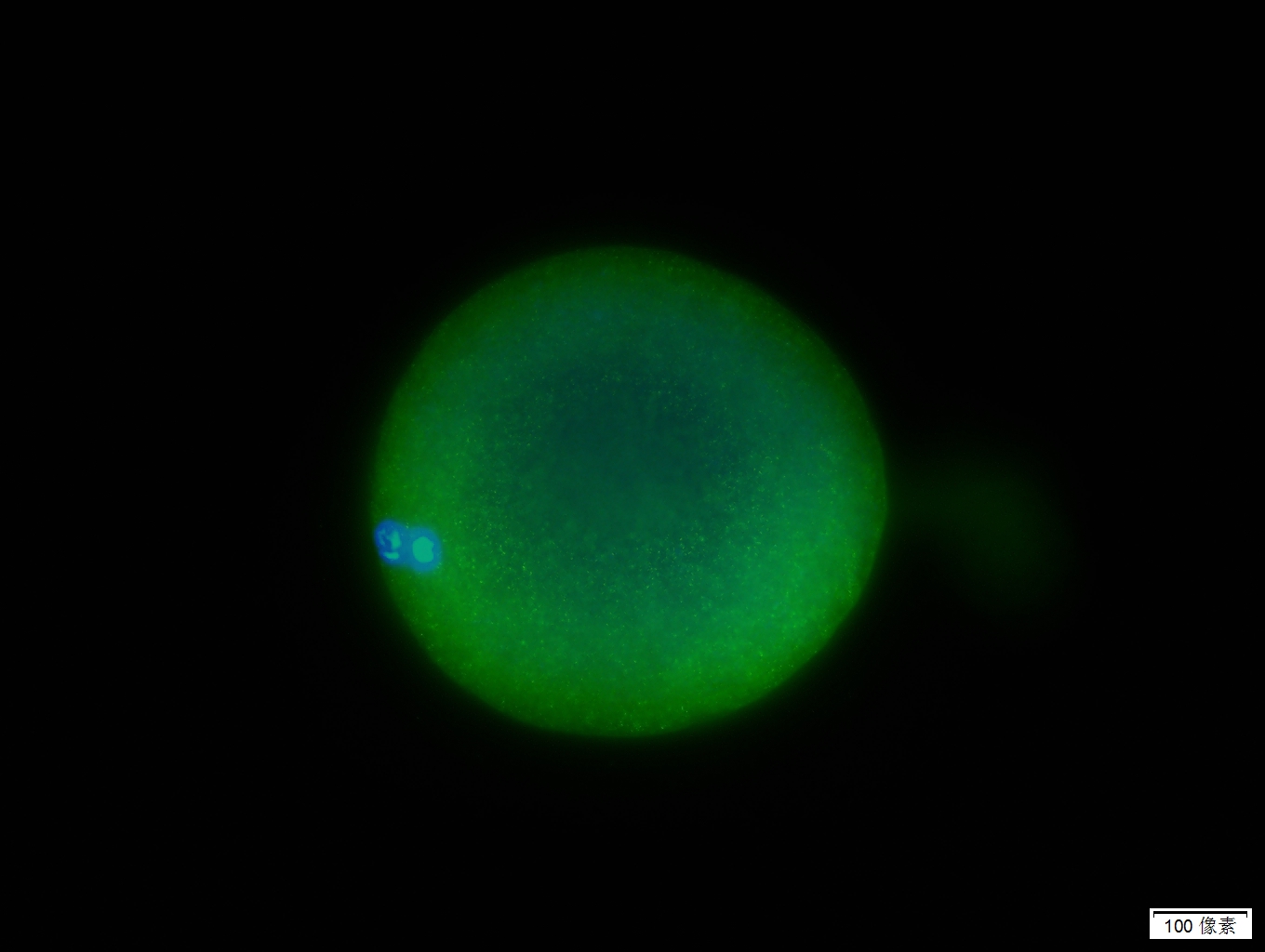

Supplement: Supplementary file 1 [file DataSheet3.ZIP › Figure3í╠/Caspase-3/Caspase3-3.6mM.jpg]

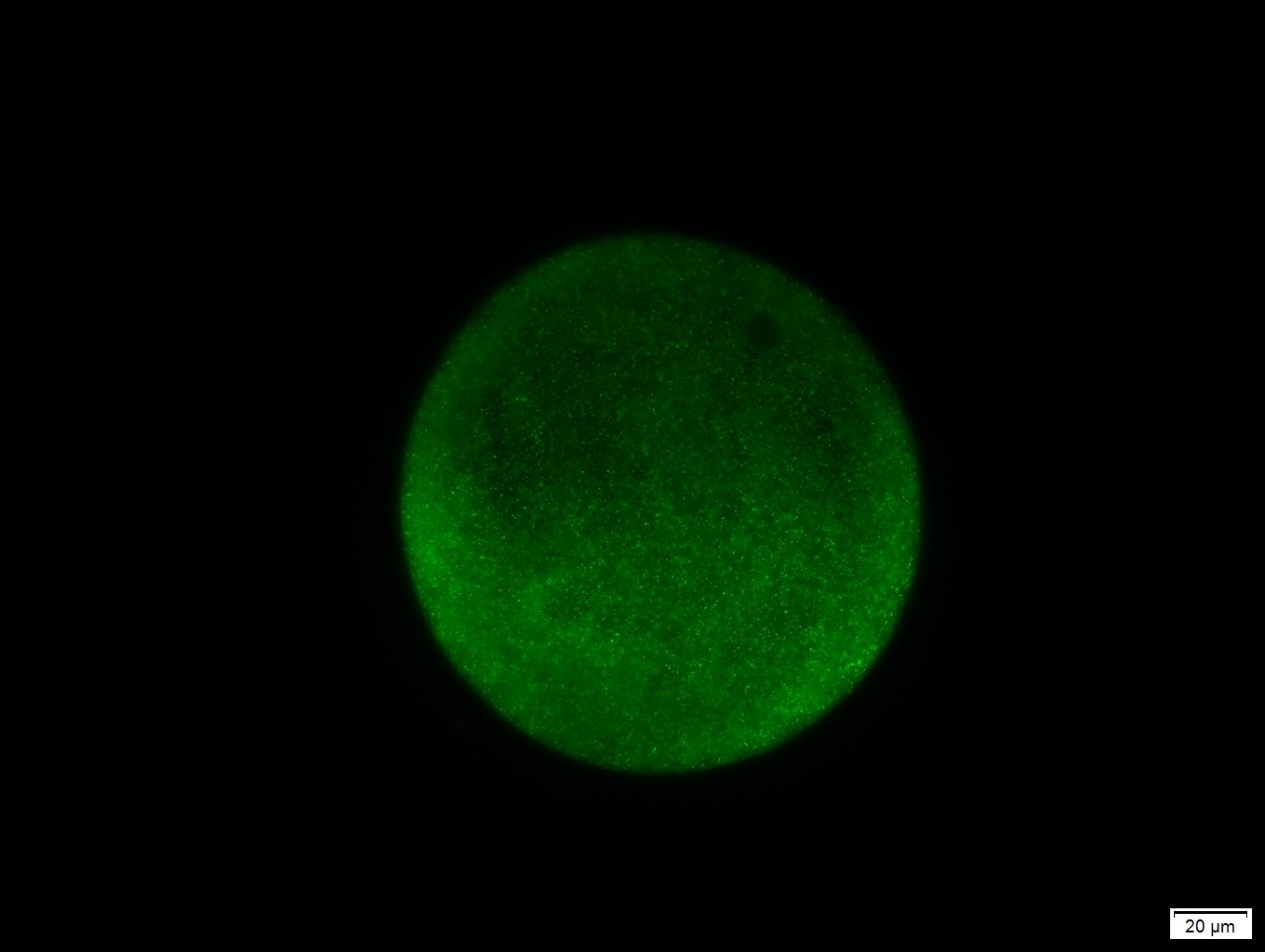

Supplement: Supplementary file 1 [file DataSheet3.ZIP › Figure3í╠/Caspase-3/Caspase3-C (1).jpg]

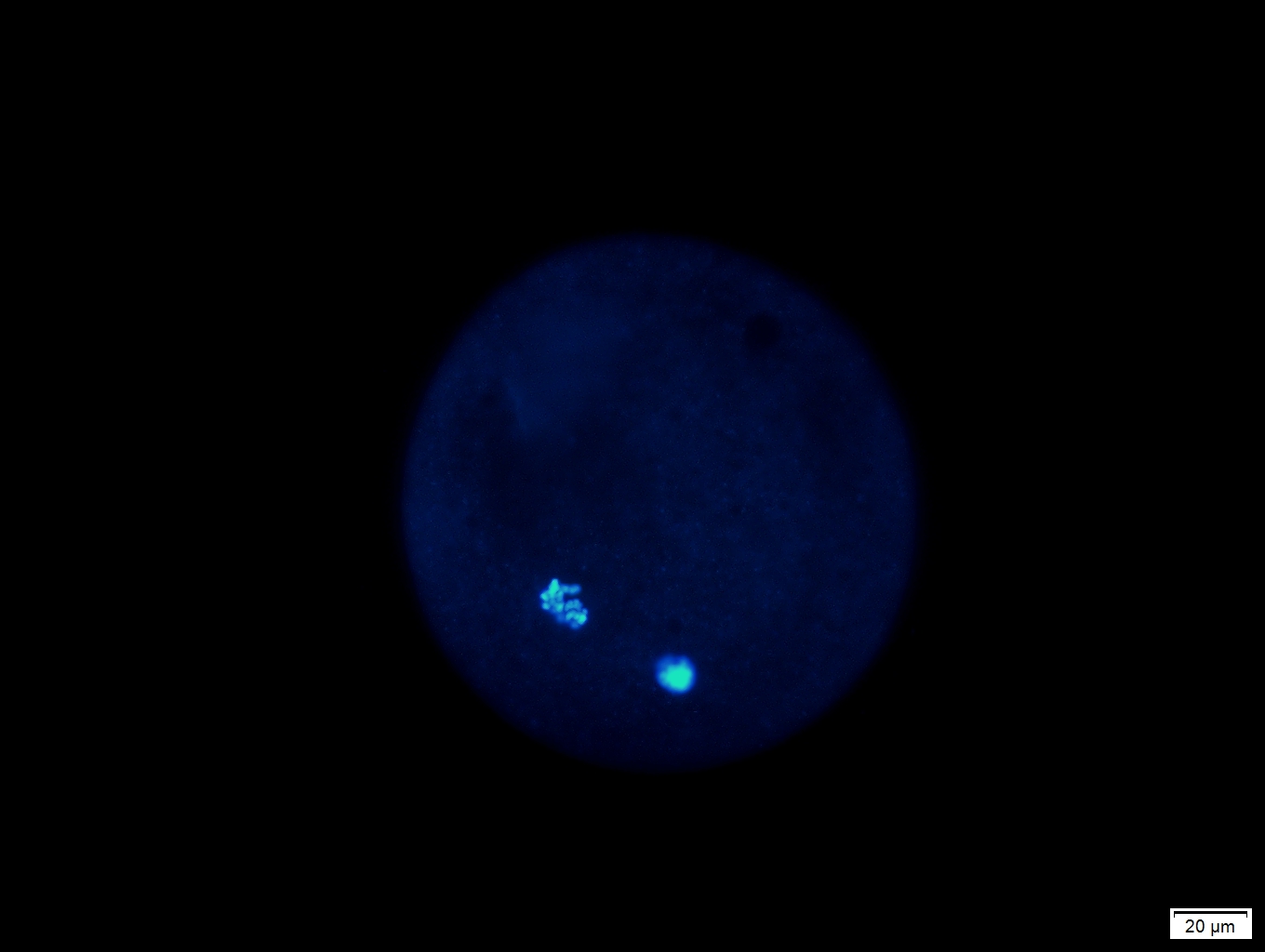

Supplement: Supplementary file 1 [file DataSheet3.ZIP › Figure3í╠/Caspase-3/Caspase3-C (2).jpg]

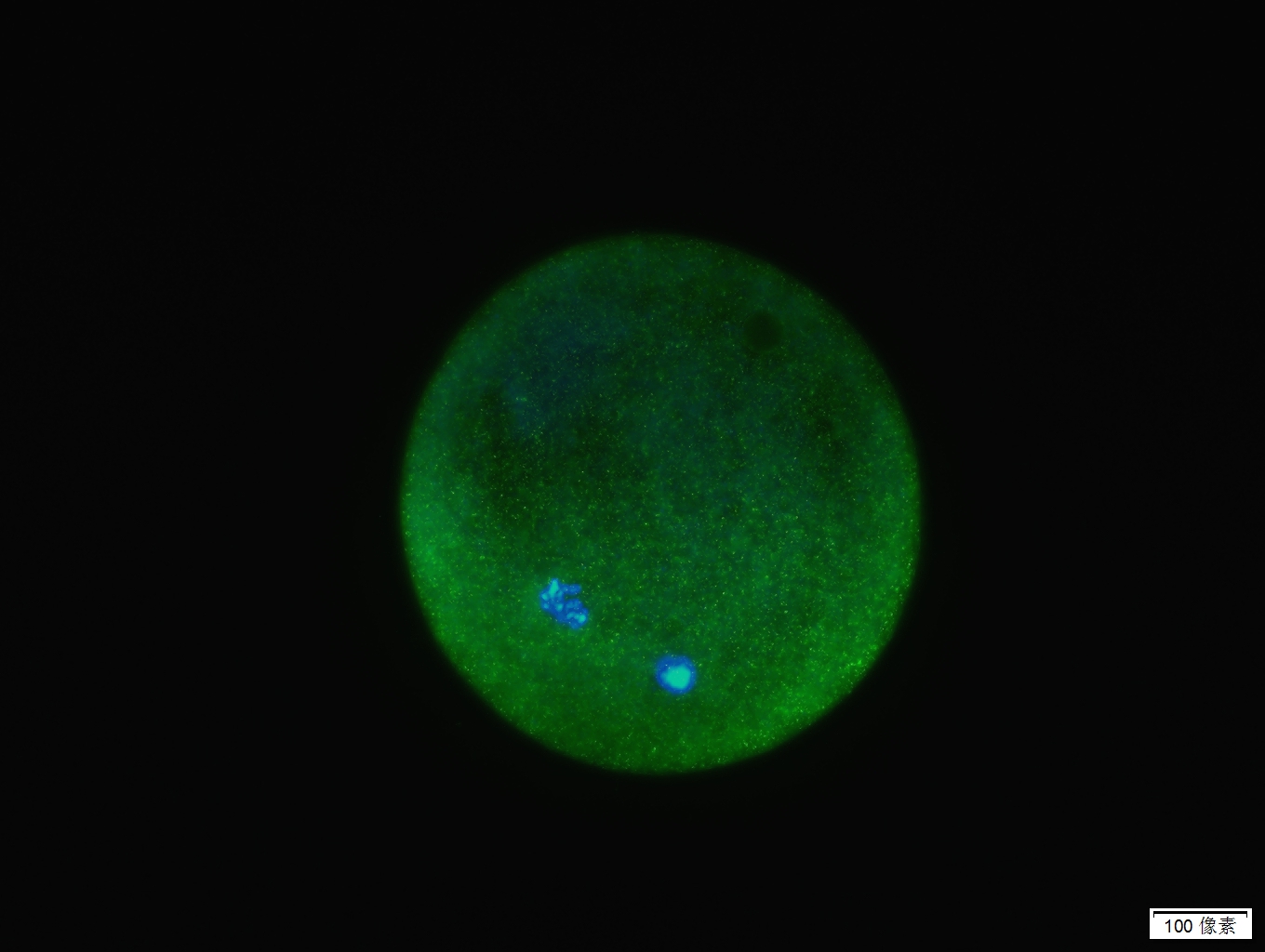

Supplement: Supplementary file 1 [file DataSheet3.ZIP › Figure3í╠/Caspase-3/Caspase3-C.jpg]

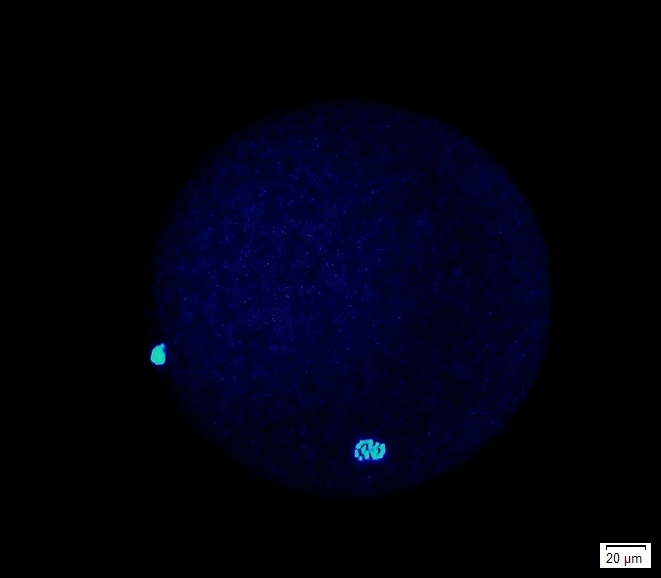

Supplement: Supplementary file 2 [file DataSheet4.ZIP › Figure4í╠/PDHA1/1 (1).jpg]

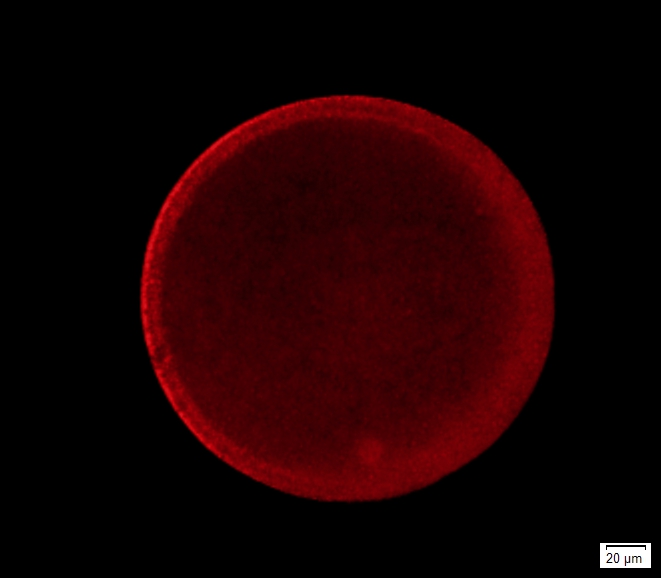

Supplement: Supplementary file 2 [file DataSheet4.ZIP › Figure4í╠/PDHA1/1 (2).jpg]

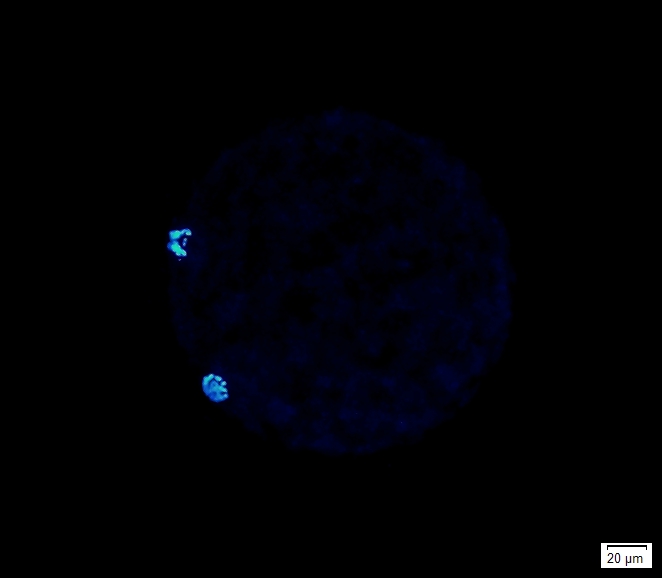

Supplement: Supplementary file 2 [file DataSheet4.ZIP › Figure4í╠/PDHA1/3 (1).jpg]

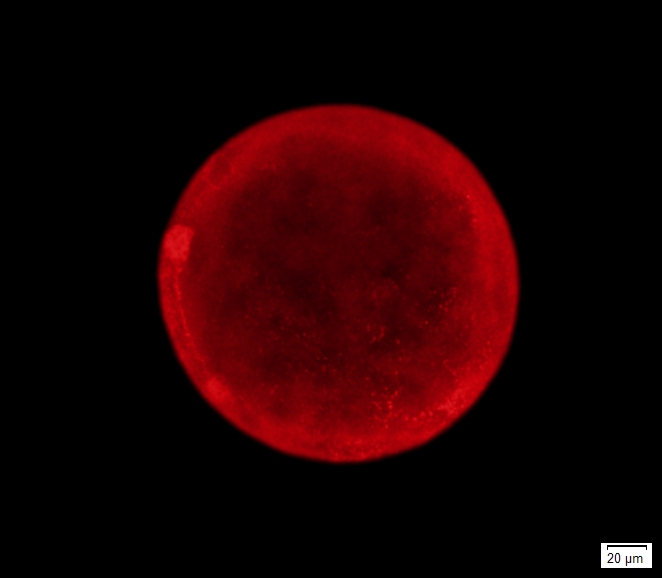

Supplement: Supplementary file 2 [file DataSheet4.ZIP › Figure4í╠/PDHA1/3 (2).jpg]

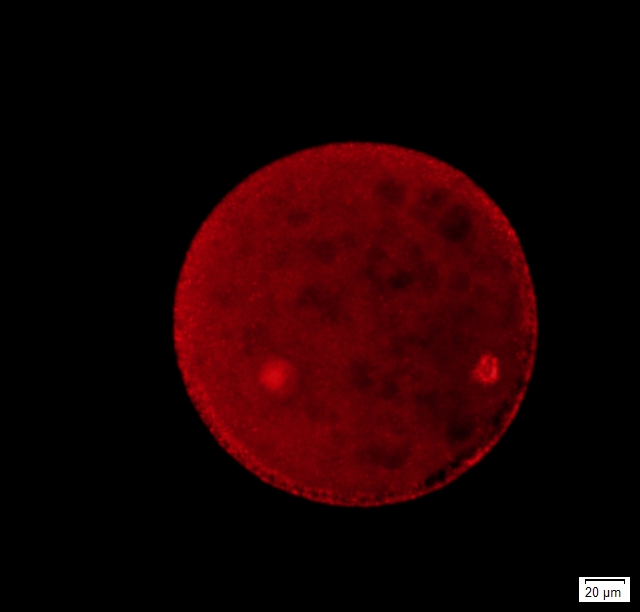

Supplement: Supplementary file 2 [file DataSheet4.ZIP › Figure4í╠/PDHA1/C (1).jpg]

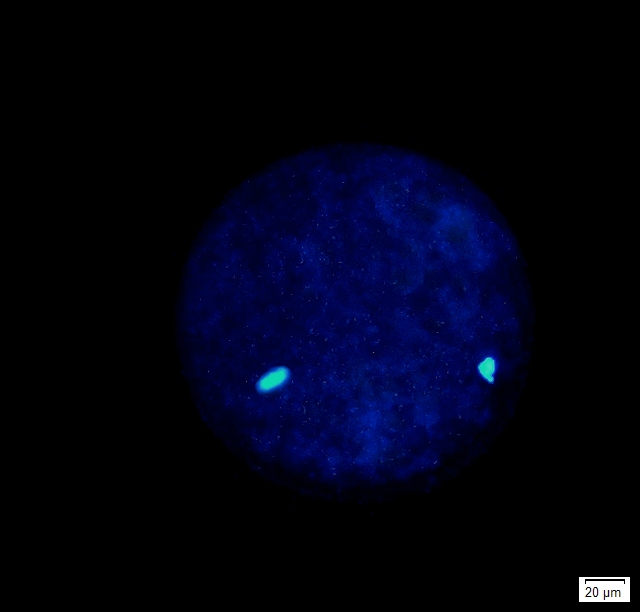

Supplement: Supplementary file 2 [file DataSheet4.ZIP › Figure4í╠/PDHA1/C (2).jpg]

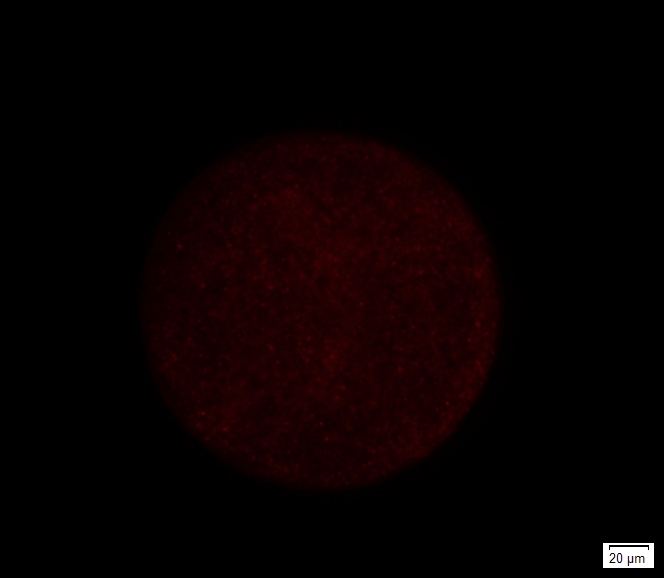

Supplement: Supplementary file 2 [file DataSheet4.ZIP › Figure4í╠/p-PDH/1 (1).jpg]

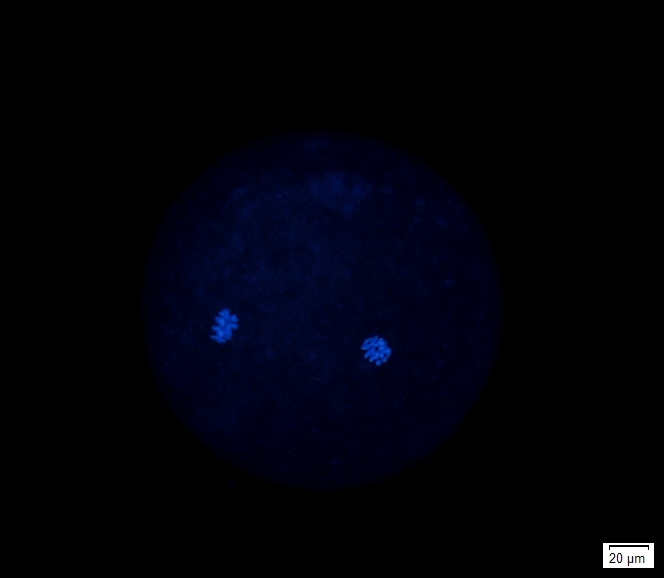

Supplement: Supplementary file 2 [file DataSheet4.ZIP › Figure4í╠/p-PDH/1 (2).jpg]

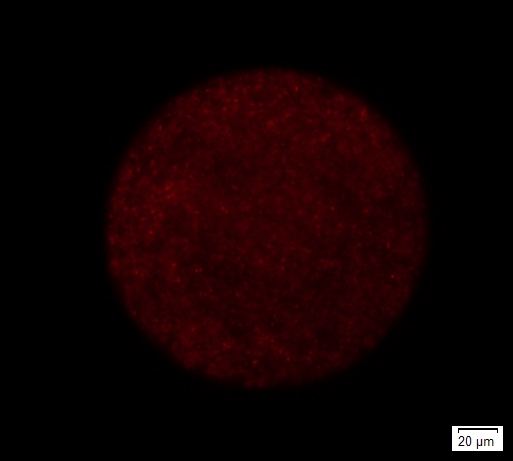

Supplement: Supplementary file 2 [file DataSheet4.ZIP › Figure4í╠/p-PDH/3 (1).jpg]

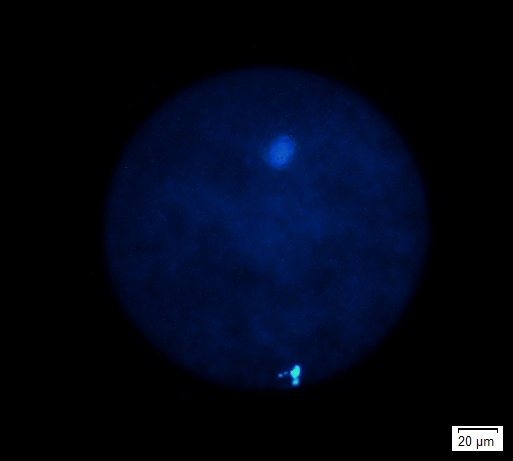

Supplement: Supplementary file 2 [file DataSheet4.ZIP › Figure4í╠/p-PDH/3 (2).jpg]

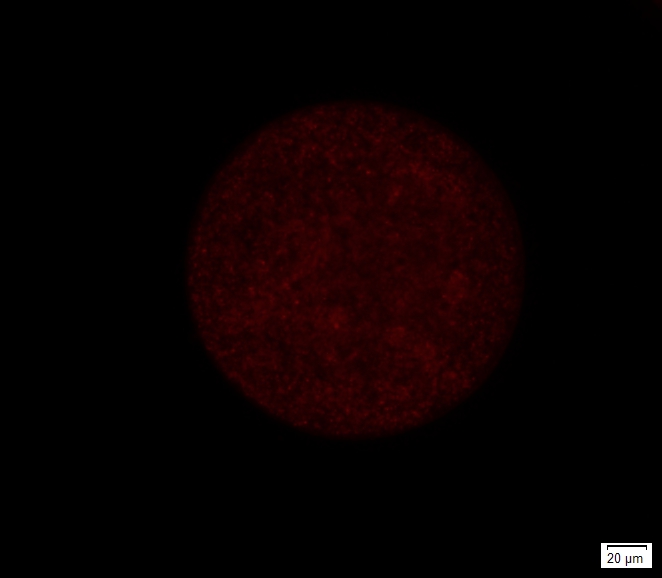

Supplement: Supplementary file 2 [file DataSheet4.ZIP › Figure4í╠/p-PDH/C (1).jpg]

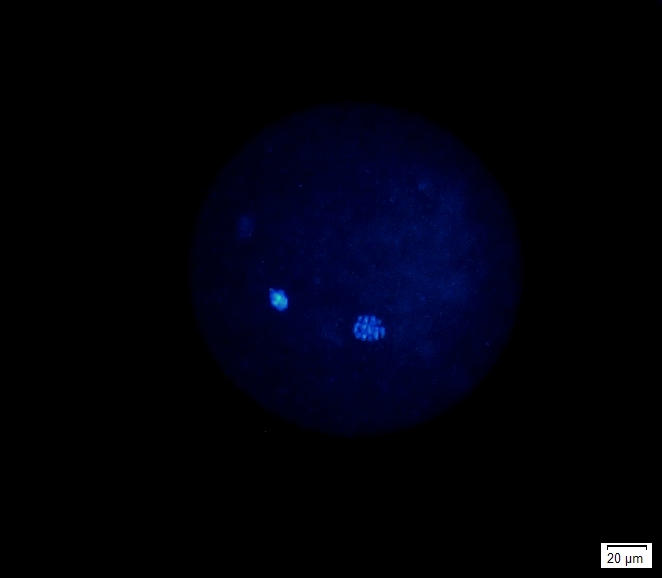

Supplement: Supplementary file 2 [file DataSheet4.ZIP › Figure4í╠/p-PDH/C (2).jpg]

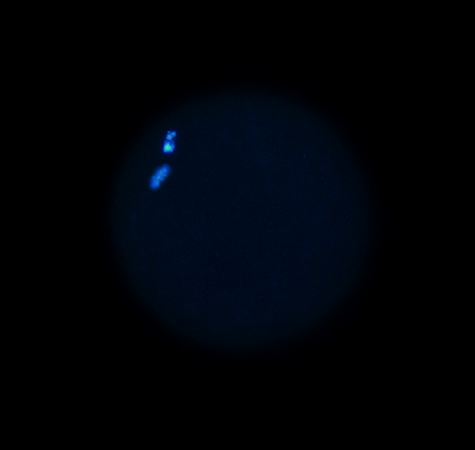

Supplement: Supplementary file 2 [file DataSheet4.ZIP › Figure4í╠/SCOT/3.6mM (2).tif]

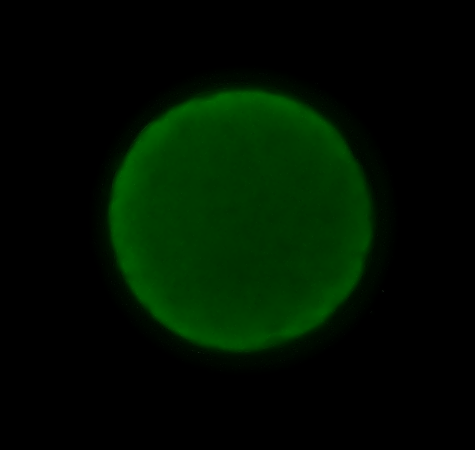

Supplement: Supplementary file 2 [file DataSheet4.ZIP › Figure4í╠/SCOT/3.6mM (3).tif]

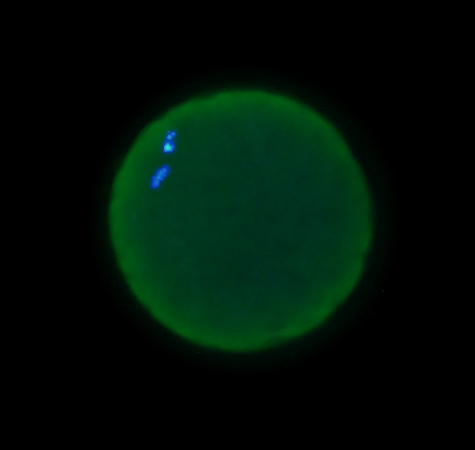

Supplement: Supplementary file 2 [file DataSheet4.ZIP › Figure4í╠/SCOT/3íú6mM (1).tif]

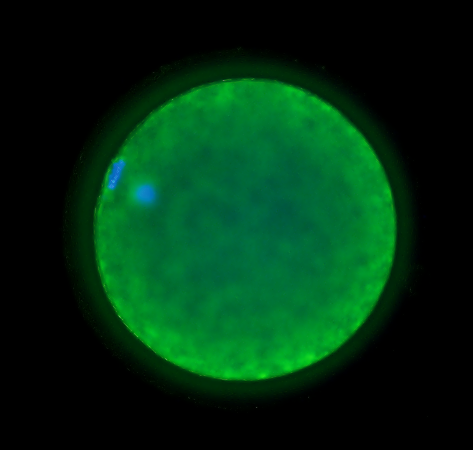

Supplement: Supplementary file 2 [file DataSheet4.ZIP › Figure4í╠/SCOT/C (1).tif]

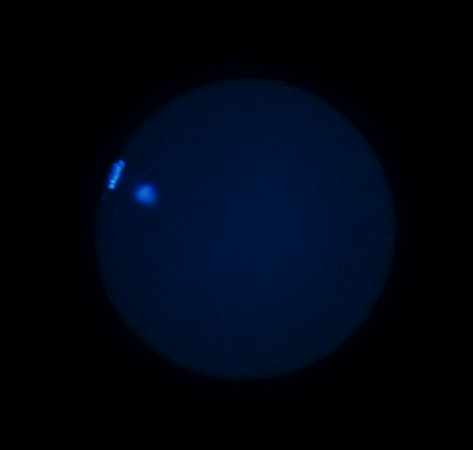

Supplement: Supplementary file 2 [file DataSheet4.ZIP › Figure4í╠/SCOT/C (2).tif]

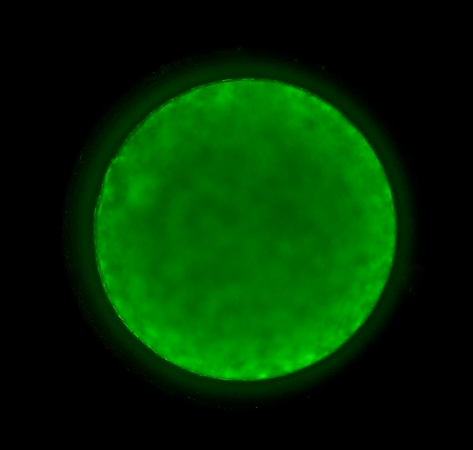

Supplement: Supplementary file 2 [file DataSheet4.ZIP › Figure4í╠/SCOT/C (3).tif]

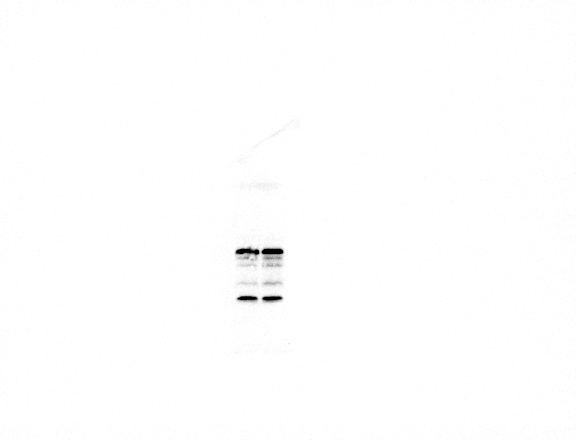

Supplement: Supplementary file 2 [file DataSheet4.ZIP › Figure4í╠/WB╓╪╕┤/p1-PDH1.tif]

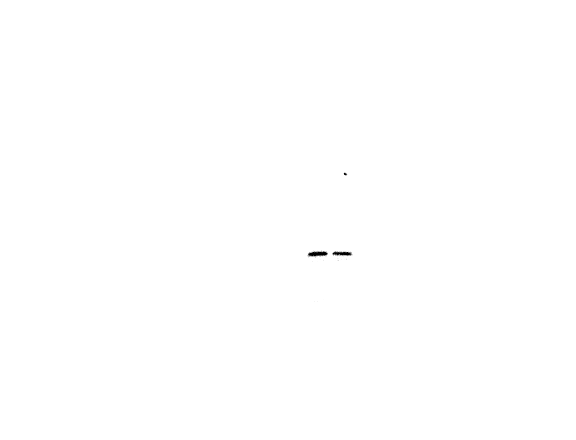

Supplement: Supplementary file 2 [file DataSheet4.ZIP › Figure4í╠/WB╓╪╕┤/PDH.tif]

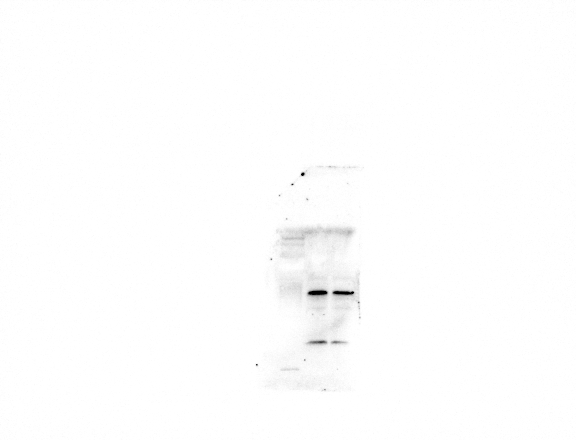

Supplement: Supplementary file 2 [file DataSheet4.ZIP › Figure4í╠/WB╓╪╕┤/PDH1.tif]

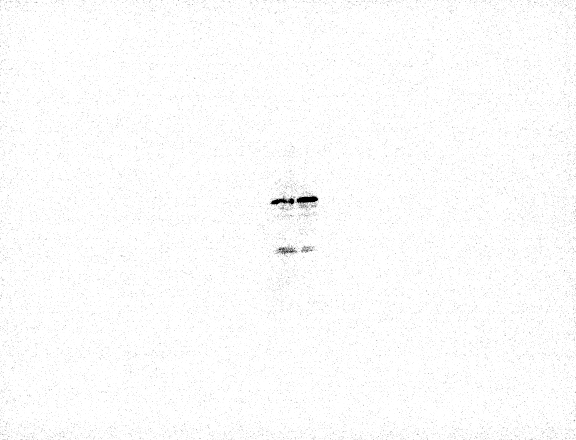

Supplement: Supplementary file 2 [file DataSheet4.ZIP › Figure4í╠/WB╓╪╕┤/p-PDH.tif]

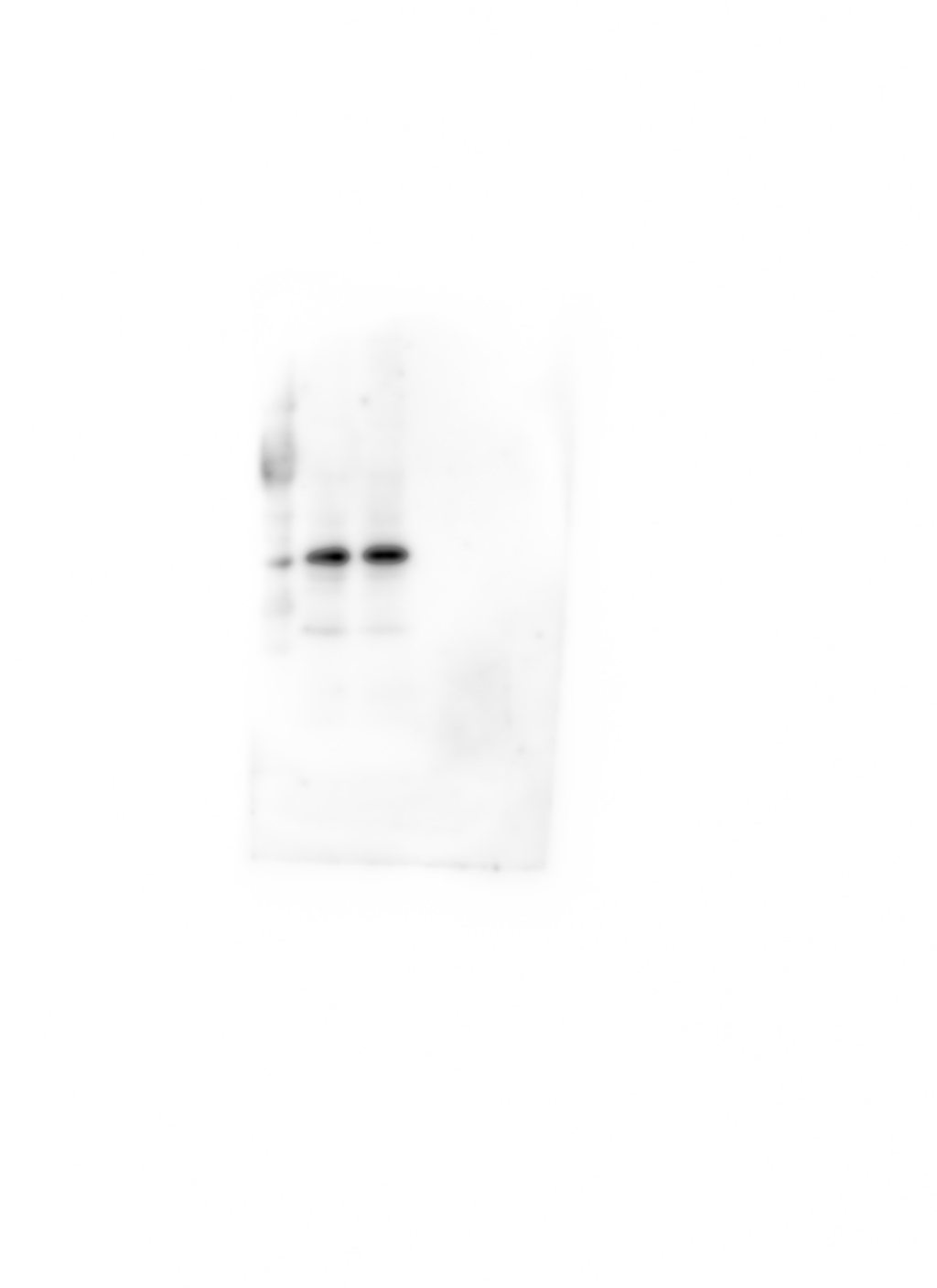

Supplement: Supplementary file 2 [file DataSheet4.ZIP › Figure4í╠/WB╓╪╕┤/p-PDHó┘.tif]

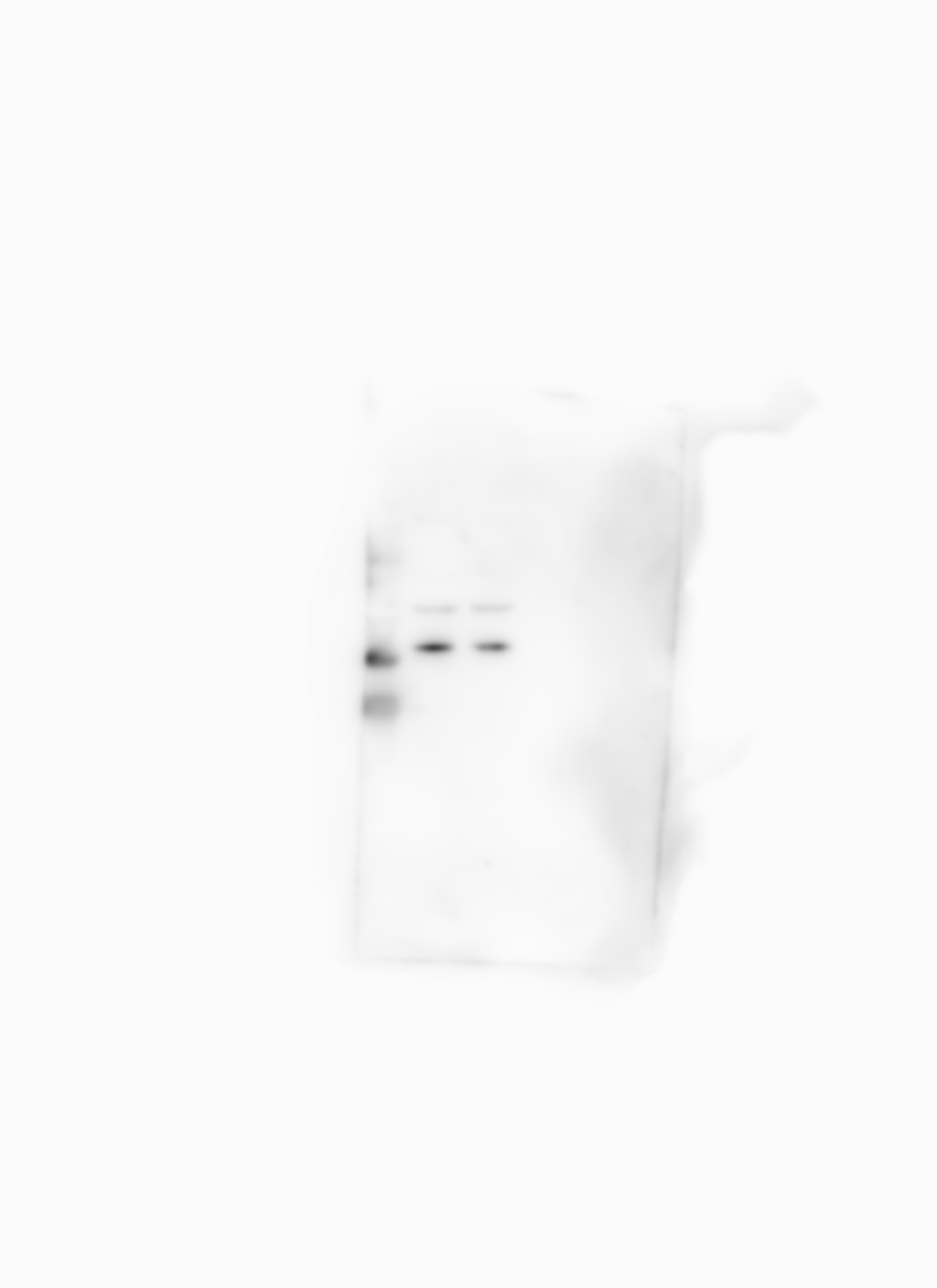

Supplement: Supplementary file 2 [file DataSheet4.ZIP › Figure4í╠/WB╓╪╕┤/zky b-actin.tif]

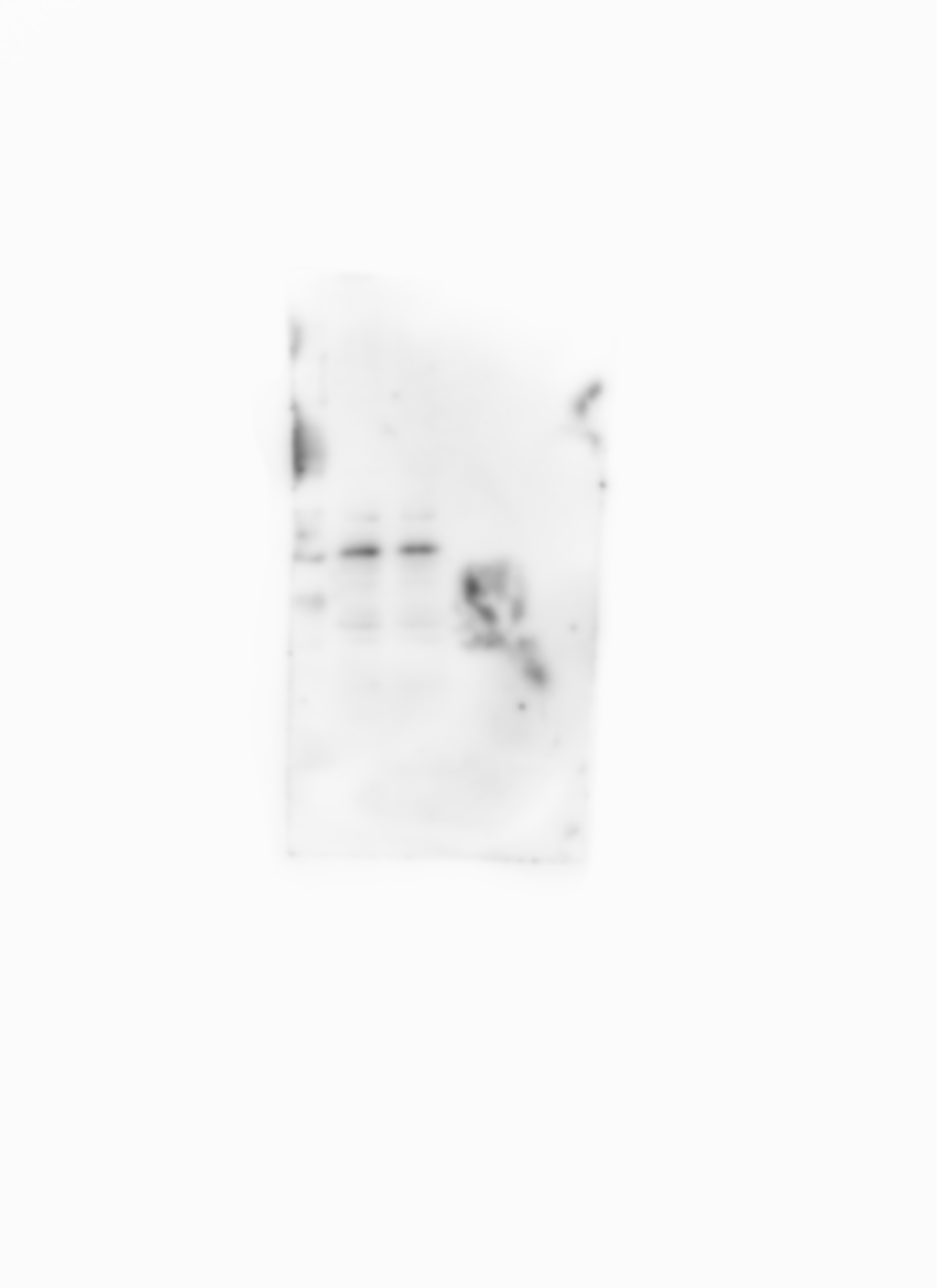

Supplement: Supplementary file 2 [file DataSheet4.ZIP › Figure4í╠/WB╓╪╕┤/zky pdh.tif]

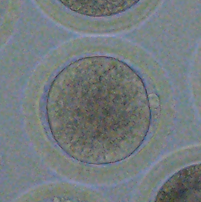

Supplement: Supplementary file 3 [file DataSheet1.ZIP › Figure1í╠/1.2mM (1).tif]

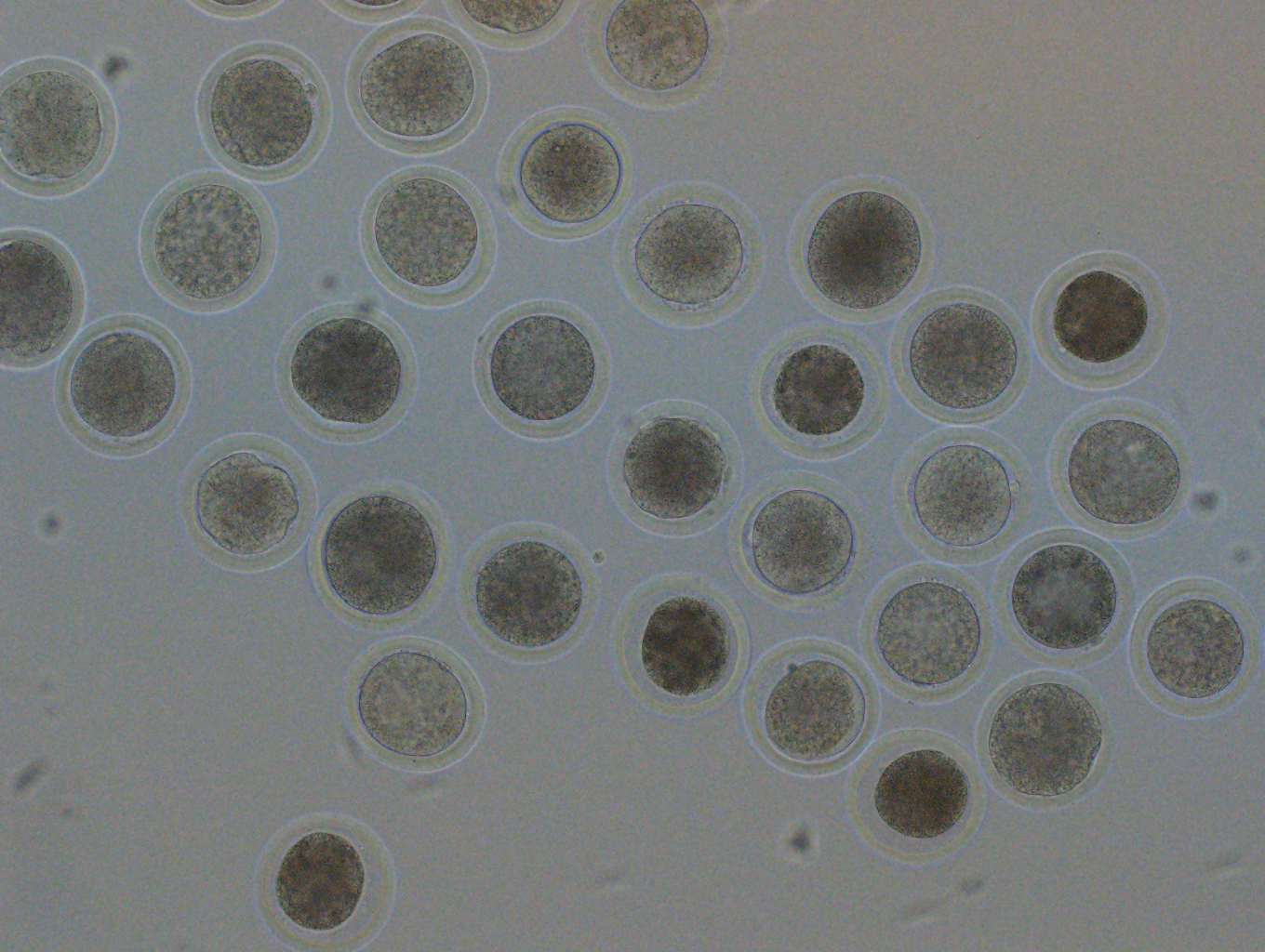

Supplement: Supplementary file 3 [file DataSheet1.ZIP › Figure1í╠/1.2mM (2).tif]

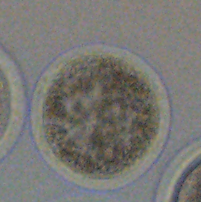

Supplement: Supplementary file 3 [file DataSheet1.ZIP › Figure1í╠/3.6mM (1).tif]

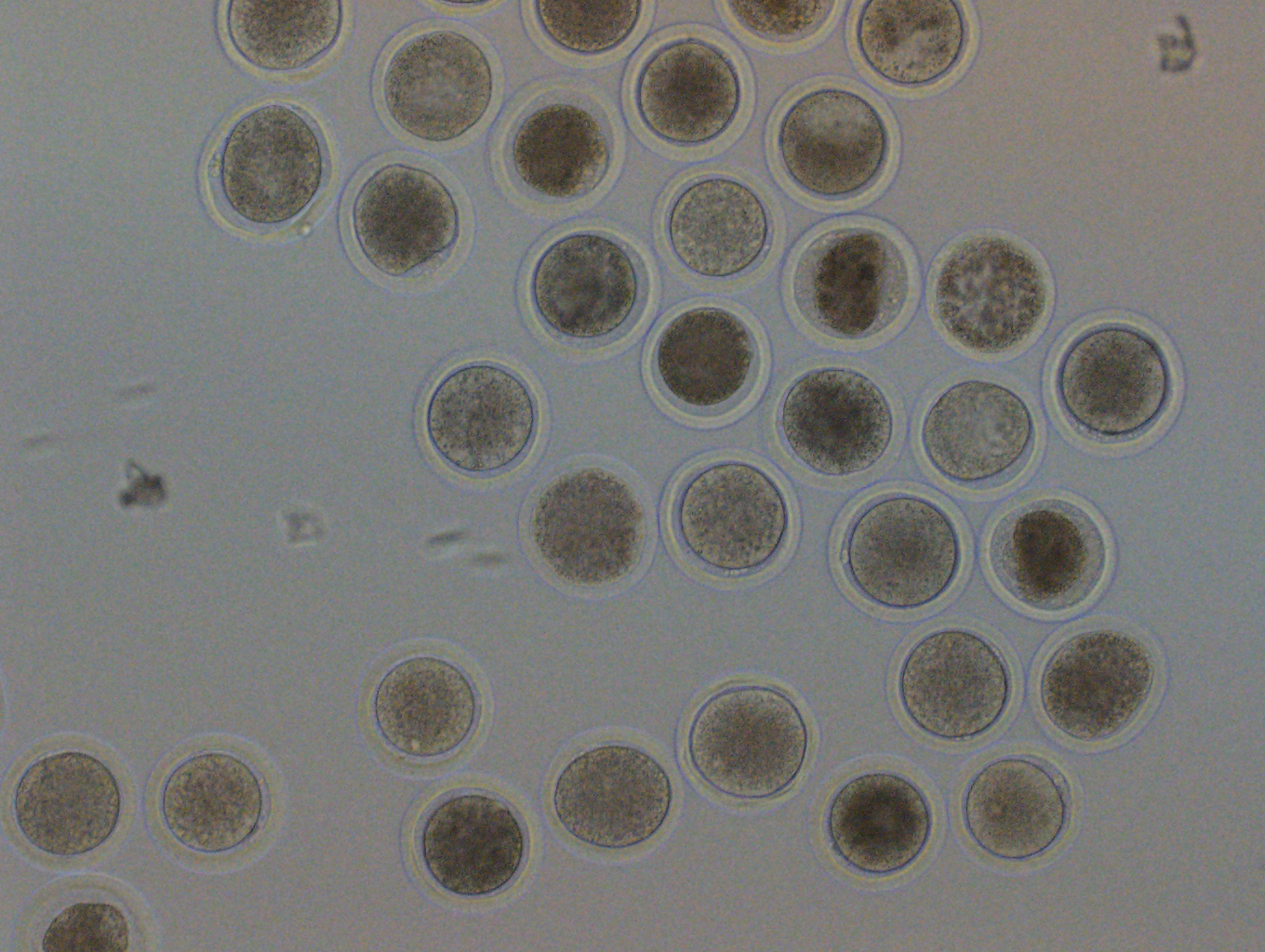

Supplement: Supplementary file 3 [file DataSheet1.ZIP › Figure1í╠/3.6mM (2).tif]

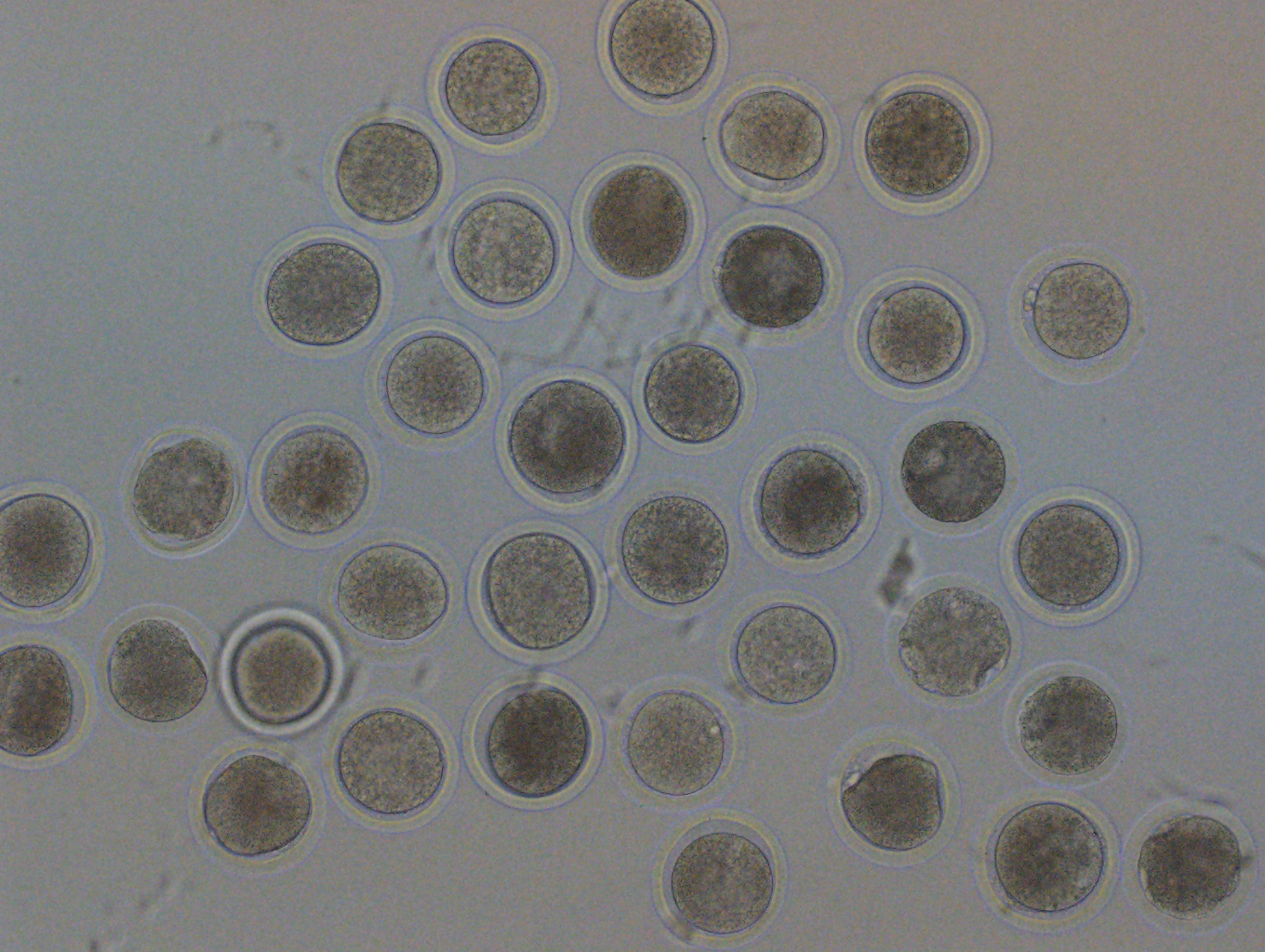

Supplement: Supplementary file 3 [file DataSheet1.ZIP › Figure1í╠/Control (1).tif]

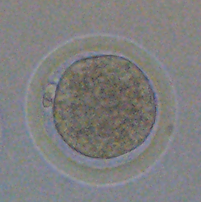

Supplement: Supplementary file 3 [file DataSheet1.ZIP › Figure1í╠/Control (2).tif]

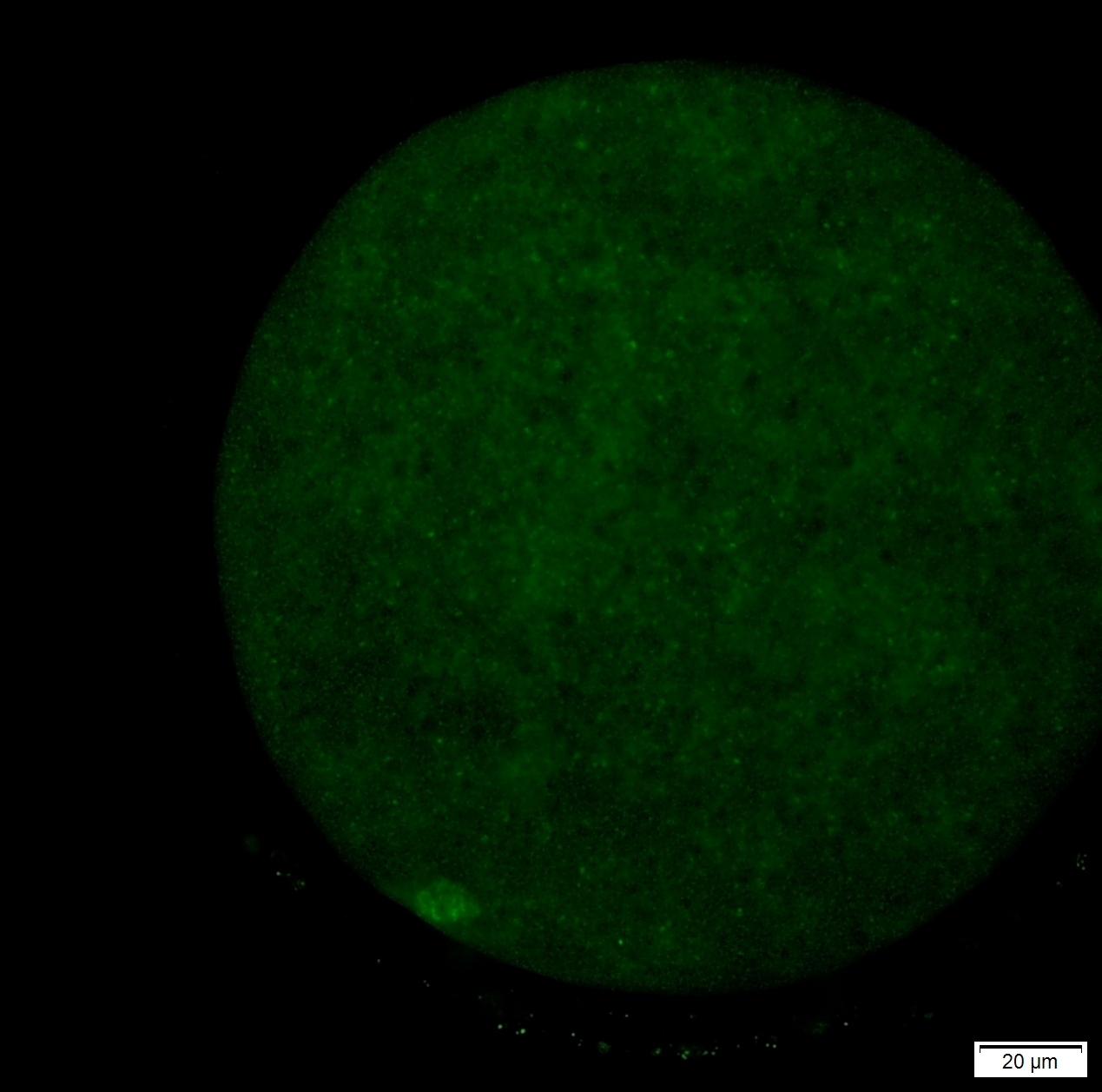

Supplement: Supplementary file 4 [file DataSheet6.ZIP › Figure6/Ace/BHB (1).jpg]

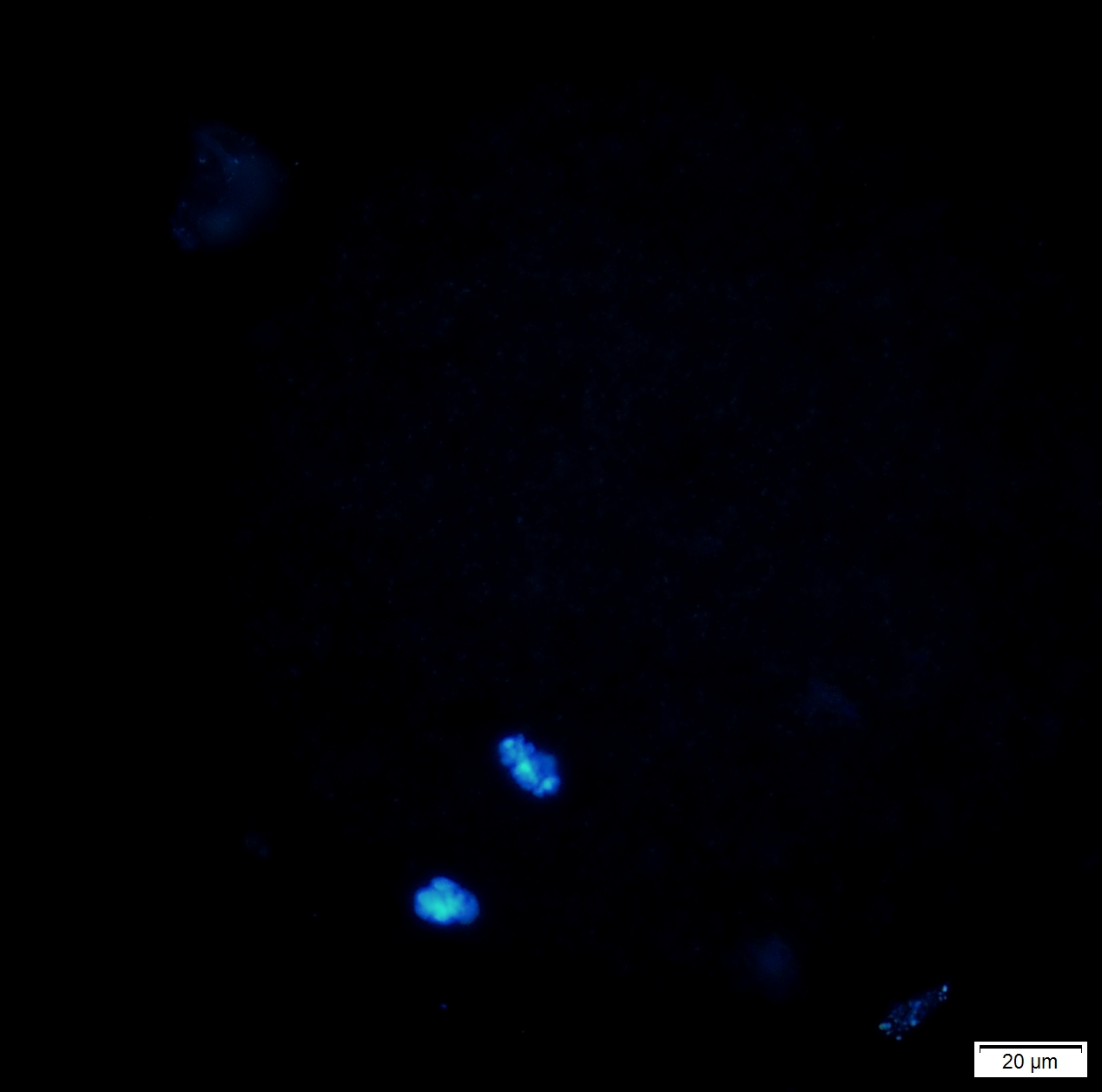

Supplement: Supplementary file 4 [file DataSheet6.ZIP › Figure6/Ace/BHB (2).jpg]

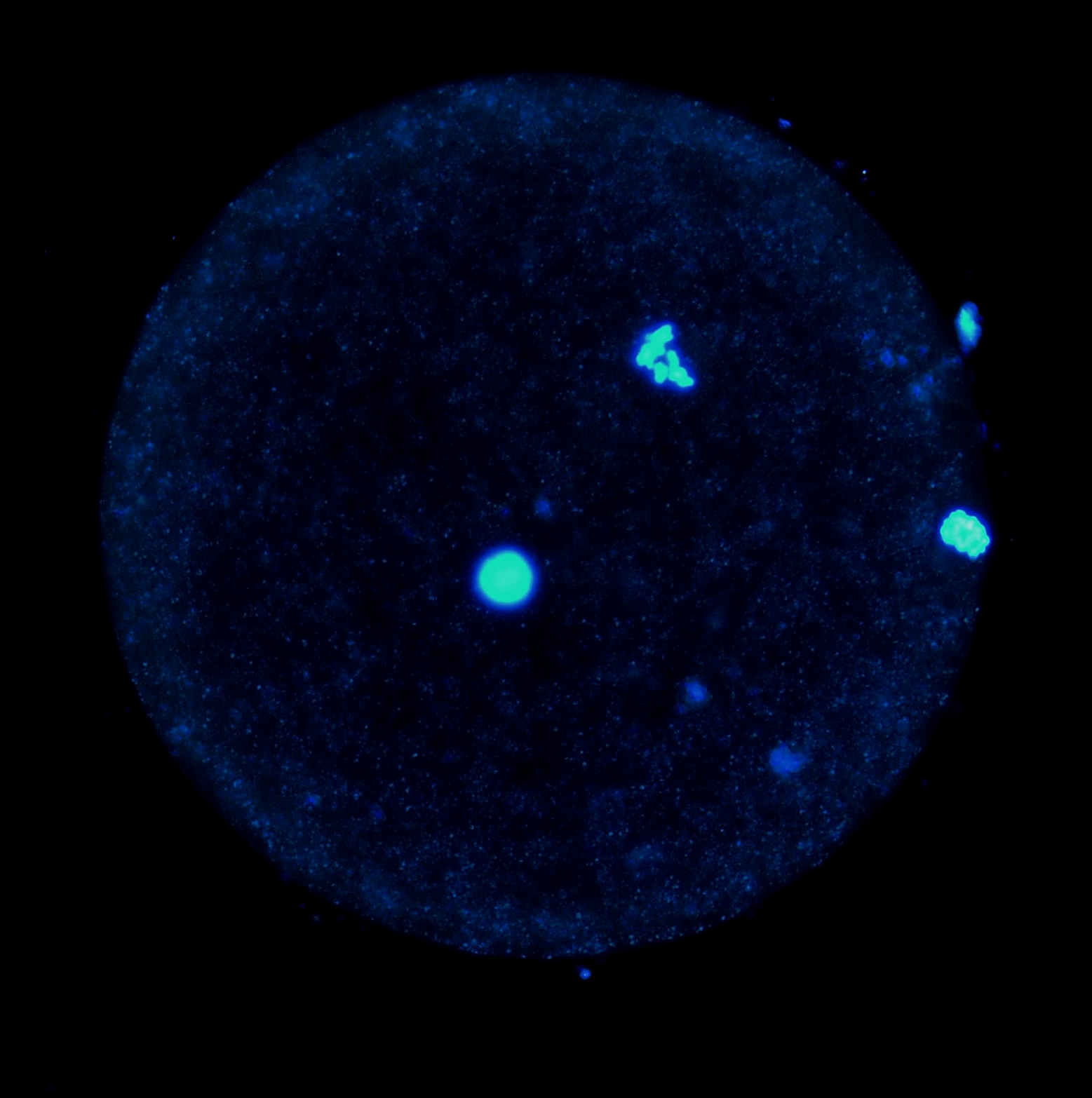

Supplement: Supplementary file 4 [file DataSheet6.ZIP › Figure6/Ace/C (1).tif]

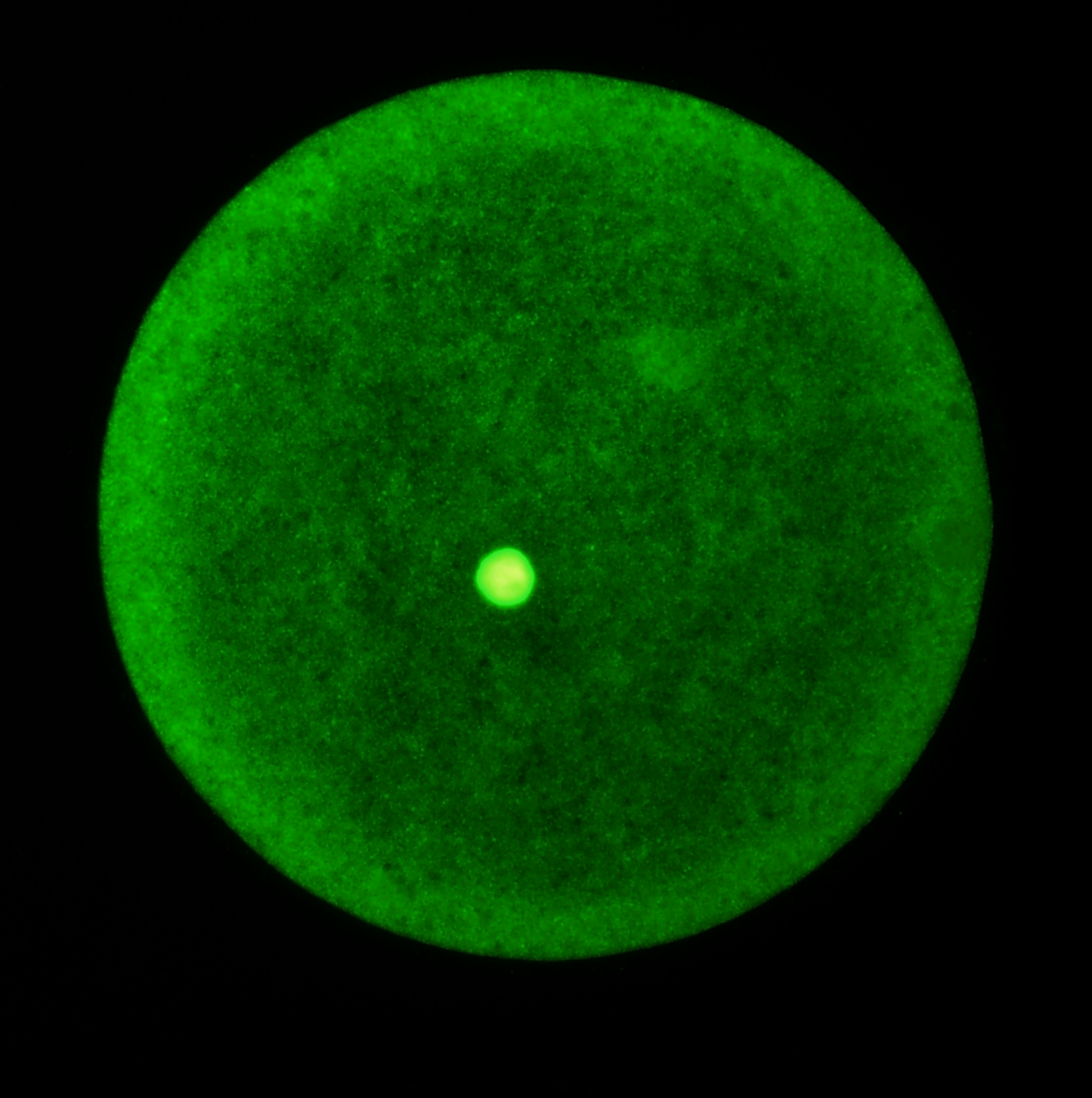

Supplement: Supplementary file 4 [file DataSheet6.ZIP › Figure6/Ace/C (2).tif]

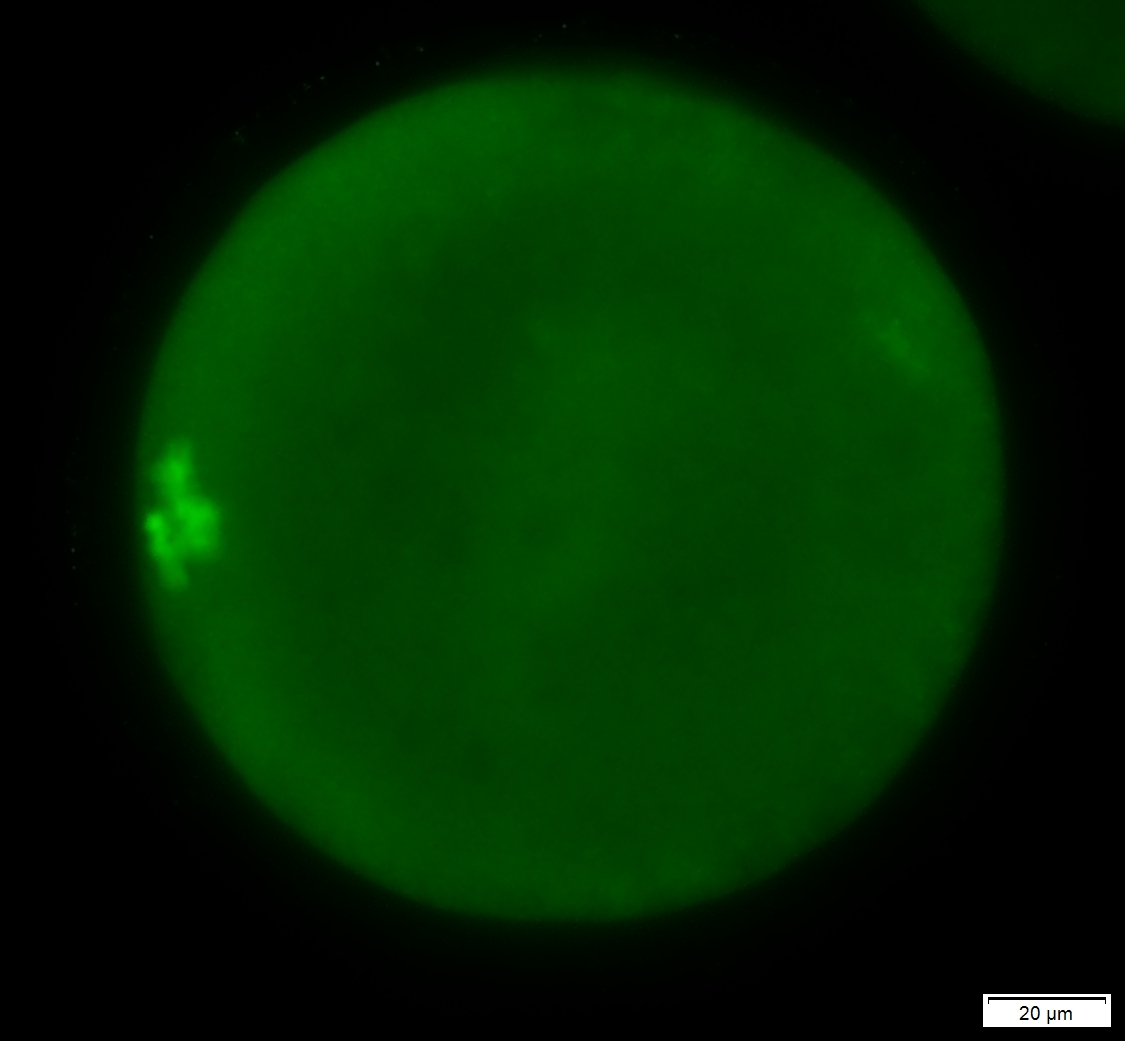

Supplement: Supplementary file 4 [file DataSheet6.ZIP › Figure6/Ace/CoA (1).jpg]

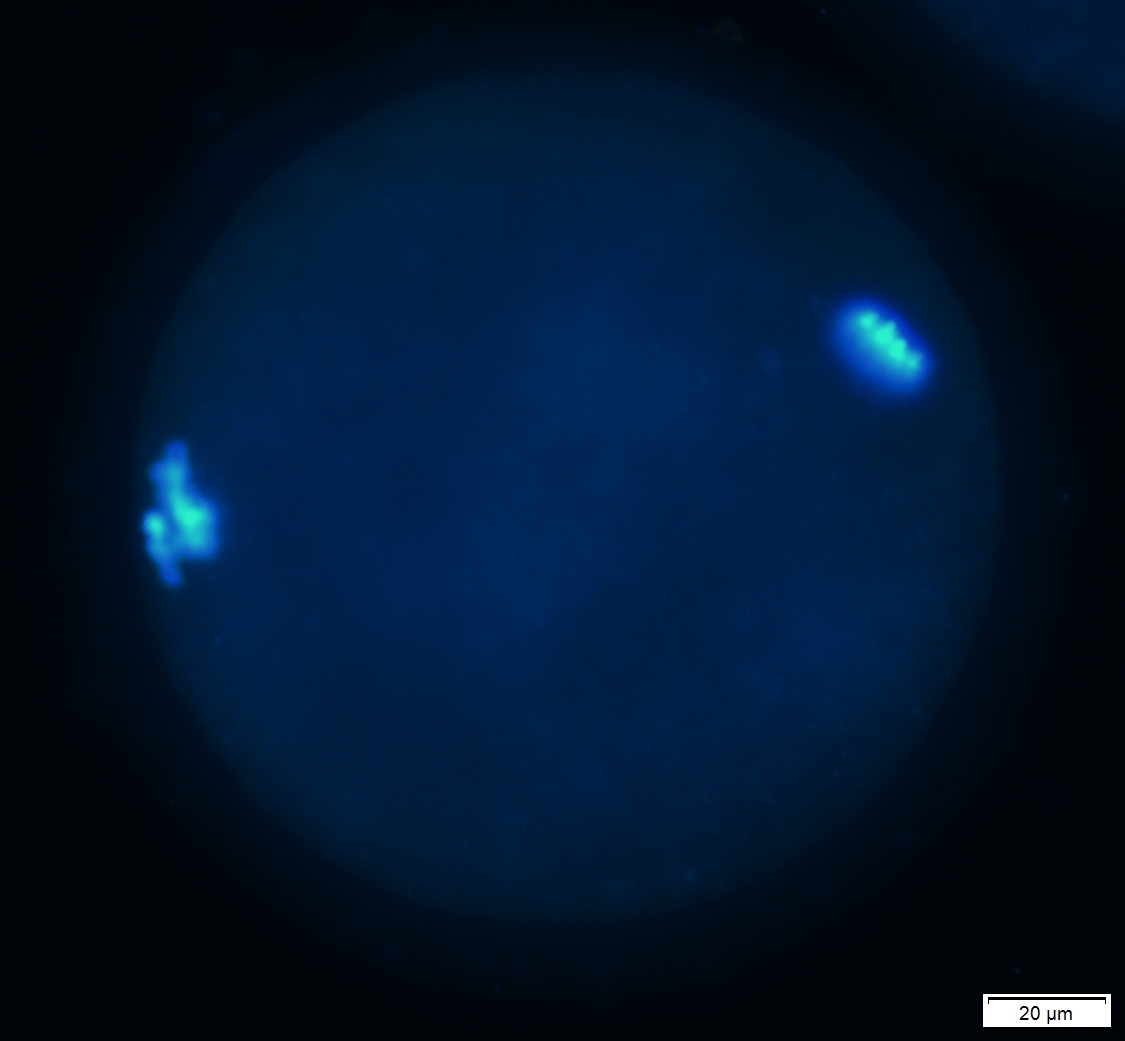

Supplement: Supplementary file 4 [file DataSheet6.ZIP › Figure6/Ace/CoA (2).jpg]

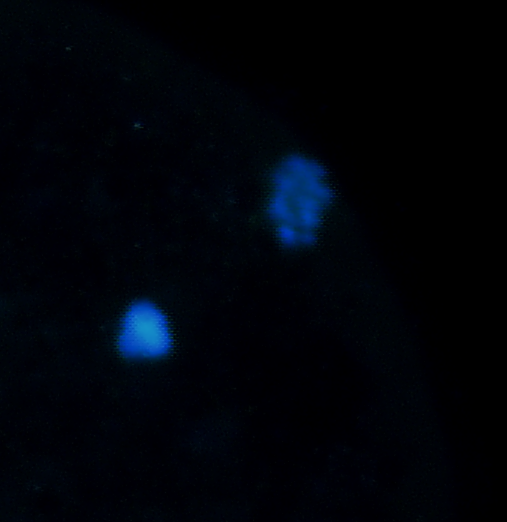

Supplement: Supplementary file 4 [file DataSheet6.ZIP › Figure6/╖─┤╕╠σ/b (1).tif]

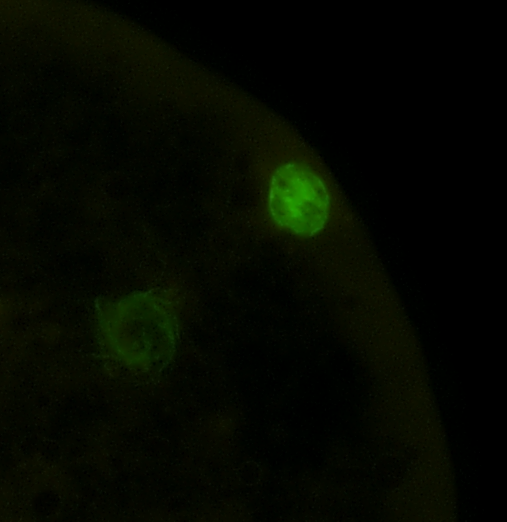

Supplement: Supplementary file 4 [file DataSheet6.ZIP › Figure6/╖─┤╕╠σ/b (2).tif]

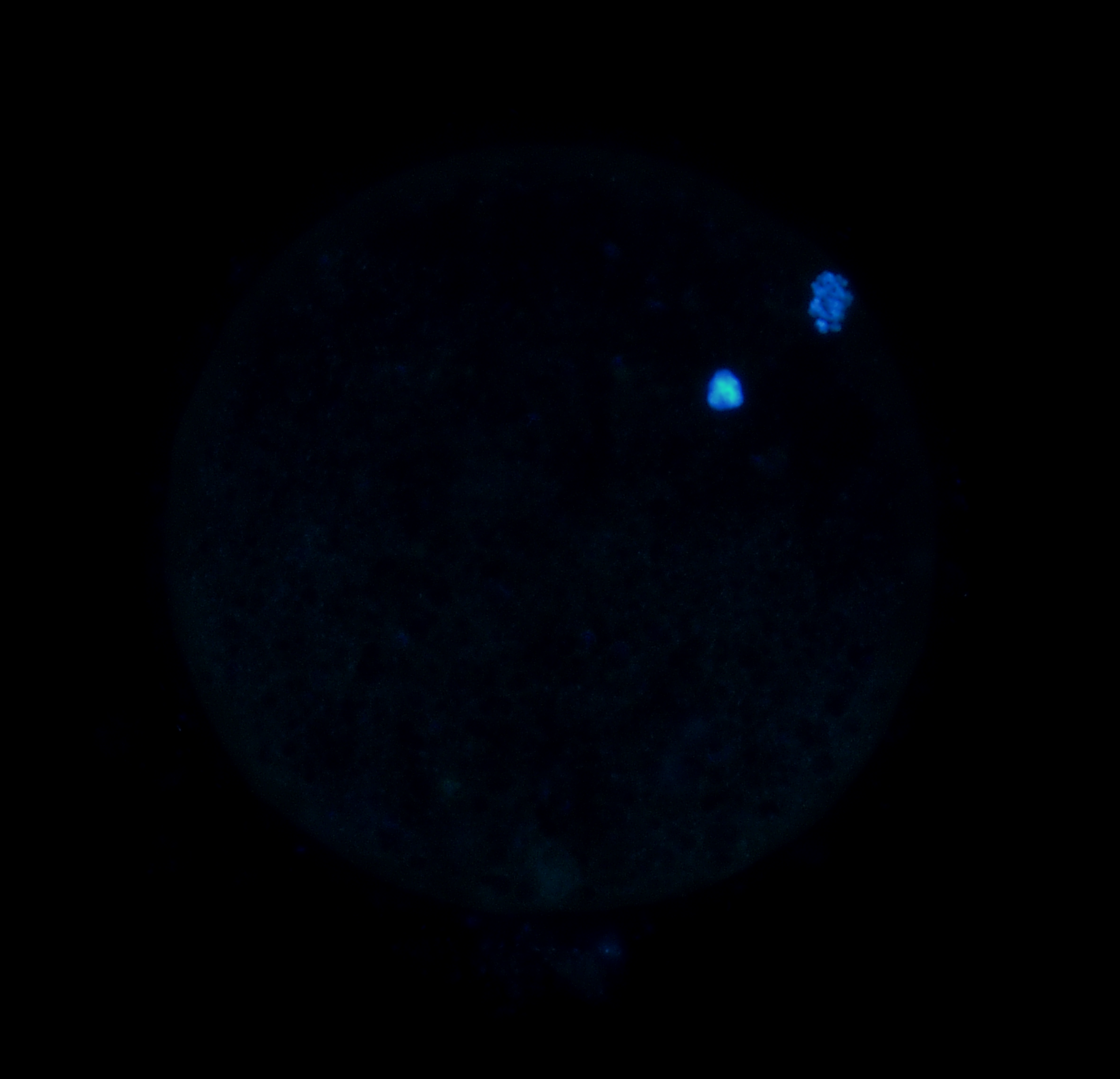

Supplement: Supplementary file 4 [file DataSheet6.ZIP › Figure6/╖─┤╕╠σ/b (3).tif]

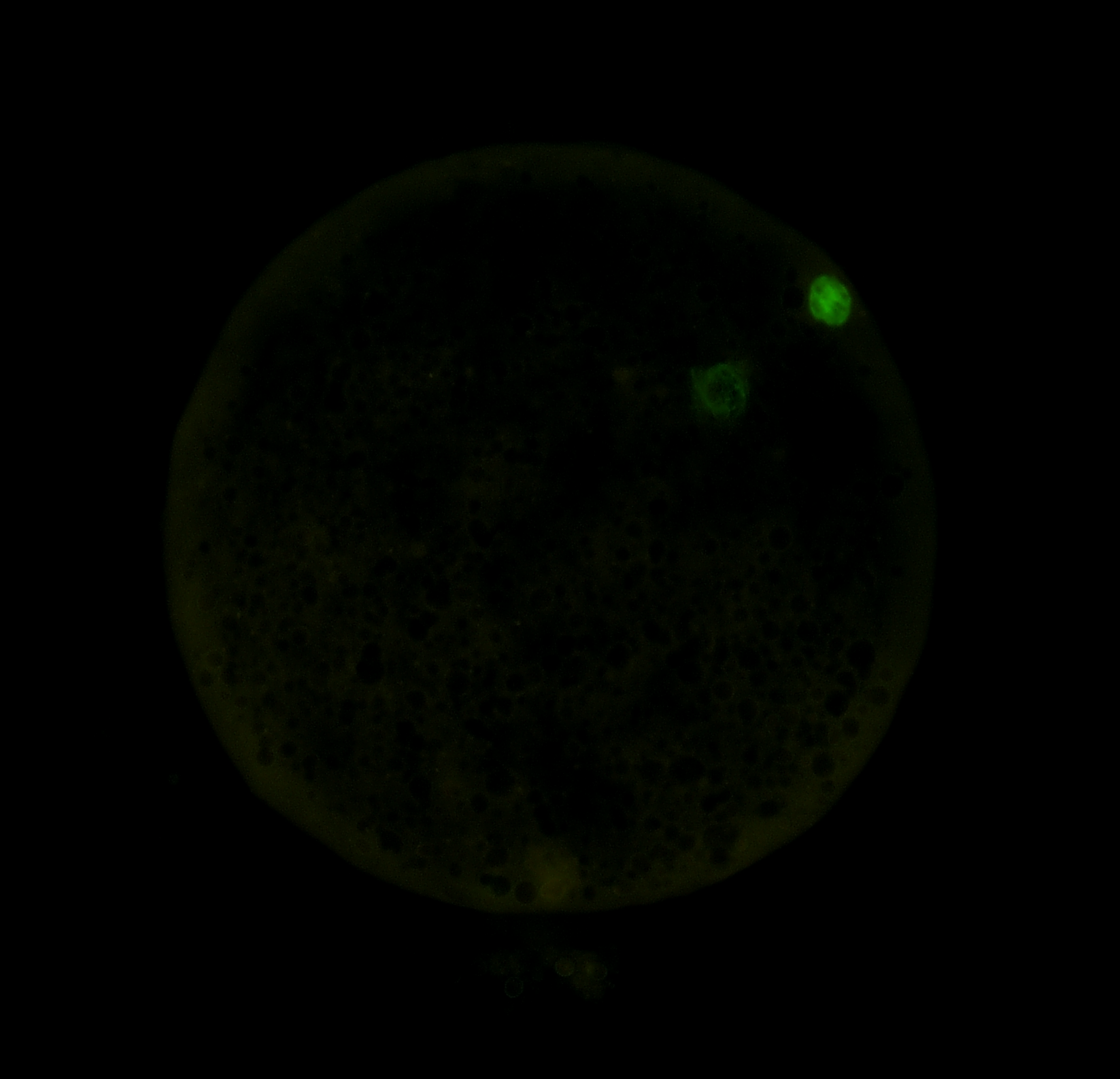

Supplement: Supplementary file 4 [file DataSheet6.ZIP › Figure6/╖─┤╕╠σ/b (4).tif]

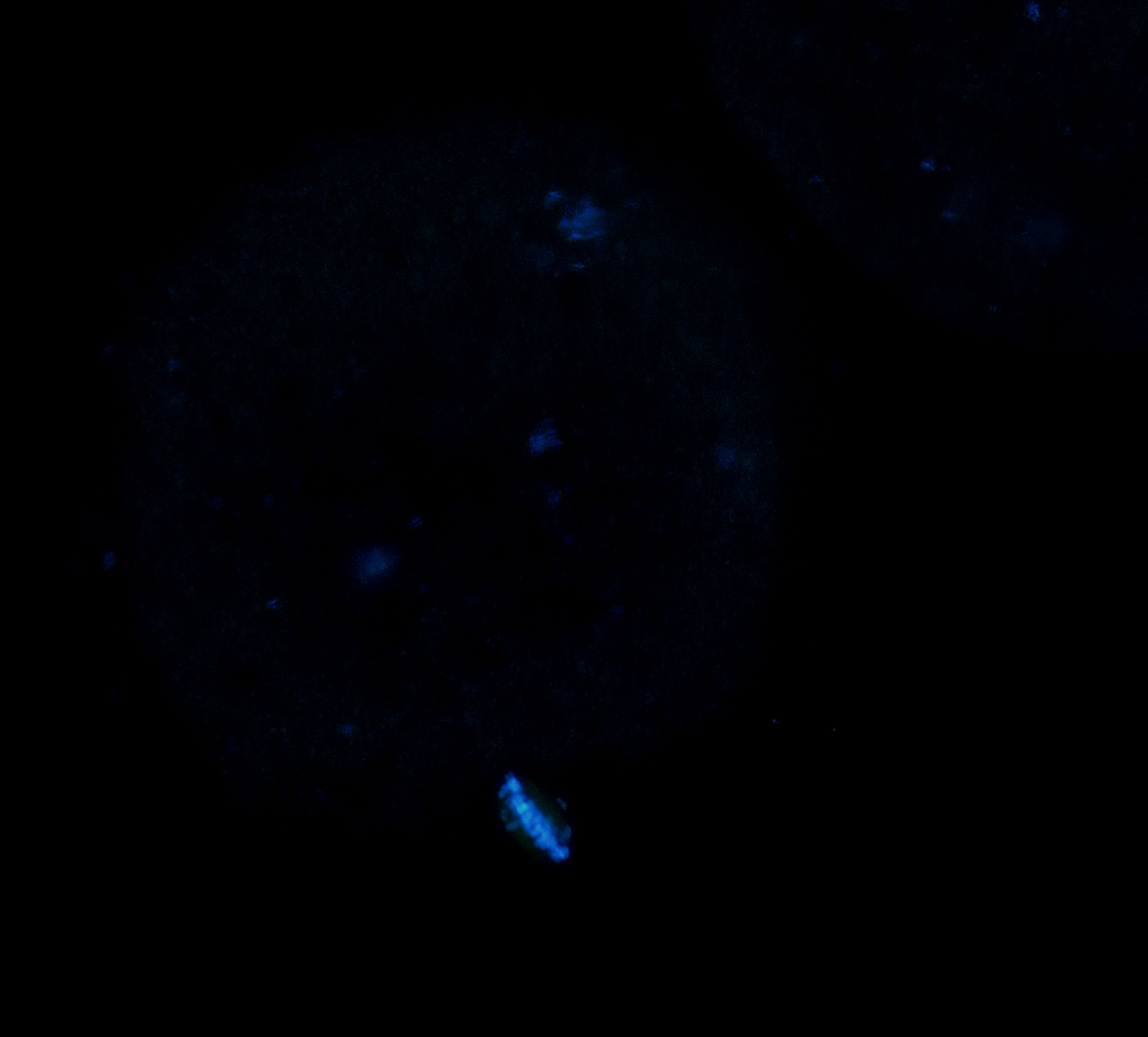

Supplement: Supplementary file 4 [file DataSheet6.ZIP › Figure6/╖─┤╕╠σ/C (1).tif]

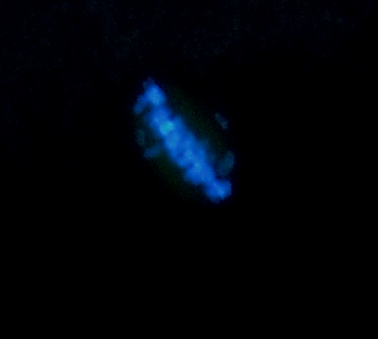

Supplement: Supplementary file 4 [file DataSheet6.ZIP › Figure6/╖─┤╕╠σ/C (2).tif]

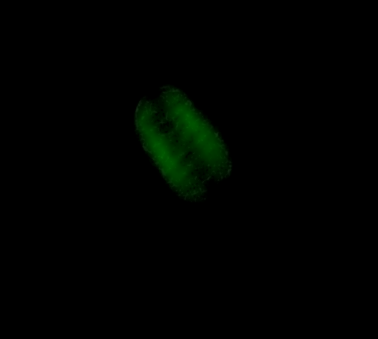

Supplement: Supplementary file 4 [file DataSheet6.ZIP › Figure6/╖─┤╕╠σ/C (3).tif]

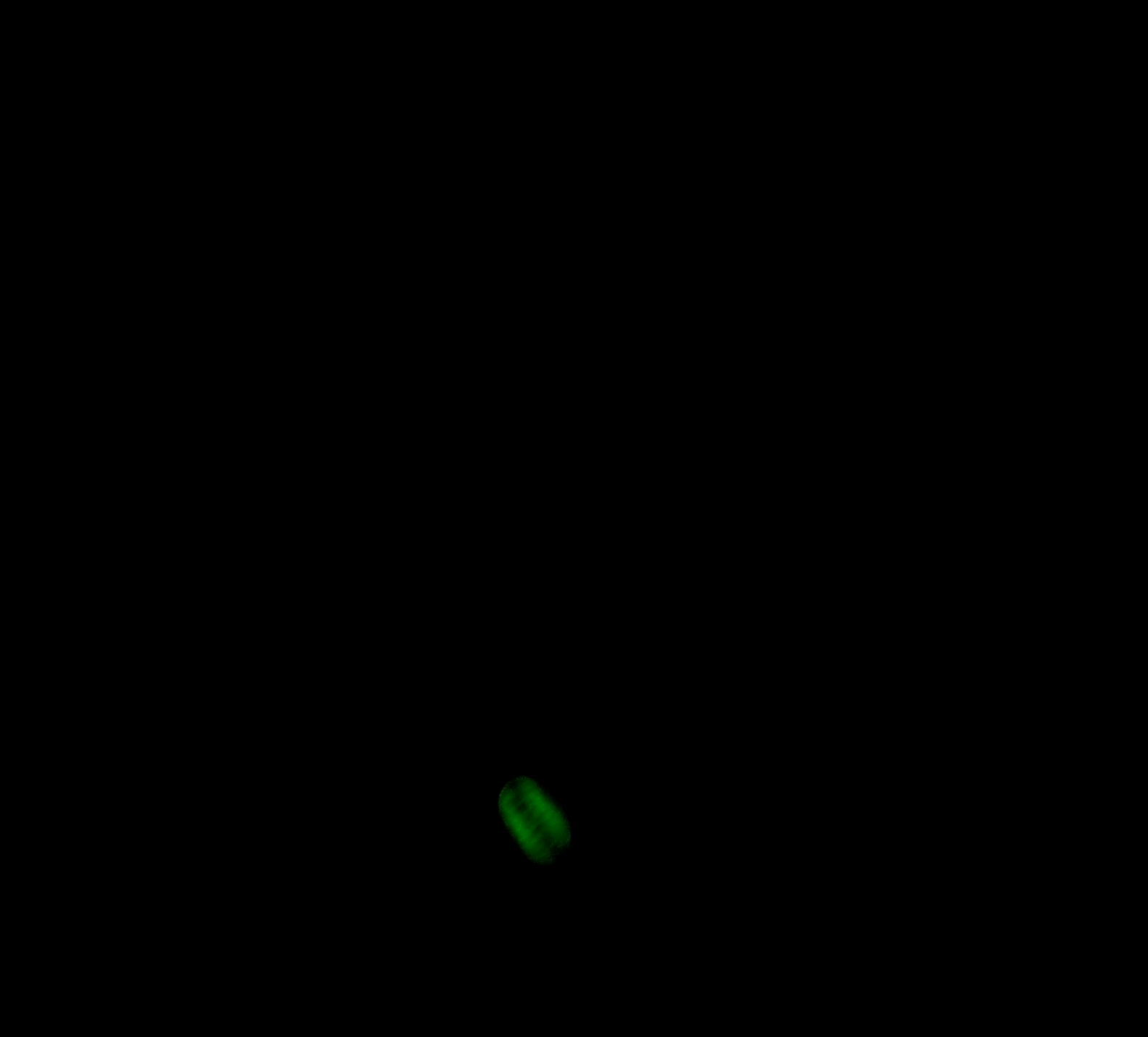

Supplement: Supplementary file 4 [file DataSheet6.ZIP › Figure6/╖─┤╕╠σ/C (4).tif]

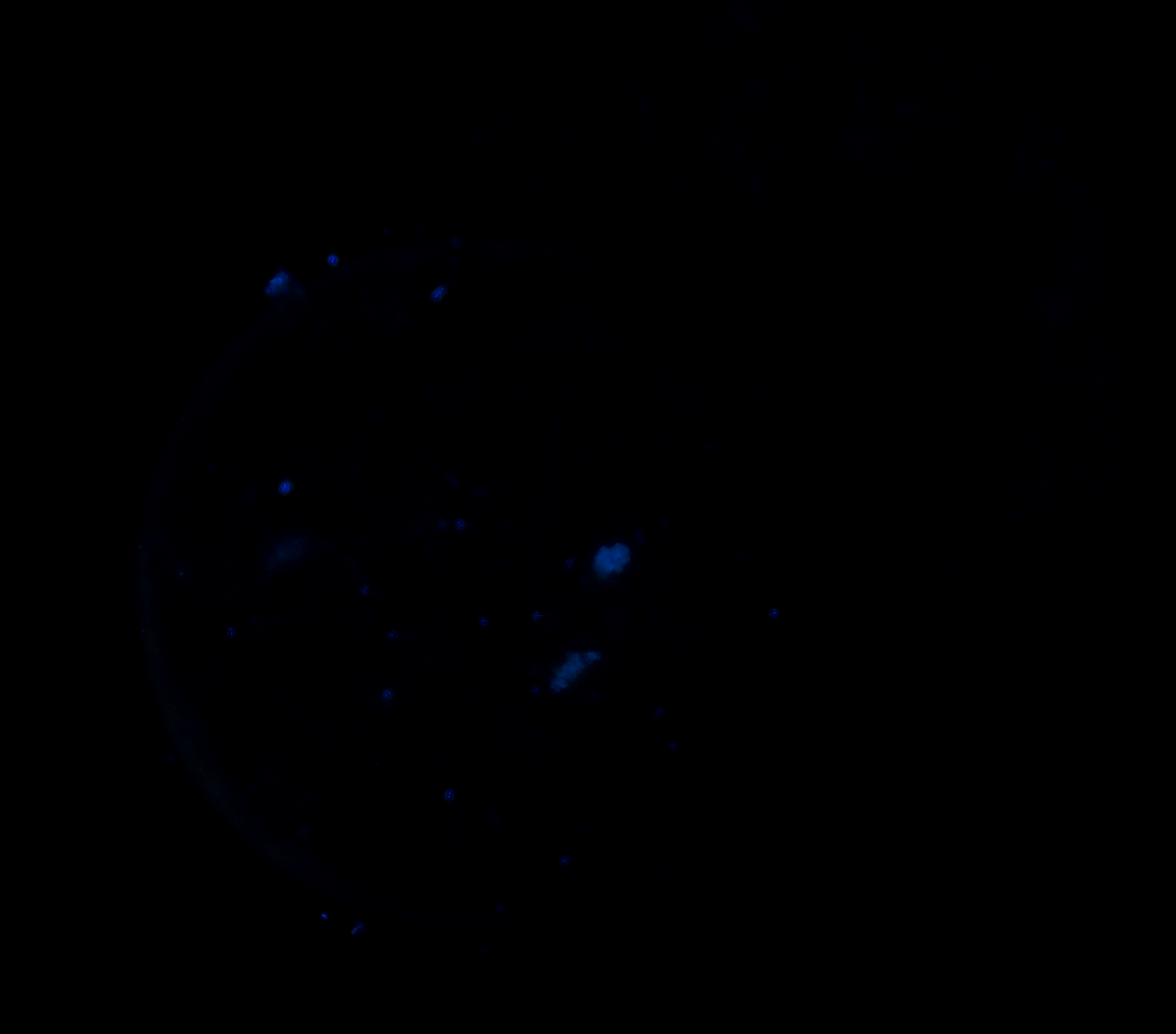

Supplement: Supplementary file 4 [file DataSheet6.ZIP › Figure6/╖─┤╕╠σ/CoA (1).tif]

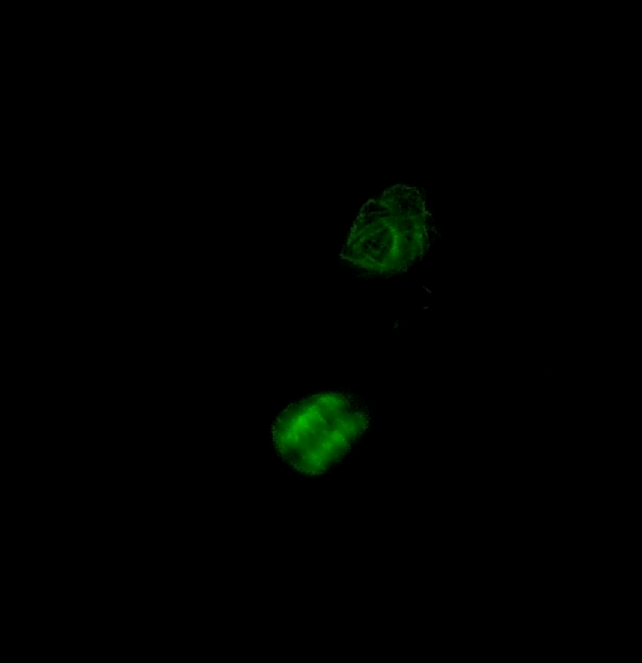

Supplement: Supplementary file 4 [file DataSheet6.ZIP › Figure6/╖─┤╕╠σ/CoA (2).tif]

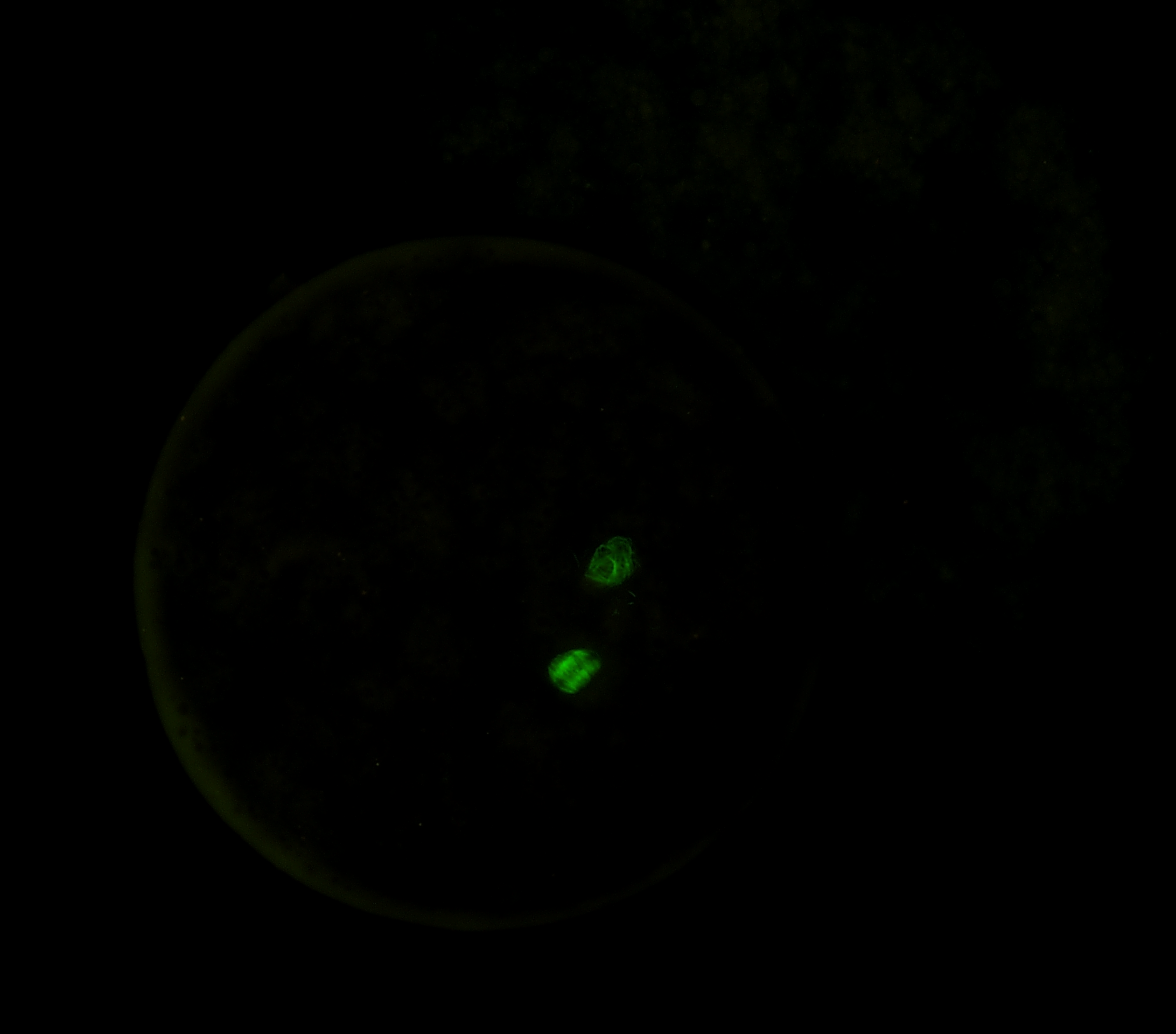

Supplement: Supplementary file 4 [file DataSheet6.ZIP › Figure6/╖─┤╕╠σ/CoA (3).tif]

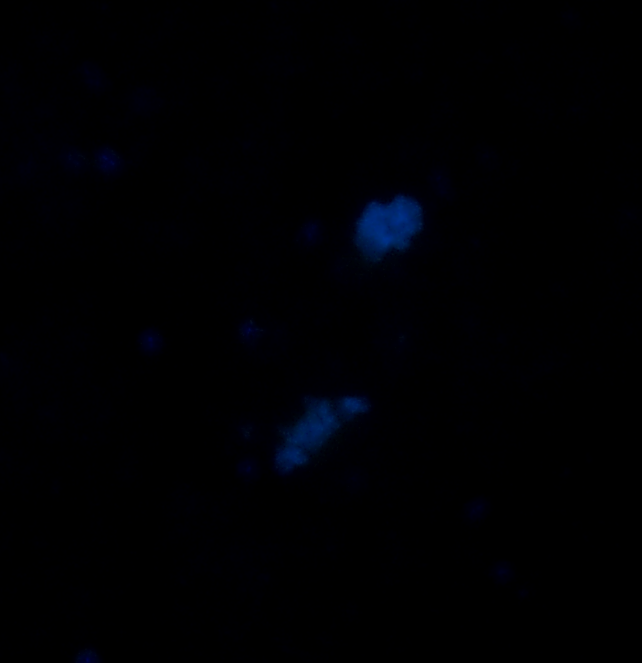

Supplement: Supplementary file 4 [file DataSheet6.ZIP › Figure6/╖─┤╕╠σ/CoA.tif]

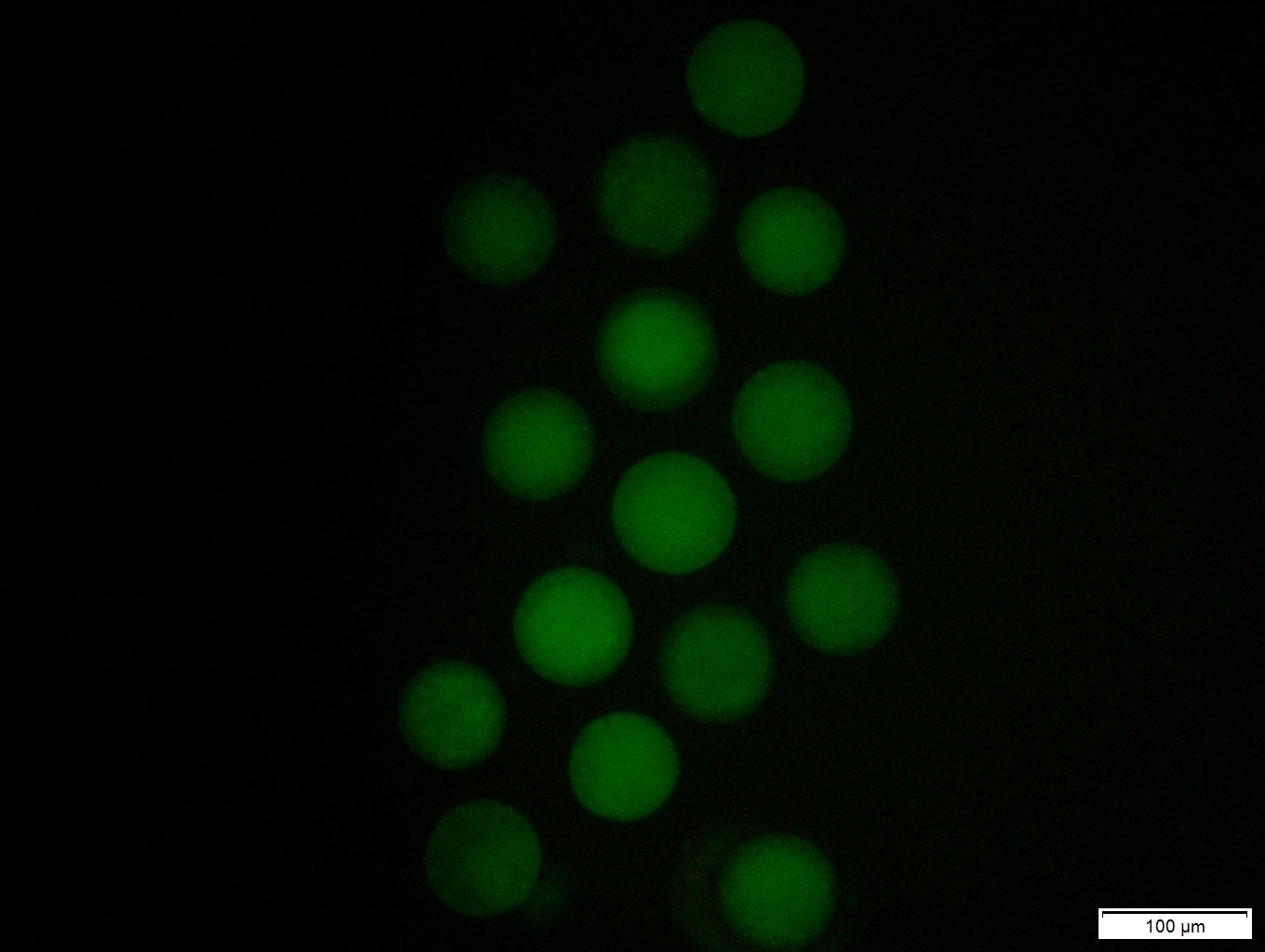

Supplement: Supplementary file 5 [file DataSheet2.ZIP › Figure2í╠/ATP/1.2-ATP.jpg]

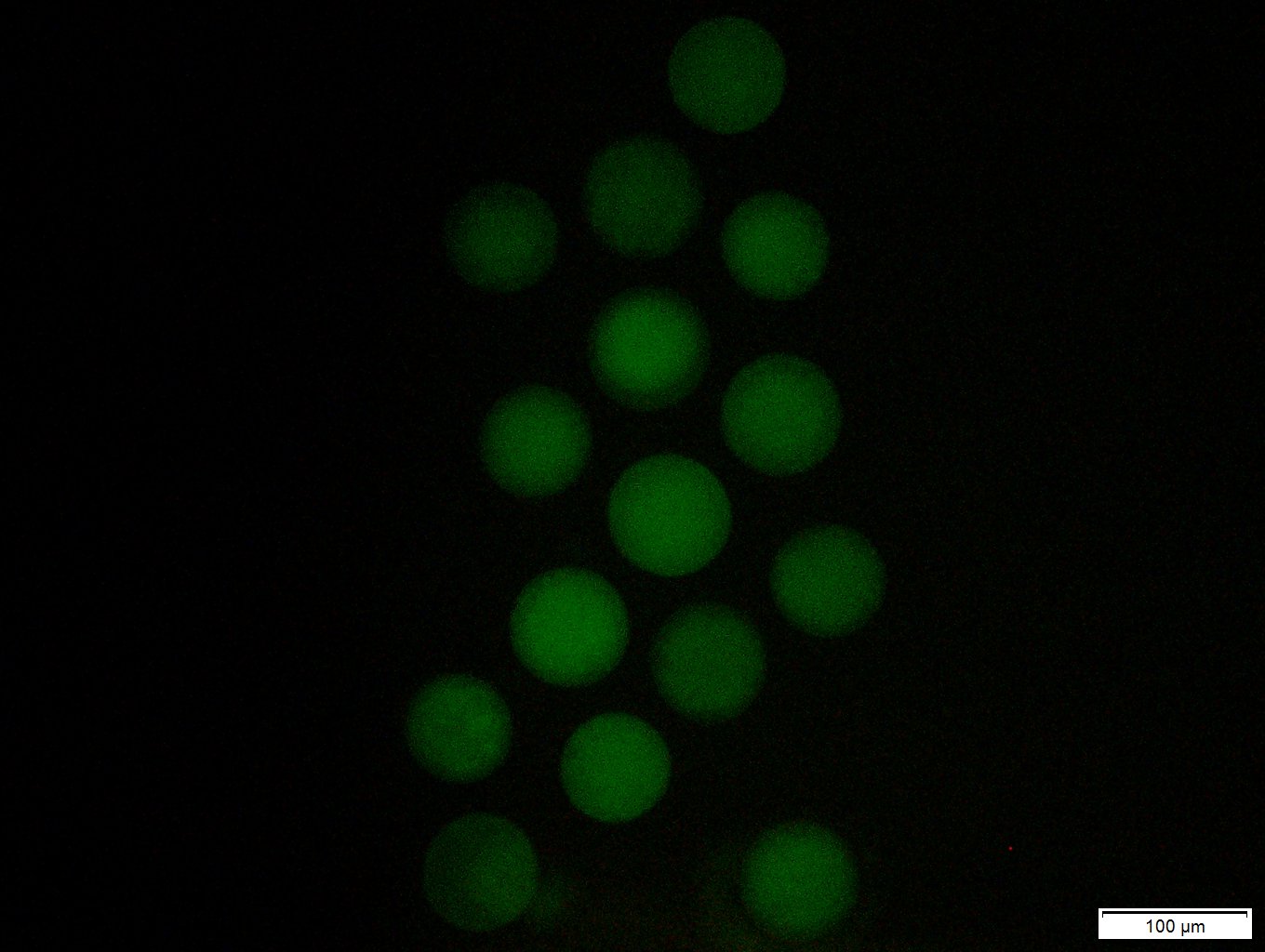

Supplement: Supplementary file 5 [file DataSheet2.ZIP › Figure2í╠/ATP/3.6-ATP.jpg]

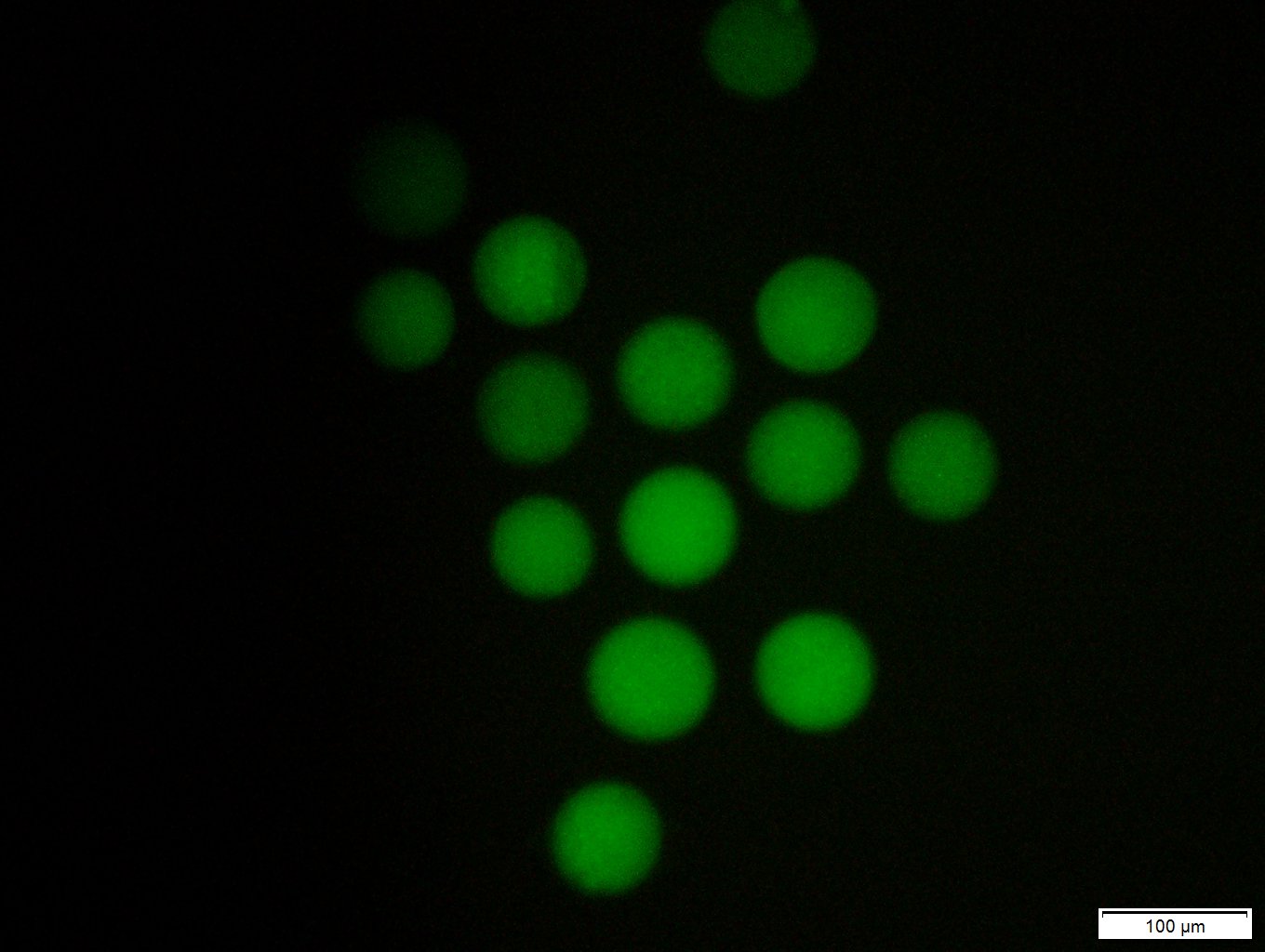

Supplement: Supplementary file 5 [file DataSheet2.ZIP › Figure2í╠/ATP/C-ATP.jpg]

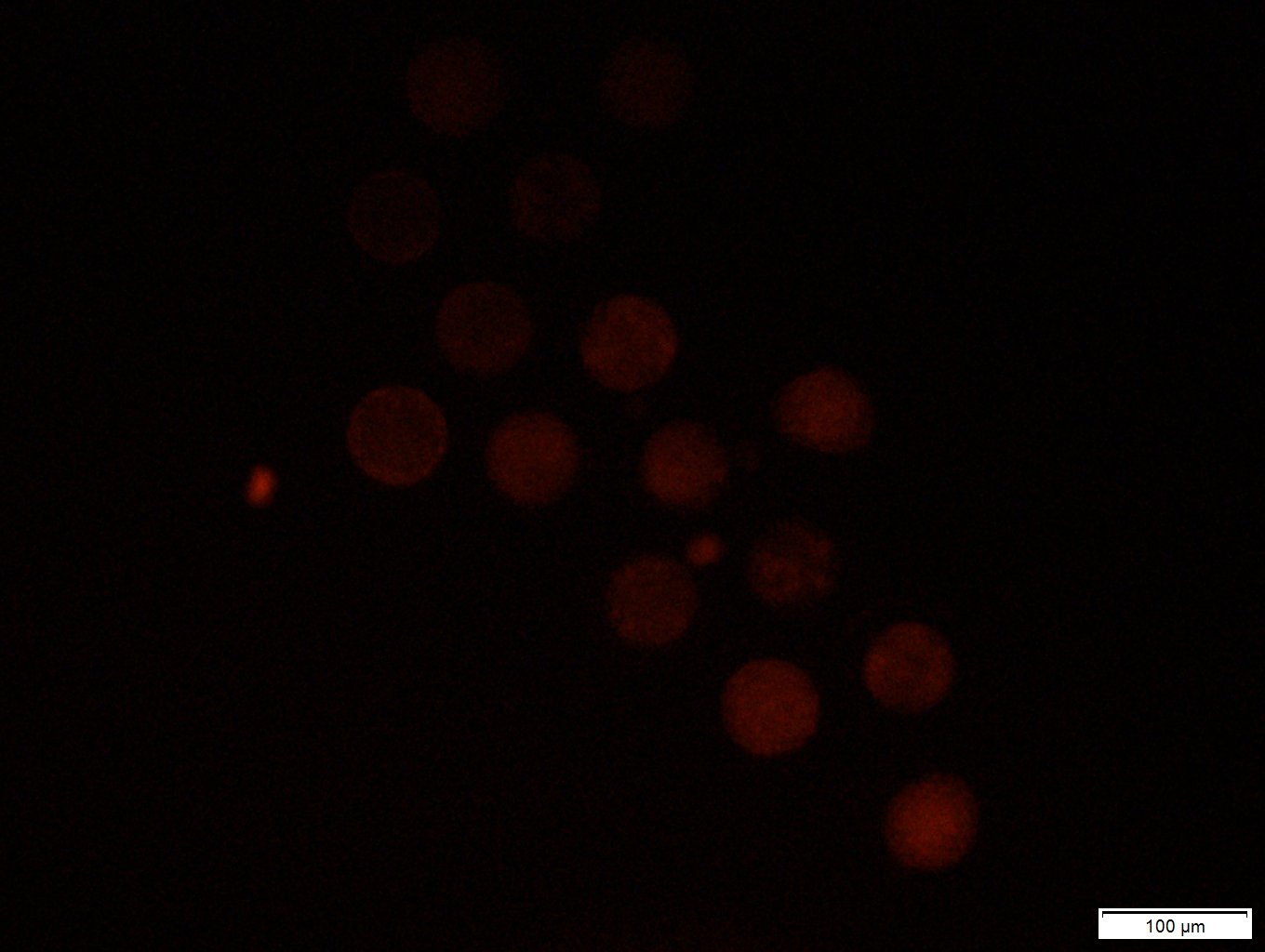

Supplement: Supplementary file 5 [file DataSheet2.ZIP › Figure2í╠/DHE/1.2-DHE.jpg]

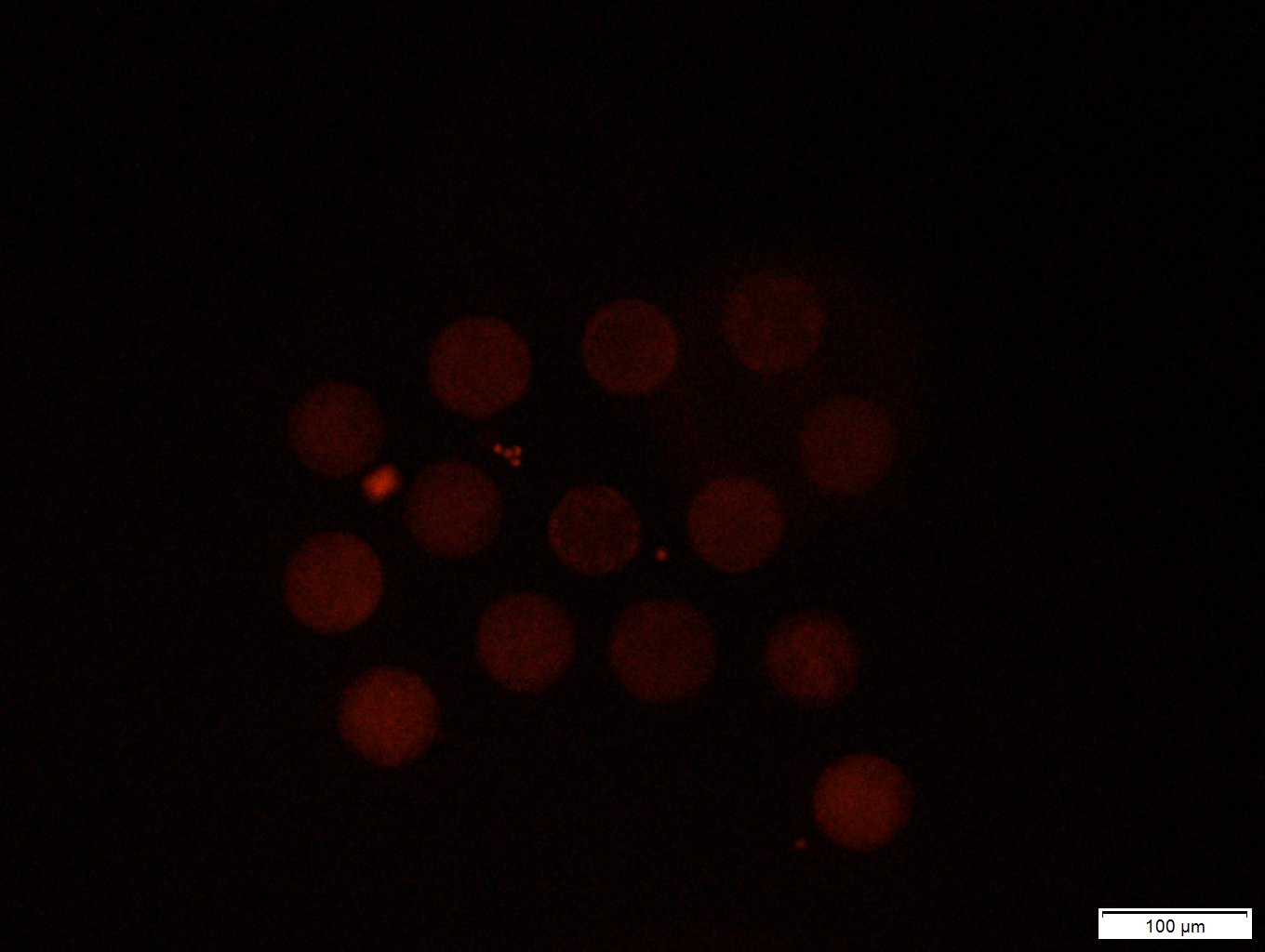

Supplement: Supplementary file 5 [file DataSheet2.ZIP › Figure2í╠/DHE/3.6-DHE.jpg]

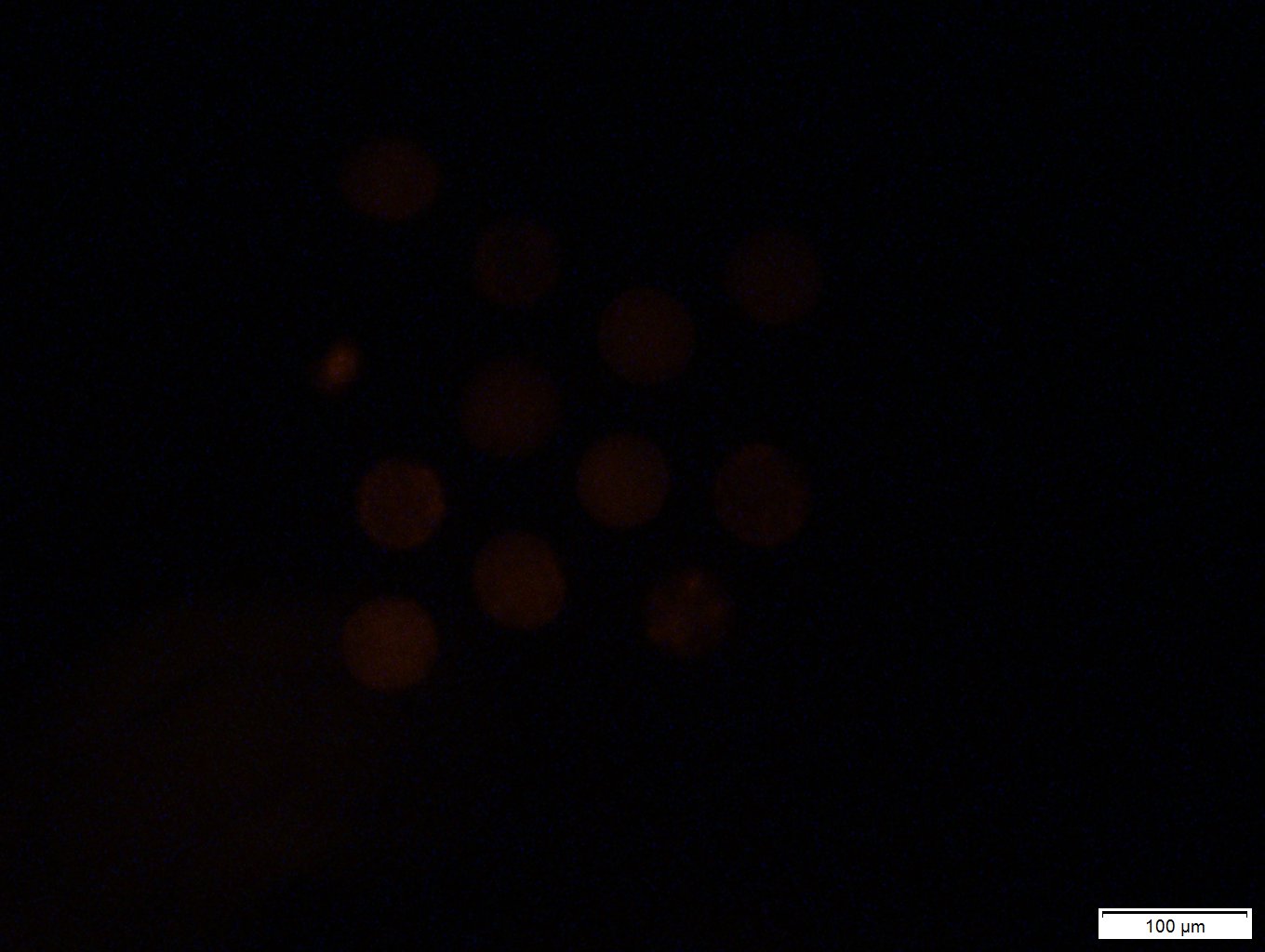

Supplement: Supplementary file 5 [file DataSheet2.ZIP › Figure2í╠/DHE/C-DHE.jpg]

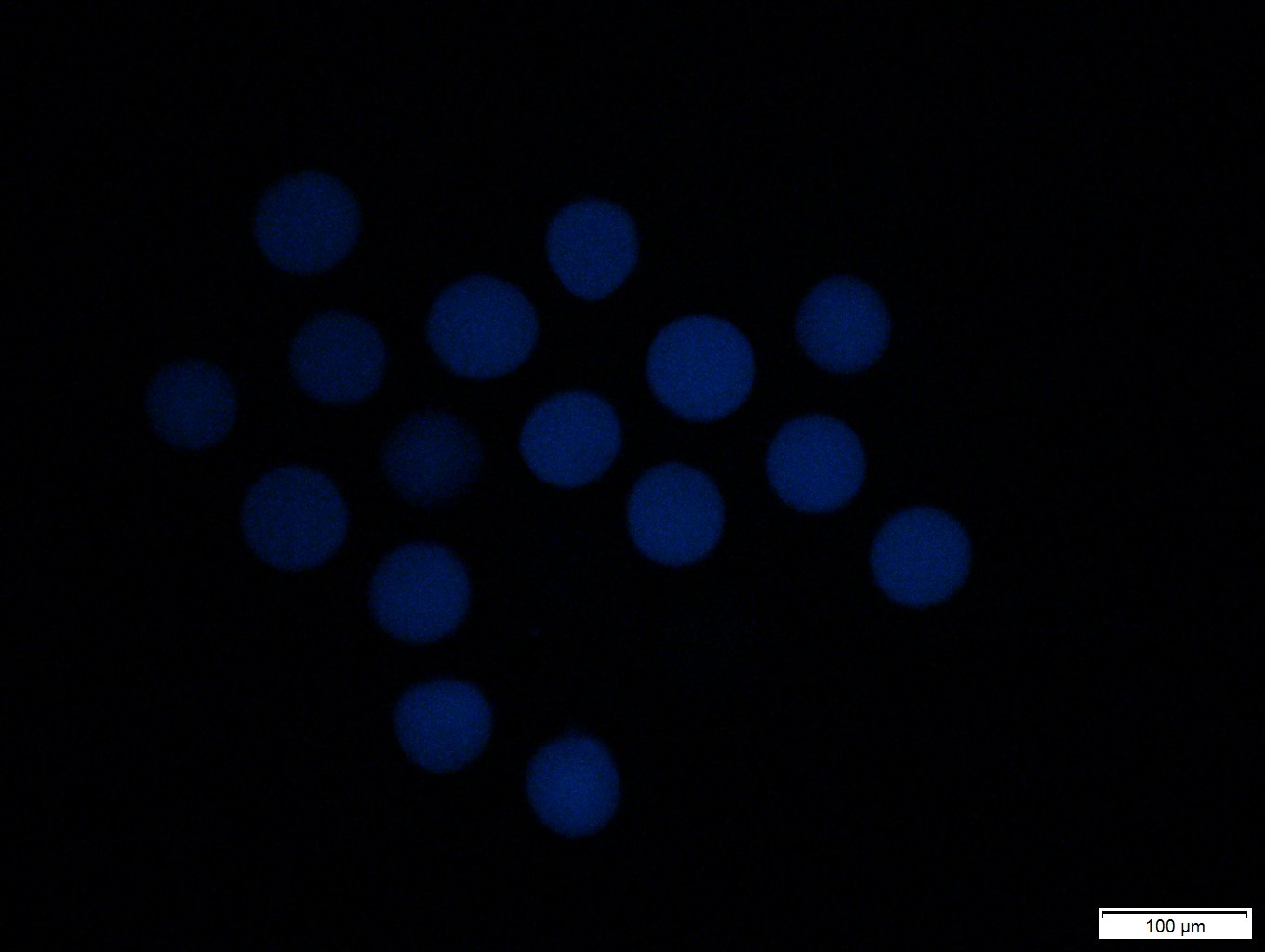

Supplement: Supplementary file 5 [file DataSheet2.ZIP › Figure2í╠/GSH/1.2-GSH.jpg]

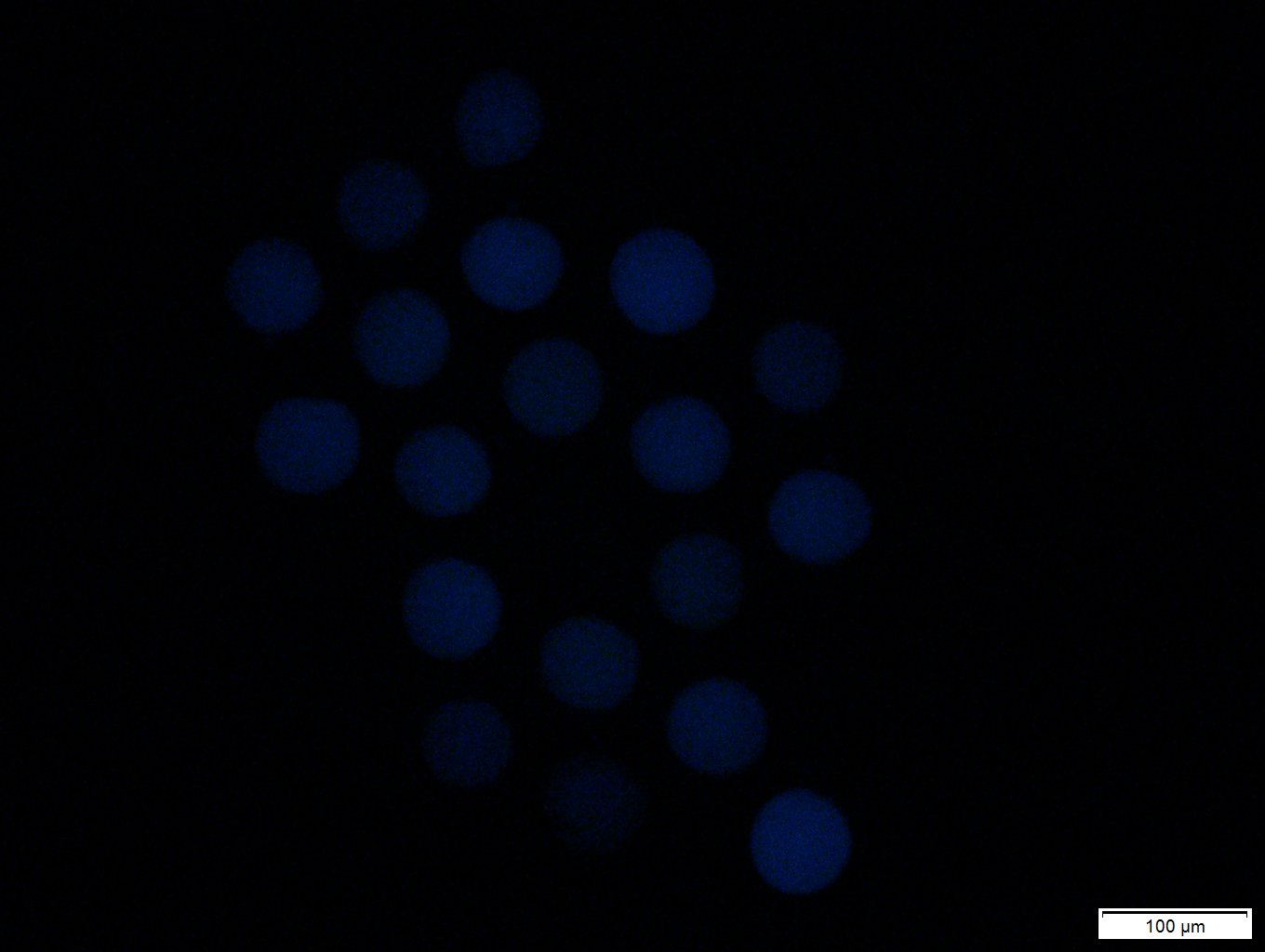

Supplement: Supplementary file 5 [file DataSheet2.ZIP › Figure2í╠/GSH/3.6-GSH.jpg]

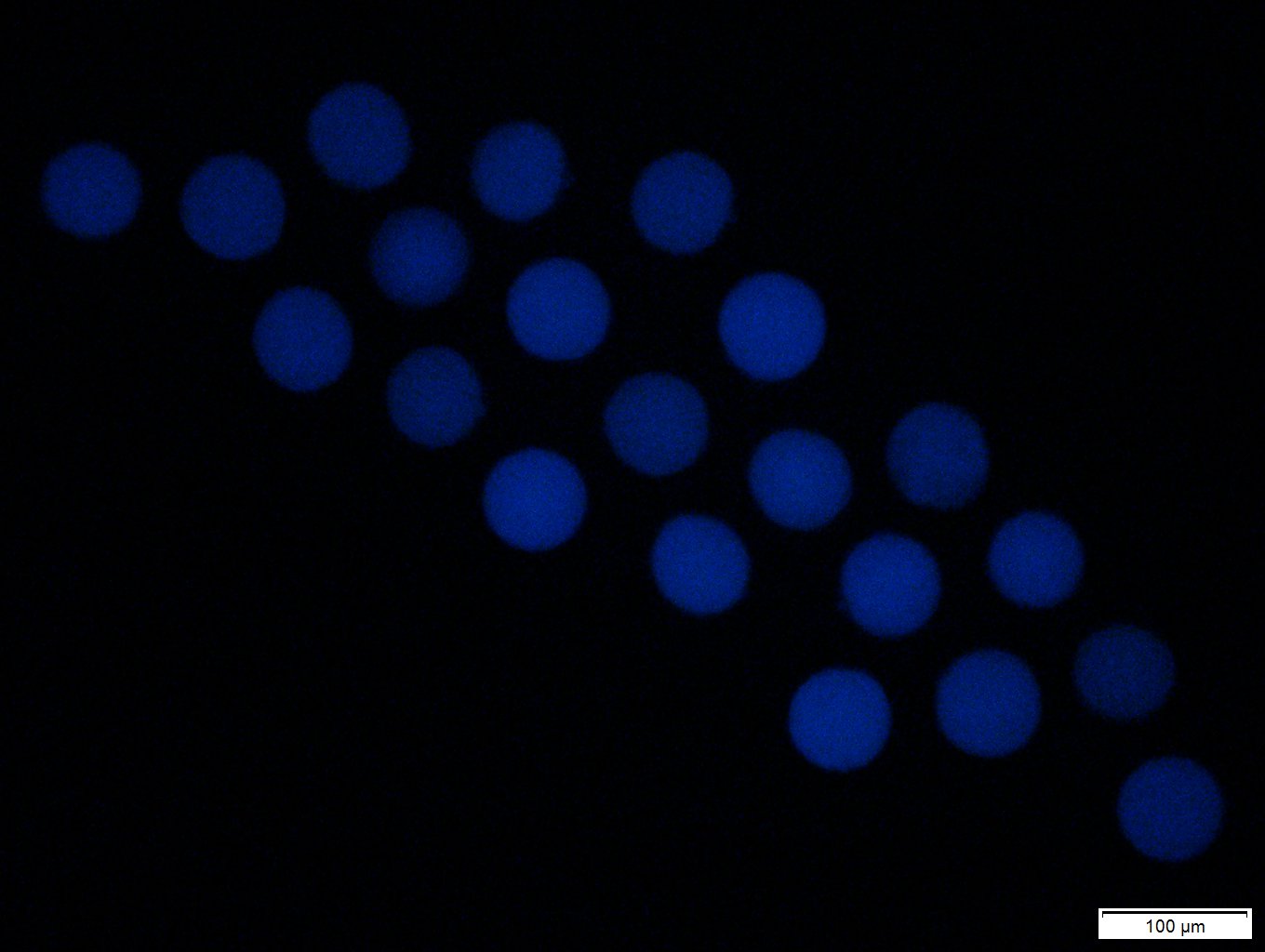

Supplement: Supplementary file 5 [file DataSheet2.ZIP › Figure2í╠/GSH/C-GSH.jpg]

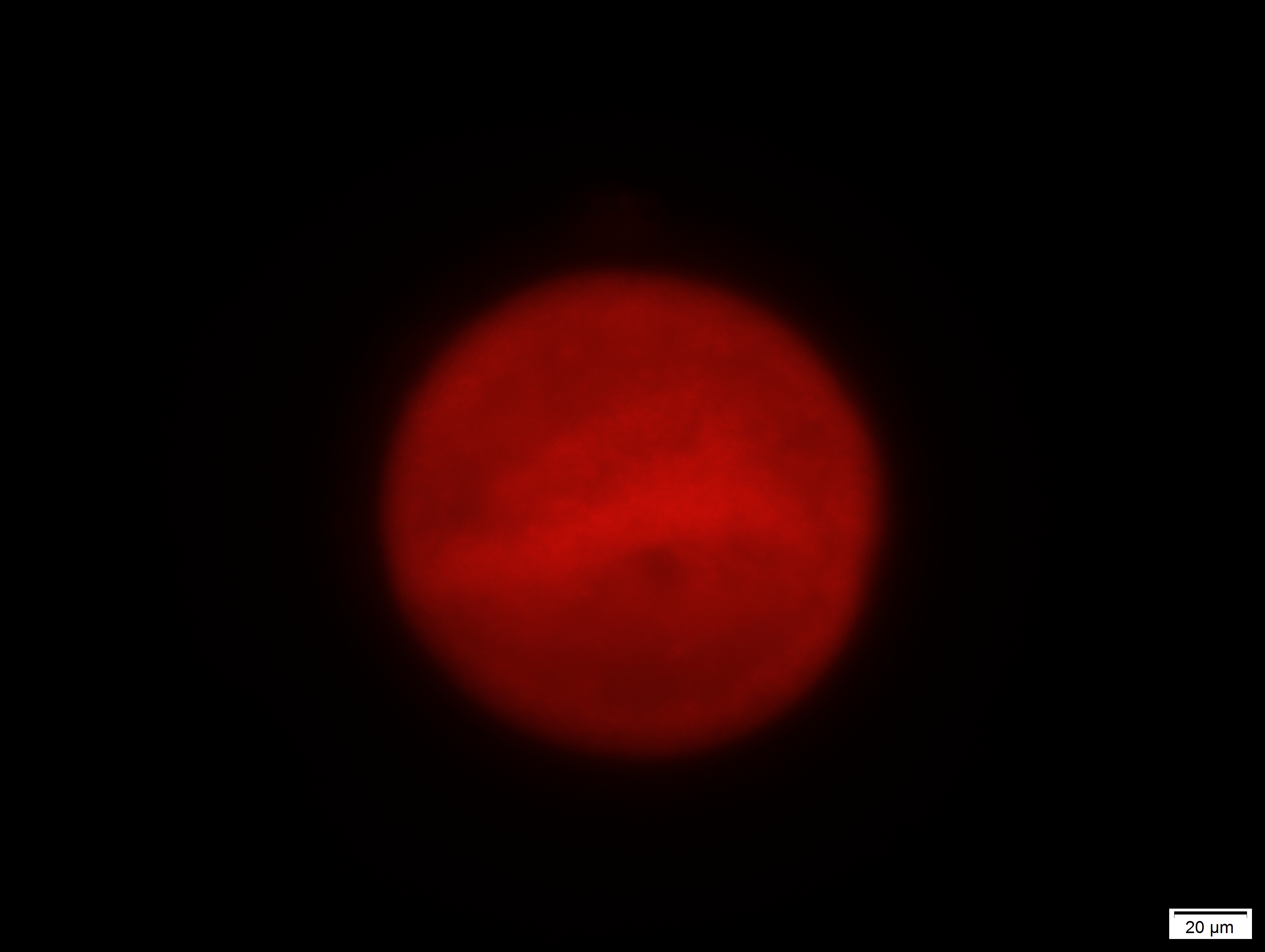

Supplement: Supplementary file 5 [file DataSheet2.ZIP › Figure2í╠/JC-1/JC-1.2mM (1).jpg]

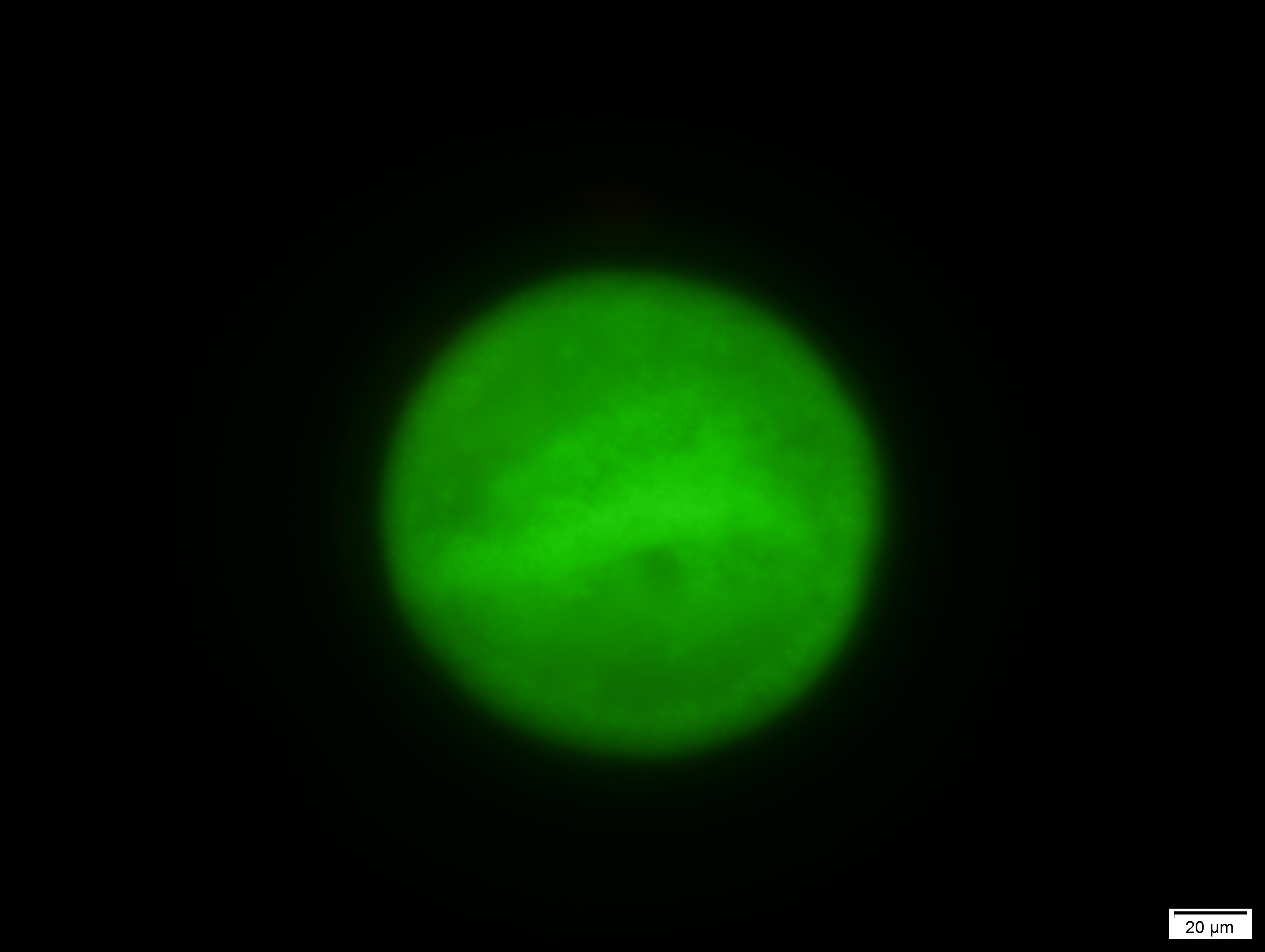

Supplement: Supplementary file 5 [file DataSheet2.ZIP › Figure2í╠/JC-1/JC-1.2mM(2).jpg]

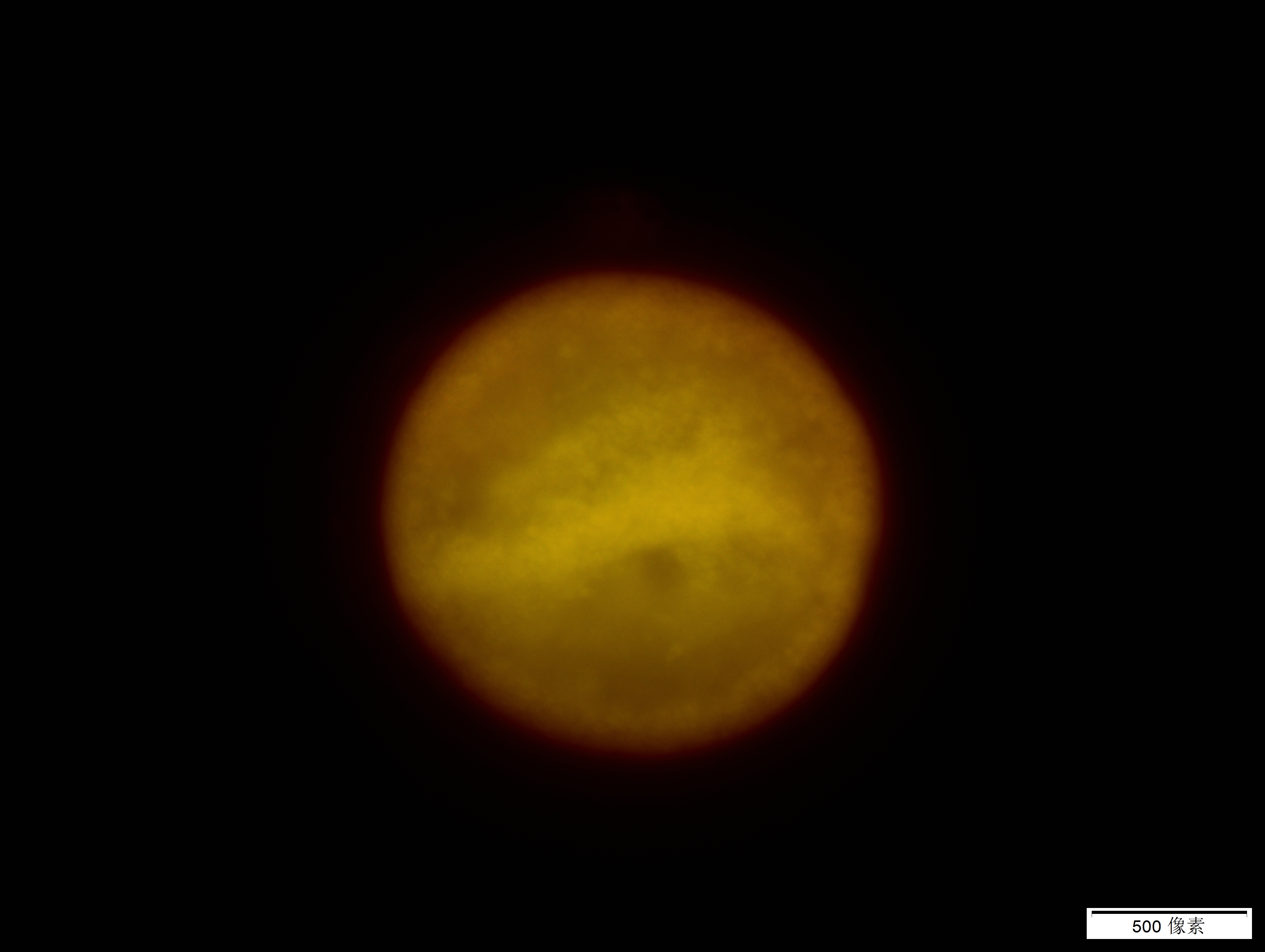

Supplement: Supplementary file 5 [file DataSheet2.ZIP › Figure2í╠/JC-1/JC-1.2mM.jpg]

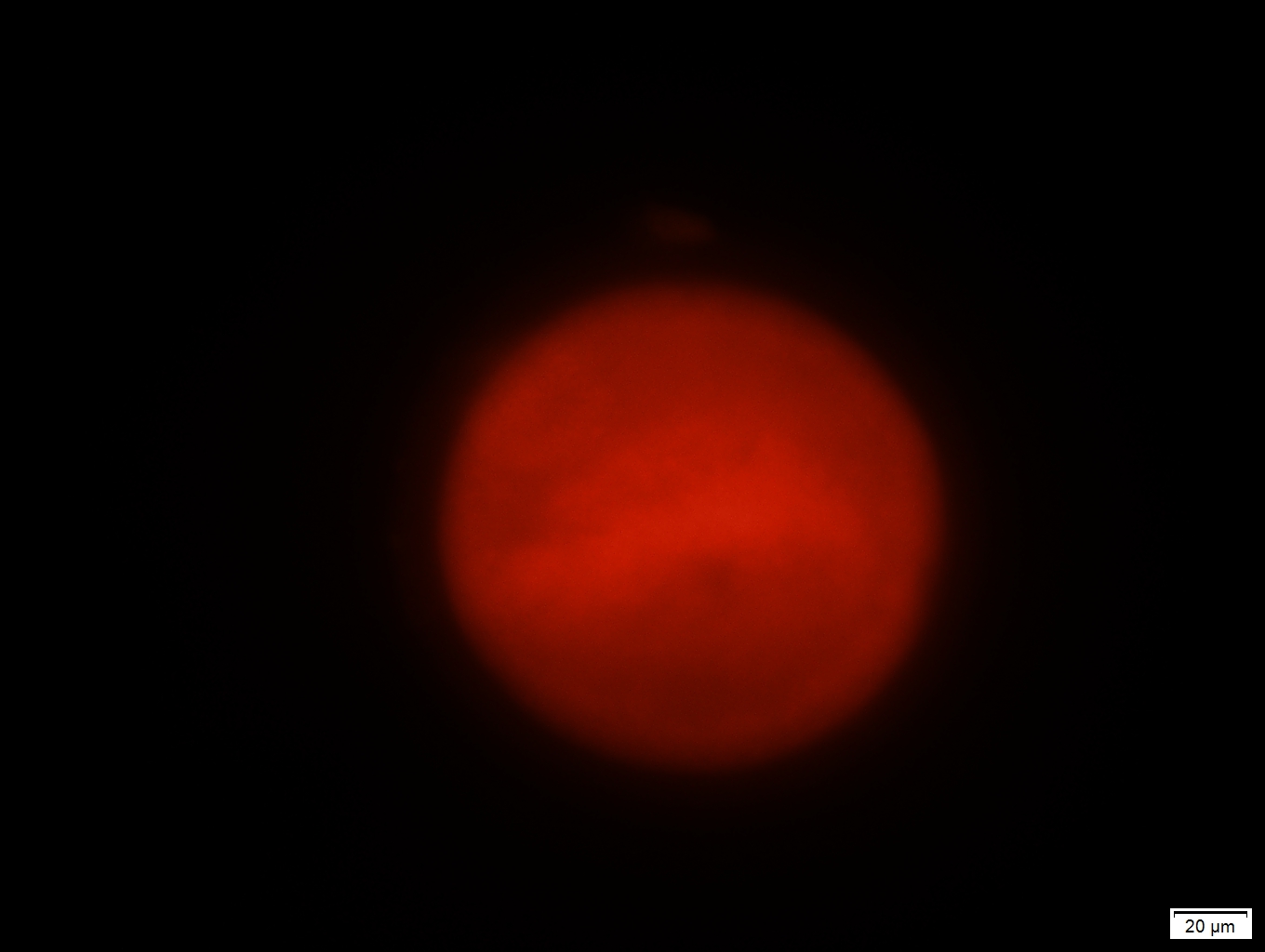

Supplement: Supplementary file 5 [file DataSheet2.ZIP › Figure2í╠/JC-1/JC-3.6mM (1).jpg]

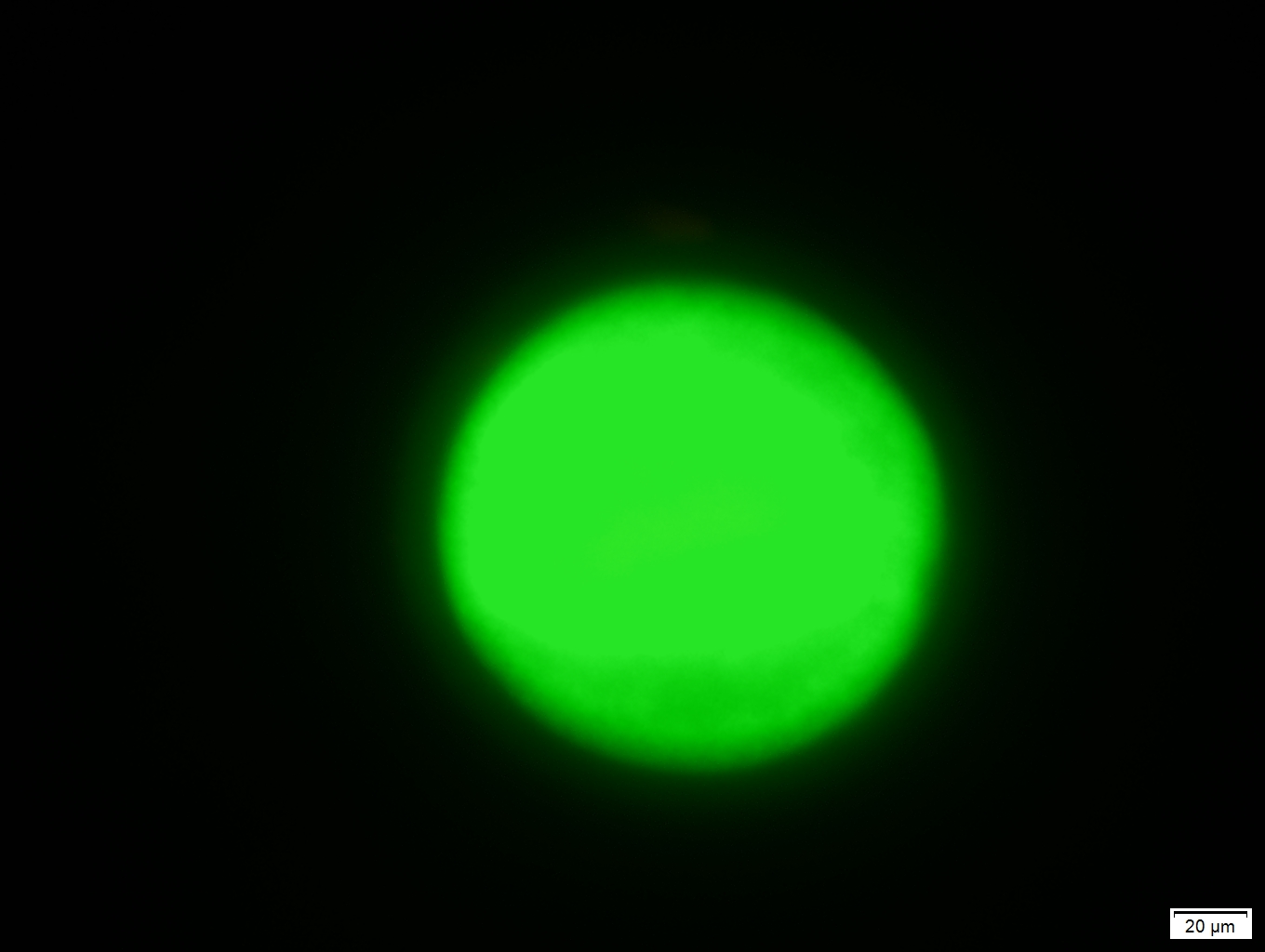

Supplement: Supplementary file 5 [file DataSheet2.ZIP › Figure2í╠/JC-1/JC-3.6mM (2).jpg]

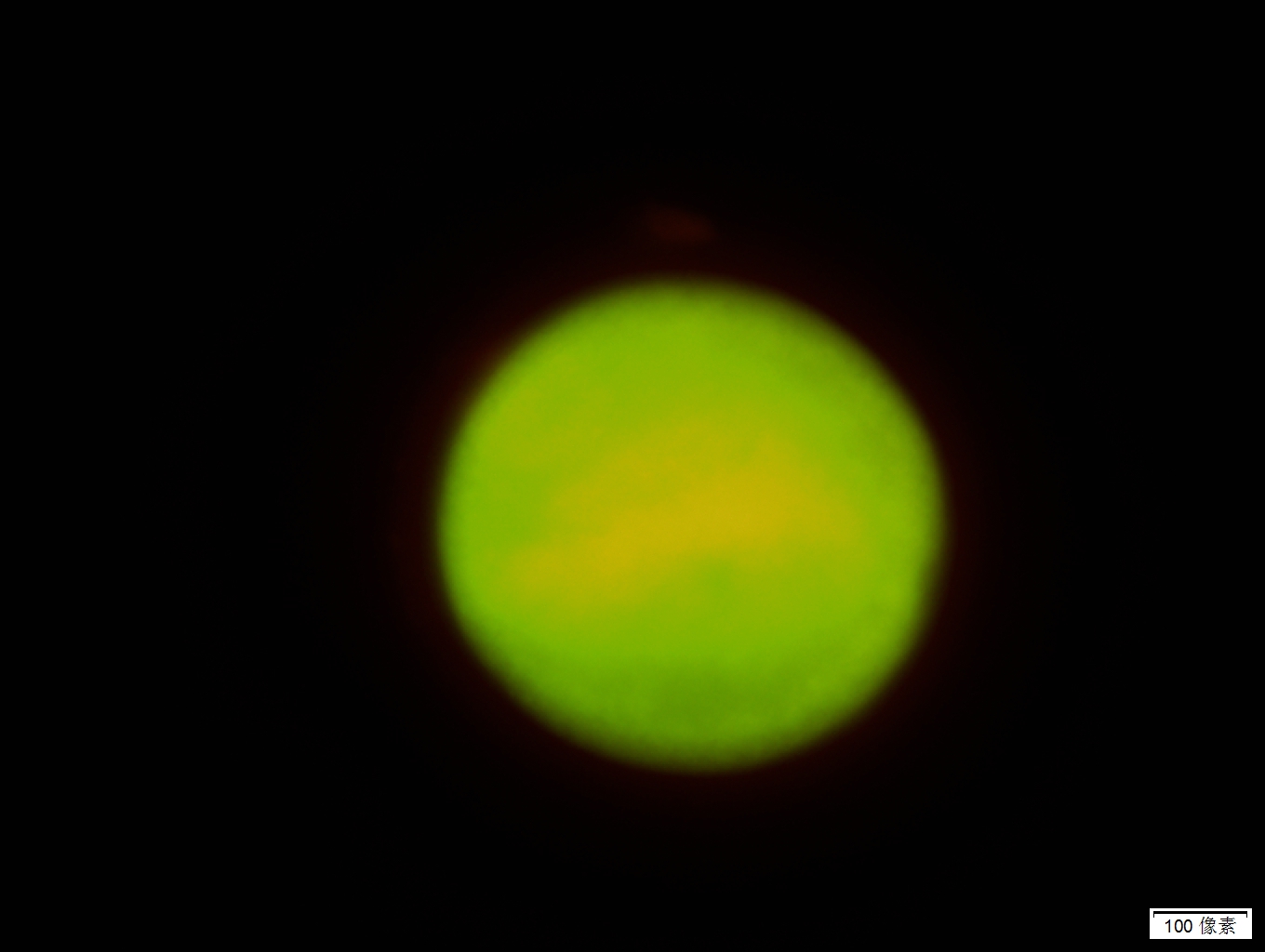

Supplement: Supplementary file 5 [file DataSheet2.ZIP › Figure2í╠/JC-1/JC-3.6mM.jpg]

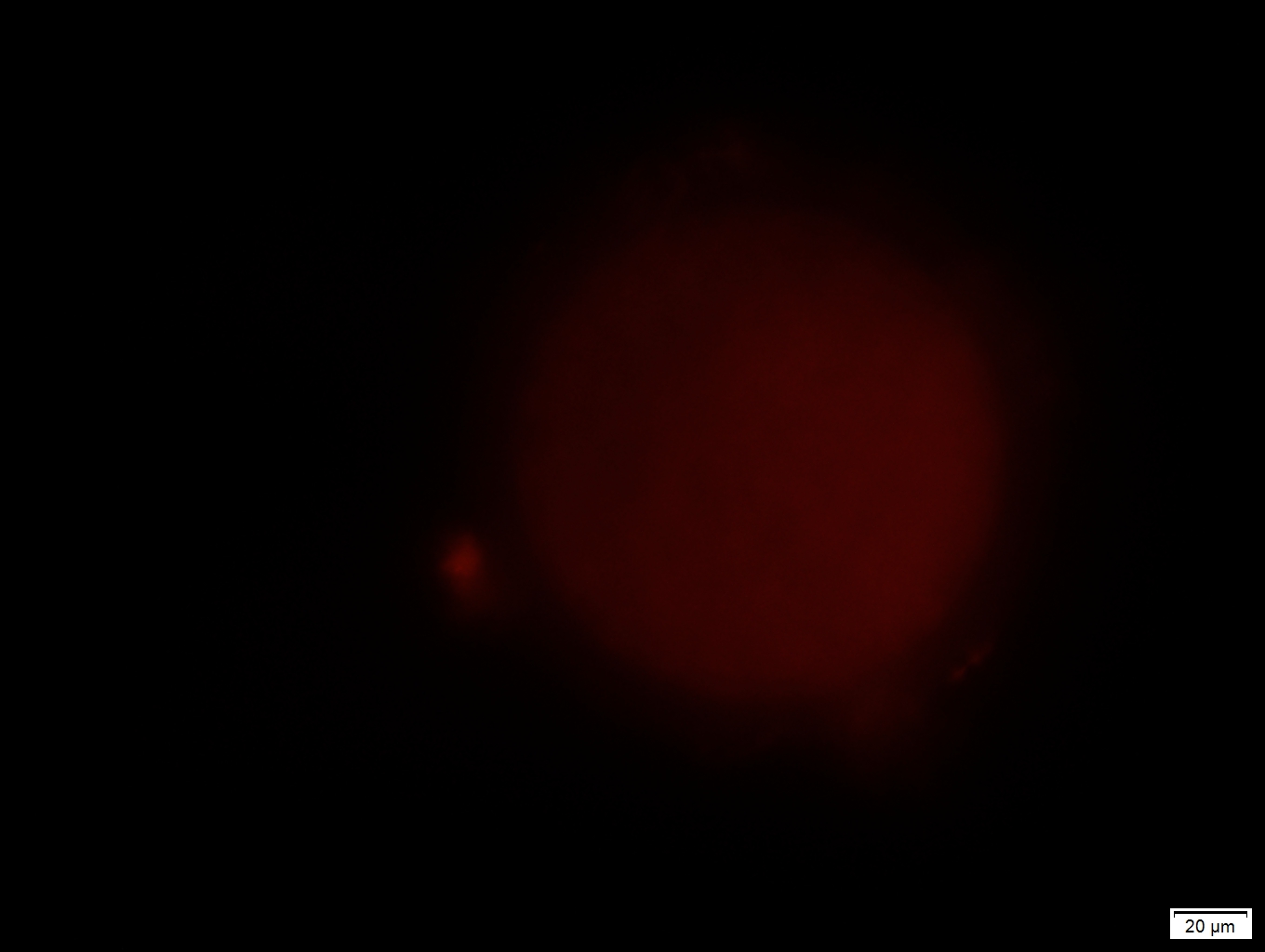

Supplement: Supplementary file 5 [file DataSheet2.ZIP › Figure2í╠/JC-1/JC-C (1).jpg]

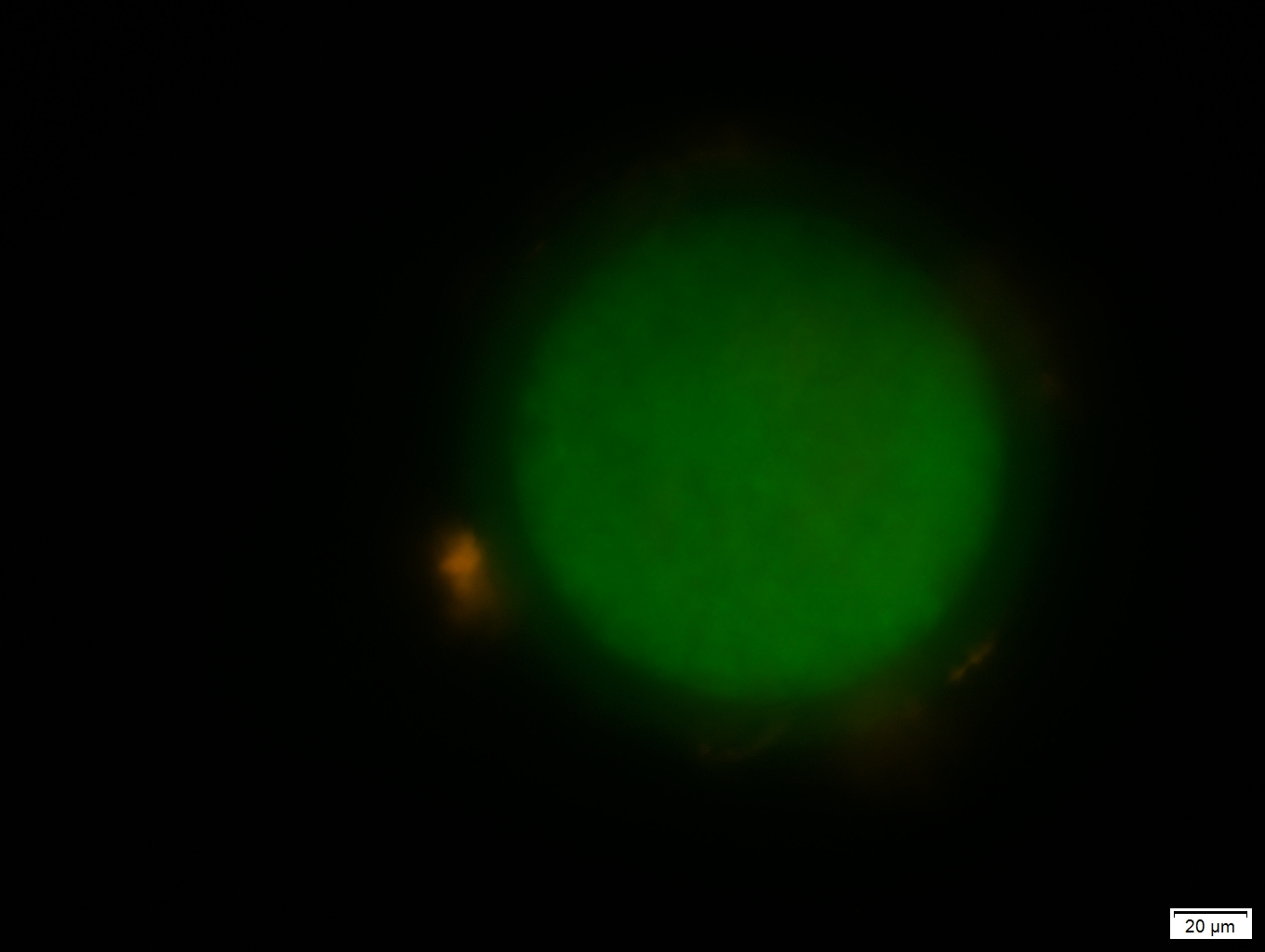

Supplement: Supplementary file 5 [file DataSheet2.ZIP › Figure2í╠/JC-1/JC-C (2).jpg]

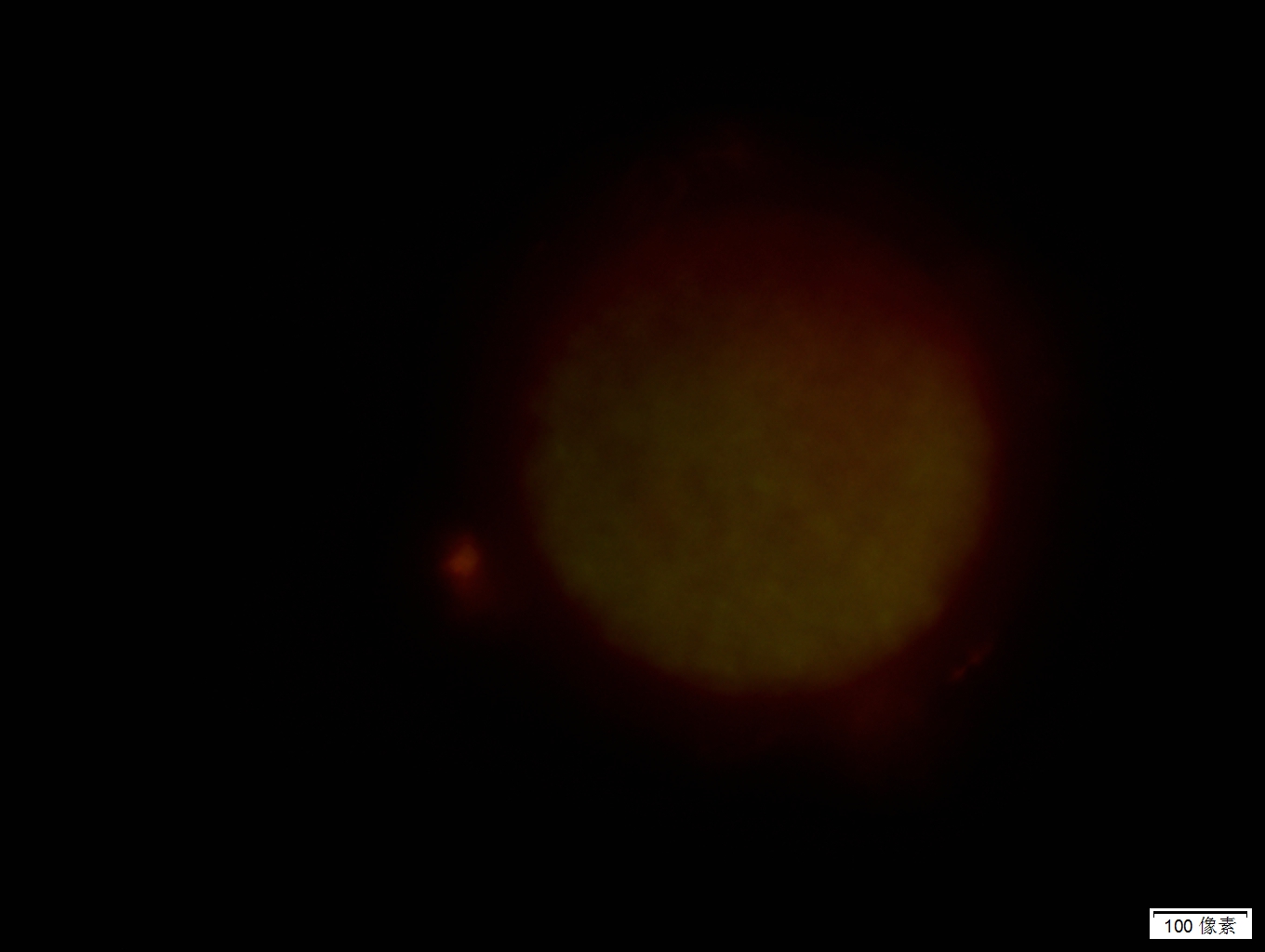

Supplement: Supplementary file 5 [file DataSheet2.ZIP › Figure2í╠/JC-1/JC-C.jpg]

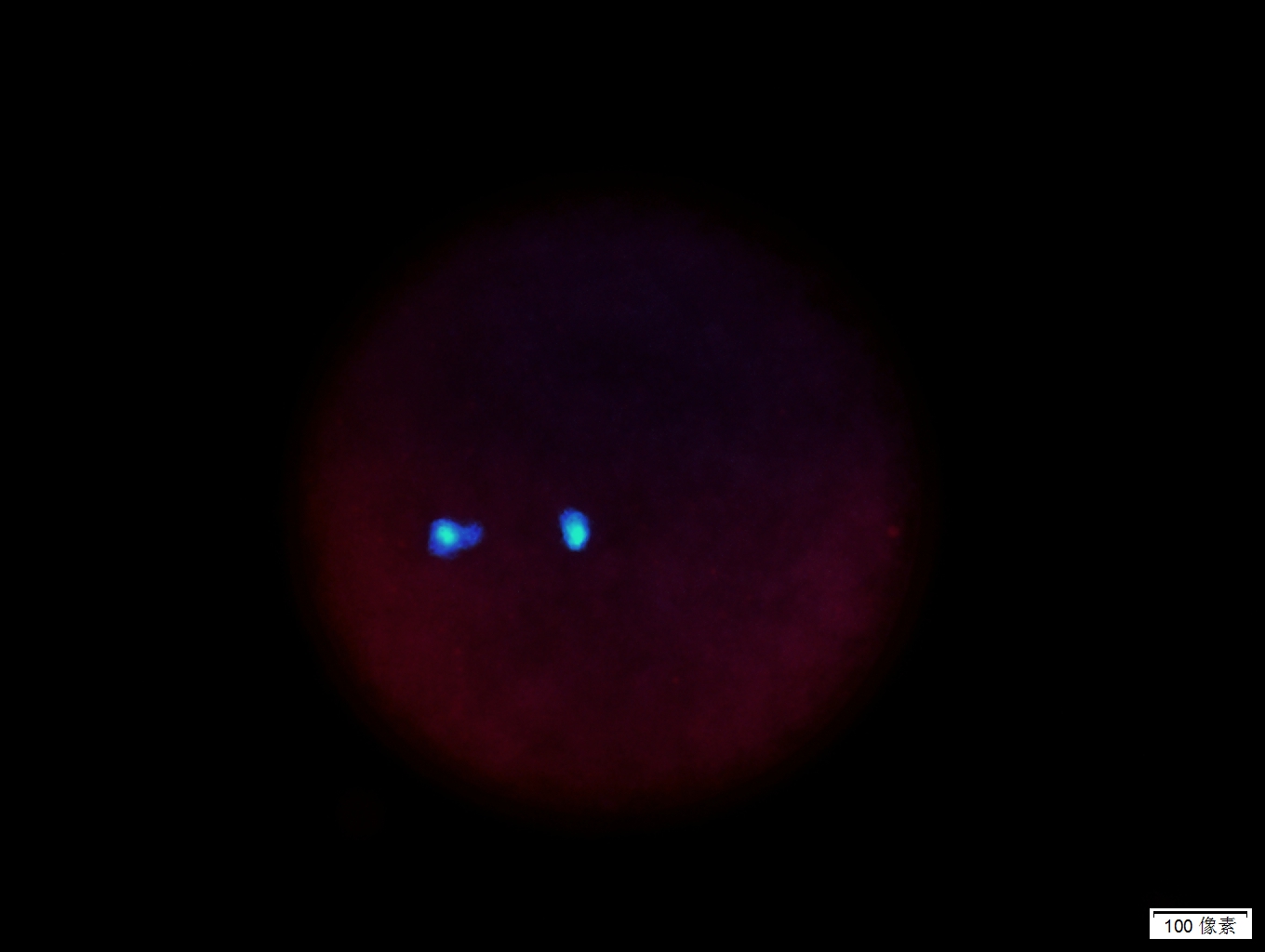

Supplement: Supplementary file 5 [file DataSheet2.ZIP › Figure2í╠/MMP/1.2.jpg]

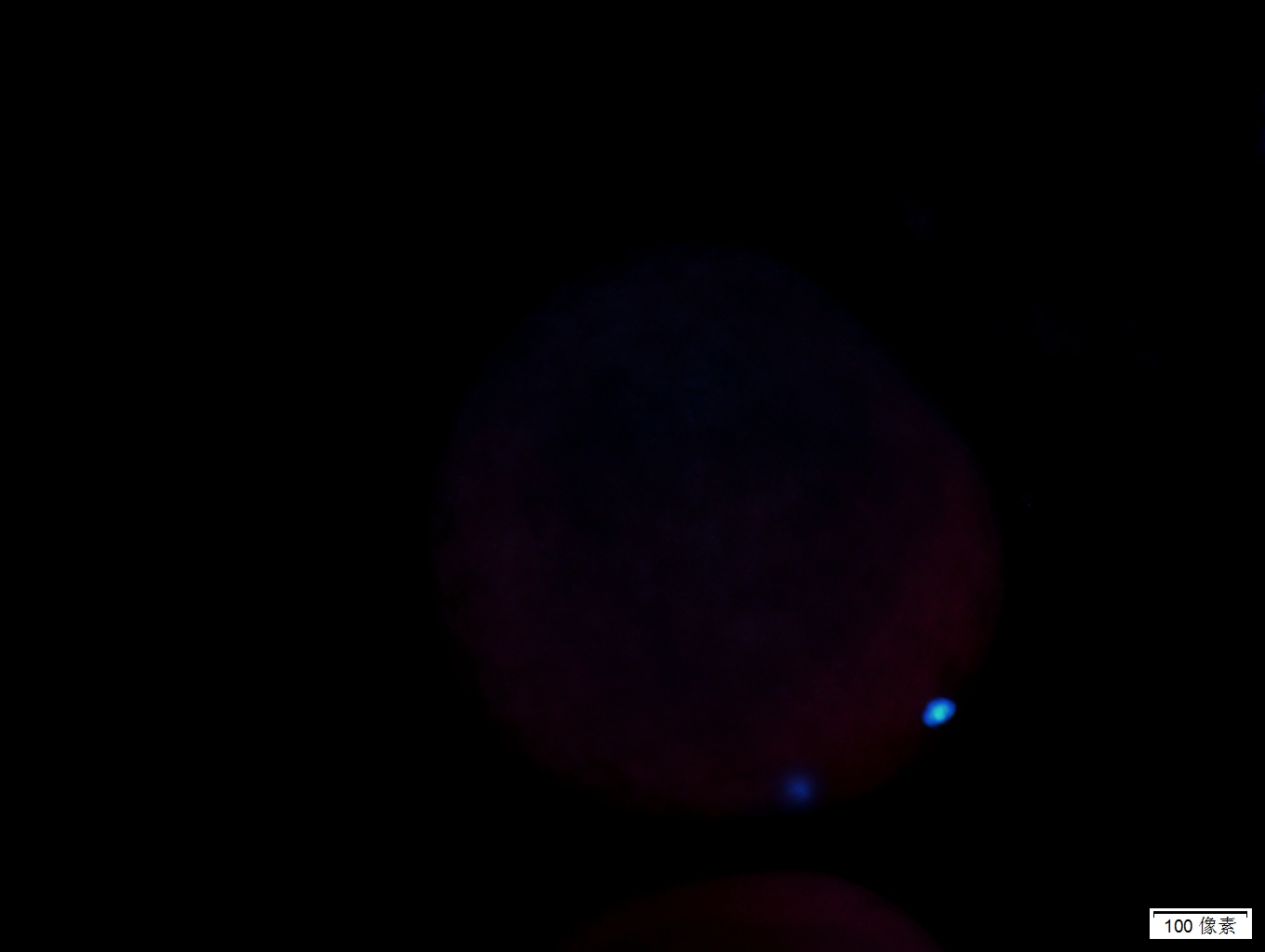

Supplement: Supplementary file 5 [file DataSheet2.ZIP › Figure2í╠/MMP/3.6.jpg]

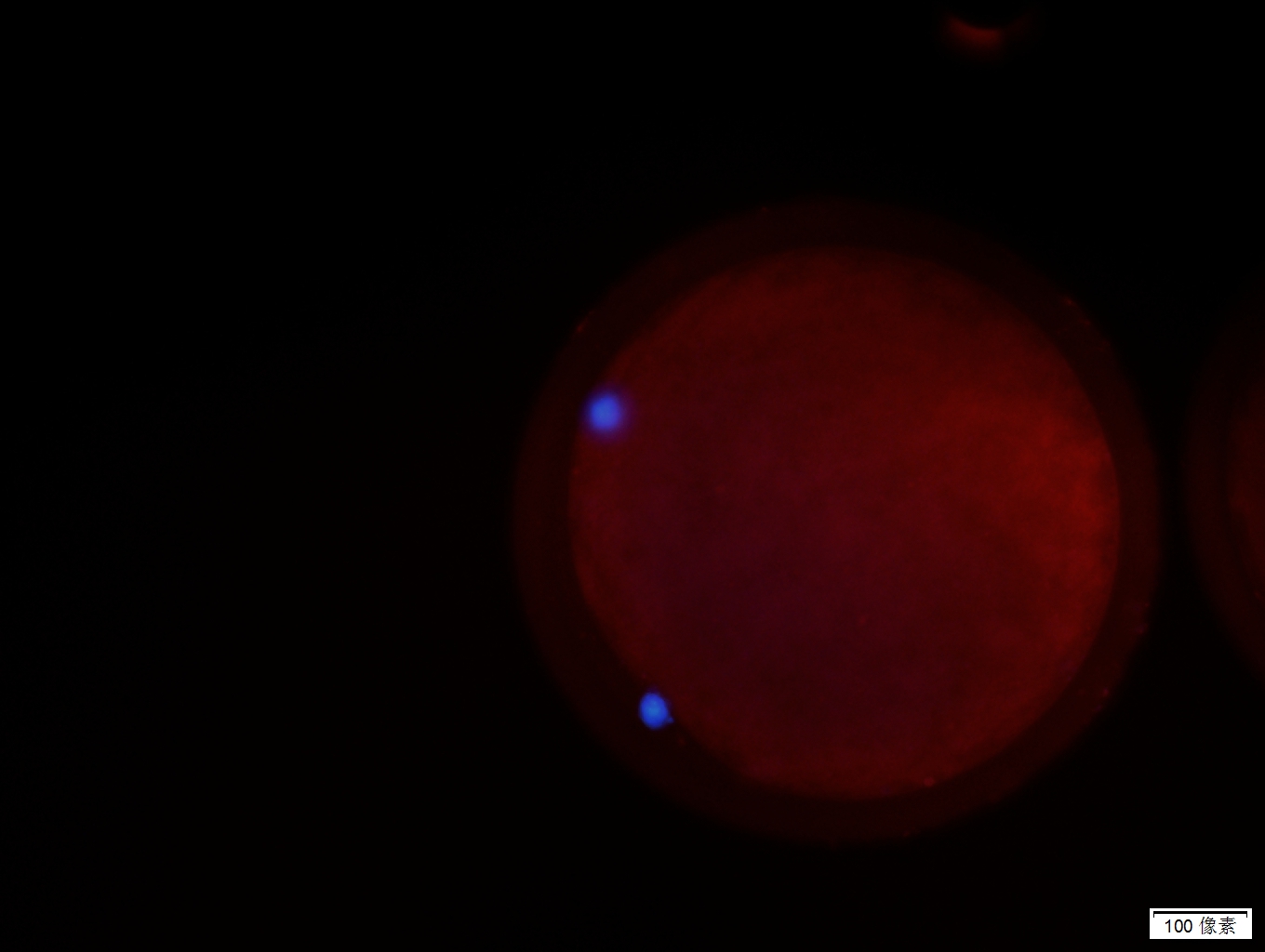

Supplement: Supplementary file 5 [file DataSheet2.ZIP › Figure2í╠/MMP/C.jpg]

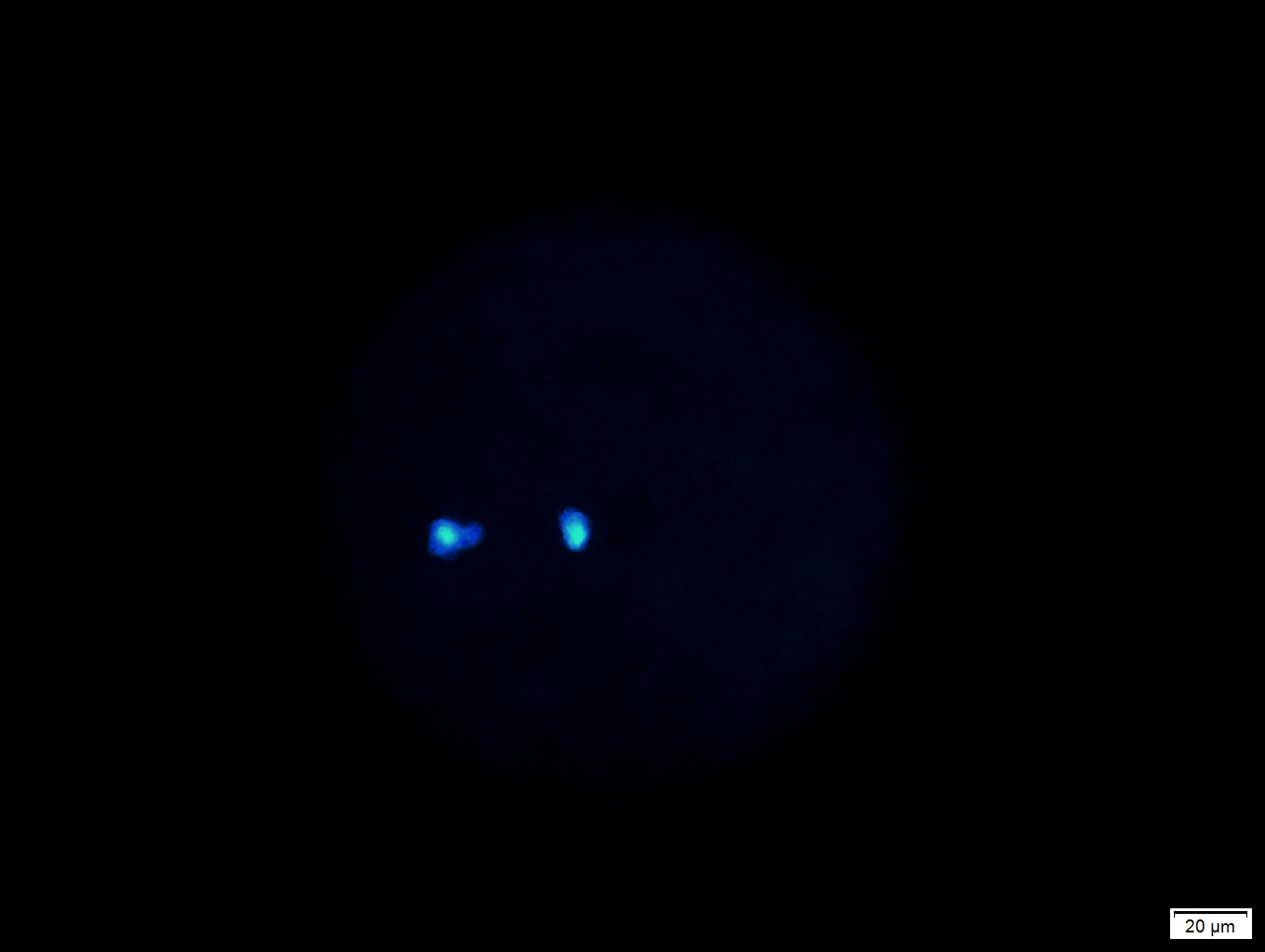

Supplement: Supplementary file 5 [file DataSheet2.ZIP › Figure2í╠/MMP/MMP-1.2mM (2).jpg]

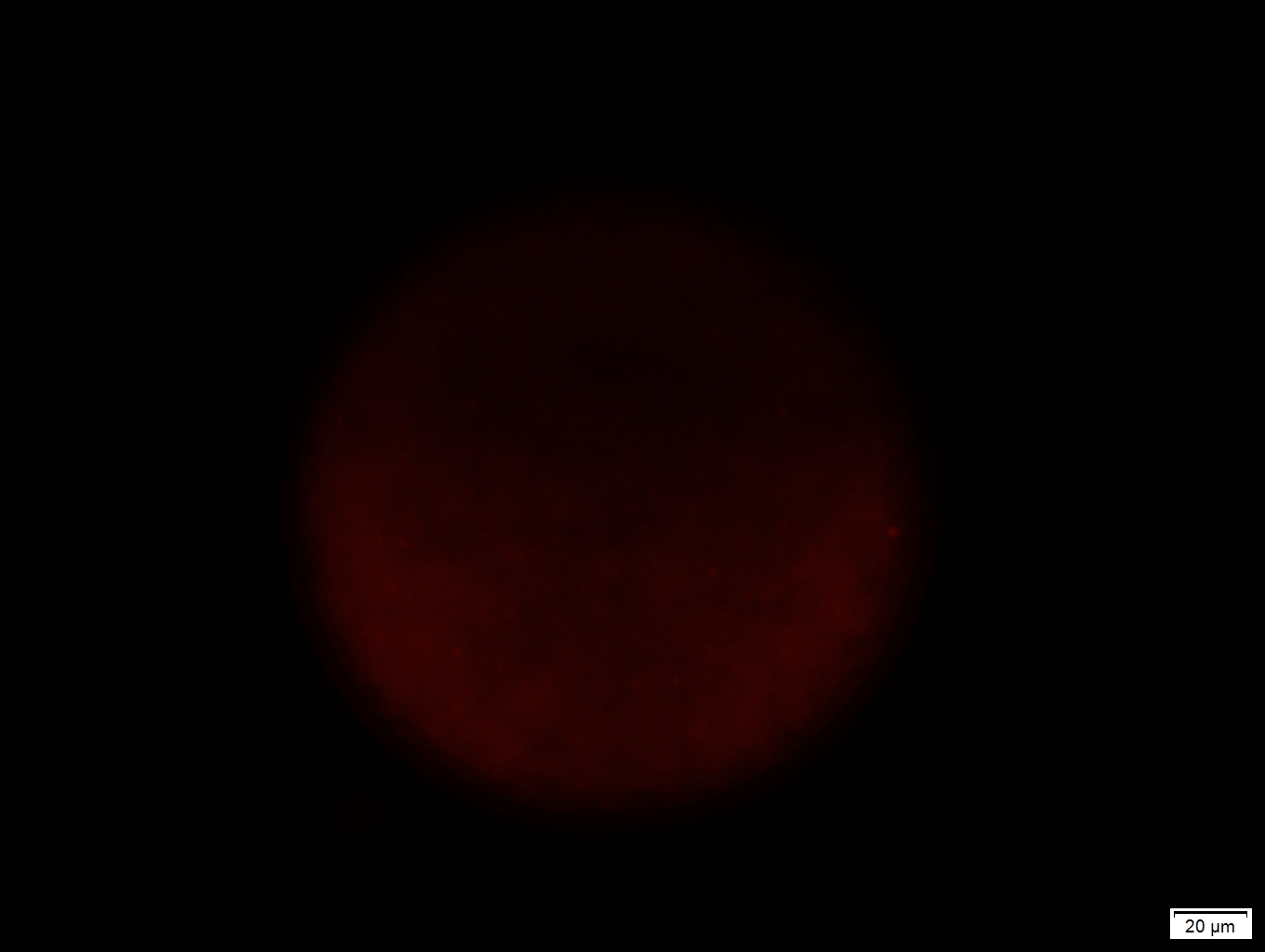

Supplement: Supplementary file 5 [file DataSheet2.ZIP › Figure2í╠/MMP/MMP-1.2mM (1).jpg]

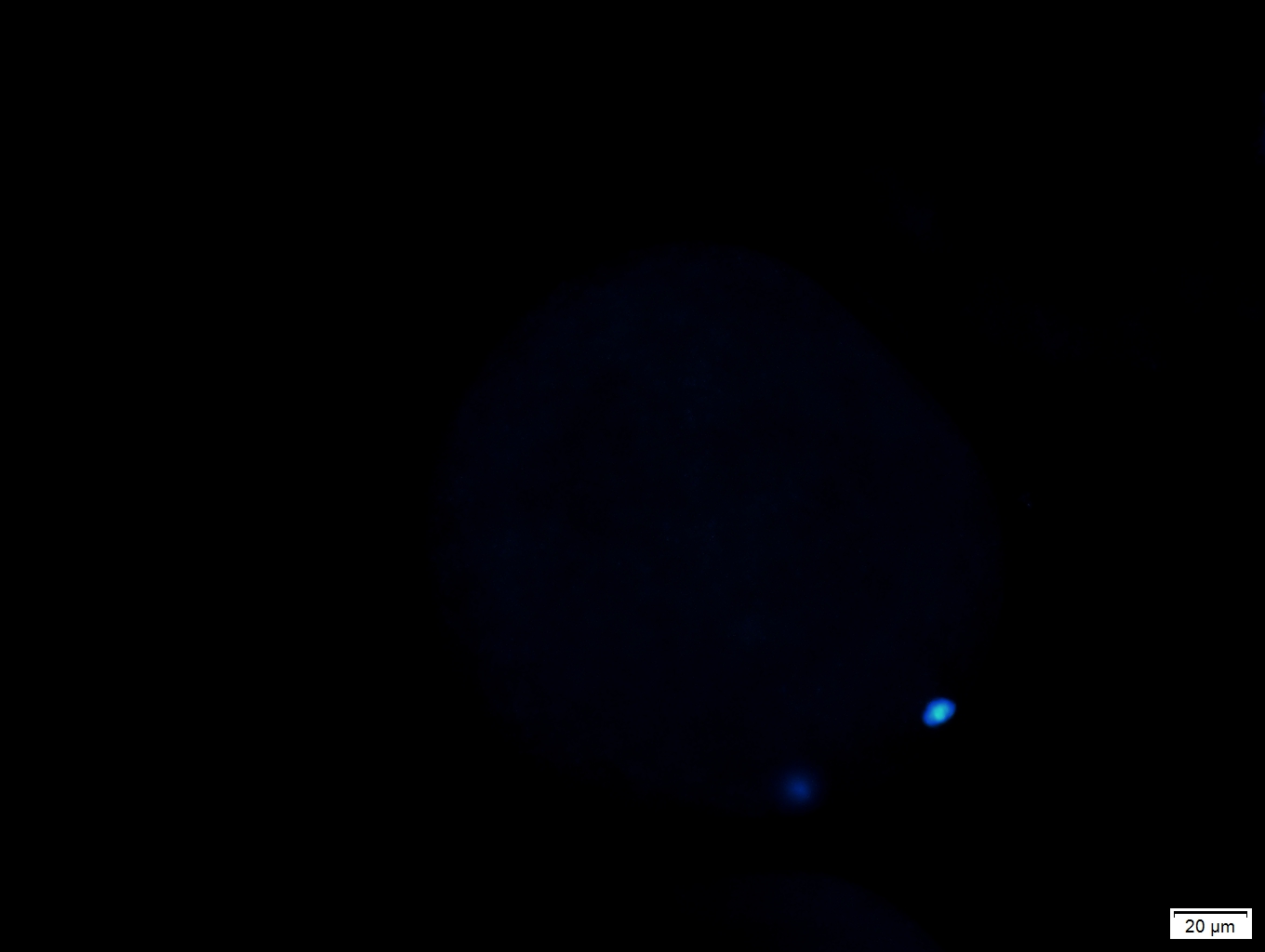

Supplement: Supplementary file 5 [file DataSheet2.ZIP › Figure2í╠/MMP/MMP-3.6mM (1).jpg]

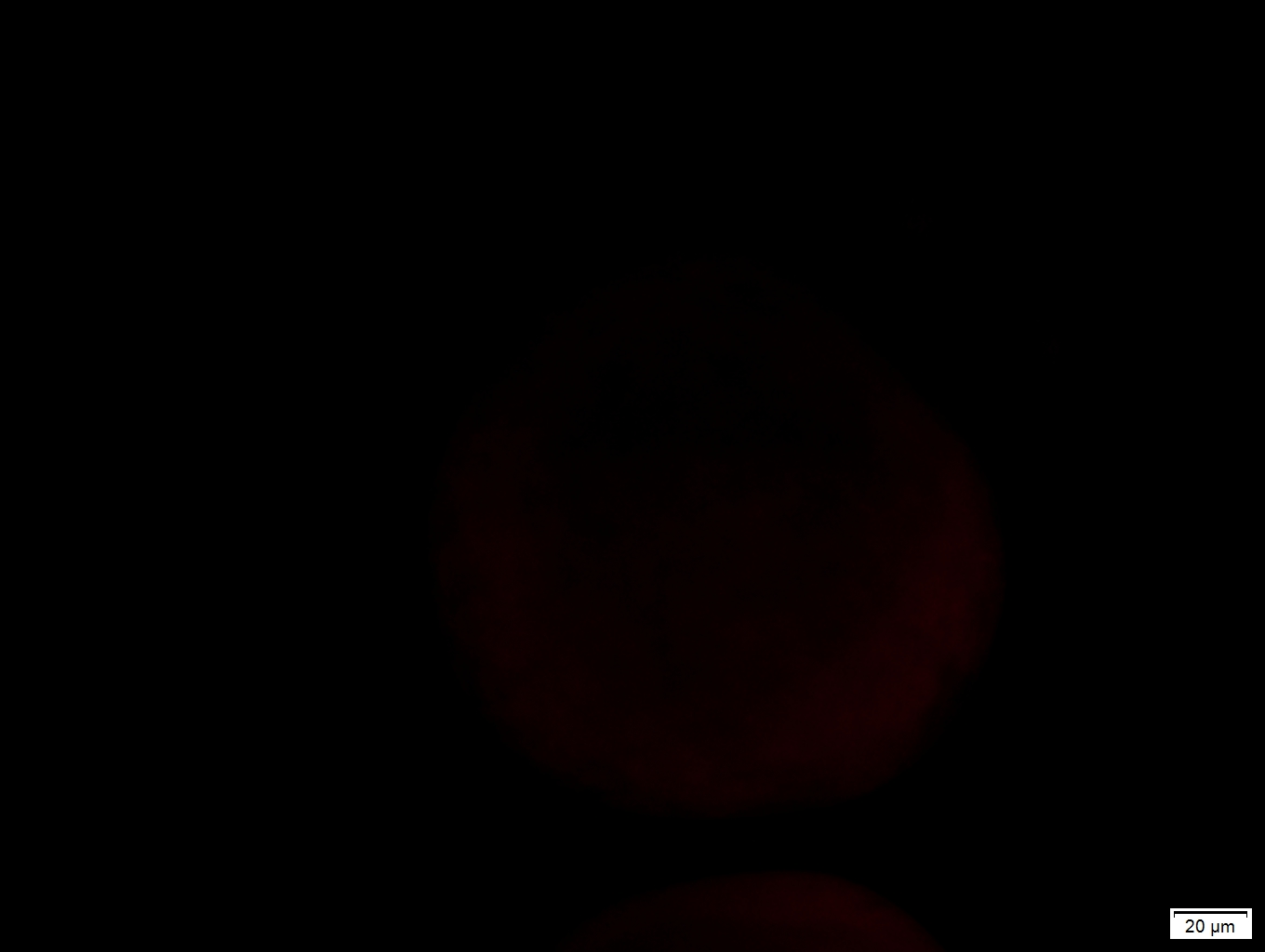

Supplement: Supplementary file 5 [file DataSheet2.ZIP › Figure2í╠/MMP/MMP-3.6mM (2).jpg]

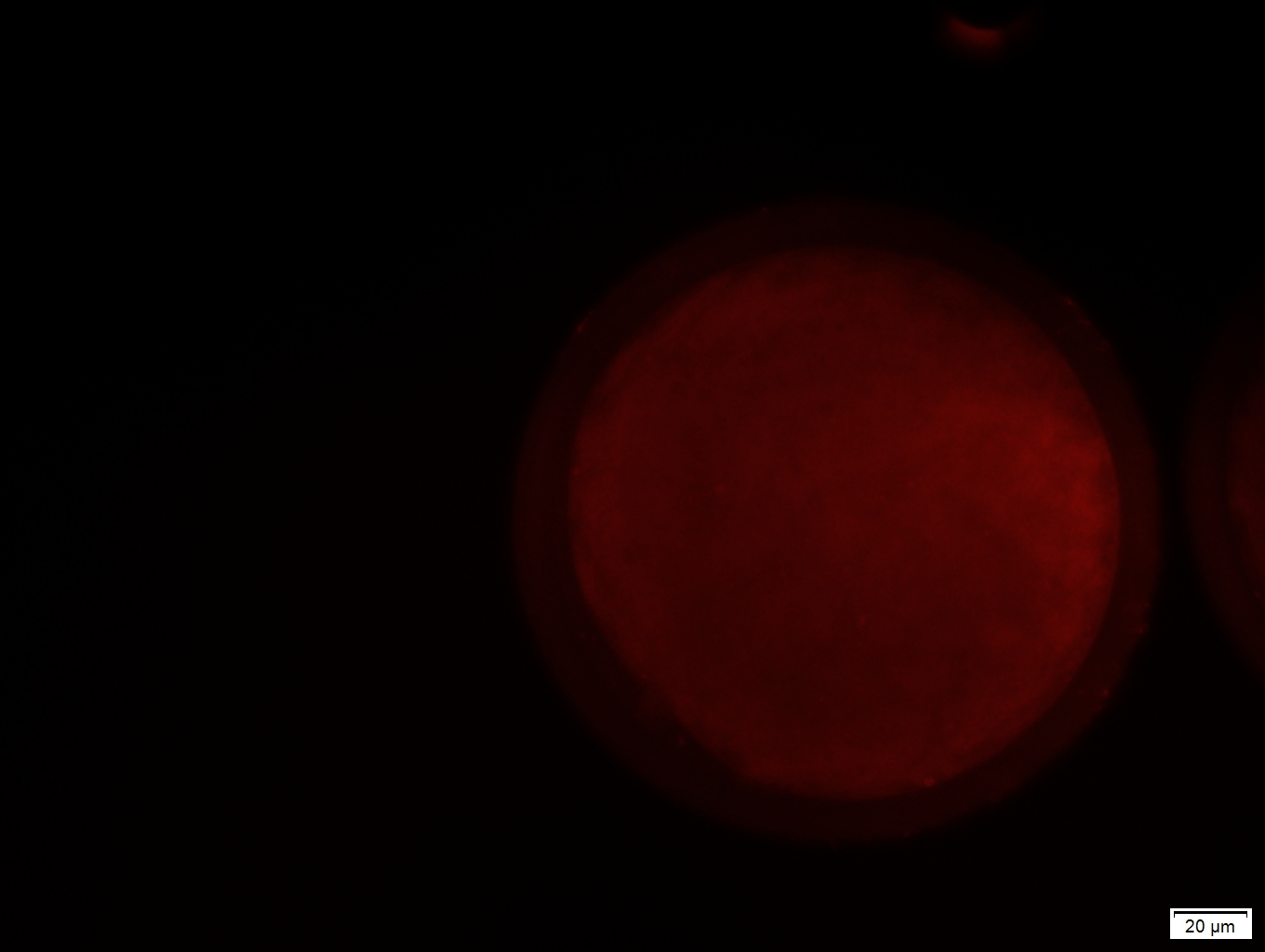

Supplement: Supplementary file 5 [file DataSheet2.ZIP › Figure2í╠/MMP/MMP-C (1).jpg]

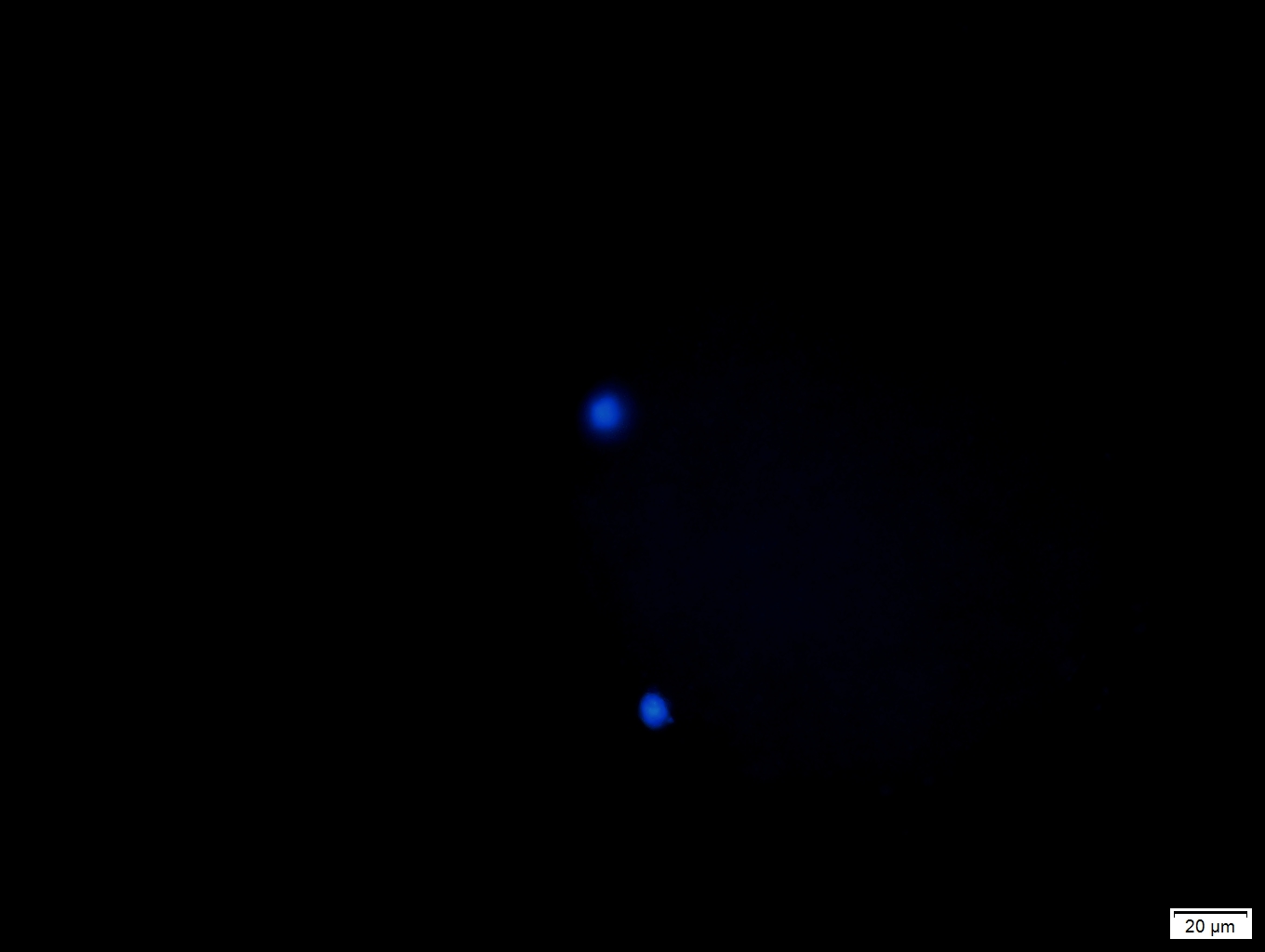

Supplement: Supplementary file 5 [file DataSheet2.ZIP › Figure2í╠/MMP/MMP-C (2).jpg]

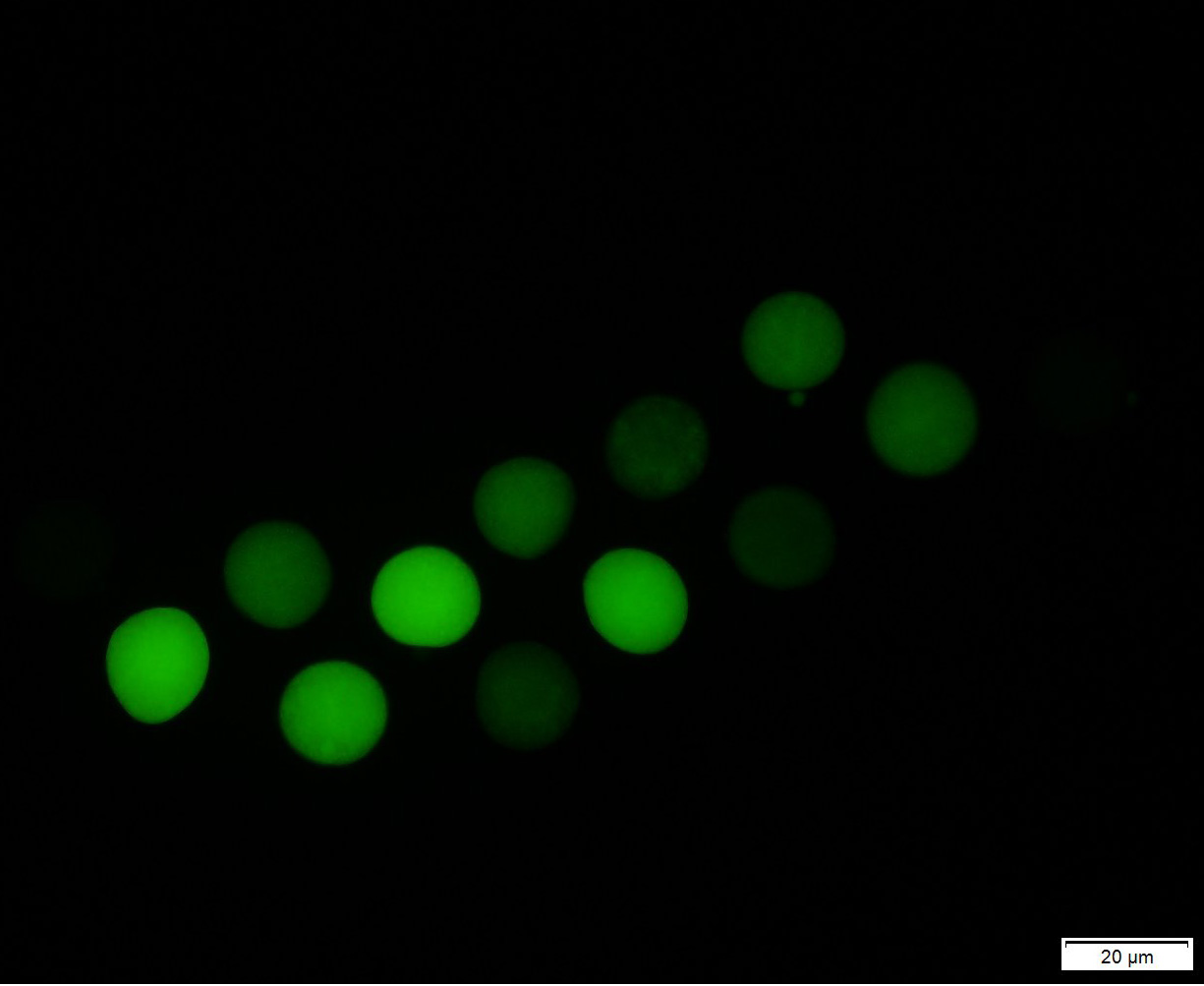

Supplement: Supplementary file 5 [file DataSheet2.ZIP › Figure2í╠/ROS/1.2.jpg]

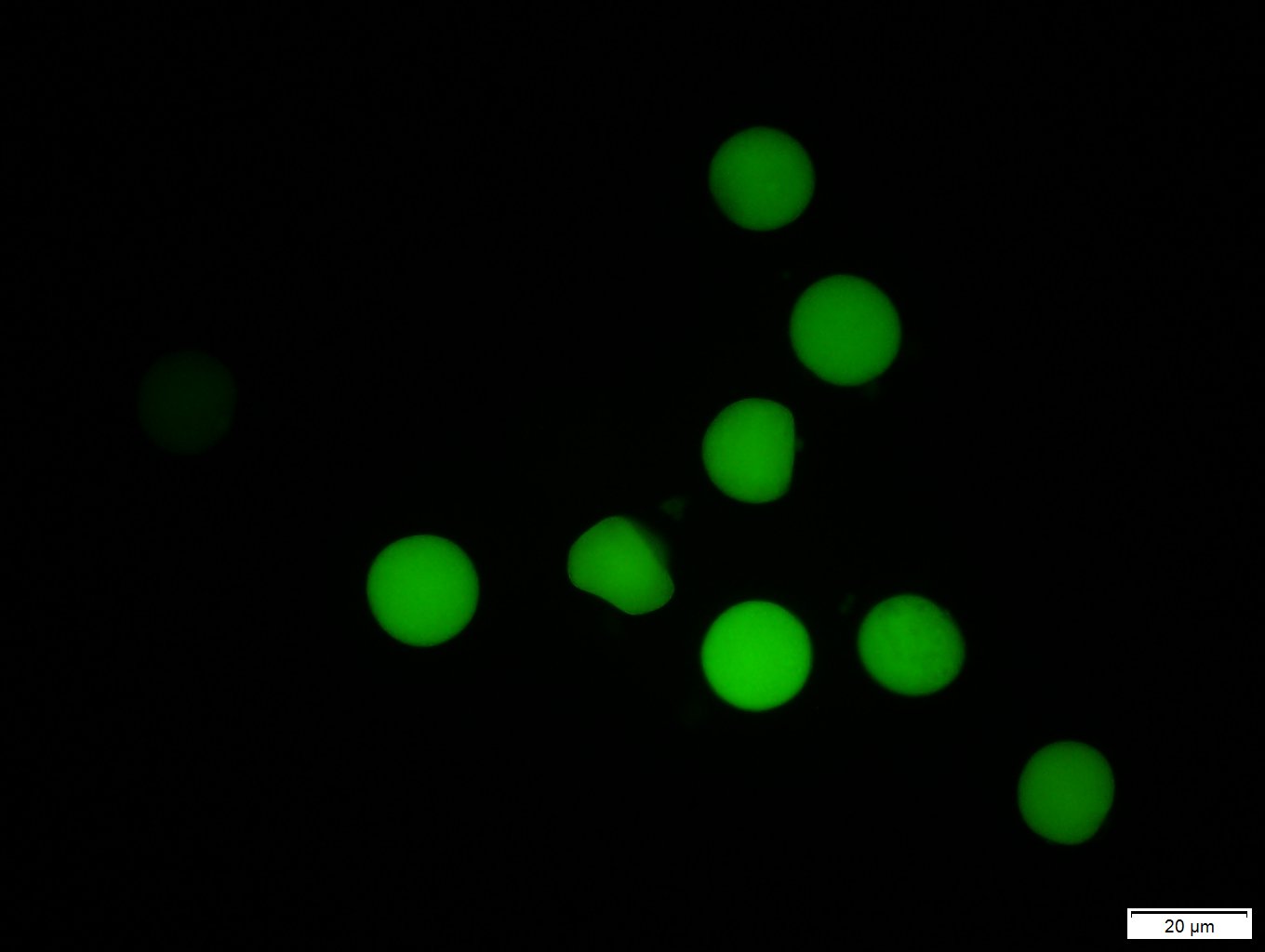

Supplement: Supplementary file 5 [file DataSheet2.ZIP › Figure2í╠/ROS/3.6.jpg]

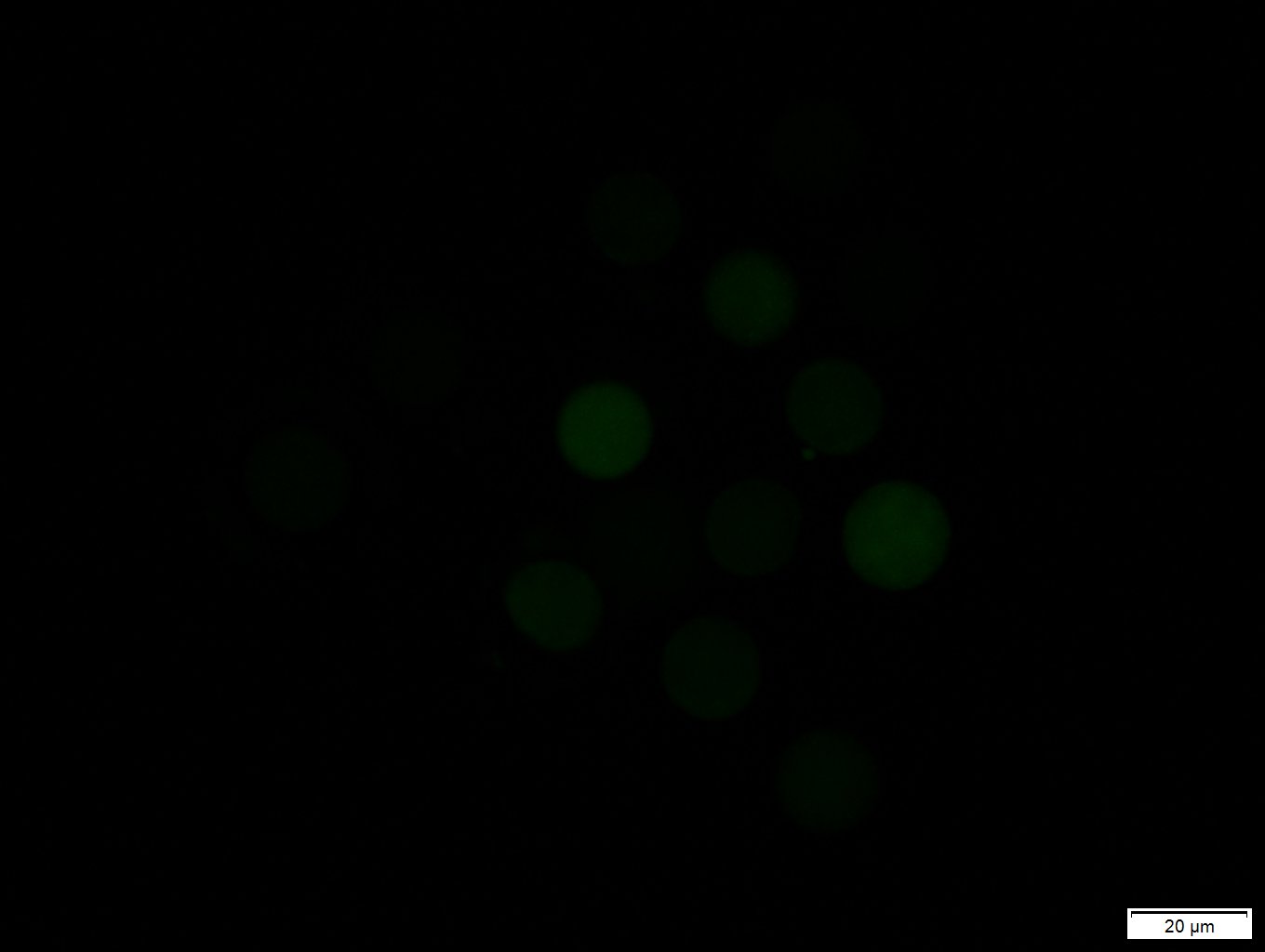

Supplement: Supplementary file 5 [file DataSheet2.ZIP › Figure2í╠/ROS/C.jpg]

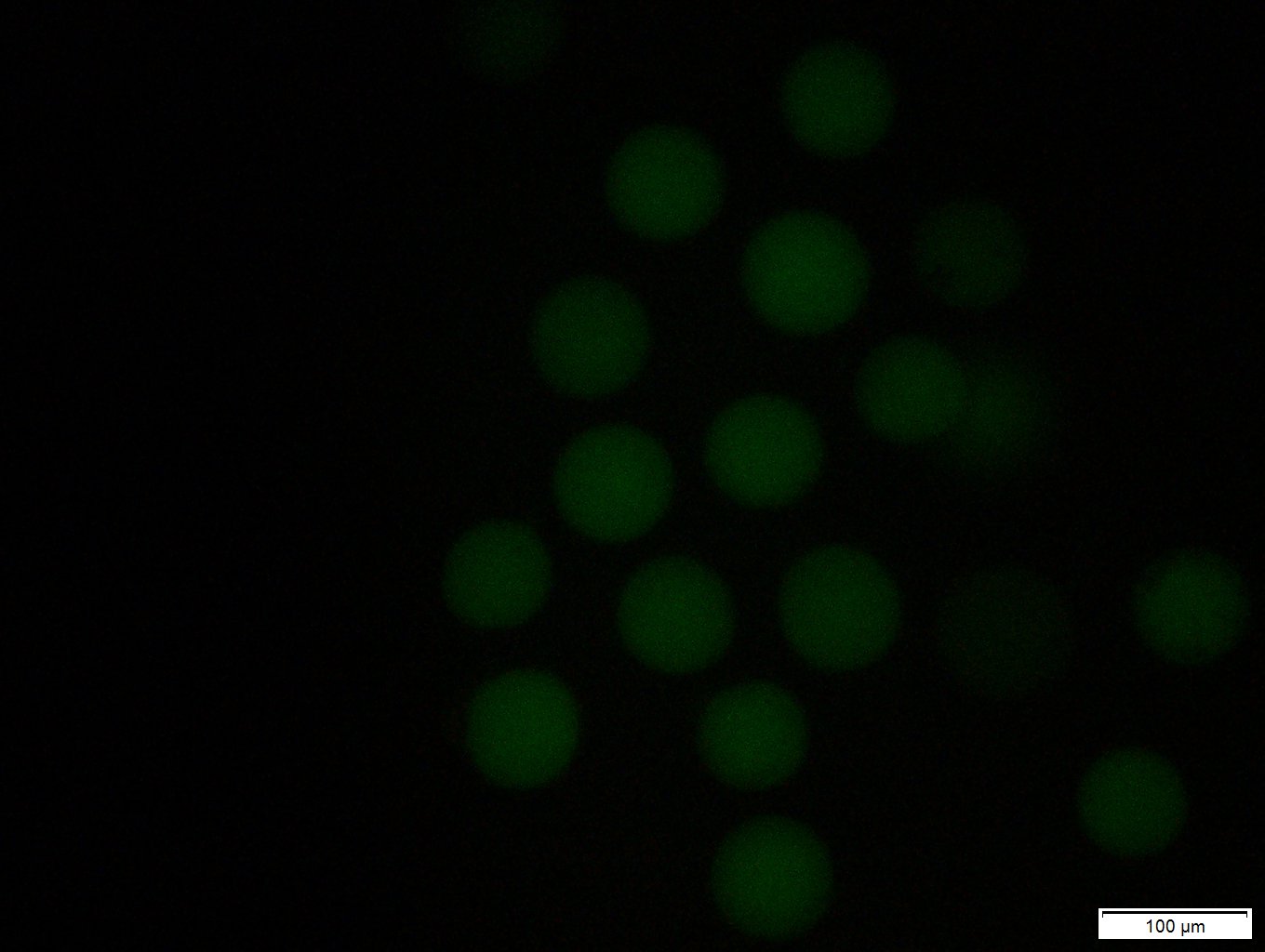

Supplement: Supplementary file 7 [file DataSheet5.ZIP › Figure5í╠/ATP/B-ATP.jpg]

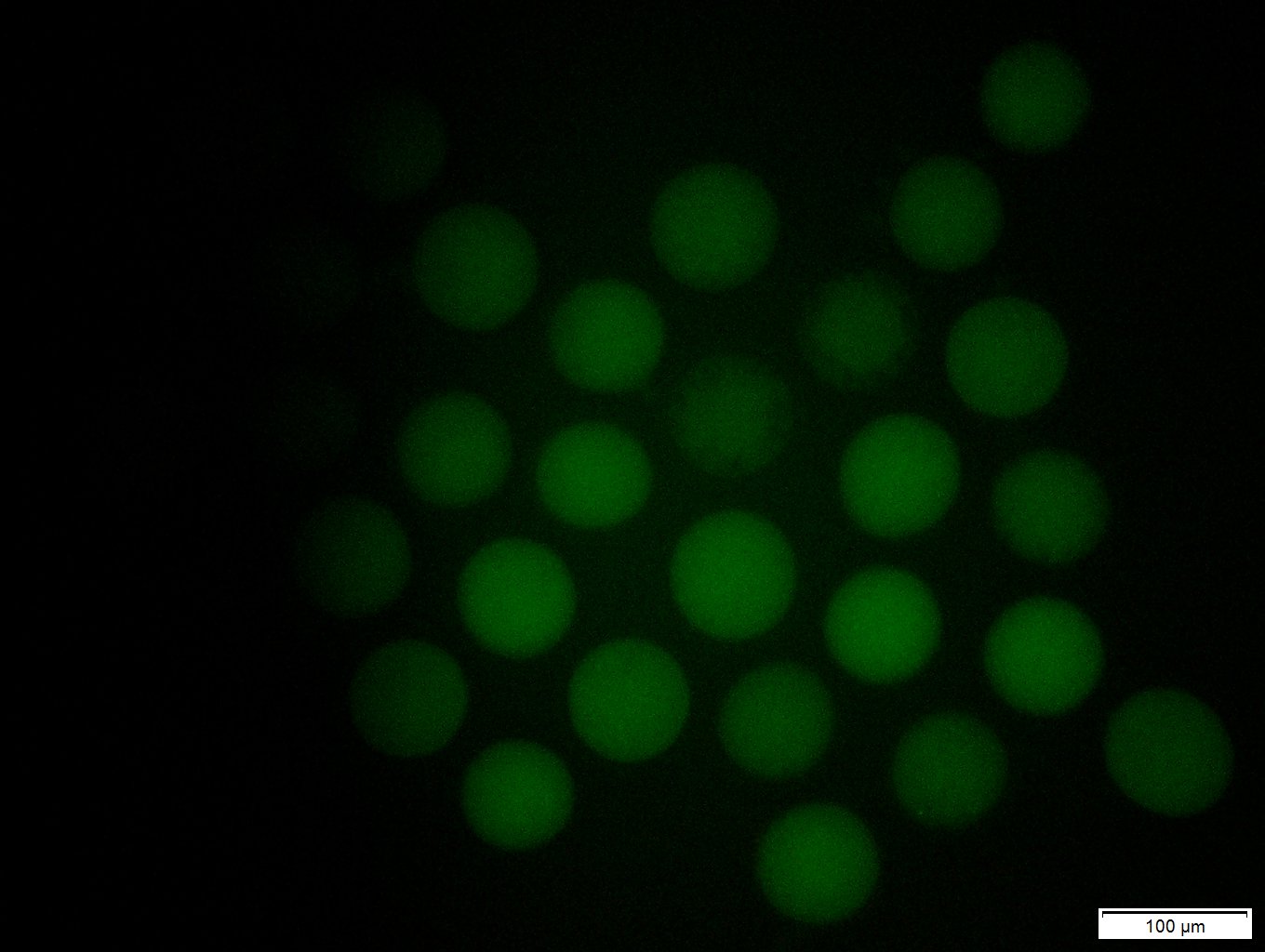

Supplement: Supplementary file 7 [file DataSheet5.ZIP › Figure5í╠/ATP/D-ATP.jpg]

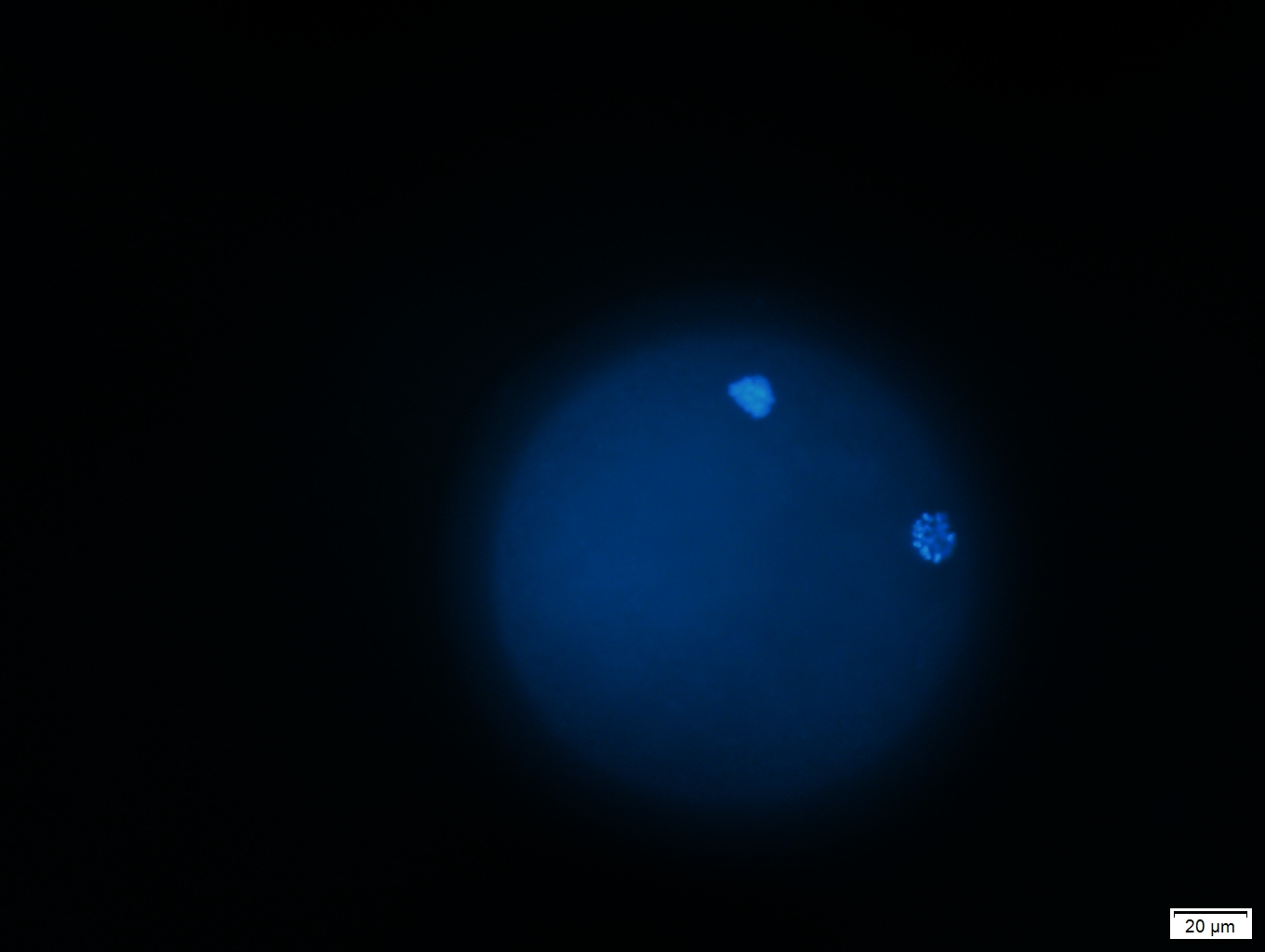

Supplement: Supplementary file 7 [file DataSheet5.ZIP › Figure5í╠/DCA┼¿╢╚ A/p-PDH-0 (1).jpg]

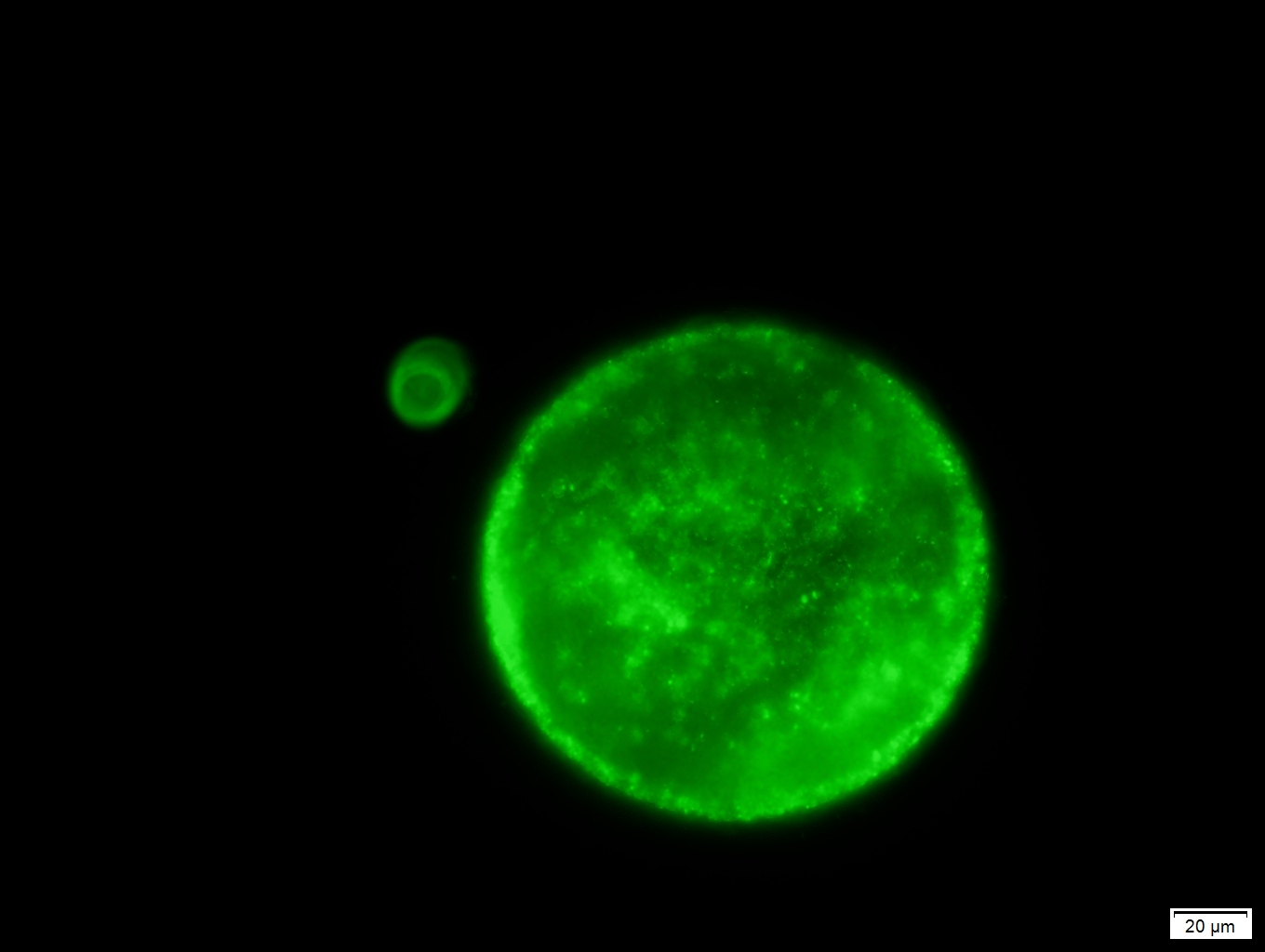

Supplement: Supplementary file 7 [file DataSheet5.ZIP › Figure5í╠/DCA┼¿╢╚ A/p-PDH-0 (2).jpg]

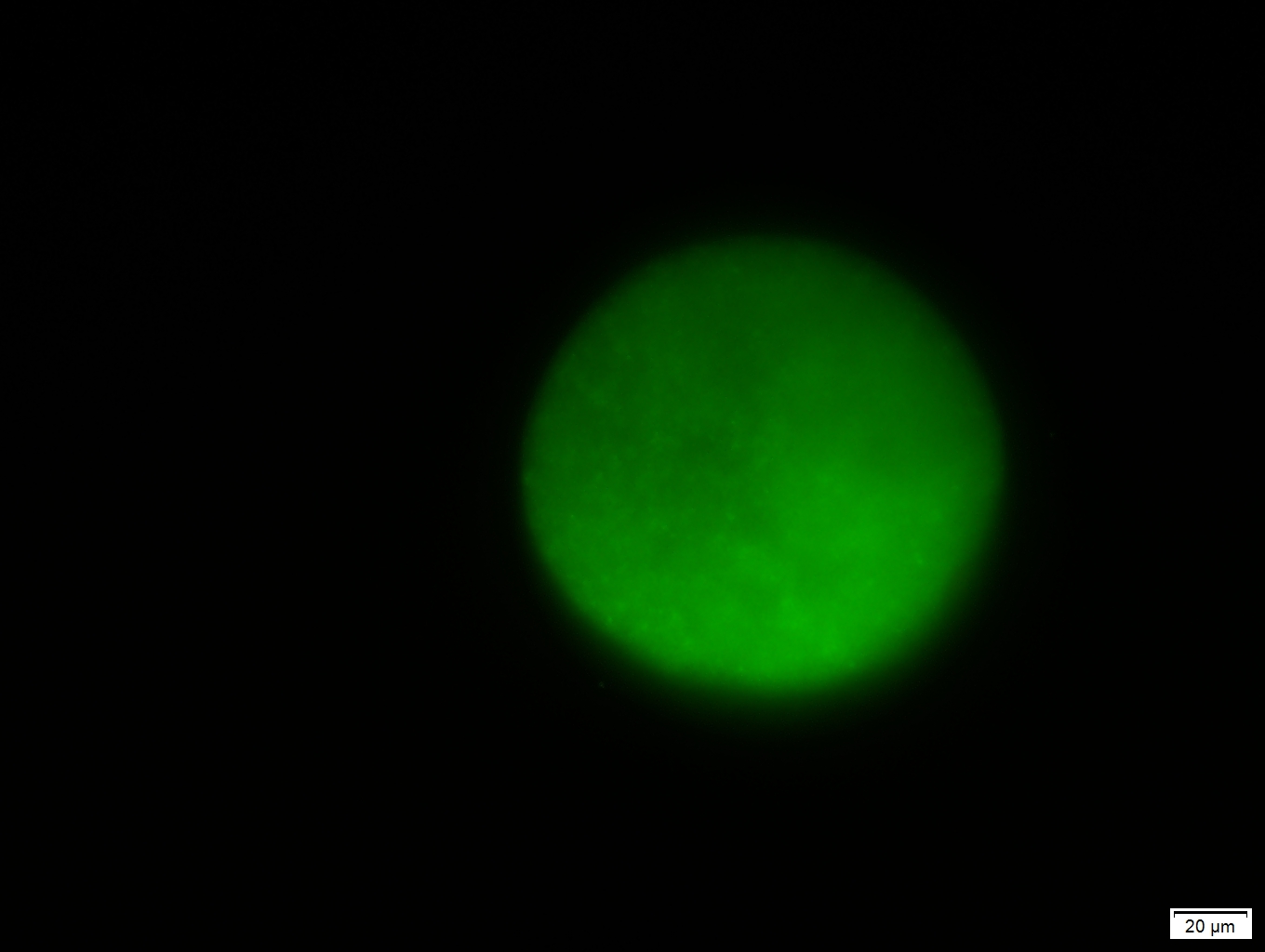

Supplement: Supplementary file 7 [file DataSheet5.ZIP › Figure5í╠/DCA┼¿╢╚ A/P-PDH-1 (1).jpg]

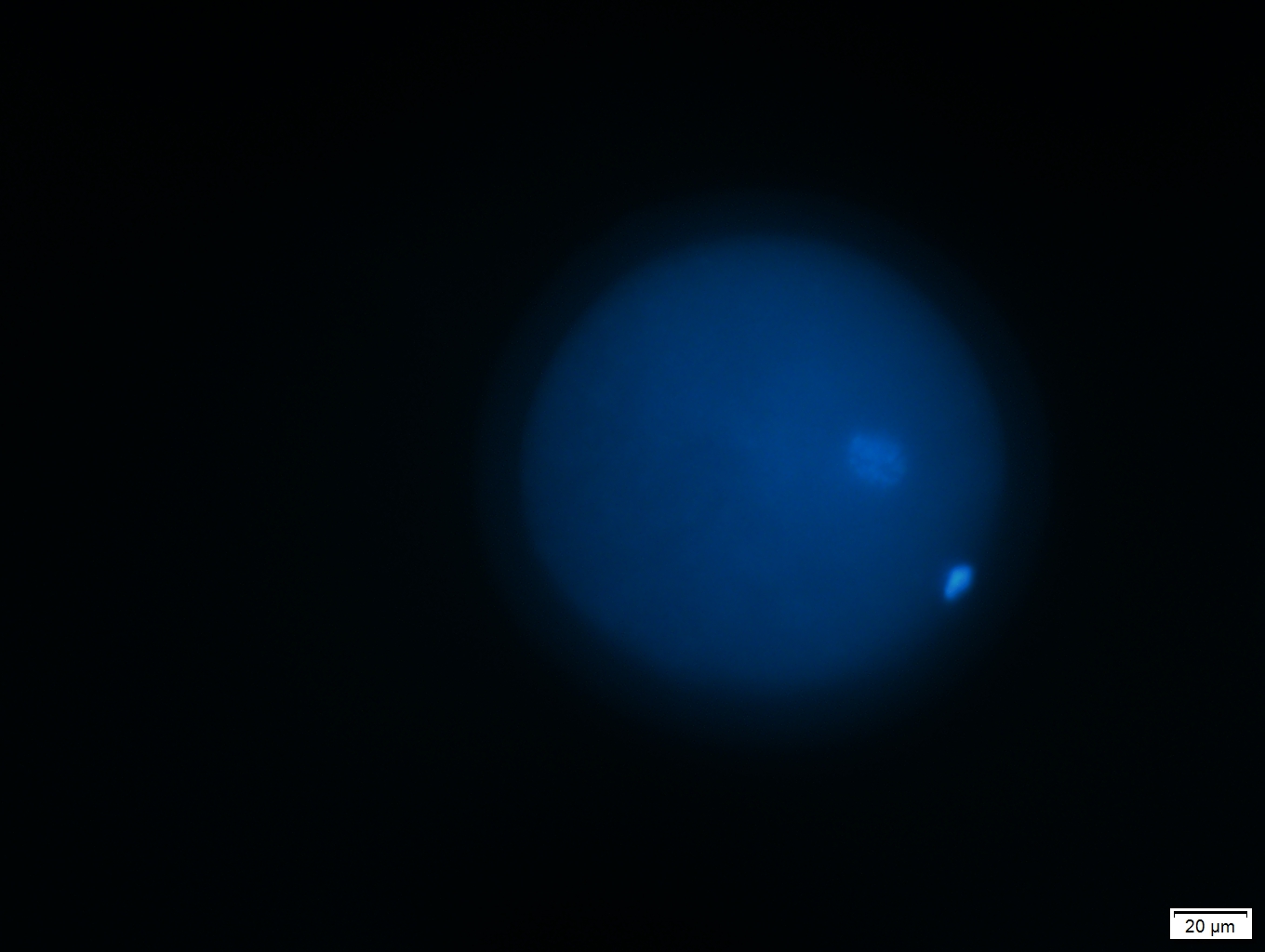

Supplement: Supplementary file 7 [file DataSheet5.ZIP › Figure5í╠/DCA┼¿╢╚ A/P-PDH-1 (2).jpg]
