# Supplementary figures and images for: β-hydroxybutyrate impairs bovine oocyte maturation via pyruvate dehydrogenase (PDH) associated energy metabolism abnormality (part 2 of 2)
Source: Front Pharmacol. 2023 Aug 11;14:1243243. doi: 10.3389/fphar.2023.1243243 (PMC10450765; doi:10.3389/fphar.2023.1243243)

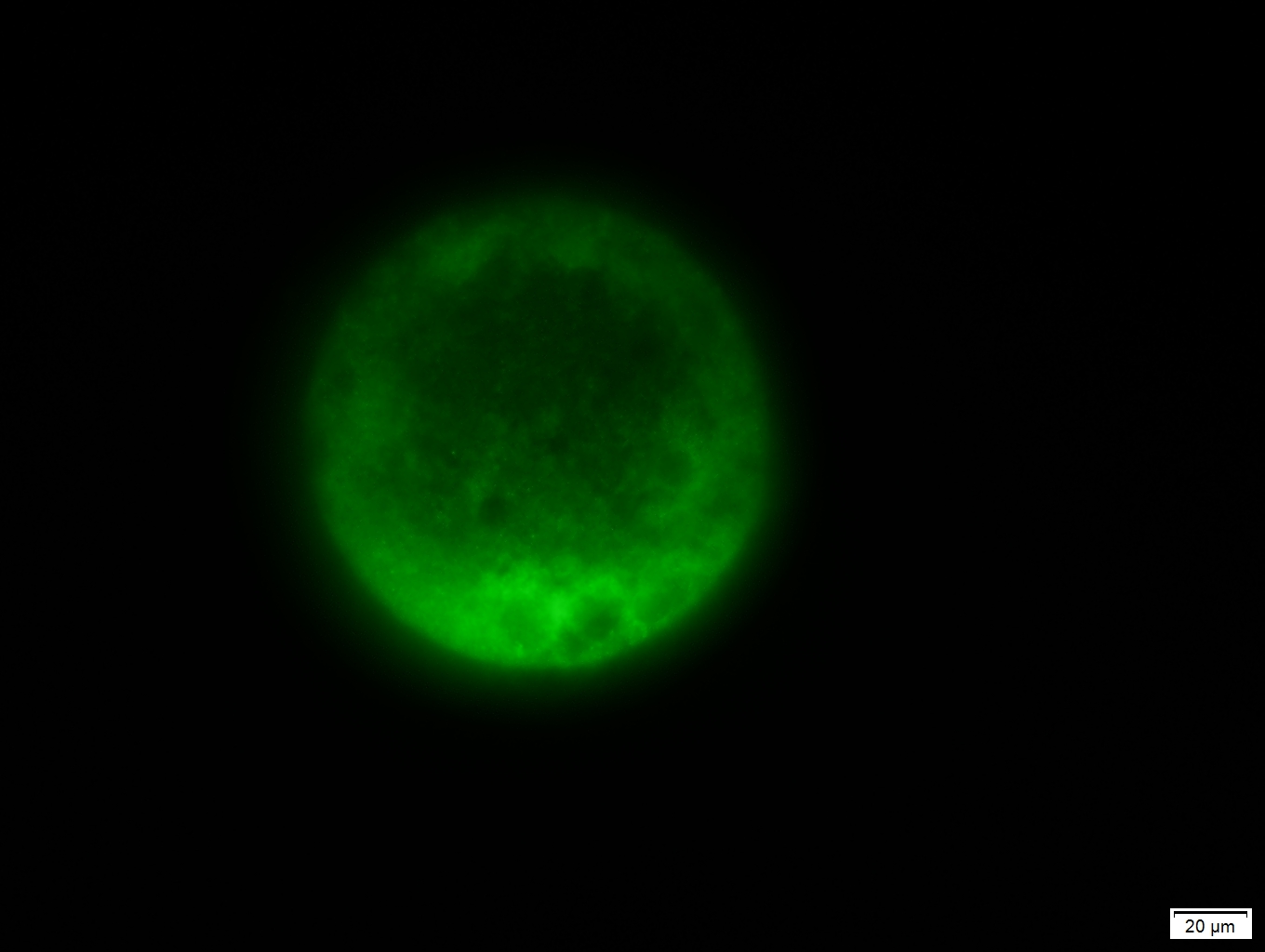

Supplement: Supplementary file 7 [file DataSheet5.ZIP › Figure5í╠/DCA┼¿╢╚ A/P-PDH-2 (1).jpg]

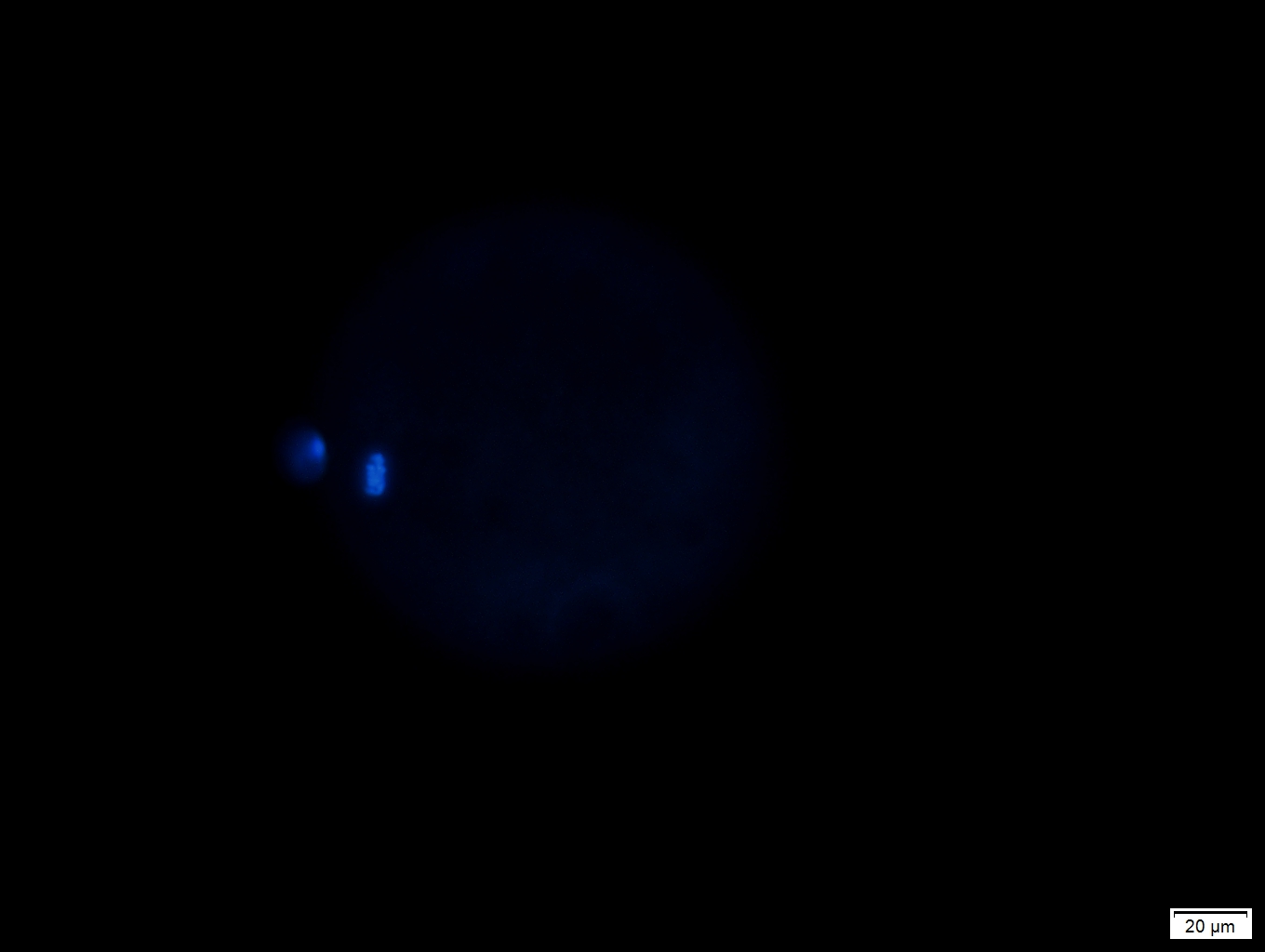

Supplement: Supplementary file 7 [file DataSheet5.ZIP › Figure5í╠/DCA┼¿╢╚ A/P-PDH-2 (2).jpg]

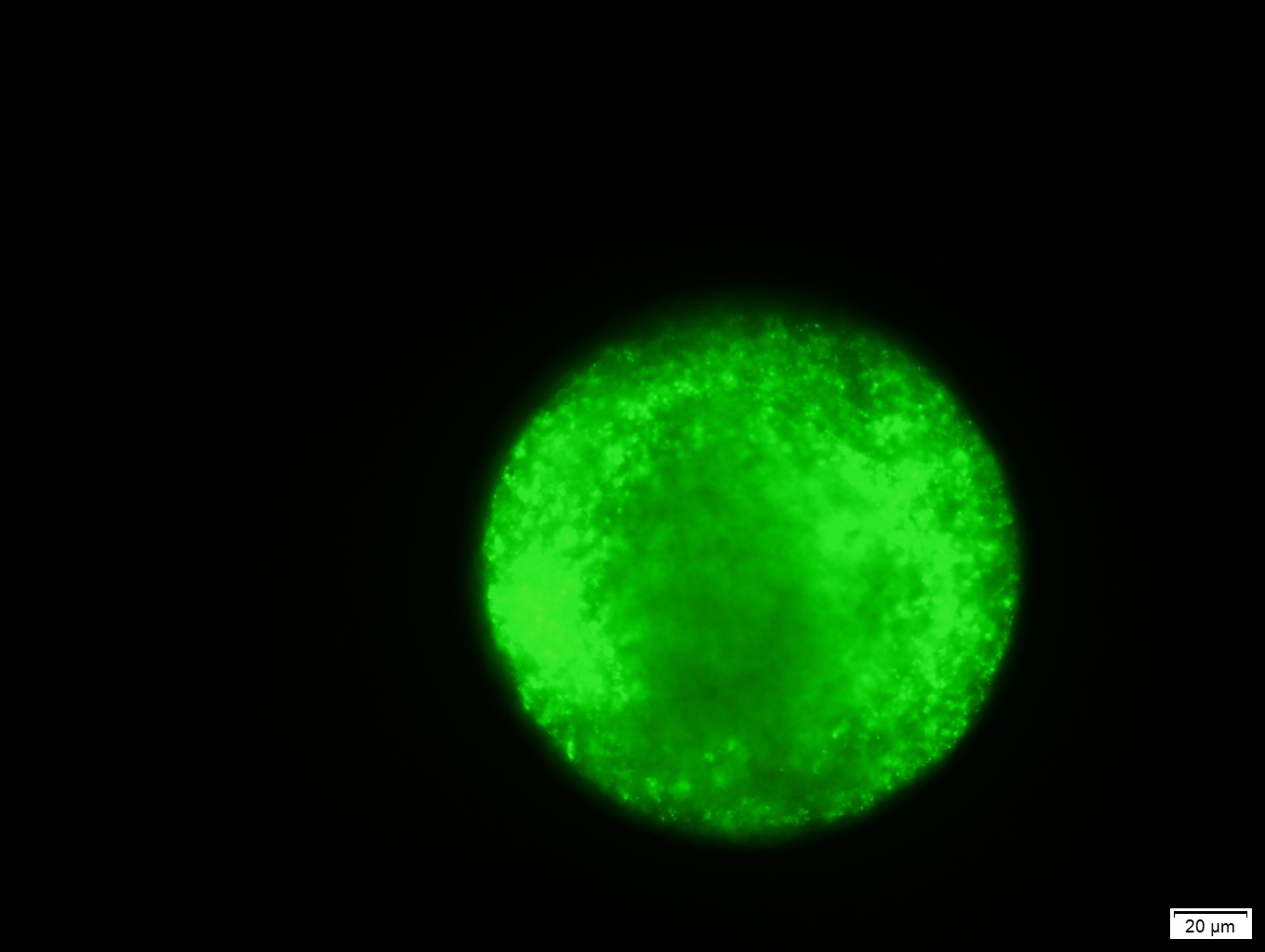

Supplement: Supplementary file 7 [file DataSheet5.ZIP › Figure5í╠/DCA┼¿╢╚ A/P-PDH-B (1).jpg]

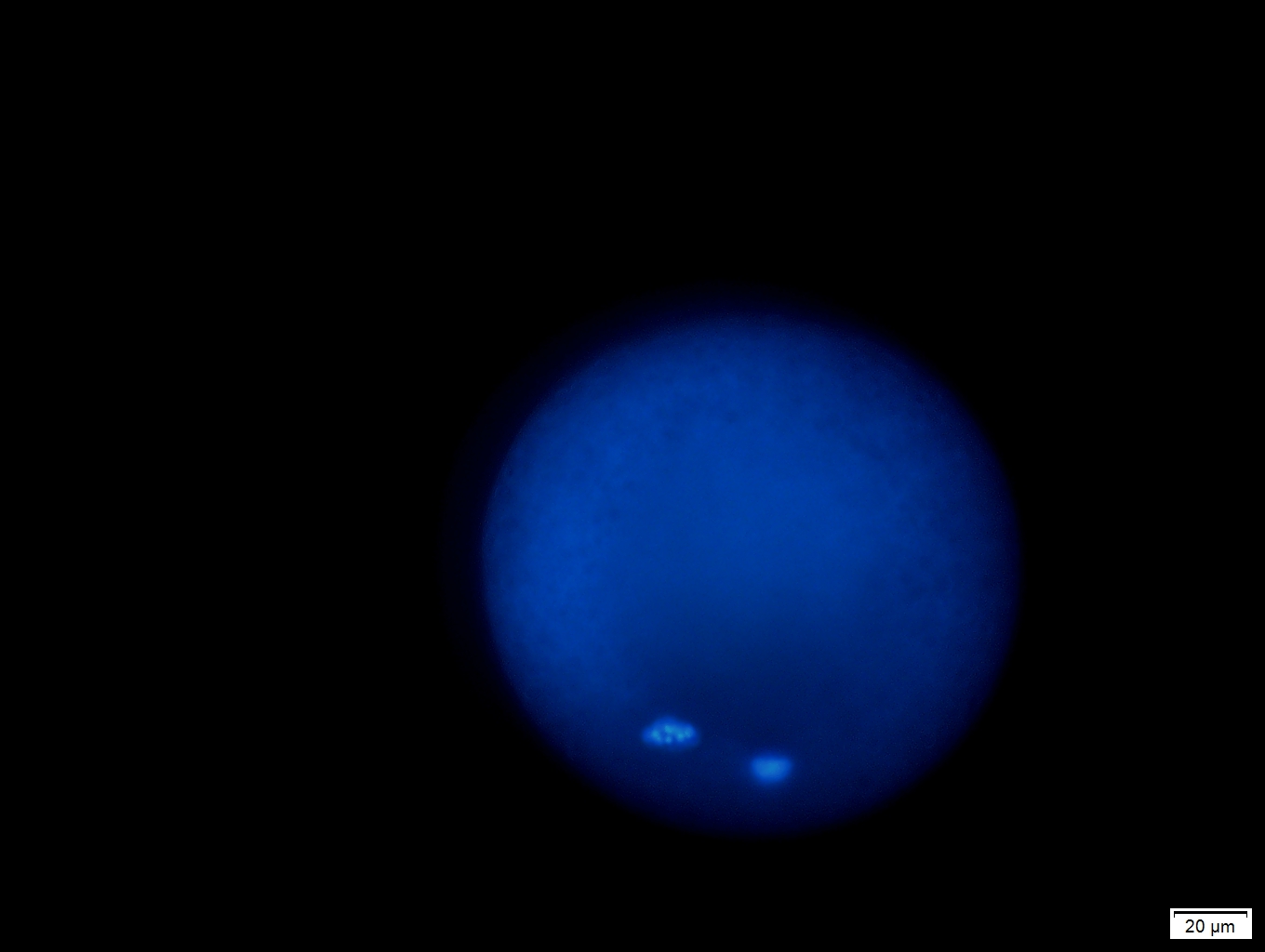

Supplement: Supplementary file 7 [file DataSheet5.ZIP › Figure5í╠/DCA┼¿╢╚ A/P-PDH-B (2).jpg]

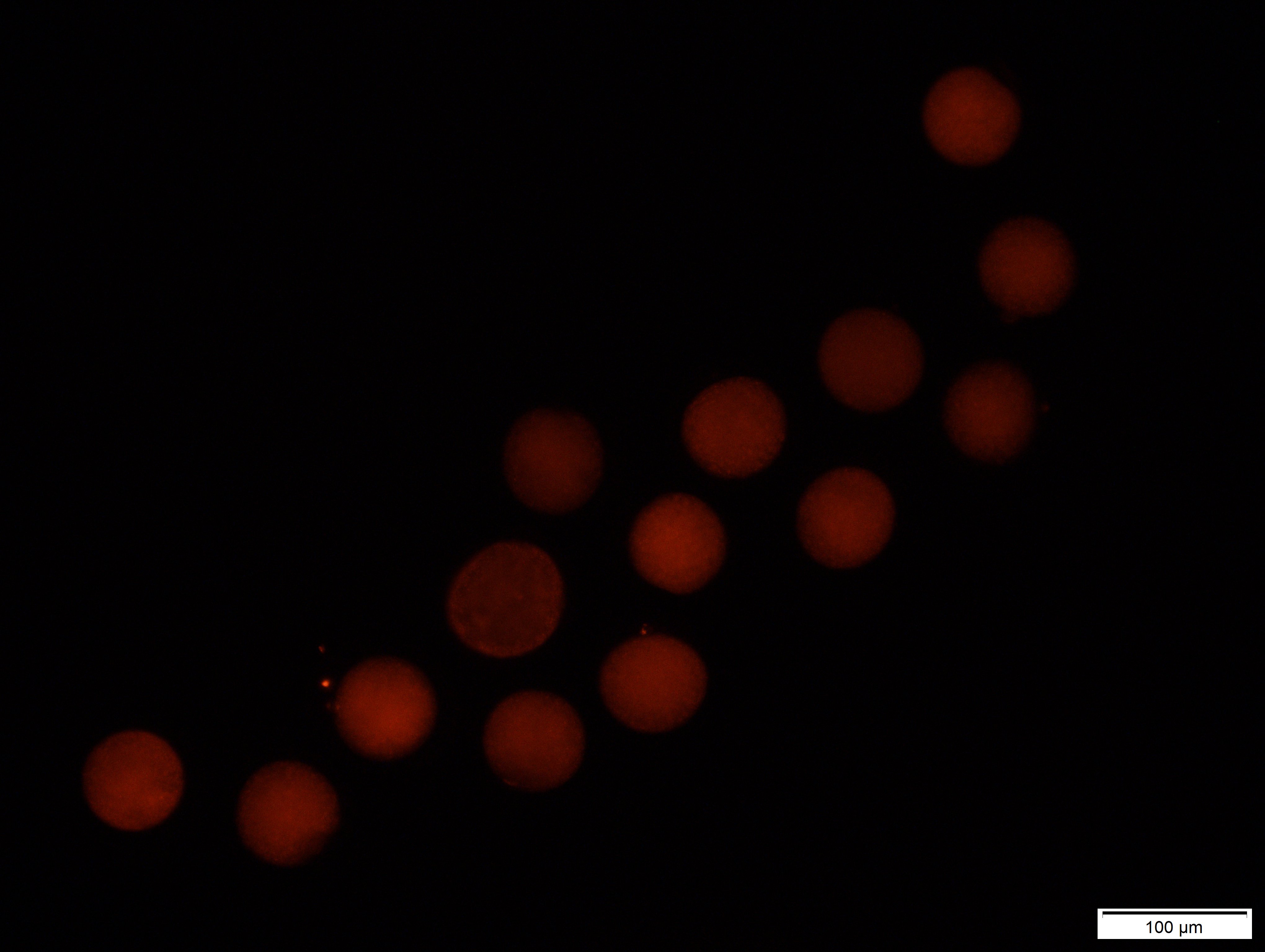

Supplement: Supplementary file 7 [file DataSheet5.ZIP › Figure5í╠/DHE/DHE-B2.jpg]

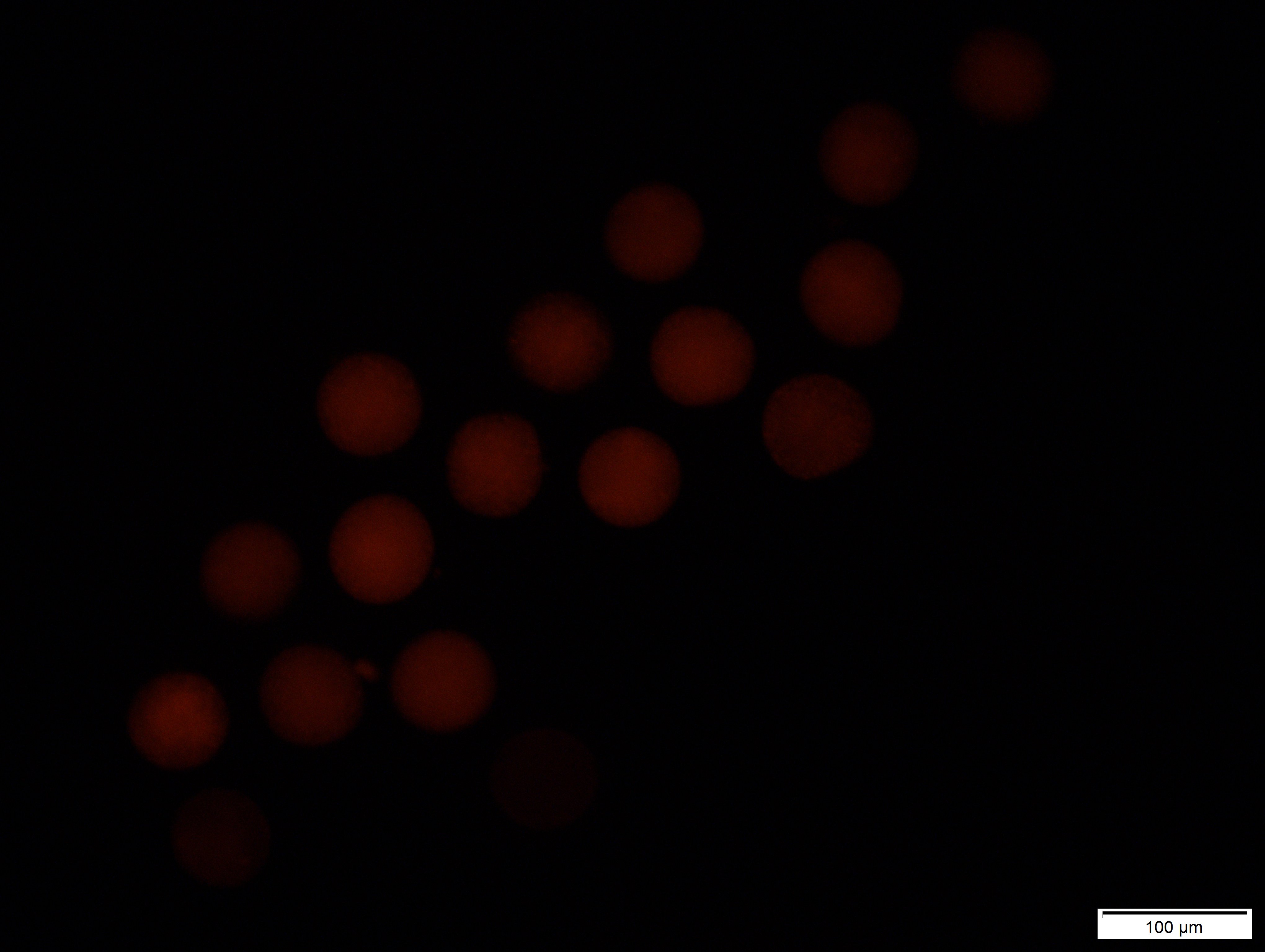

Supplement: Supplementary file 7 [file DataSheet5.ZIP › Figure5í╠/DHE/DHE-D2.jpg]

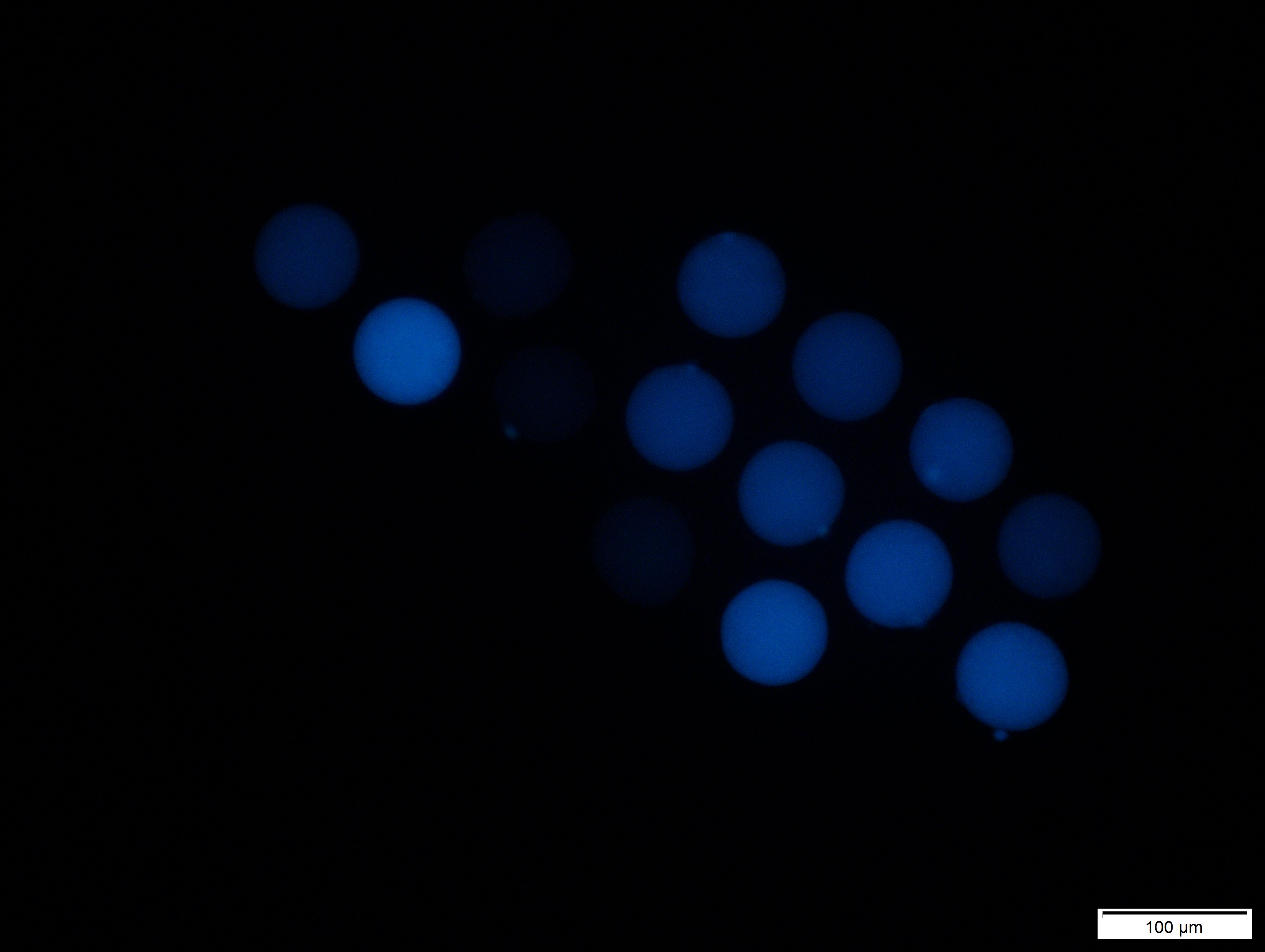

Supplement: Supplementary file 7 [file DataSheet5.ZIP › Figure5í╠/GSH/GSH-B1.jpg]

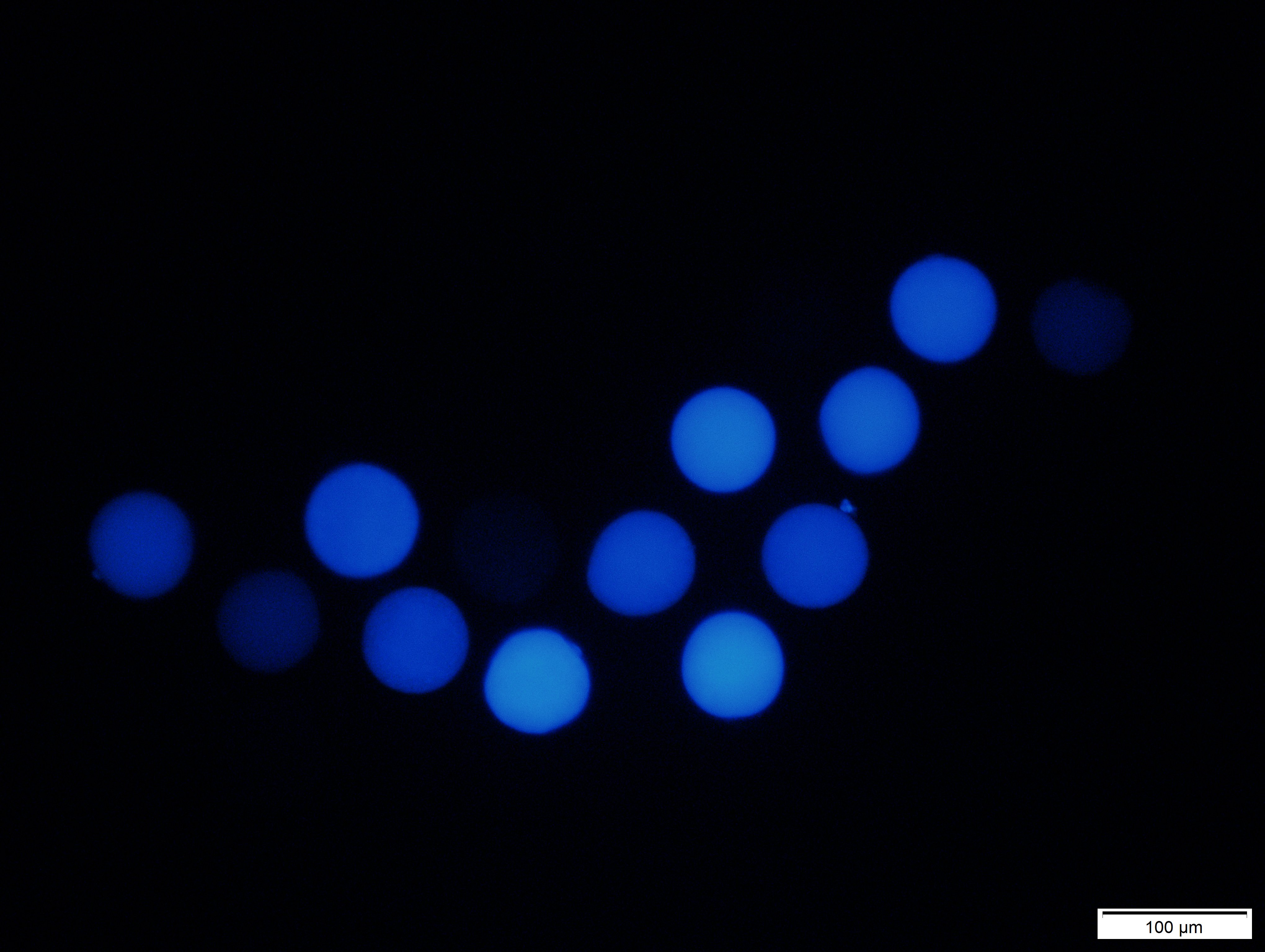

Supplement: Supplementary file 7 [file DataSheet5.ZIP › Figure5í╠/GSH/GSH-D2.jpg]

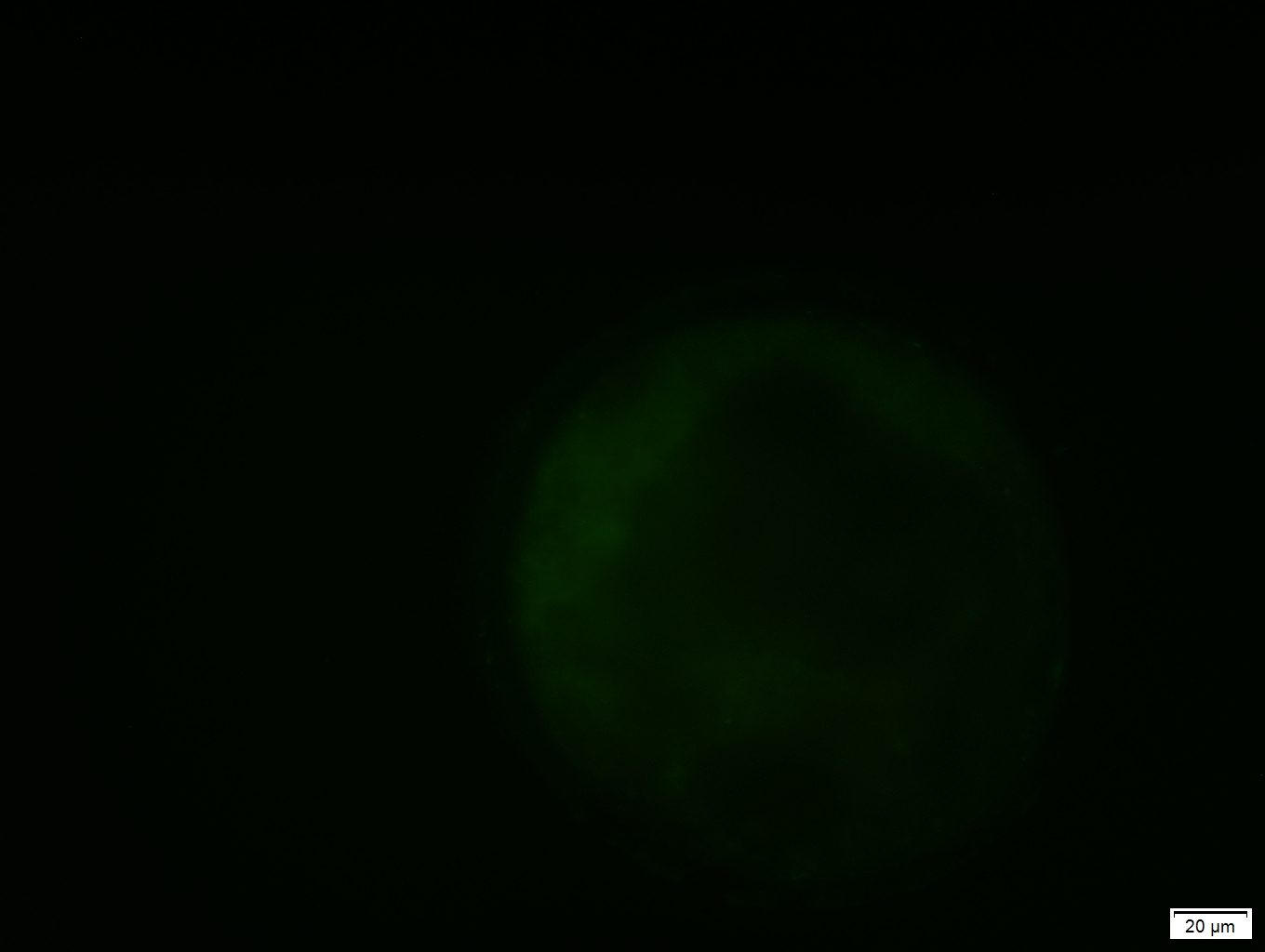

Supplement: Supplementary file 7 [file DataSheet5.ZIP › Figure5í╠/JC-1/BB-J (1).jpg]

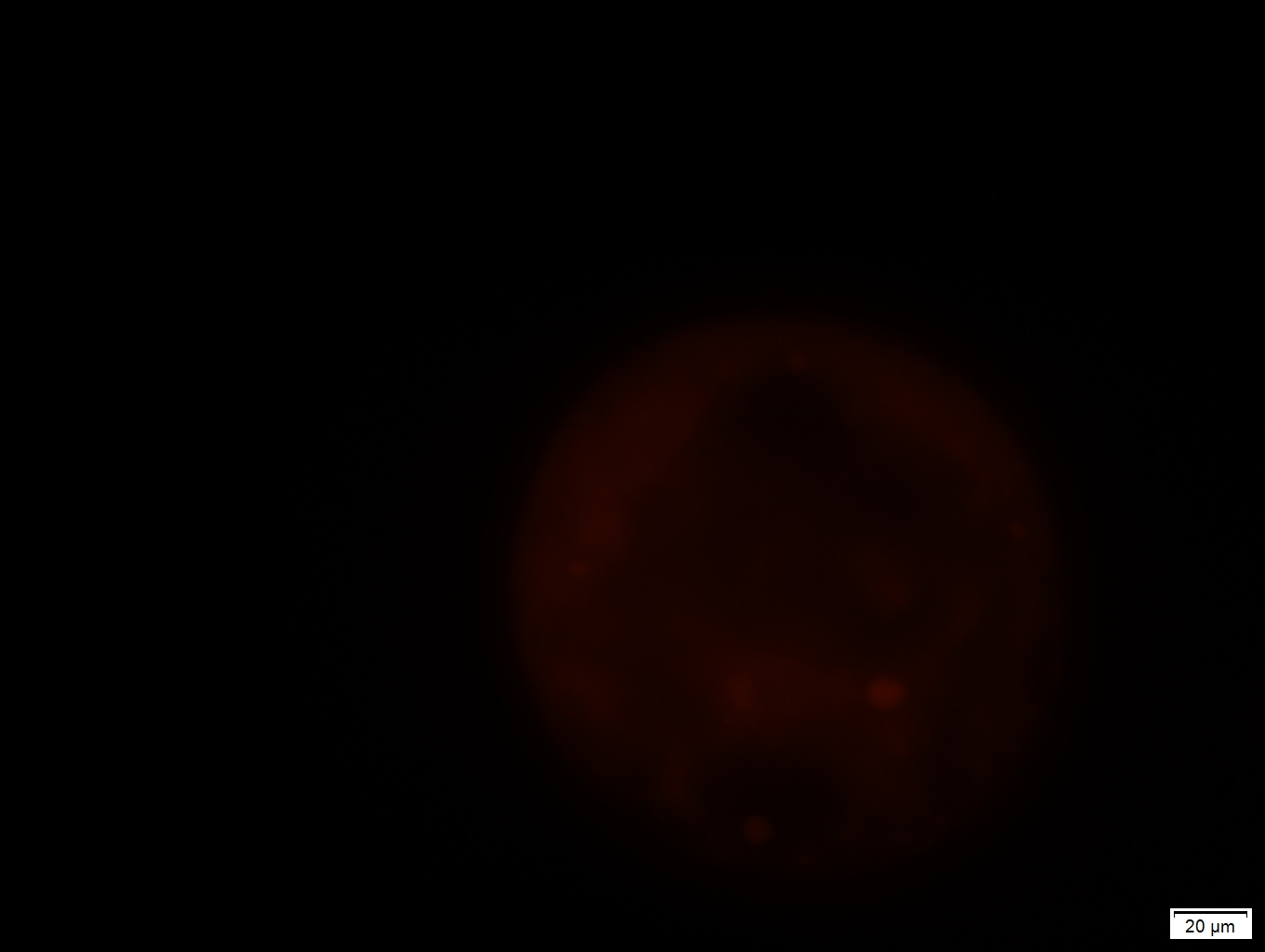

Supplement: Supplementary file 7 [file DataSheet5.ZIP › Figure5í╠/JC-1/BB-J (2).jpg]

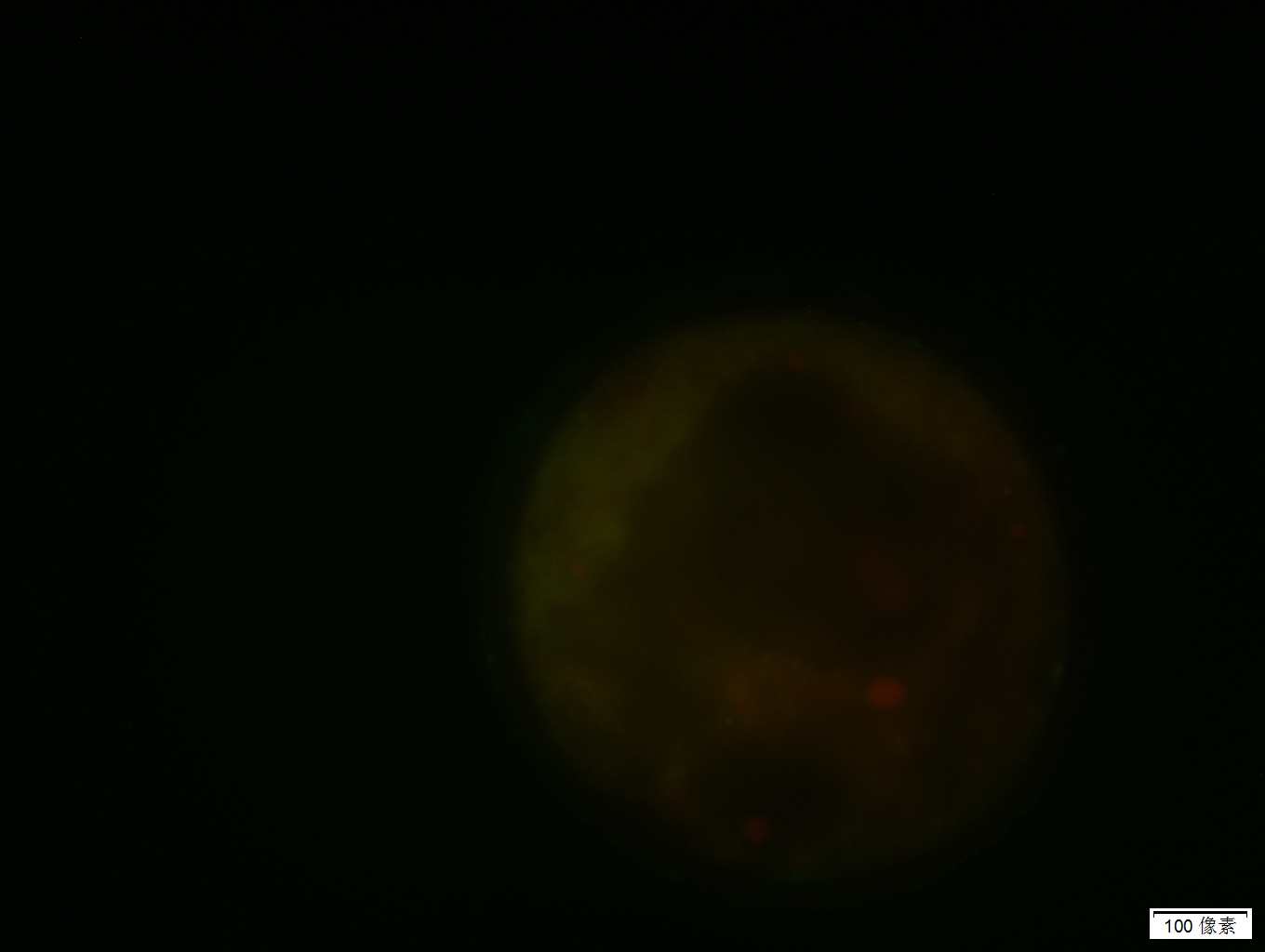

Supplement: Supplementary file 7 [file DataSheet5.ZIP › Figure5í╠/JC-1/BB-J.jpg]

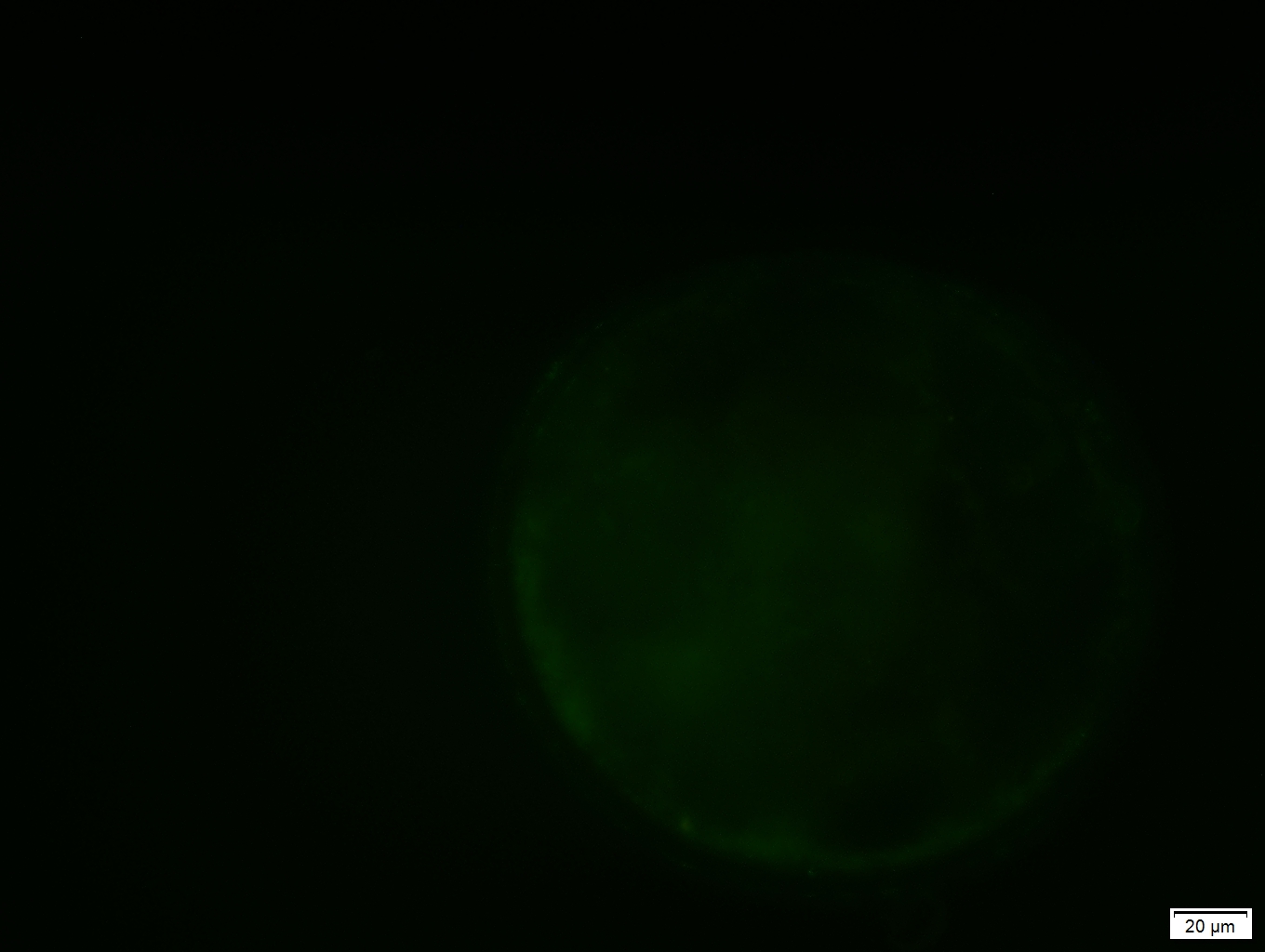

Supplement: Supplementary file 7 [file DataSheet5.ZIP › Figure5í╠/JC-1/DD-J (1).jpg]

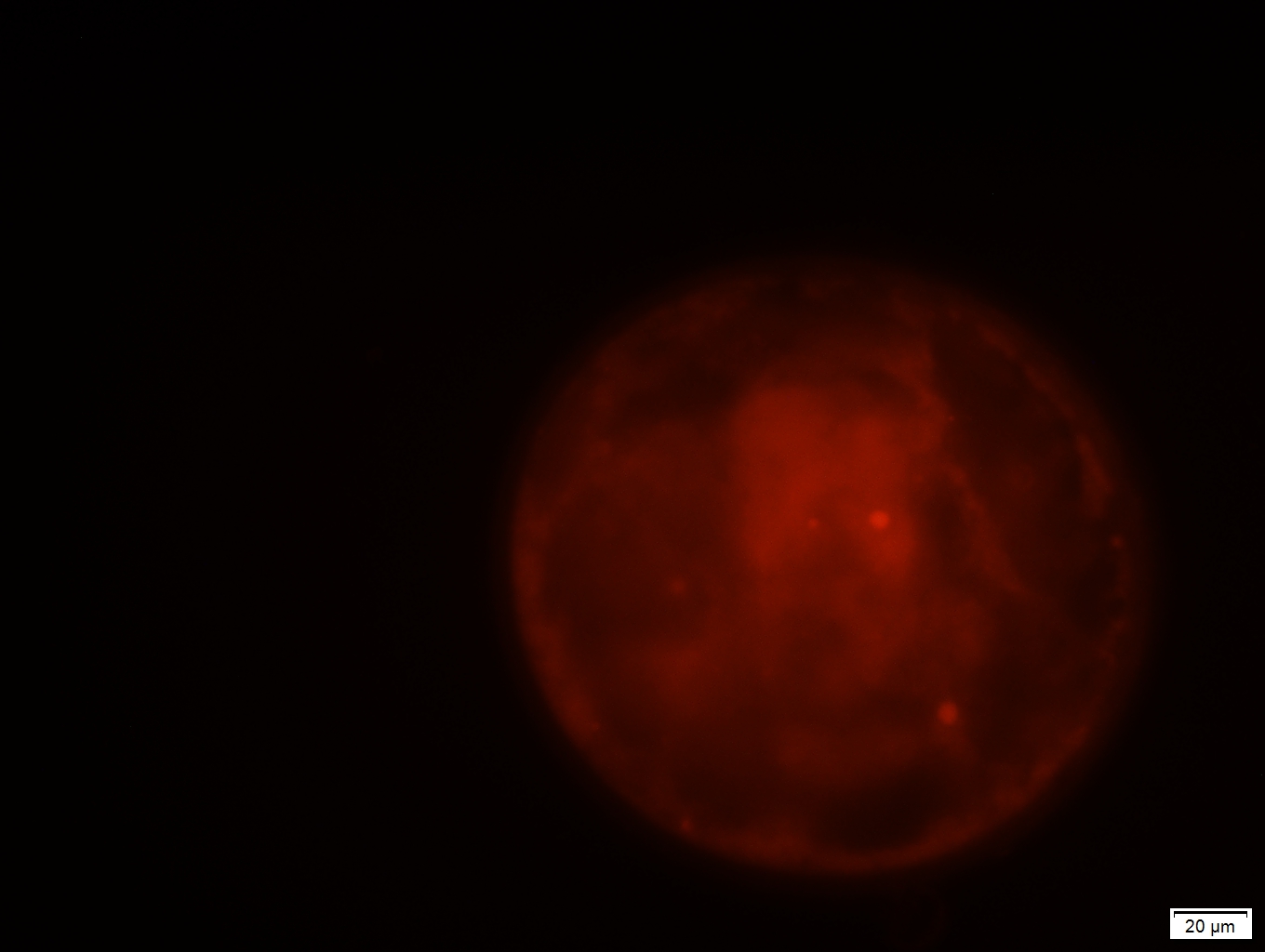

Supplement: Supplementary file 7 [file DataSheet5.ZIP › Figure5í╠/JC-1/DD-J (2).jpg]

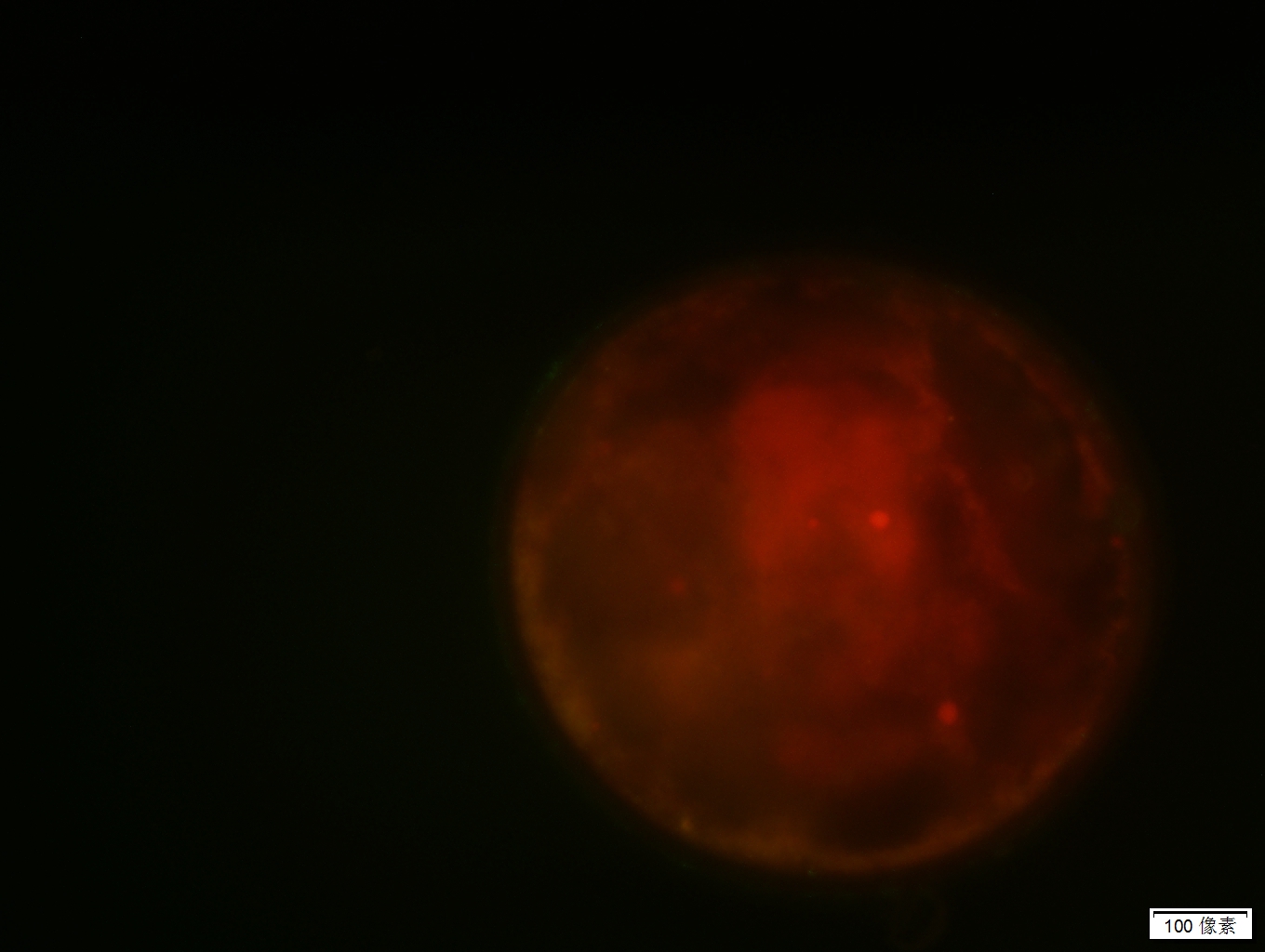

Supplement: Supplementary file 7 [file DataSheet5.ZIP › Figure5í╠/JC-1/DD-J.jpg]

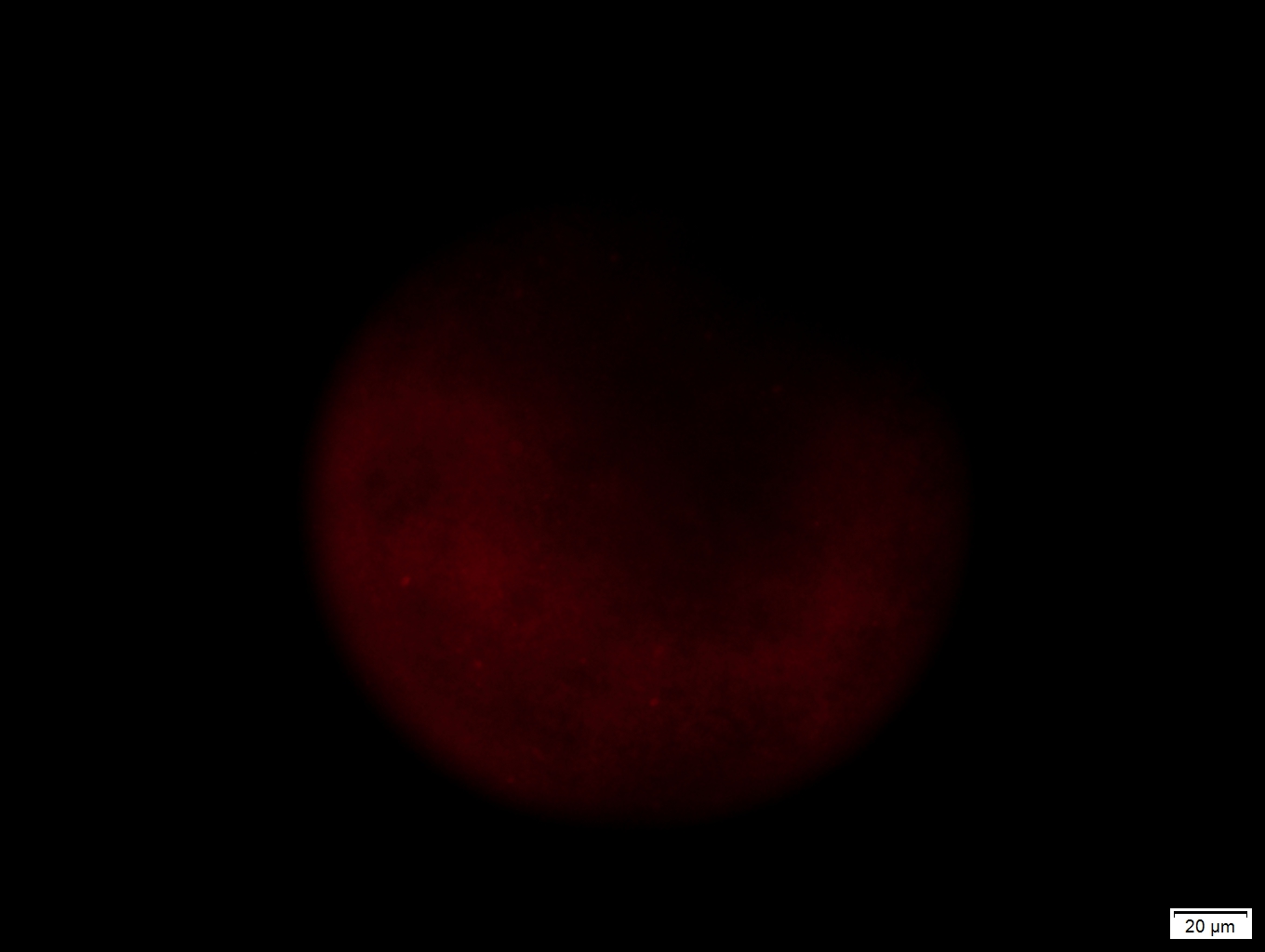

Supplement: Supplementary file 7 [file DataSheet5.ZIP › Figure5í╠/MMP/MMP-BHB (1).jpg]

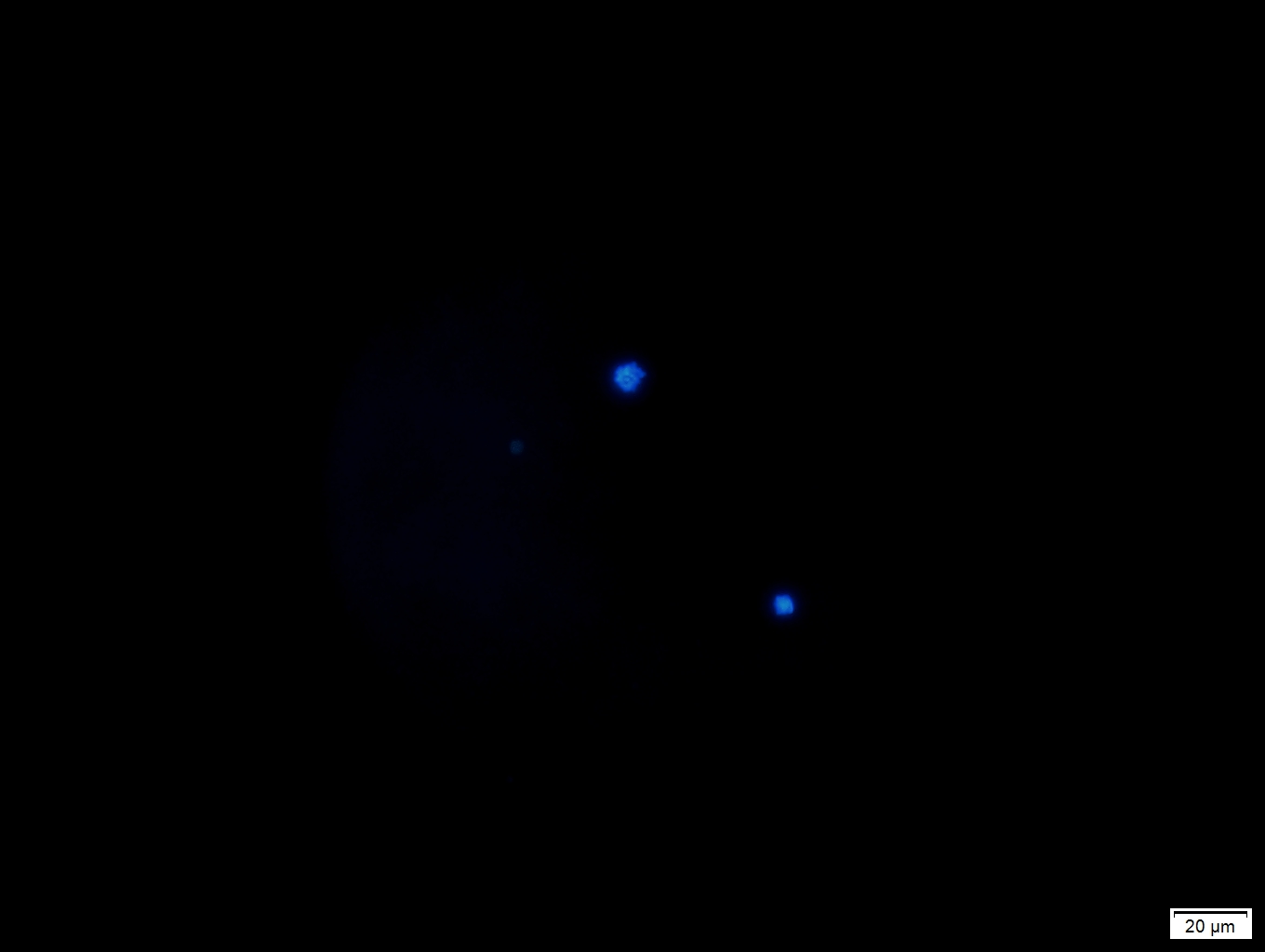

Supplement: Supplementary file 7 [file DataSheet5.ZIP › Figure5í╠/MMP/MMP-BHB (2).jpg]

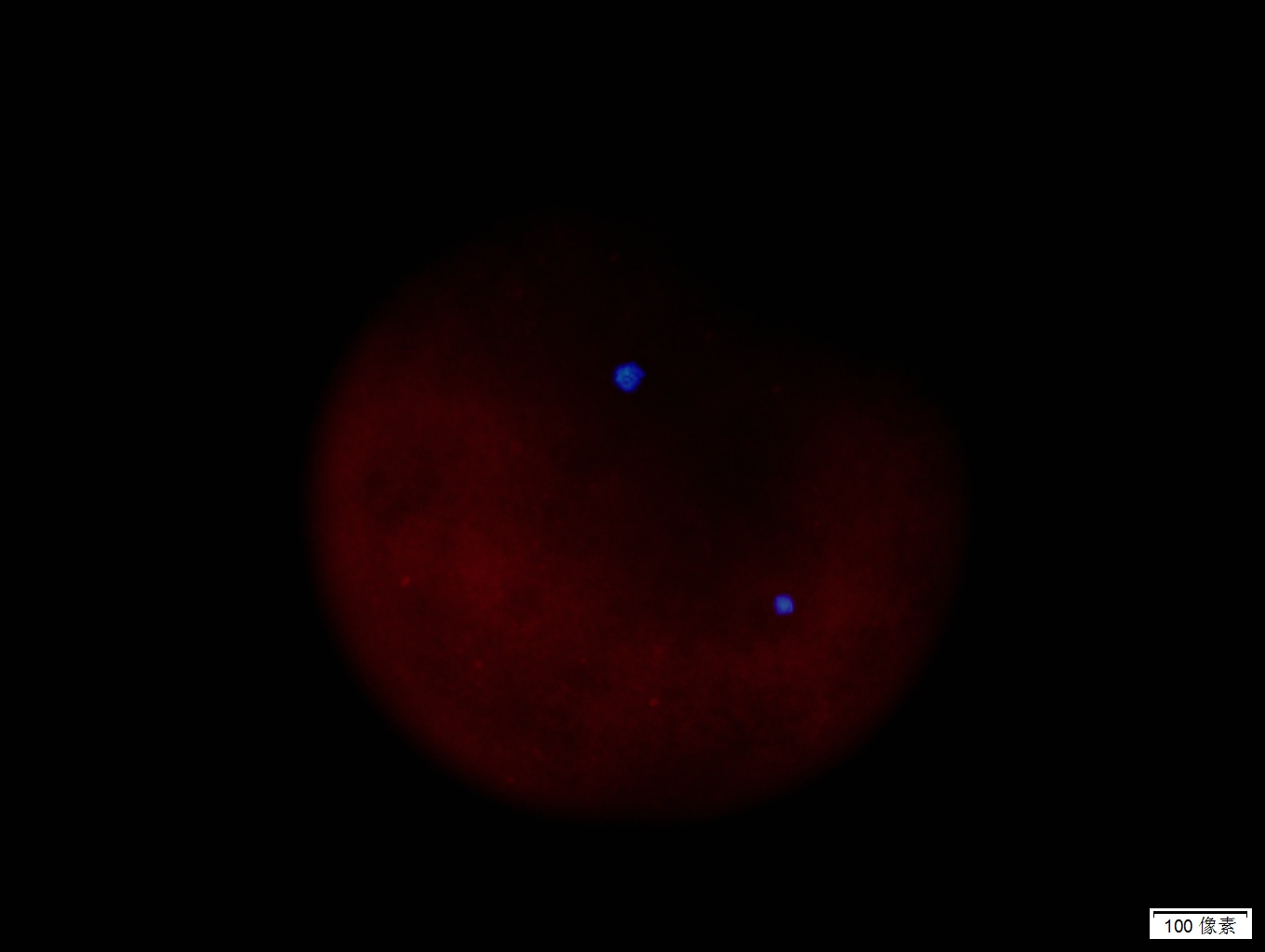

Supplement: Supplementary file 7 [file DataSheet5.ZIP › Figure5í╠/MMP/MMP-BHB.jpg]

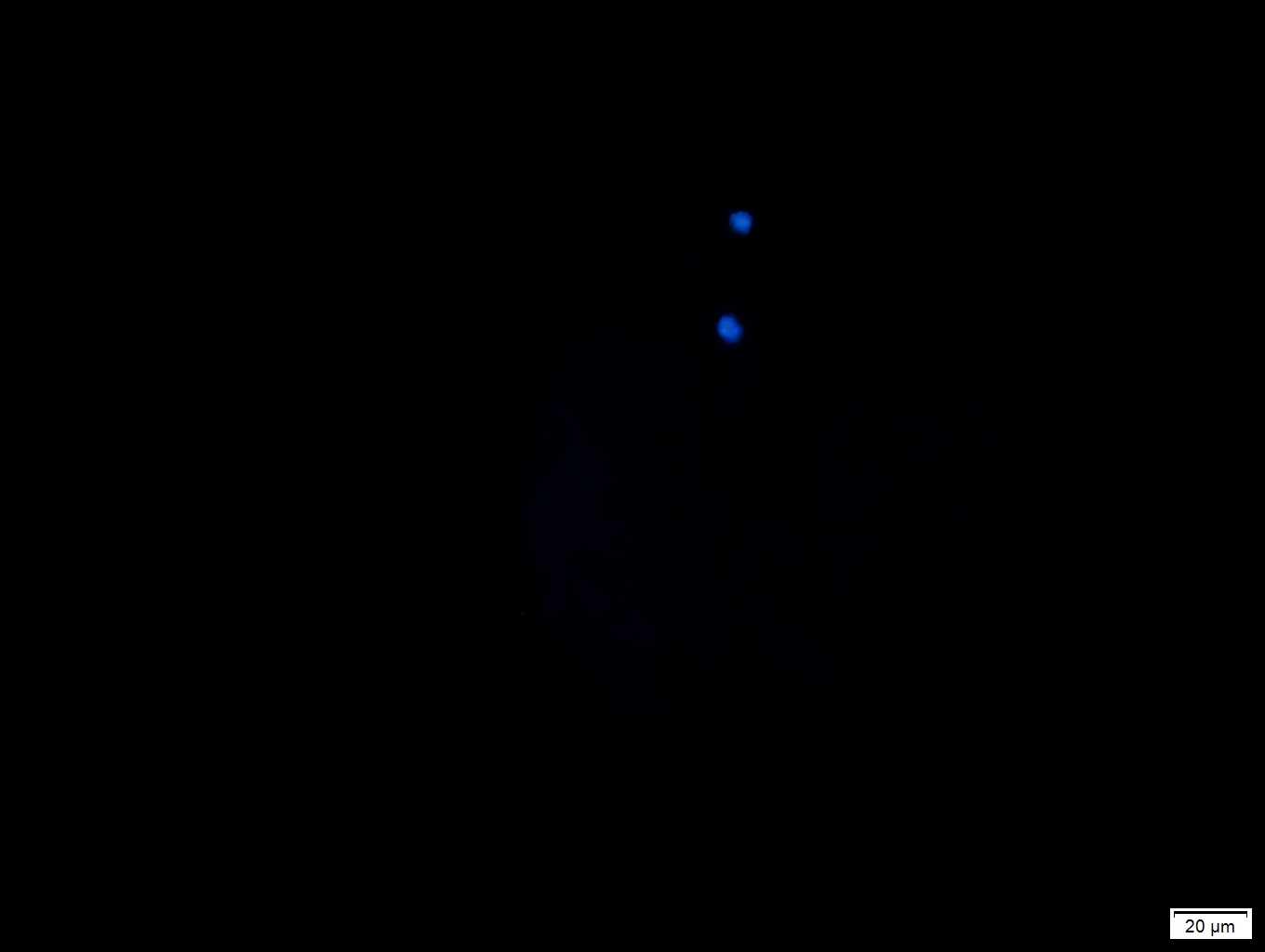

Supplement: Supplementary file 7 [file DataSheet5.ZIP › Figure5í╠/MMP/MMP-DCA (1).jpg]

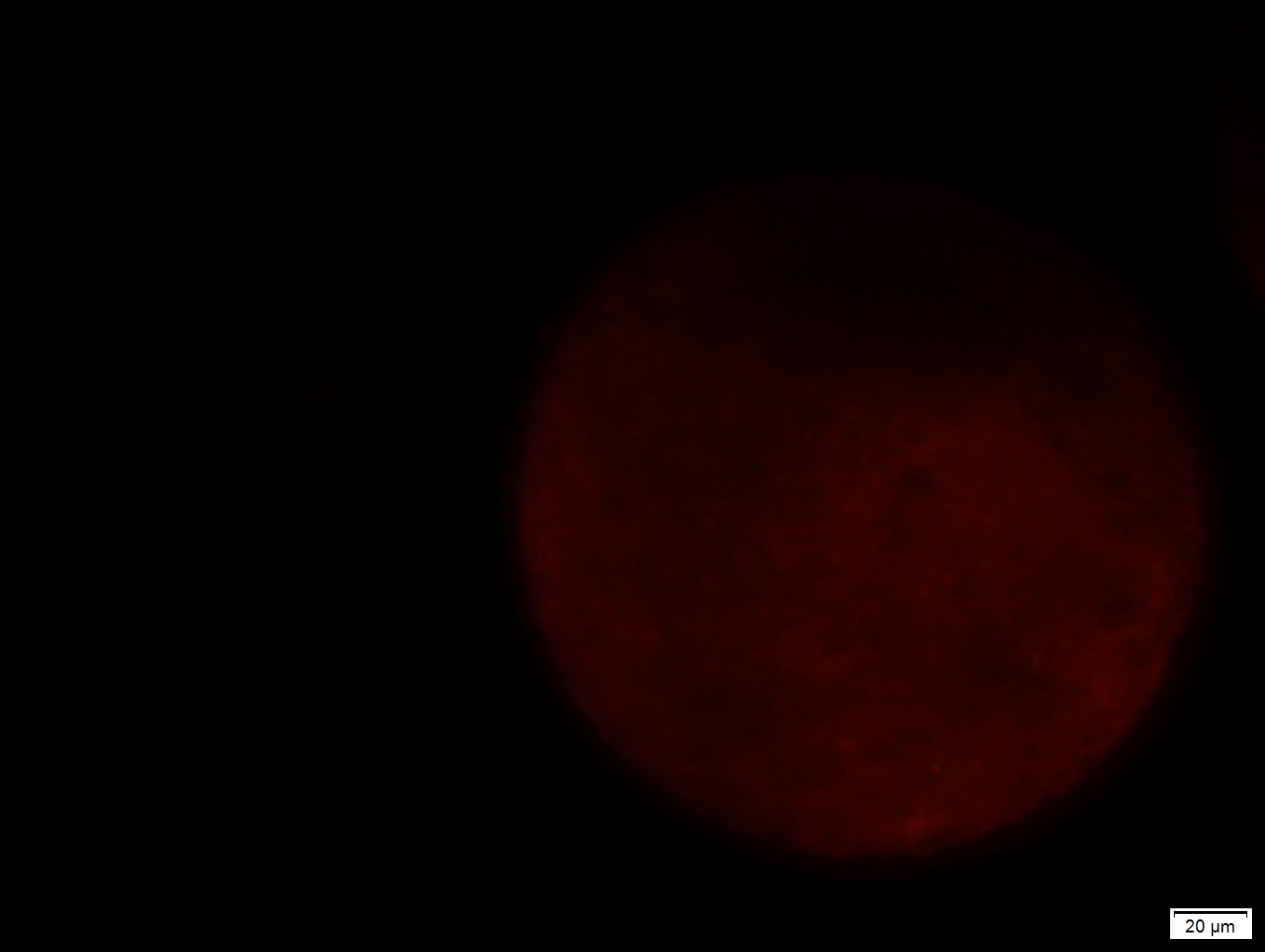

Supplement: Supplementary file 7 [file DataSheet5.ZIP › Figure5í╠/MMP/MMP-DCA (2).jpg]

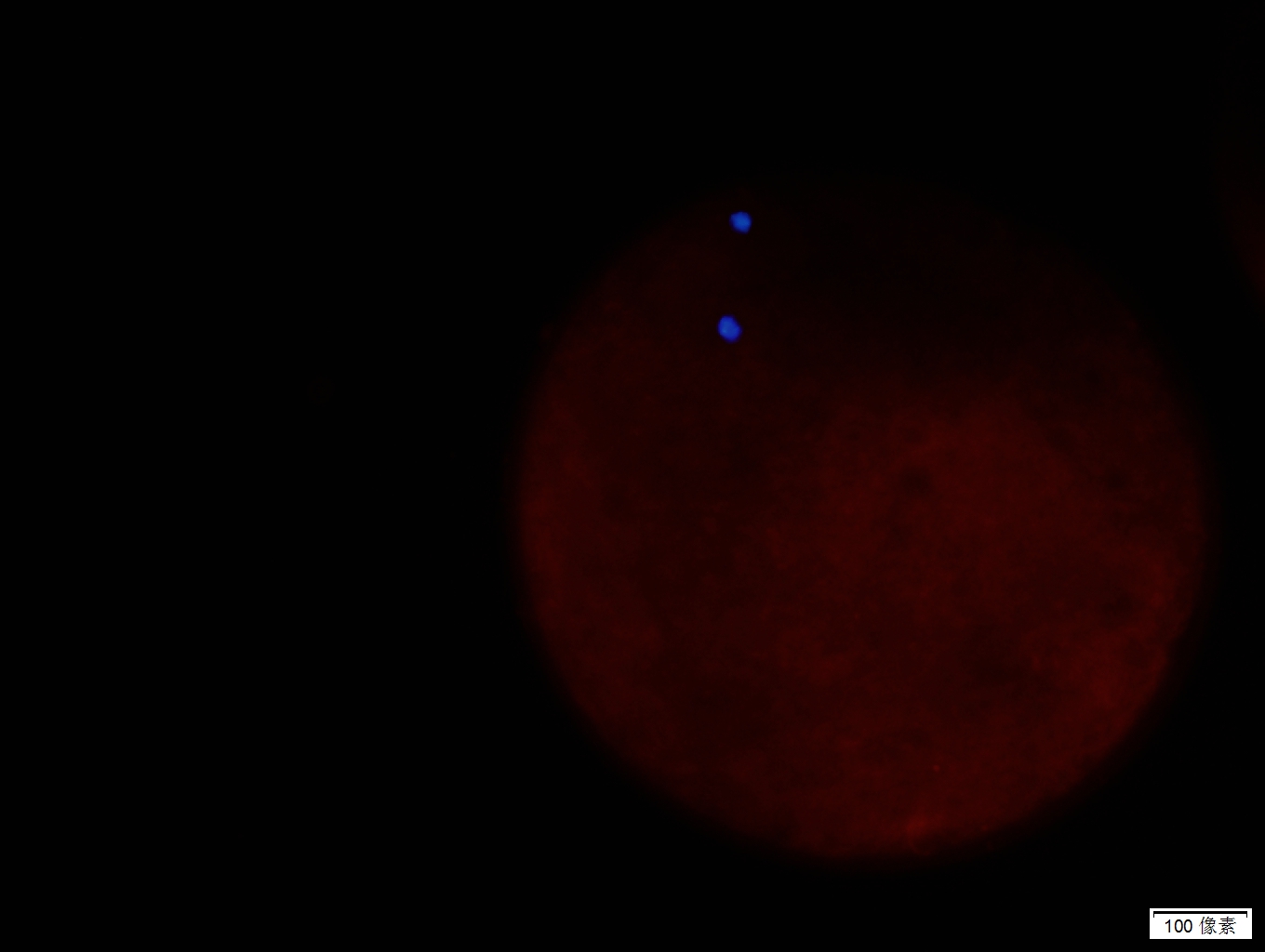

Supplement: Supplementary file 7 [file DataSheet5.ZIP › Figure5í╠/MMP/MMP-DCA.jpg]

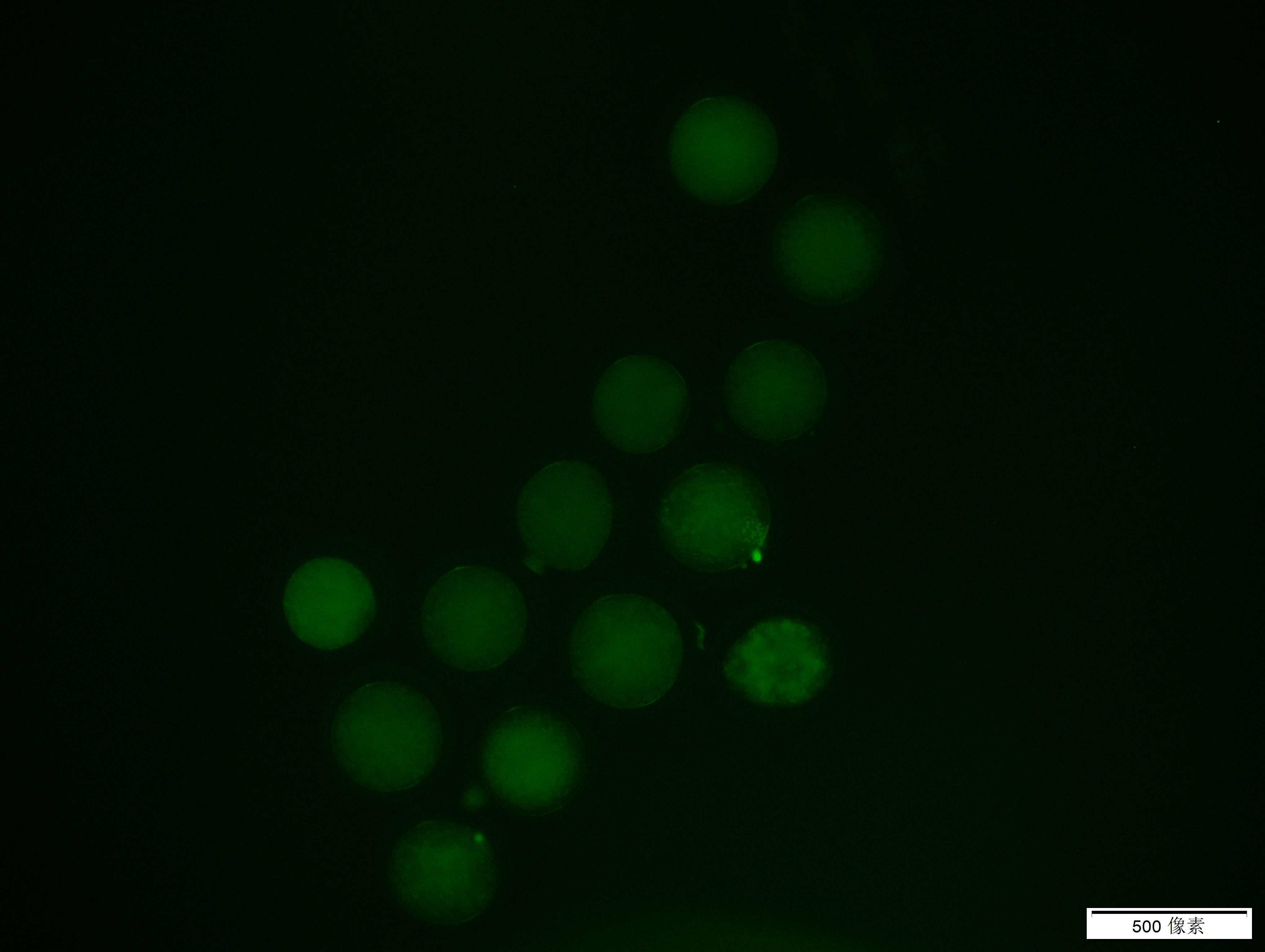

Supplement: Supplementary file 7 [file DataSheet5.ZIP › Figure5í╠/ROS/ROS-B (1).jpg]

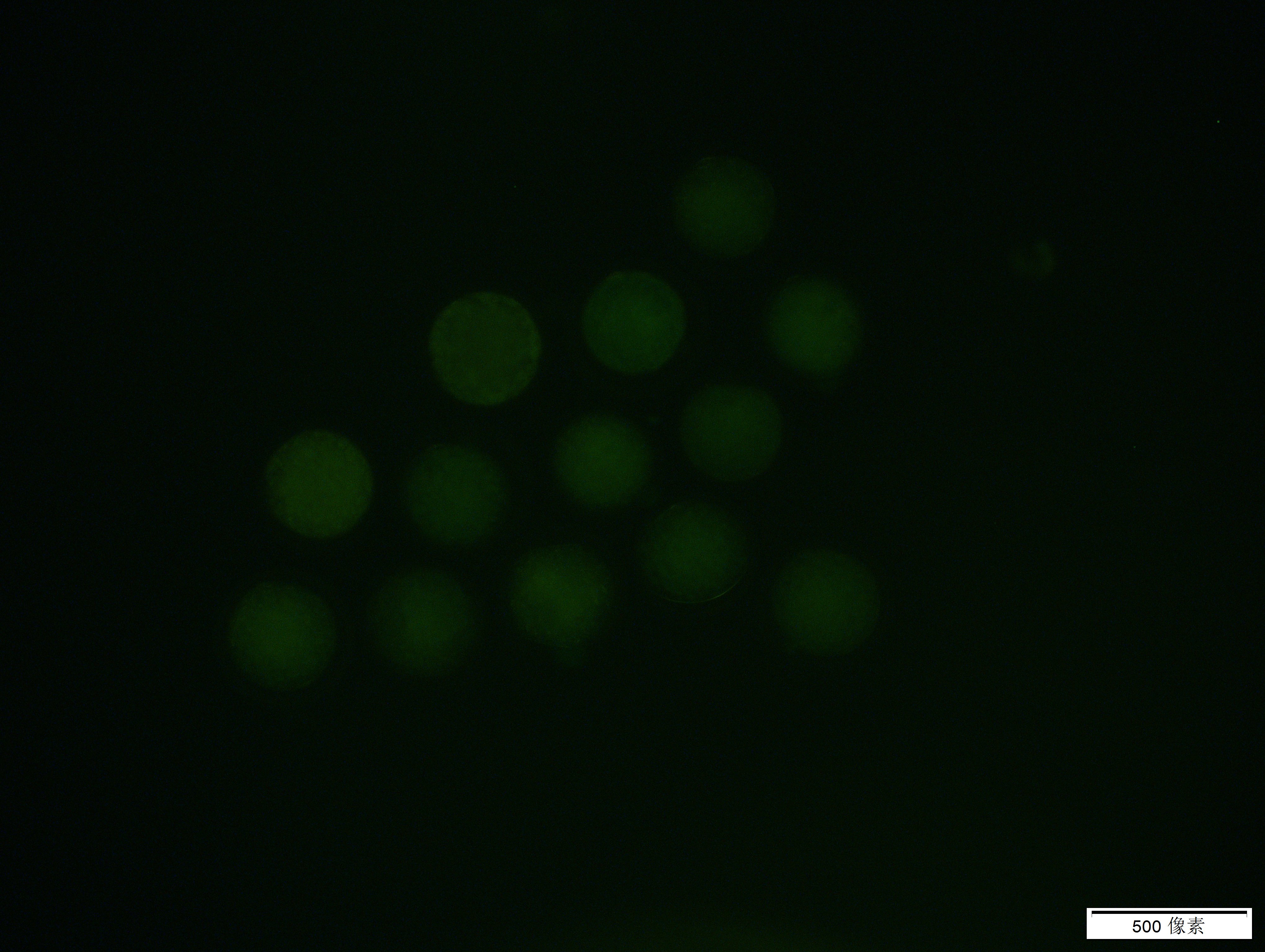

Supplement: Supplementary file 7 [file DataSheet5.ZIP › Figure5í╠/ROS/ROS-D.jpg]
